# Single-cell transcriptomics unveils xylem cell development and evolution

**DOI:** 10.1186/s13059-022-02845-1

**Published:** 2023-01-09

**Authors:** Chia-Chun Tung, Shang-Che Kuo, Chia-Ling Yang, Jhong-He Yu, Chia-En Huang, Pin-Chien Liou, Ying-Hsuan Sun, Peng Shuai, Jung-Chen Su, Chuan Ku, Ying-Chung Jimmy Lin

**Affiliations:** 1 Department of Life Science, National Taiwan University, Taipei, 10617 Taiwan; 2 Genome and Systems Biology Degree Program, National Taiwan University and Academia Sinica, Taipei, 10617 Taiwan; 3 Institute of Plant and Microbial Biology, Academia Sinica, Taipei, 11529 Taiwan; 4 Institute of Plant Biology, National Taiwan University, Taipei, 10617 Taiwan; 5 Department of Forestry, National Chung Hsing University, Taichung, 40227 Taiwan; 6 College of Forestry, Fujian Agriculture and Forestry University, Fuzhou, 350002 China; 7 Department of Pharmacy, National Yang Ming Chiao Tung University, Taipei, 11221 Taiwan

**Keywords:** Single-cell RNA sequencing, Xylem, Libriform fiber, Vessel element, Tracheid, Ray parenchyma cell, Wood formation, Cross-species comparison, Tissue evolution, Trait reversal

## Abstract

**Background:**

Xylem, the most abundant tissue on Earth, is responsible for lateral growth in plants. Typical xylem has a radial system composed of ray parenchyma cells and an axial system of fusiform cells. In most angiosperms, fusiform cells comprise vessel elements for water transportation and libriform fibers for mechanical support, while both functions are performed by tracheids in other vascular plants such as gymnosperms. Little is known about the developmental programs and evolutionary relationships of these xylem cell types.

**Results:**

Through both single-cell and laser capture microdissection transcriptomic profiling, we determine the developmental lineages of ray and fusiform cells in stem-differentiating xylem across four divergent woody angiosperms. Based on cross-species analyses of single-cell clusters and overlapping trajectories, we reveal highly conserved ray, yet variable fusiform, lineages across angiosperms. Core eudicots *Populus trichocarpa* and *Eucalyptus grandis* share nearly identical fusiform lineages, whereas the more basal angiosperm *Liriodendron chinense* has a fusiform lineage distinct from that in core eudicots. The tracheids in the basal eudicot *Trochodendron aralioides*, an evolutionarily reversed trait, exhibit strong transcriptomic similarity to vessel elements rather than libriform fibers.

**Conclusions:**

This evo-devo framework provides a comprehensive understanding of the formation of xylem cell lineages across multiple plant species spanning over a hundred million years of evolutionary history.

**Supplementary Information:**

The online version contains supplementary material available at 10.1186/s13059-022-02845-1.

## Background

Plant development involves apical and lateral growth. Apical growth elongates the main plant axes [1] by growing upward from shoot apical meristem (SAM) and downward through root apical meristem (RAM) [2]. Extensive studies have revealed the developmental processes of apical growth [3–5]. The division of a single stem cell type in SAM provides a continuous source of daughter cells for organogenetic differentiation horizontally into leaves or flowers and basally into stems, vascular cambium and vascular tissues [3, 4]. In RAM, different stem cell types produce their own daughter cells with distinct cell fates [2, 5]. Lateral growth, which thickens the plant body, is bifacial in nature with a single layer of stem cells/initial cells in vascular cambium growing inward into xylem and outward into phloem [1, 6–8].

The pluripotency of all stem cells from SAM, RAM, and vascular cambium are constantly maintained by organizer cells [2, 8–13]. The organizer cells are surrounded by stem cells in RAM [5], but located at the interface of stem cells and vascular tissue in SAM and vascular cambium [1, 4]. In SAM, organizer cells are generated from basal division of stem cells and then differentiate into vascular tissue [4]. Recent studies in *Arabidopsis* started to reveal the mechanisms behind stem cell proliferation in vascular cambium and differentiation into xylem [6–8, 14]. In vascular cambium, organizer cells are derived from periclinal division of stem cells and differentiate into xylem [1, 8]. The transcription factor (TF) CLASS III HOMEODOMAIN-LEUCINE ZIPPER (HD-ZIP III) promotes xylem identity and cellular quiescence in organizer cells [8], and VNS TFs (VND-, NST/SND-, SMB-related proteins) then induce xylem cell terminal differentiation along with programmed cell death [15–17].

The developmental trajectories of apical roots and shoots have been explored at high resolution using single-cell RNA sequencing (scRNA-seq) [18–30]. The spatial and temporal distribution of individual cells from different stages revealed highly heterogeneous transcriptomes of cell lineages from cell proliferation, identity determination to differentiation. Previous scRNA-seq analyses have focused on plant apical growth in leaves [24, 29], flowers [25], shoot tips [27], and roots [18–23, 28, 30], leaving stems the least studied organ with high-resolution profiling methods. Such organ-level protoplast isolation, a prerequisite for single-cell analyses, has been mainly performed using herbaceous plants, mostly *Arabidopsis*, which tends to yield limited numbers of vascular tissue cells due to the relatively small proportion of vascular tissue in the four organs mentioned above [18–30]. The identified xylem cells in previous scRNA-seq analyses of herbaceous plants were only within the range of 72 to 1205 cells, which would be insufficient for better understanding the cell lineages in xylem development. In addition, *Arabidopsis* lacks ray parenchyma cells under normal growth conditions [31–33] and has immature fiber development without complete programmed cell death [34, 35]. These limitations of herbaceous plants underscore the need for woody plants in a comprehensive study of xylem development.

The stem-differentiating xylem (SDX) of woody plants comprises axial and radial systems that develop from two types of initials in vascular cambium [36]. Fusiform initials differentiate into the axial system, which consists of vessel elements and libriform fibers in most angiosperms and tracheids in gymnosperms and other vascular plants. The radial system contains ray parenchyma cells from ray initials. Together these xylem cells make up the vast majority of mass in woods and are the most abundant tissue on Earth [37]. Over the past hundred years, the development of individual xylem cell types has mostly been studied through anatomical observations, leaving the molecular mechanisms behind their differentiation still largely unknown. In this study, we aim to unravel the developmental processes of plant xylem through scRNA-seq analyses of four woody species representing evolutionary divergent angiosperms. To ensure the yield of xylem single cells, we adapted our established protocol for collecting SDX protoplasts from debarked stems of *Populus trichocarpa* (black cottonwood) [38, 39]. Comparing to previous organ-level scRNA-seq studies, our method exerts tissue-level cell isolation for a better resolution to explore the developmental paths of xylem cells. The other three species include another core eudicot *Eucalyptus grandis*, the basal eudicot *Trochodendron aralioides*, and the magnoliid *Liriodendron chinense*. These selected species encompass the known xylem cell types, including tracheids, vessel elements, libriform fibers, and ray parenchyma cells, allowing us to comprehensively explore xylem development in an evolutionary context.

## Results

### Single-cell and microdissection RNA sequencing reveal SDX cell identities

**Fig. 1.**
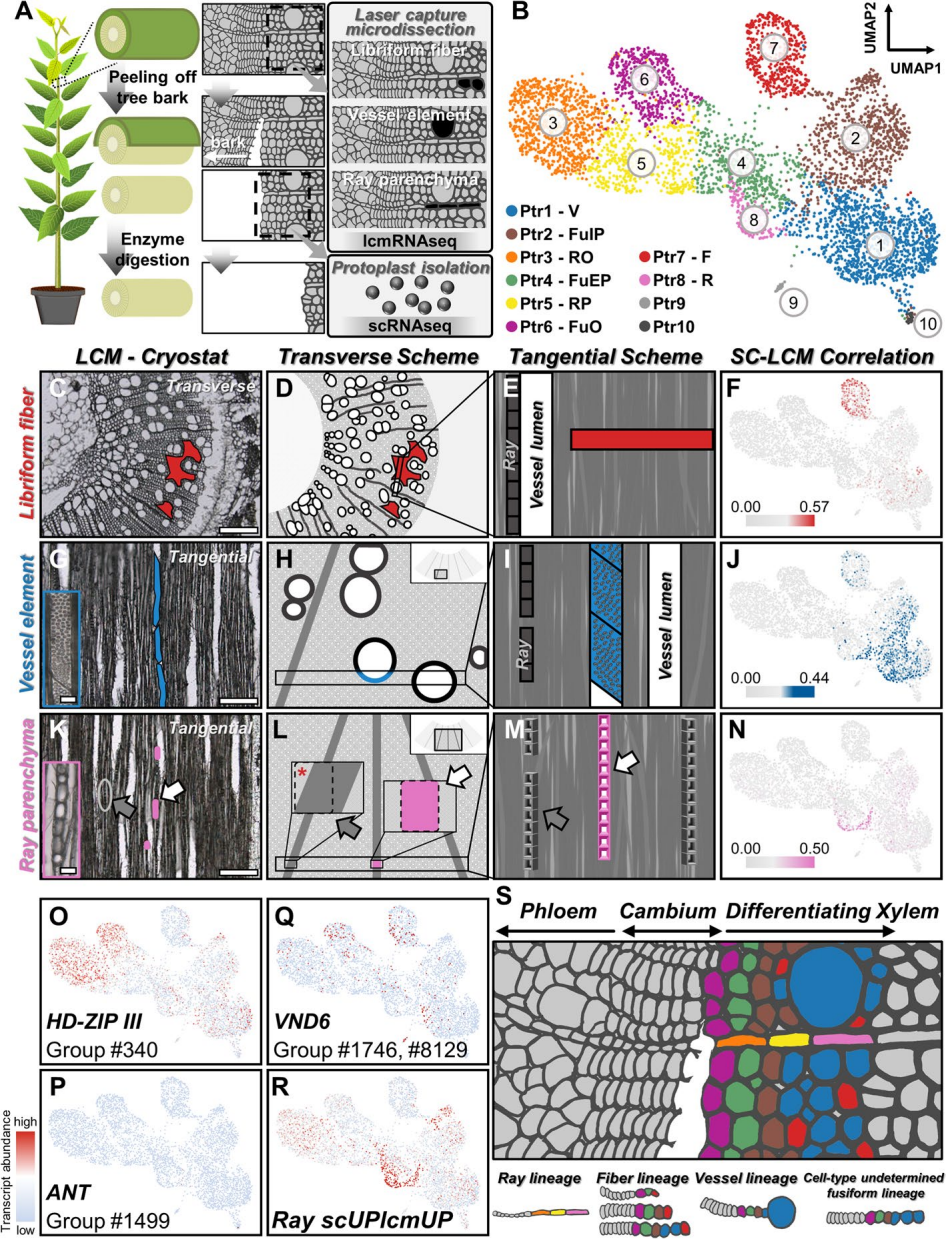
scRNA-seq and lcmRNA-seq reveal cell clusters involved in SDX development. **A** Schematic of the workflow for lcmRNA-seq of libriform fibers, vessel elements, and ray parenchyma cells (shown as black area) and scRNA-seq of SDX protoplasts. For SDX protoplast isolation, the stems of greenhouse-grown *P. trichocarpa* were processed by bark peeling and cell-wall enzyme digestion. **B** Ten cell clusters, Ptr1 to Ptr10, were obtained through unsupervised *K*-means clustering and visualized by UMAP. V, vessel element. FuIP, fusiform intermediate precursor. RO, ray organizer. FuEP, fusiform early precursor. RP, ray precursor. FuO, fusiform organizer. F, libriform fiber. R, ray parenchyma cell. **C–N** LCM for three cell type collection and their transcriptomic correlations to scRNA-seq results. Real transverse or tangential sections of *P. trichocarpa* stems for libriform fiber (red area) (**C**), vessel element (blue area) (**G**), and ray parenchyma cell (pink area) (**K**) collection. Scale bars, 200 μm. The blue and pink rectangles at the left bottom in **G** and **K** are the magnified vessel elements and ray parenchyma cells. Scale bars, 25 μm. The illustrations for three cell type collection from transverse and tangential perspectives (**D**, **E**, **H**, **I**, **L**, **M**). In **E** and **I**, the white box represents a vessel element with empty lumen. In **L**, the pink box with dashed lines is the area for ray parenchyma cell collection. The other dashed line box indicates the potential contamination caused by collecting the ray parenchyma cells not from the middle of a section. Such dashed line box contains dark-gray area as ray parenchyma cells and light-gray area as the neighbor libriform fibers shown as the red asterisk. In **K–M**, the white and gray arrows indicate the collected and avoided ray parenchyma cells, respectively. The transcriptomic correlations of libriform fibers (**F**), vessel elements (**J**), and ray parenchyma cells (**N**) to the single-cell transcriptomes, respectively. **O–R** The transcript abundance of *HD-ZIP III* (**O**), *ANT* (**P**), *VND6* (**Q**), and scUPlcmUP genes in ray parenchyma cells (**R**) in scRNA-seq results. **S** The proposed cell lineages during SDX development shown on a schematic transverse section of *P. trichocarpa*, including ray, fiber, vessel lineages, and a cell-type undetermined fusiform lineage. The SDX protoplast isolation releases around six layers of SDX cells from the stem surface after debarking, so around six layers are labeled with colors. The phloem part removed with tree bark and the inner part (inner than six layers) are labeled as gray. Fusiform organizer (purple), fusiform early precursor (green), fusiform intermediate precursor (brown), vessel element (blue), libriform fiber (red), ray organizer (orange), ray precursor (yellow), and ray parenchyma cell (pink)

We profiled the transcriptomes of 25,166 individual SDX cells from 7 biological replicates of *P. trichocarpa* using two different platforms, 10x Genomics Chromium technology and massively parallel single-cell RNA-seq2.0 (MARS-seq2.0) (Additional file 1: Fig. S1). The identified xylem cell numbers are around 10 to 500 folds higher than those in previous studies on herbaceous plants (Additional file 1: Fig. S2). Using first batch of 10x scRNA-seq results with 4705 cells (Fig. 1A), unsupervised K-means clustering divides the cells into 10 clusters Ptr1 to Ptr10 (Additional file 1: Fig. S3A), which are visualized using Uniform Manifold Approximation and Projection (UMAP) (Fig. 1B). Current definitions of SDX cell types are mainly based on anatomical observations [36, 40], and no suitable marker genes are available for assigning cell types to the scRNA-seq cell clusters. To correlate morphology and transcriptomes, we established strategies of laser capture microdissection (LCM) coupled with RNA sequencing (lcmRNA-seq) for three cell types. Transverse sections were used to collect libriform fibers (Additional file 1: Fig. S4), with the laser cutting pathways avoiding vessel elements and ray parenchyma cells (red area in Fig. 1C–E, and Additional file 1: Fig. S5). Tangential sections were used for collecting vessel elements and ray parenchyma cells (Fig. 1G–I, K–M, Additional file 1: Fig. S4, S6). For vessel elements, we only cut the cells with obvious and intact pitted cell wall (blue area in Fig. 1G–I and Additional file 1: Fig. S6A–C). For ray parenchyma cells, we only collected cells located at the center of each tangential section (pink area in Fig. 1K–M, and Additional file 1: Fig. S6D–H), avoiding ray parenchyma cells on both sides that could not be easily separated from neighbor cells (Fig. 1K–M).

To identify the cell types of the scRNA-seq cell clusters, we examined the transcriptomic correlation between each cell cluster from scRNA-seq and each cell type from lcmRNA-seq. The cell clusters Ptr7 (red), Ptr1 (blue), and Ptr8 (pink) show the highest correlation to libriform fibers, vessel elements, and ray parenchyma cells, respectively (Fig. 1F, J, N, Additional file 1: Fig. S7). We also found the highest numbers of differentially expressed genes (DEGs) upregulated in both transcriptomes of these cell clusters and their corresponding lcmRNA-seq cell type transcriptomes (scUPlcmUP genes) (Additional file 1: Fig. S8).

We found that the scUPlcmUP genes of libriform fibers (Ptr7) are heavily enriched with secondary cell wall (SCW) biosynthesis genes, especially those for monolignol biosynthesis (Additional file 1: Fig. S9A–N, S10, Additional file 2). Ray parenchyma cells (Ptr8) also have moderate expression of monolignol biosynthesis genes (Additional file 1: Fig. S10, Additional file 2), consistent with previous observations that ray parenchyma cells in *Picea* also contribute to lignification [41]. Vessel elements barely express monolignol biosynthesis genes (Additional file 1: Fig. S10, Additional file 2), supporting the good-neighbor or post-mortem hypothesis [42, 43] that the lignification of vessel elements is non-cell-autonomous. The exclusive expression of previously reported libriform fiber marker genes, *IRX15-L* (*PdDUF579-9*) and *ASPARTIC PROTEASE 66* (*PtAP66*) [44], in the libriform fiber cell cluster (Ptr7) provide further evidence for the cell type annotation (Additional file 1: Fig. S11). Expansins are the proteins that loosen cell wall to promote cell expansion [45, 46], and their most highly expressed gene, *Potri.001G240900* (Additional file 1: Fig. S12), is significantly enriched in vessel elements (Additional file 1: Fig. S9O). The highest expansin transcript abundance would facilitate the enormous requirement for radial cell wall expansion of vessel elements, the cells with the widest cell lumen among three cell types. Furthermore, we found many photosynthesis-related genes among the scUPlcmUP genes in ray parenchyma cells (Additional file 1: Fig. S9P–W), which agrees with previous observations that ray parenchyma cells possess chlorophyll-containing plastids to carry out woody tissue photosynthesis [47–50]. Since the terminal stage of xylem cell differentiation represents the dead xylem cells, it would be impossible to profile the transcriptome of “mature” xylem cells. The annotated libriform fibers, vessel elements, and ray parenchyma cells represent the cells with determined cell types and under differentiation toward the mature stage. By integrating scRNA-seq–lcmRNA-seq correlation, scUPlcmUP gene distribution and known gene functions, we revealed the transcriptomic profiles and single-cell clusters of vessel elements (Ptr1), libriform fibers (Ptr7), and ray parenchyma cells (Ptr8).

### Two distinct lineages of fusiform and ray cell differentiation from organizer cells

The three cell types Ptr1/7/8 at cell-type determined stages were annotated (Fig. 1B), while the rest of the cell clusters remained unidentified (Fig. 1B). All stem cells from SAM, RAM, and vascular cambium are constantly maintained by the organizer cells [2, 14]. Among the HD-ZIP III TFs in *Arabidopsis*, *AtHB8* is sufficient to induce the function of organizer cells derived from vascular cambium to determine xylem identity [8], and HD-ZIP III was reported to participate in interfascicular cambium development in *Populus* [51]. The homologous genes of *AtHB8* in *P. trichocarpa* were mainly expressed in Ptr3 and Ptr6 (Fig. 1O, Additional file 1: Fig. S13A). Since organizer cells were found to locate right next to vascular cambium [8], organizer cells are supposed to be the cells with least differentiating level in the cells isolated from debarked stems. Ptr3 and Ptr6 locate at the farthest position of differentiating libriform fibers/vessel elements/ray parenchyma cells, suggesting the identity of Ptr3 and Ptr6 as organizer cells. During protoplast isolation, the tree bark was peeled off from the stems and removed. The breakage happened in the area between SDX and cambium [38]. Namely, phloem and initials were on the bark side, and organizer cells and SDX remained on the surface of the stems. We detected none or extremely low expression of *ANT* (a stem cell/initial cell marker gene [34]; Fig. 1P, Additional file 1: Fig. S13B) or *PEAR1* (a phloem marker gene [34]; Additional file 1: Fig. S13C) in any of the cell clusters, as expected from the removal of initials and phloem during the debarking step of protoplast preparation [38] (Fig. 1A). The results led to a highly possible developing path as initials differentiating into organizer cells then into xylem cells, and the position of organizer cells, between stem cells/initials, and xylem cells (Fig. 1S), is similar to that in SAM and vascular cambium [2, 14].

Intensive cell wall deposition is a critical indication of xylem development. In other words, xylem development involves a process of thin-wall cells turning into thick-wall cells. We selected the gene sets from the GO terms for cell wall, cellulose and hemicellulose, and plotted the transcript abundance of these gene sets on the UMAP (Additional file 1: Fig. S14A, S14B). The results showed an increasing enrichment of transcript abundance toward libriform fibers and vessel elements (Additional file 1: Fig. S14A, S14B). Organizer cells are derived directly from vascular meristem. We also extracted the gene sets of GO term as meristem, and their transcript abundances were highly enriched in both organizer cell clusters (Additional file 1: Fig. S14C). The enriched transcript abundance in the gene sets of meristem in organizer cells indicated the “start” of the cell lineage on the UMAP, and the transcript abundance in the gene sets of cell wall genes increased toward libriform fibers and vessel elements showing “start to end” on the lineage. A time-course transcriptomic analysis on horizontal growth was performed in a previous study using a series of 20-μm tangential sections across the cambial and SDX regions [52]. We compared our results to the marker genes identified in the previous study and found many genes with consistent expression pattern throughout SDX development (Additional file 1: Fig. S14D, S14E). Thus, the relative transcript abundance of the genes in the GO terms (cell wall and meristem) (Additional file 1: Fig. S14A, S14B, S14C) and the series sections across cambial/SDX regions (Additional file 1: Fig. S14D, S14E) both provide additional layers of time-course evidences for our cell lineages.

The transcript abundance of the respective upregulated DEGs of vessel elements (Ptr1) and ray parenchyma cells (Ptr8) (Additional file 1: Fig. S15, S16) further resolved the cell clusters into two cell lineages based on their differential enrichment. Namely, since vessel elements and ray parenchyma cells belong to fusiform and ray lineages, respectively, the expressing patterns of the upregulated genes in each cell type would indicate their lineages (Additional file 1: Fig. S15, S16). The sequence Ptr6-Ptr4-Ptr2-Ptr1 is the developmental path from fusiform organizer (Ptr6) through fusiform early precursor (Ptr4) and fusiform intermediate precursor (Ptr2) to vessel elements (Ptr1). *VND6* members in the *VNS* family, the master regulators of vessel element differentiation [15], and the expansin gene (*Potri.001G240900*) are also expressed in this fusiform cell lineage (Fig. 1Q, Additional file 1: Fig. S13D, S13F, S15A). In addition, two paralogous MYB transcription factors in poplar, PdMYB156 and PdMYB221, were reported as critical regulators on the development of vessel elements and libriform fibers [53, 54]. The in situ hybridization results showed that these two *MYB*s were highly expressed in both vessel elements and libriform fibers [53]. In our scRNA-seq results, the homologous genes of these two *MYB*s in *P. trichocarpa* showed enriched transcript abundances in the fusiform lineages (Additional file 1: Fig. S17A, S17B). The enriched transcript abundance of upregulated genes and known regulators both support our proposed fusiform lineages. The cell clusters Ptr3 (ray organizer), Ptr5 (ray precursor), and Ptr8 (ray parenchyma) form the other cell lineage (Fig. 1B, R, Additional file 1: Fig. S13E, S15B), which is derived from ray initials. The nomenclature of each cell cluster basically represents three groups of cells: (i) cells of such cell type, (ii) cells under the differentiation toward such cell type, (iii) cells under the differentiation away from such cell type. For example, Ptr6 as fusiform organizer cells would contain three groups of cells: the organizer cells, the cells under the differentiation from initials into fusiform organizer cells, and the organizer cells already under differentiation toward differentiating xylem. Cell differentiation is a continuous process, and cell clustering methods divide cells into discrete clusters based on their transcriptomic profiles. The nomenclature of cell clusters in this study represents our working hypothesis for SDX developmental paths (fusiform lineage: Ptr6-Ptr4-Ptr2-Ptr1; ray lineage: Ptr3-Ptr5-Ptr8) and was adopted to facilitate our discussion of the cell clusters. Our working hypothesis from scRNA-seq results was also supported by the in situ hybridization results from previous studies [44, 53], including *ESK1a* for libriform fibers (Additional file 1: Fig. S17C), *ABR1* for organizer cell location (Additional file 1: Fig. S17D), and *PdMYB156*/*221* for fusiform lineage (Additional file 1: Fig. S17A, S17B).

We notice that fusiform and ray lineages converge at fusiform early precursors (Ptr4) and ray precursors (Ptr5) before entering the cell-type determined stages (Fig. 1B). To validate this observation, we applied another cell clustering and dimensionality reduction pipeline MetaCell [55], which similarly showed that these two cell lineages merged together at the middle stage of differentiation (Additional file 1: Fig. S18). Among all pairwise comparisons of cell cluster transcriptomic profiles, the pair Ptr4-Ptr5 also has very high correlation (Additional file 1: Fig. S19). This striking transcriptomic similarity between fusiform early precursors and ray precursors suggests that fusiform and ray cells would differentiate into a similar state before continuing their unique developmental pathways.

In summary, we uncovered the detailed cell lineages in *P. trichocarpa* SDX (Additional file 1: Fig. S20). Through the clustering of single-cell transcriptomic profiles and identification of different cell types, we provided novel marker genes (Additional file 3) with expression specific to each cell type (Additional file 1: Fig. S21, S22). Using a conserved developmental mechanism involving stem cells and organizer cells comparable to those in SAM and RAM, xylem fusiform and ray cells exert their differentiation through two distinct trajectories.

### Highly similar transcriptomes among SDX in different internodes

We performed bulk RNA-seq for stem xylem from different internodes, and anatomical analyses were also performed for the examination of xylem developmental stages in *P. trichocarpa* (Additional file 1: Fig. S23). Our anatomical analyses found primary growth (bundle vasculature) above 4th internode (from the top), transition zone between 4th and 5th internodes, and secondary growth below 5th internode (Additional file 1: Fig. S23A, S23B). The stems used for protoplast isolation in *P. trichocarpa* were below 12th internodes to ensure the xylem materials were all from secondary xylem. We collected the xylem samples from five sections of internodes to represent different developmental stages of in secondary xylem (Additional file 1: Fig. S23A). Sections 1 to 5 include 6th to 9th, 10th to 13th, 14th to 17th, 18th to 21st and 26th to 29th internodes, respectively. Six biological replicates were used for the xylem in every section for bulk RNA-seq. In the principal component analysis (PCA) results, the low percentage of PC1 suggests the absence of a dominant factor to differentiate the samples (Additional file 1: Fig. S23C), which demonstrated that the percentage of the variation across different developmental internodes is subtle. Very few differentially expressed genes (DEGs) were detected, with only 12, 6, 11, 11, and 18 DEGs from sections 1 to 5, respectively (Additional file 1: Fig. S23C). Many of these DEG functions are unknown, and the rest of the annotated DEG functions are basically unrelated to xylem development (Additional file 4). Given that these DEGs only correspond to less than 0.1% of all expressed 23,441 genes in secondary xylem of these five sections (Additional file 1: Fig. S23C), the transcriptomic profiles from different internodes are nearly identical. These results suggested that xylem development is highly similar in different internodes with secondary growth and that the developmental lineages discovered in our single-cell datasets are representative of the overall stem-differentiating xylem developmental trajectories.

### Identifying SDX cell types across angiosperms

**Fig. 2.**
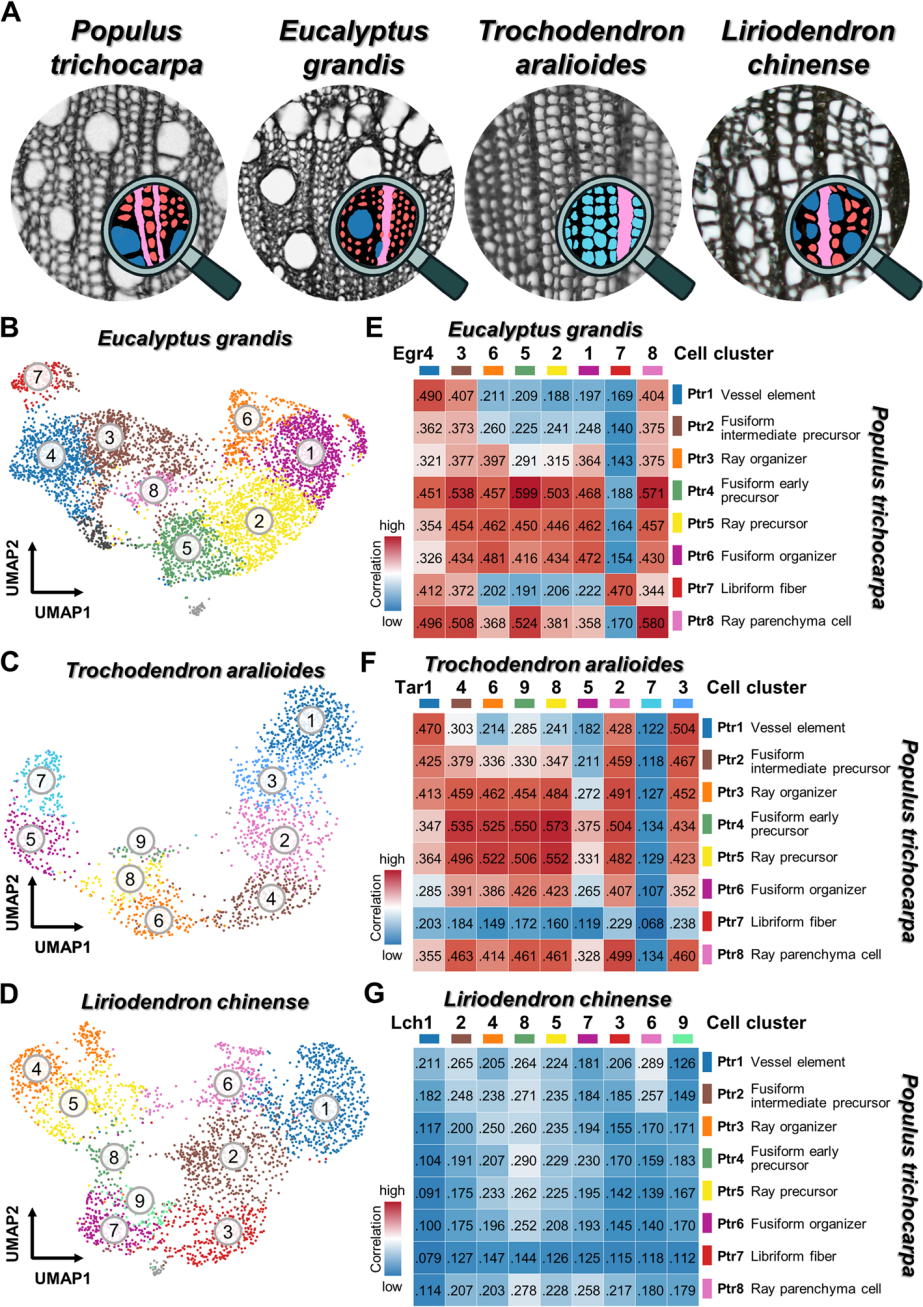
Comparative anatomy and single-cell transcriptomics of SDX in *P. trichocarpa*, *E. grandis*, *T. aralioides*, and *L. chinense*. **A** The SDX anatomy of four woody species. *P. trichocarpa*, *E. grandis*, and *L. chinense* contain libriform fibers (red), vessel elements (blue), and ray parenchyma cells (pink). *T. aralioides* possesses tracheids (cyan) and ray parenchyma cells (pink). **B**–**D** The unsupervised *K*-means clustering and UMAP plots from the SDX scRNA-seq results of *E. grandis* (**B**), *T. aralioides* (**C**), and *L. chinense* (**D**), respectively. **E**–**G** The pairwise correlation analysis of the SDX scRNA-seq results between *P. trichocarpa* and *E. grandis* (**E**), *T. aralioides* (**F**), or *L. chinense* (**G**), respectively. The orders of cell clusters in *E. grandis* (e.g., Egr4-3-6-5-2-1-7-8), *T. aralioides*, or *L. chinense* are corresponding to those in Additional file 1: Fig. S3

To further explore evolutionary conservation and divergence of SDX development in angiosperms, we performed scRNA-seq of SDX cells in three additional woody species, including *E. grandis*, *T. aralioides* (wheel tree), and *L. chinense* (Chinese tulip poplar). *E. grandis* also belongs to the same evolutionary clade as *P. trichocarpa* in core eudicots and may possess similar xylem characteristics. *L. chinense* resides in magnoliids as an early diverging lineage, which allows the investigation of xylem development under a larger evolutionary scale. In contrast to the vast majority of flowering plants, *T. aralioides* possesses tracheids instead of vessel elements and libriform fibers, which is a rare trait reversal during angiosperm evolution (Fig. 2A) [56, 57]. *L*. *chinense* protoplasts were sequenced using the 10x Genomics platform as for *P*. *trichocarpa*. Samples from *E*. *grandis* and field-collected *T*. *aralioides* were replete with cellular debris, which prompted us to adopt fluorescence-activated cell sorting coupled with the plate-based MARS-seq2.0 protocol to sequence transcriptomes of single sorted protoplasts with minimal debris contamination. We obtained the transcriptomic profiles from 5494 (*E. grandis*), 1993 (*T. aralioides*) and 2977 (*L. chinense*) individual SDX cells, respectively, which were grouped into 10 cell clusters in each species. (Fig. 2B–D, Additional file 1: Fig. S3). The DEGs of each cell cluster in the three species were then identified (Additional file 5–7).

Cross-species pairwise correlation analysis of transcriptomic profiles was performed to compare the major cell clusters of *E. grandis*, *T. aralioides*, and *L. chinense* to those of *P. trichocarpa* (Fig. 2E–G). Overall, the correlations between *P. trichocarpa* and *E. grandis* or *T. aralioides* are higher than those between *P. trichocarpa* and *L. chinense* (Fig. 2E–G), reflecting the closer phylogenetic relationships among the three eudicots than between eudicots and the more basal magnoliid angiosperm. Considering that the SDX cell types in *L. chinense* are anatomically the same as in *P*. *trichocarpa* [58] (Fig. 2A), two scenarios might explain their low transcriptomic correlation. First, most expressed genes are not related to the developmental paths and show low correlations. The SDX cell differentiation might be regulated by only a few key regulatory genes. Although most of gene expressions showed low correlations, the organizer cells can still develop into the same cell types under the control of these key regulators. Second, the cell types are actually of different developmental origins but share similar morphology due to convergent evolution.

We were able to identify representative cell clusters in *E. grandis* corresponding to different cell clusters in *P. trichocarpa*. Egr5, Egr7, and Egr8 correspond to the fusiform early precursors (Ptr4), libriform fibers (Ptr7), and ray parenchyma (Ptr8) cell clusters in *P. trichocarpa*, respectively, with the mutually highest correlation (mutual best-hit) in all-against-all comparison (Fig. 2E). Egr4 is the best-hit of vessel elements (Ptr1), and Ptr1 is the second best-hit of Egr4 (Fig. 2E). Both Egr6 and Egr1 have the highest correlation with fusiform organizer cells (Ptr6). Fusiform and ray organizer cells share high transcriptomic similarity in *P. trichocarpa* (Additional file 1: Fig. S19), indicating that Egr6 and Egr1 could be the fusiform and ray organizer cells.

As an evolutionarily reversed trait, the terminally differentiated fusiform cells in *T. aralioides* are tracheids, which are a primitive form with the combined functions of vessel elements and libriform fibers. The *T. aralioides* cell clusters Tar1 and Tar3 show the highest correlations with *P*. *trichocarpa* vessel elements (Ptr1) (Fig. 2F) and thus likely represent tracheids. Tar2 and ray parenchyma cells (Ptr8) are mutual best-hits, suggesting Tar2 represents ray parenchyma cells. For each of the three eudicot species, we identified marker genes from the DEGs exclusively expressed in different cell clusters (Additional file 1: Fig. S24, S25, Additional file 8–10). Notably, all cell clusters in *T. aralioides* show very low correlation to libriform fibers (Ptr7) (Fig. 2F), which implies that the fusiform lineage in *T. aralioides* has only one path toward tracheid/vessel characteristics. In other words, *T. aralioides* tracheids share a similar developmental path with vessel elements. Tracheids have both water transportation and mechanical support functions, which are the specialized functions of vessel elements and libriform fibers, respectively [36]. Here our results indicate the transcriptomic similarity between *T*. *aralioides* tracheids and *P*. *trichocarpa* vessel elements, which share the water transportation function as their common feature.

### Common and distinct developmental trajectories

**Fig. 3.**
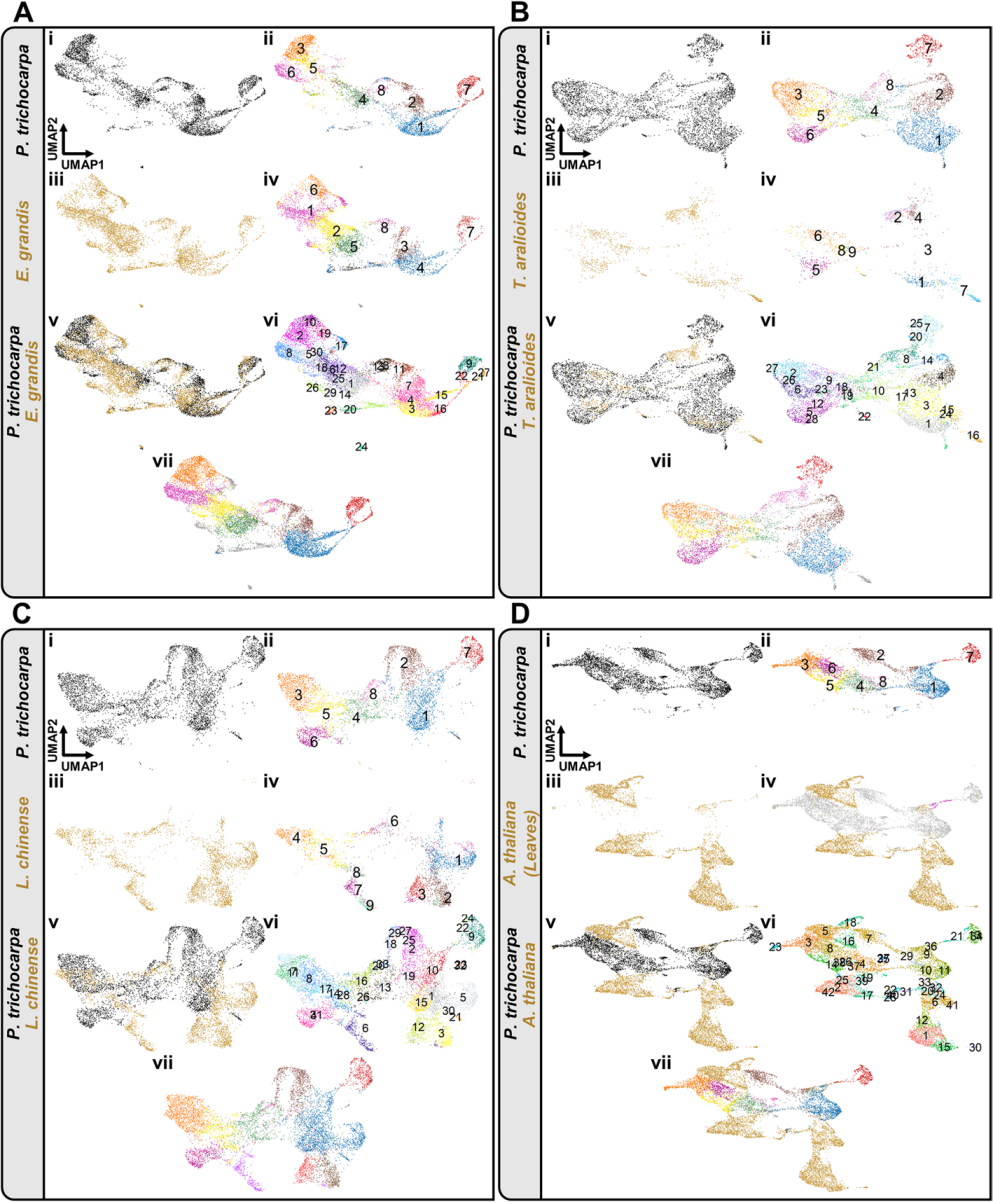
Two-species clustering and visualization of scRNA-seq data between *P. trichocarpa* and *E. grandis*, *T*. *aralioides*, *L. chinense*, or *Arabidopsis thaliana*. **A–C** Two-species clustering of SDX single cells in *P. trichocarpa* and *E. grandis* (**A**), *T. aralioides* (**B**), or *L. chinense* (**C**). Single-species unsupervised K-means clustering (i–iv). Two-species graph-based cell clustering using orthologous genes (v–vii). In (i), (iii), and (v), black dots are SDX cells from *P*. *trichocarpa* and gold dots are cells from *E*. *grandis*, *T*. *aralioides*, and *L. chinense*. In (ii) and (iv), the colors of cell clusters for each species are based on their single-species cell clustering results. The cell clusters in two-species clustering (vi). In (vii), the colors of two-species clustering are derived from that of single-species clustering (see “Methods”). **D** Two-species clustering of SDX cells in *P. trichocarpa* and leaf cells in *A. thaliana*. Single-species unsupervised *K*-means clustering (i–iii). Two-species graph-based cell clustering using orthologous genes (iv–vii). In (i) and (v), black dots are SDX cells from *P*. *trichocarpa*. In (iii), (iv), (v), and (vii), gold dots are cells from *A. thaliana*. In (iv), gray dots represent the SDX cells from *P. trichocarpa*, and the xylem cells identified in previous *Arabidopsis* studies are in magenta. In (ii) and (vii), the colors of cell clusters are based on the single-species cell clustering results. The cell clusters in two-species clustering (vi)

**Fig. 4.**
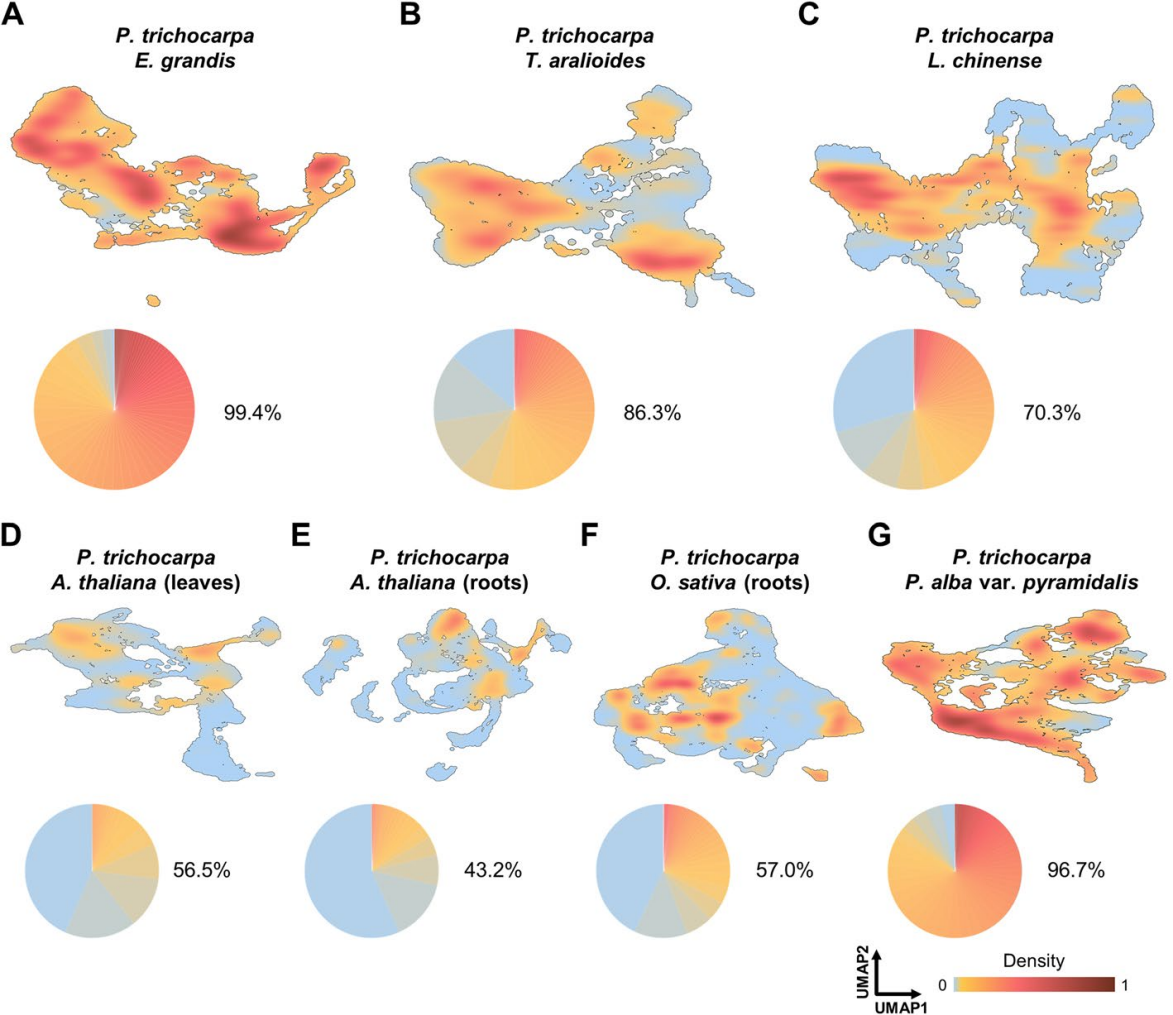
Distribution density of cells from two species. **A–G** Upper panel, cross-species distribution density of cells from *P. trichocarpa* and *E. grandis* (**A**), *T. aralioides* (**B**), *L. chinense* (**C**), *A. thaliana* leaves (**D**), *A. thaliana* roots (**E**), *O. sativa* (**F**), or *P. alba* var. *pyramidalis* (**G**) was calculated and two-dimensional-projected onto UMAP plots (Additional file 1: Fig. S26). Densities from 0 to 1 are divided into 500 bins with different color shading, with proportions of different densities shown in a pie chart in each panel. Lower panel, for each pair of species, the distribution overlap (indicated next to the pie chart) is the sum of proportions excluding the lowest bin, where cells from the two species barely co-localize

The cell lineages in the four woody species were further compared by integrating their scRNA-seq data using the Seurat canonical correlation analysis pipeline [59] for two-species graph-based cell clustering based on orthologous genes (Fig. 3). In addition, we devised a method to quantify distribution overlap between cells from different species (Fig. 4; Additional file 1: Fig. S26). *P. trichocarpa* and *E. grandis* two-species clustering (Fig. 3A(v), 3A(vi)) shows highly overlapped (Fig. 4A; overall distribution overlap >99%) fusiform and ray cell lineages (Fig. 3A(vii)). Egr6 was grouped with the *P. trichocarpa* ray organizers (Ptr3) in the two-species clustering (Fig. 3A(v)–(vii)), which reflects Egr6 as the best-hit of Ptr3 (Fig. 2E). We thus can also observe two cell lineages in the SDX of *E. grandis*, which are highly similar to those of *P. trichocarpa* differentiated from two types of organizer cells (Fig. 3A).

Two-species clustering of *P. trichocarpa* and *T. aralioides* also supports the conclusion from pairwise correlation analysis (Fig. 3B). Almost no cells from *T. aralioides* are grouped with libriform fibers (Ptr7) of *P. trichocarpa*, and Tar1 and Tar3 overlap with *P. trichocarpa* vessel elements (Ptr1) in UMAP. The fusiform lineage in *T. aralioides* has fusiform organizer (Tar5), fusiform early precursor (Tar9), fusiform intermediate precursor cells (low cell number), and tracheid cells (Tar1/Tar3) (Fig. 3B(v)–(vii)). The ray lineage started from the ray organizer (Tar6) to ray precursor (Tar8) then to ray parenchyma cells (Tar2) (Fig. 3B(v)–(vii)). Based on the similar developmental paths among the three woody eudicots, we could identify distinct modules of orthologous groups of genes with temporal expression patterns along the pseudotime of each cell lineage in two-species analyses (Additional file 1: Fig. S27, S28, Additional file 11, 12).

In the two-species clustering of *P. trichocarpa* and *L. chinense*, we found that their ray lineages are highly similar, but the fusiform lineage in *L. chinense* exhibits a distinct path from that in *P. trichocarpa* (Fig. 3C). We identified cell clusters corresponding to fusiform early (Ptr4/Lch8), intermediate (Ptr2/Lch2), and late precursors/vessel elements (Ptr1/Lch1), which barely overlap between *P. trichocarpa* and *L. chinense* (Figs. 3C(v)–(vii) and 4C). Despite their low transcriptomic correlation (Fig. 2G), fusiform organizer cells (Lch7) and libriform fibers (Lch3) of *L. chinense* could also be identified based on the expression of its homologs of the *P. trichocarpa* marker genes (Additional file 1: Fig. S29).

Overlapping cell populations between *P*. *trichocarpa* and each of the other three plants provides important insights into similarities and variation in developmental trajectories. To test whether such overlap could be caused by over-correction for dimensionality reduction in two-species analysis, we conducted the same procedure for *P. trichocarpa* SDX cells with each of the previously reported scRNA-seq dataset from *Arabidopsis* leaves (Fig. 3D), *Arabidopsis* roots (Additional file 1: Fig. S30A) and rice (*Oryza sativa*) roots (Additional file 1: Fig. S30B), where a sizable number of xylem or vascular cells were present (Additional file 1: Fig. S2). Most of the SDX cells from *P. trichocarpa* did not co-localize with the root and leaf cells from *Arabidopsis* and rice (Fig. 3D, Additional file 1: Fig. S30A, S30B). The distribution overlap between cells of *P. trichocarpa* and the other three woody species is much higher than between *P. trichocarpa* and *Arabidopsis* or rice (Fig. 4A–F). All of the xylem marker genes identified in previous scRNA-seq studies could be detected in our SDX scRNA-seq dataset (Additional file 1: Fig. S31). The distribution of the root and leaf xylem cells (magenta, Fig. 3D(iv), Additional file 1: Fig. S30A(iv), S30B(iv)) only barely overlaps with that of the *P*. *trichocarpa* SDX vessel elements (Ptr1), libriform fibers (Ptr7), and the transition area of these two clusters. In addition, almost no *Arabidopsis* xylem cells co-localize with the cell-type determined libriform fiber cluster (Fig. 3D(iv), Additional file 1: Fig. S30A(iv)), which is consistent to the absence of libriform fibers of their source tissue as the primary roots in 5-day-old *Arabidopsis* [60–64]. The *Arabidopsis* xylem cells also do not overlap with *P*. *trichocarpa* ray parenchyma cells (Ptr8) (Fig. 3D(iv), Additional file 1: Fig. S30A(iv)), consistent to previous finding that ray parenchyma cells cannot be observed in *Arabidopsis* under normal growing conditions [31–33]. The relatively limited xylem cells from previous studies of *Arabidopsis*, which has incomplete xylem development and cell types, only provide a partial understanding of xylem transcriptomic variation. Overall, our results demonstrate more conserved and overlapping developmental paths of SDX across divergent woody angiosperms, whereas the developmental trajectories of SDX in the rosid *P*. *trichocarpa* are clearly distinct from xylem in different organs in another rosid *Arabidopsis*.

**Fig. 5.**
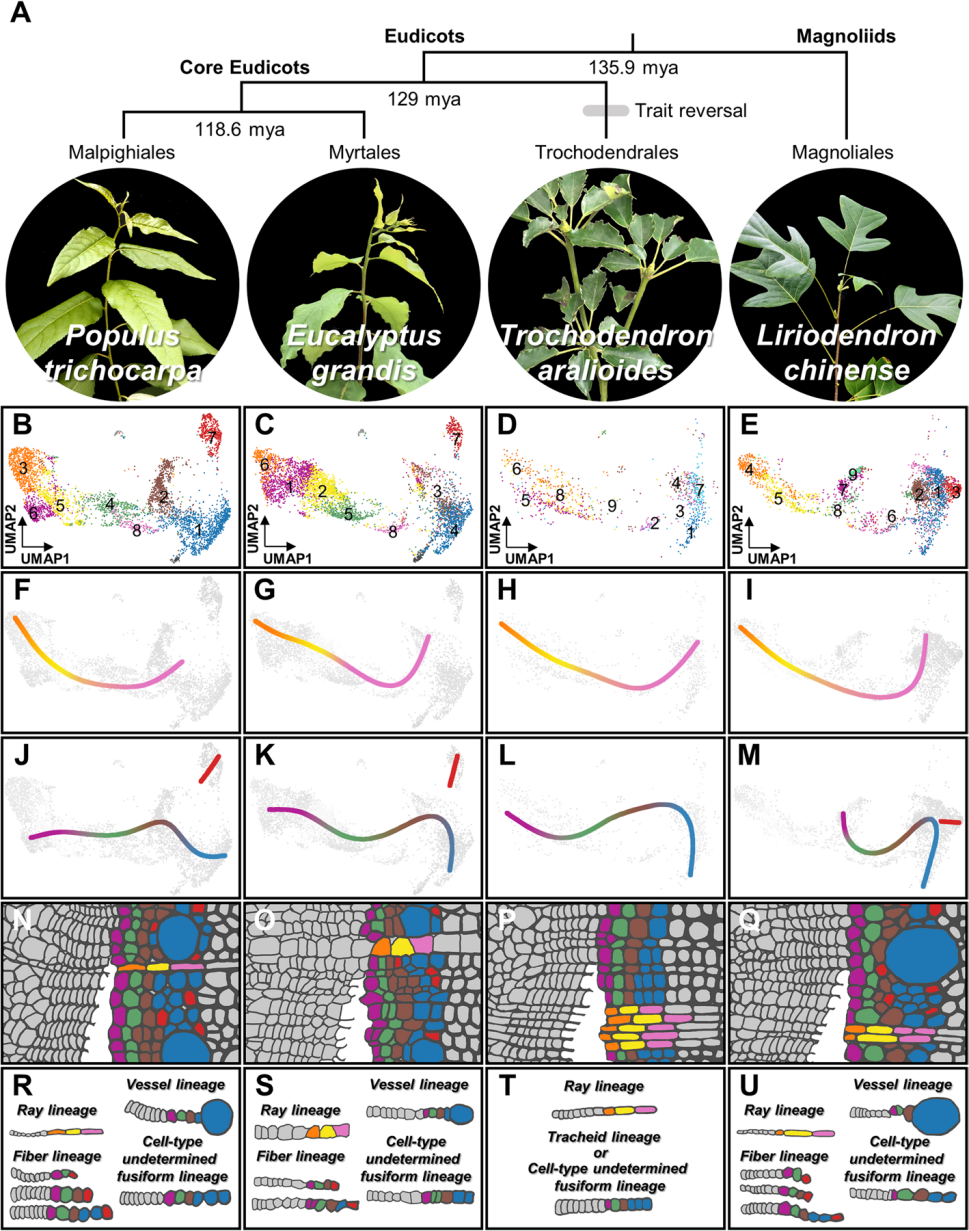
Cell lineages in SDX development in four woody angiosperms. **A** Phylogenetic relationships and estimated divergence times [65] of *P. trichocarpa*, *E. grandis* (both core eudicots), *T. aralioides* (basal eudicots), and *L. chinense* (magnoliids). Trait reversal: gain of tracheids and loss of vessel elements and libriform fibers. **B**–**E** Combined four-species analyses and two-dimensional visualization of SDX scRNA-seq data. Individual cells are colored as in Figs. 1 and 2. **F**–**M** The ray (**F**–**I**) and fusiform (**J**–**M**) lineages in the four species. **N–U** Schematics of different cell lineages across the four species

The conclusions from single- (Figs. 1 and 2) and two-species analyses (Figs. 3 and 4) are further supported by four-species combined analyses (Fig. 5). All four woody angiosperms have nearly identical ray cell lineages in their SDX (Fig. 5F–I). The three eudicots share similar fusiform cell lineages (Fig. 5J–L), except that *T. aralioides* does not have libriform fibers (Fig. 5L). The magnoliid *L. chinense* has a distinct fusiform cell lineage with fusiform organizer cells and libriform fibers different from those of eudicots (Fig. 5M).

### Expression of known genes in xylem cell lineages and their potential functions

We further examined the expression patterns of the known genes related to xylem development (Additional file 13), including (1) *VND6* and *SND1* in the VNS family, master regulators of xylem/vasculature development [15]; (2) *SND2*, the common direct target of *VND6* and *SND1* [66]; (3) cellulose, hemicellulose, and lignin biosynthesis genes for SCW deposition; (4) expansin for cell expansion; and (5) photosynthetic genes. In the three eudicots, *P. trichocarpa*, *P. alba* var. *pyramidalis*, and *E. grandis*, we found high expression of *VND6*, *SND1*, and *SND2* in fusiform lineages, SCW biosynthesis genes in both fusiform and ray lineages, and the photosynthetic genes mainly in ray lineages (Additional file 13). In *L. chinense*, the expression level of the orthologs of *VND6*, *SND1*, *SND2*, expansin A6, and hemicellulose biosynthesis genes were much lower than that in three eudicots due to their low expression. These discrepancies in gene expression patterns between *L. chinense* and core eudicots underscore their distinct SDX cell lineages. Cellulose synthases, lignin biosynthesis genes, and photosynthetic genes exhibited similar expression patterns in each cell type among four angiosperms, which illustrates the conserved and critical roles of these genes during xylem development.

**Fig. 6.**
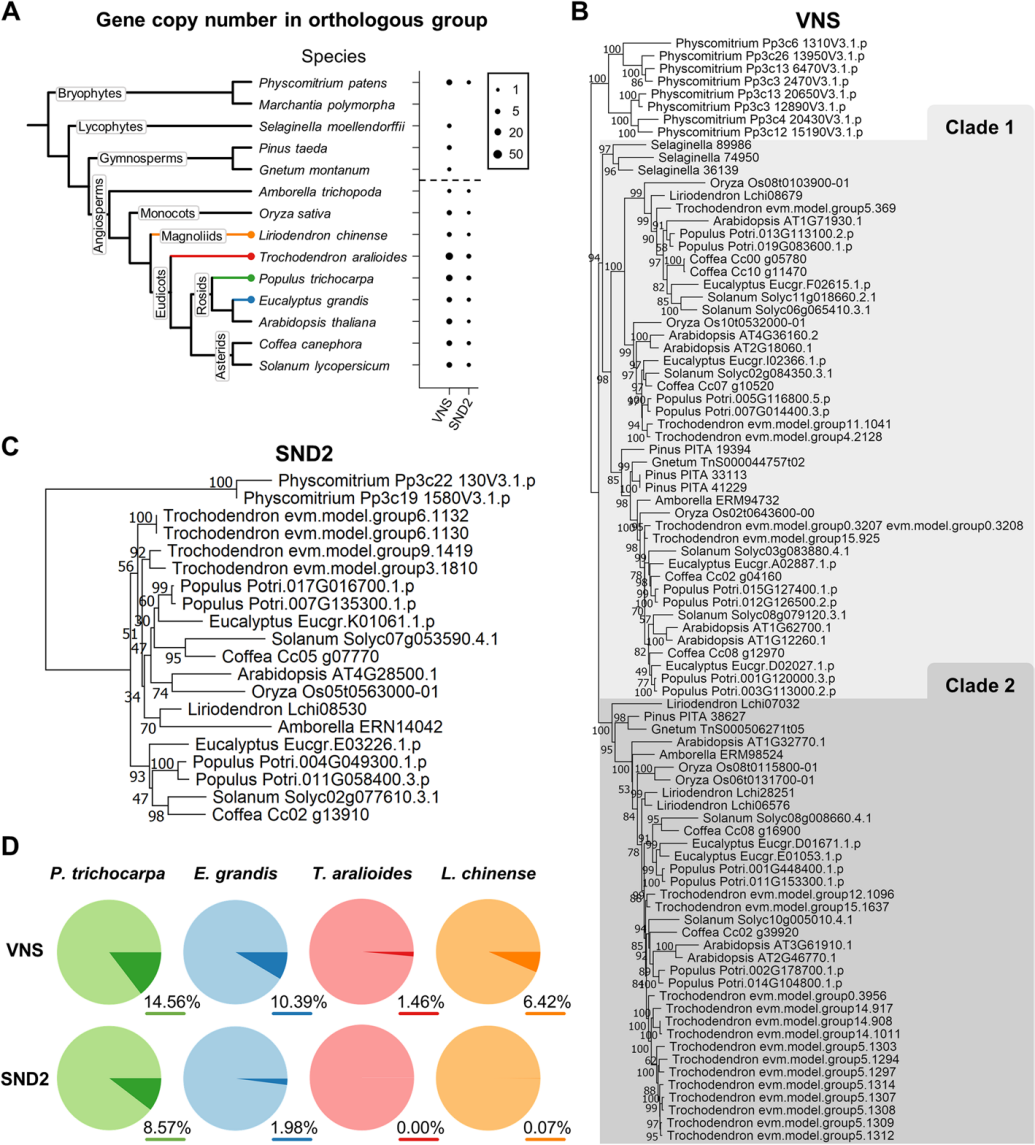
The distribution, phylogeny, and expression of *VNS* and *SND2* genes. **A** Gene copy numbers (represented by dot size) in orthologous groups of VNS family and *SND2* genes across 14 plant species. The dashed line marks the boundary between angiosperms and other plants. **B–C** Phylogenies of *VNS* (**B**) and *SND2* (**C**) genes from 14 species. **D** Pie charts show the transcript detection of *VNS* and *SND2* in each species. Darker color region in each pie chart represents the proportion of cells with detectable transcript abundance of *VNS* or *SND2* genes. The proportion of darker color region in each pie is shown at the bottom-right. Light color region represents the proportion of cells with zero UMI of *VNS* or *SND2* genes

The VNS family was reported to be the master regulator for xylem development in both vessel- and tracheid-bearing species through direct activation of *SND2* [15, 66, 67]. Both the *VNS* family and *SND2* were identified across all angiosperms, including *L. chinense*, with similar gene numbers (Fig. 6A), suggesting their potential conserved roles in regulating angiosperm xylem cell differentiation. Similar to vessel-bearing angiosperms, *T. aralioides* also possesses homologs of VNS and SND2 (Fig. 6A). In contrast, gymnosperms, which also have tracheids instead of vessels, only have VNS but not SND2 homologs (Fig. 6A, Additional file 1: Fig. S32). In addition, the SCW biosynthesis genes, expansin, and photosynthetic genes identified in scUPlcmUP genes (Additional file 1: Fig. S9) are conserved with similar gene copy numbers across angiosperms (Additional file 1: Fig. S32).

To reveal the evolutionary patterns of these regulators and genes of xylem cell differentiation, we compared their phylogenies with the scRNA-seq expression patterns (Fig. 6B–D, Additional file 14). The VNS family of vascular plants is divided into two clades, and both of which contain genes from the four woody angiosperms with expression detected (Fig. 6B, D). Our results suggest that VNS family exerts conserved functions throughout angiosperms, which was consistent to a previous study on VNS in land plants [15]. *SND2*, a direct target of VNS, was reported to be the key regulator of libriform fiber development [17, 68]. The absence of *SND2* genes in many gymnosperms and seedless vascular plants is thus consistent with their lack of libriform fibers (Fig. 6A). Intriguingly, *T. aralioides*, which also lacks libriform fibers, does possess *SND2* genes (Fig. 6A, C), but their expression was not detected in SDX cells (Fig. 6D). Namely, the function of SND2 could be turned off by the absence of this gene or its expression.

## Discussion

Every direction of plant development, including upward/downward apical growth and inward/outward lateral growth, starts from stem cell proliferation followed by cell identity determination and then cell differentiation [2]. In the past few decades, the cell proliferation, identity determination, and differentiation in SAM for upward and RAM for downward growth have been extensively studied [3–5], providing plentiful marker genes for recent scRNA-seq analyses to map the detailed cell lineages in apical growth. For lateral growth, vascular cambium is embedded inside the stems and cannot be directly visualized, so the physiological and morphological studies for xylem and phloem development appear to be much challenging. Recent studies, mostly in *Arabidopsis*, started to reveal bifacial stem cell proliferation in vascular cambium [6–8, 14], but the corresponding cell lineages still remain unclear. Furthermore, *Arabidopsis* does not have ray parenchyma cells [31–33], and its fiber cells remain immature without undergoing programmed cell death [34, 35], so insights from *Arabidopsis* would only show a partial perspective on xylem development. The SDX in woody plants generally has a full set of axial (libriform fibers and tracheids/vessel elements) and radial cells (ray parenchyma cells), and all types of cells develop into their mature stage through complete programmed cell death, providing a complete picture of xylem development trajectories. However, the intensive cell wall deposition and lignification with highly enriched phenolic compounds and other secondary metabolites constitute a major obstacle for many experiments. Take LCM for example, the penetration of laser cutting through SDX with thickened cell wall would be much more difficult than that for other tissue types with thinner cell wall. In addition, most woody plants are recalcitrant to genetic transformation, so functional validations of genes are often intractable. In short, woody species are suitable but more challenging for xylem research, whereas herbaceous plants are less ideal for unveiling complete xylem development.

The exploration of xylem cell development has been limited by the lack of comprehensive marker genes for cell type annotation. LCM was successfully used to isolate libriform fibers and vessel elements in *P. trichocarpa* for transcriptomic profiling [69, 70], but the collection of ray parenchyma cells has remained challenging. Ray parenchyma cells in *P. trichocarpa* are uniseriate (single-layered) and surrounded by libriform fibers and vessel elements. To avoid the contamination from the other two cell types, we carefully collected a very small area of ray parenchyma cells in each tangential section. Since extremely low amounts of area were collectable for ray parenchyma cells in each section, tremendous labor and time was needed for the section preparation and cell collection. The time required for collecting enough ray parenchyma cells was sixfold more than that required for libriform fibers or vessel elements. The even more challenging task was to collect all three cell types from the same stem internodes with very limited tissue for the in parallel comparison of their transcriptomic profiles. Unlike in situ hybridization targeted on a few genes, transcriptomic sequencing of in situ LCM-harvested samples provides comprehensive information for robust annotation of different xylem cell types and their representative gene sets for future studies.

A comprehensive understanding of plant cell development and evolution through comparative analyses of multi-species scRNA-seq data has been hindered by technical difficulty in robust protoplast isolation in divergent species spanning a large evolutionary scale. scRNA-seq requires high protoplast yield while minimizing cell debris. The lignified secondary cell wall in woody tissue, such as SDX, usually cannot be thoroughly removed during enzyme digestion, which causes a dilemma that higher protoplast yield comes with more abundant cell debris. Our efficient SDX protoplast isolation method for *P. trichocarpa* [38, 39] was successfully applied to *L. chinense* to harvest SDX protoplasts for 10x scRNA-seq. In contrast, SDX protoplast isolation of *E. grandis* and *T. aralioides* was contaminated by abundant cell debris that can interfere with single-cell isolation in 10x Genomics Chromium. We thus used fluorescence-activated cell sorting coupled with the plate-based MARS-seq2.0 method [71] to increase the efficiency of single-cell isolation for scRNA-seq. With the establishment of SDX protoplast isolation methods for *L. chinense*, *E. grandis*, and *T. aralioides* coupled with droplet- or plate-based library preparation, we were able to perform for the first time an evo-devo scRNA-seq analysis of cell lineages in four divergent plant species. Based on the clustering of homologous genes, our two-species and multi-species analyses could reveal the similarity and differences between the cell types and trajectories across these angiosperms.

A recent xylem scRNA-seq study on a single species, *Populus* sp., concluded a different xylem developmental path [72]. In their proposed cell lineages, vessel elements (fusiform cells) differentiated from the same precursor cells as ray parenchyma cells (non-fusiform cells) rather than libriform fibers (the other fusiform-cell type). This model contradicts with previous anatomical evidence that both fusiform-cell types differentiate from the same precursors that are distinct from those of non-fusiform cells. Our results combining scRNA-seq and lcmRNA-seq (in situ cell-type transcriptomic analysis) strongly support previous anatomical observations and provide a consistent view in other woody angiosperms.

Another recent study on SDX of *Populus alba* var. *pyramidalis* [44] concluded that cambium and xylem mother cells differentiate into fusiform and ray lineages. The separation of the ray and fusiform lineages is consistent with our results. However, their marker genes used for cambium annotation, *WOX4a*, *WOX4b*, *PXY*, *PIN1b*, *PIN1c*, are expressed in both cambium and ray parenchyma cells (Additional file 1: Fig. S11 in Chen et al., 2021) [44]. Besides, *HD-ZIP III* genes were used to identify xylem mother cells and organizers, and these cells are in the early stage of xylem development [8, 51, 73]. A combined analysis of *P. trichocarpa*, *P. alba* var. *pyramidalis*, *E. grandis*, *T. aralioides*, and *L. chinense* shows that these *HD-ZIP III* genes are not always expressed in these early stage cells (Additional file 1: Fig. S33A). The reliance on the genes as universal markers resulted in mis-annotation by swapping ray parenchyma cells and early stage cells (Additional file 1: Fig. S33B). This misplacement of these two cell types led to a reverse cell lineage, in which ray parenchyma cells differentiate into xylem mother cells/organizers. Such incorrect interpretation underlines the risk of cell-type annotation and in situ hybridization validation based on only a few genes, as well as the necessity of using whole transcriptomic profiles from lcmRNA-seq for accurate cell-type annotation in our study (Additional file 1: Fig. S33C).

In this study, we used *HD-ZIP III* as the marker gene for cell type annotation of organizer cells, but its gene expression pattern is not conserved between *P. trichocarpa* and other species in this study. The results raised an issue: Is *HD-ZIP III* a robust marker gene for the identification of organizer cells? If the answer is yes, then organizer cells would not exist in all species due to the nearly absence of *HD-ZIP III* expression in some species (Additional file 1: Fig. S33A). If the answer is no, then it brings up the next issue: Whether all species in this study possess organizer cells? If organizer cells are a common cell type, then different species would use various mechanisms, without *HD-ZIP III*, for the production of organizer cells. If organizer cells do not exist in certain species, the initials would require other cell types or other mechanisms for the maintenance. Based on the current knowledge, SAM, RAM, and vascular cambium in *Arabidopsis* all require organizer cells for the maintenance of stem cells/initials, the existence of organizer cells in all of the species in this study appears to be highly possible. *HD-ZIP III*, instead, seems not necessary for organizer cells in every species.

In *Arabidopsis*, organizer cells were found in the vessel element differentiation, but whether the differentiation of libriform fibers and ray parenchyma cells involve organizer cells remained unknown. HD-ZIP III played a critical role in organizer cells [8] in *Arabidopsis* and was used as a marker gene for organizer cell annotation in *P. trichocarpa* in this study. Besides the regulation of vessel element differentiation, previous studies in *Arabidopsis* and poplar have shown that HD-ZIP III can also accelerate the differentiation of libriform fibers [73, 74], suggesting the existence of organizer cells in the cell types beyond vessel elements.

Two contradicting conclusions were reported on the cell types of tracheary elements in *T. aralioides* [75, 76]. A previous study reported that *T. aralioides* possesses primitive vessel elements as the second type of tracheary elements in addition to tracheids [75]. While these two cell types have very similar cell length and width, the primitive vessel elements possess perforation plates without pit membranes. However, another study maintained that *T. aralioides* is vessel-less [76] and suggested that the pit membranes of some tracheids are destroyed in planta, causing them to appear anatomically similar to primitive vessel elements [76]. In our multi-species results, the fusiform lineage in *T. aralioides* is highly similar to that of vessel elements in other eudicots, and it does not diverge into two terminal cell types. Based on these, two possible scenarios can explain the observation of cells that look like primitive vessel elements. First, if there are two types of tracheary elements, tracheids and primitive vessel elements probably share comparable developmental paths that cannot be easily distinguished. Second, if there is only one type of tracheary elements, the tracheids might enter terminal differentiation in different stages, causing their slight anatomical variations.

It is still debated whether the cell fate of fusiform cells is determined at the very beginning in the initial cells or later during the differentiation of xylary derivatives [77]. Based on the ubiquitous expression of *VNS* family genes in all early fusiform cells [78], it has been proposed that all fusiform cells originally differentiate toward vessel elements but become committed toward libriform fibers upon an external or positional cue [77]. As a response to mechanical stress, for example, tension wood contains drastically increased libriform fibers [36, 79]. In this study, the fusiform lineage in *T. aralioides* is similar to that in *P. trichocarpa* and *E. grandis* (Fig. 5J–L). The fusiform lineage in *T. aralioides* solely developed into tracheid, and both tracheids and vessel elements share highly similar transcriptomes. The results indicated that the fusiform lineages of *P. trichocarpa* and *E. grandis* undergo the same differentiation path as that of *T. aralioides* into vessel elements or tracheids, which supports the hypothesis from the previous studies. Interestingly, the libriform fibers (Ptr7) are the most disjoint cell cluster (Fig. 1B, Additional file 1: Fig. S18), implying the differentiation into libriform fibers took place rapidly, leaving few intermediate cells to be captured for scRNA-seq. Such disconnection in cell states has also been demonstrated for malaria parasite cell types, where an abrupt transcriptomic change is caused by fast turn-on/off of transcriptional modules [80]. Our results suggest that fusiform cells differentiate into vessel elements by default, but can rapidly switch to libriform fibers.

## Conclusions

Using lcmRNA-seq and scRNA-seq of multiple species, our results provide crucial insights into the formation and developmental dynamics of SDX. In particular, we discovered that (1) the radial (ray parenchyma cells) and axial (libriform fibers and tracheids/vessel elements) systems in SDX develop through two cell lineages from ray and fusiform organizer cells to precursor cells then to terminally differentiated cells (Fig. 5N–U); (2) The transcriptomic profile of tracheids is most similar to that of vessel elements instead of libriform fibers, with the lack of *SND2* genes or their expression in SDX in vessel-less seed plants; (3) The variation in the single-cell transcriptomic profiles reflects both evolutionary relationships across species and developmental divergence across organs (Fig. 4); (4) In contrast to highly conserved ray lineages, fusiform lineages are more variable across core eudicots, basal eudicots, and magnoliids (Fig. 5). Xylem constitutes the largest biomass on Earth [37] as the essential tissue for two major functions in vascular plants. The mechanical support allows vascular plants to dominate current terrestrial ecosystems by towering over their bryophyte relatives. Water transportation delivers critical elements to whole plant bodies. By integrating high-resolution single-cell analyses across multiple species with evolutionary divergence of over a hundred million years, we provide a comprehensive view of how cells form and vary in SDX. This approach is applicable to other plant species, and further studies of the entire plant kingdom can potentially shed light on the origin of vascular development.

## Methods

### Plant materials and sampling

*P. trichocarpa* plants were grown in a walk-in growth chamber under 16-h light/8-h dark conditions at 24–26°C. Tree stems were collected from 8-month-old plants. For protoplast isolation, stems below the 12th internode were used. For LCM, the 8th internode of the tree stems was harvested. *E. grandis* plants were grown in a walk-in greenhouse for 8 months with sunlight and temperature around 20–30°C. Tree stems below the 18th internode were collected for protoplast isolation. The branches from wild-grown *T. aralioides* in Hutian Elementary School (Yangmingshan National Park, Taiwan) were collected for protoplast isolation. Around 1-year-old plants of *L. chinense* were purchased from Nanping Senke Seedlings Co., Ltd. (Fujian, China), and the stems below around the 10th internode were used for protoplast isolation.

### SDX protoplast isolation

The SDX protoplast isolation was carried out mainly based on our previously established protocol [38, 39, 81]. Tree stems were cut into 8-cm segments. Debarked stem segments were dipped into freshly prepared enzyme solution (20 mM MES, pH 5.7, 0.25 M mannitol, 20 mM KCl, 1.5% wt/vol cellulase R10, 0.4% wt/vol Macerozyme R10, 10 mM CaCl_2_, and 0.1% BSA) for ~3 h at room temperature. Enzyme-digested stem segments were transferred to MMG solution (4 mM MES, pH 5.7, 0.25 M mannitol and 15 mM MgCl_2_, room temperature) or modified MMG solution (4 mM MES, pH 5.7, 0.25 M mannitol and 20 mM KCl, room temperature, for subsequent 10x Chromium scRNA-seq) to release SDX protoplasts. The protoplasts were filtered with 70-μm nylon mesh, pelleted at 900*g* centrifugation for 3 min at room temperature, and resuspended in MMG solution. The isolated SDX protoplasts were observed using a fluorescence microscope (Additional file 1: Fig. S34).

### 10x Chromium scRNA-seq

Single-cell sequencing libraries of two biological replicates (one replicate in one batch, total two batches) for *P. trichocarpa* and one biological replicate for *L. chinense* SDX protoplasts were generated using Chromium Single Cell 3’ Library and Gel Bead Kit v2 or v3 (10x Genomics) according to user manual. The *P. trichocarpa* SDX protoplasts in the second batch (10x_Batch2 in Additional file 1: Fig. S35A) were stained with a working concentration of 0.008 μg/μL fluorescein diacetate (Sigma-Aldrich, USA) and sorted by a BD FACSAria™ III cell sorter (BD, USA) before generating the single-cell sequencing libraries. To minimize the formation of doublets or even multiplets, the number of loaded cells was following the instruction in the manual of 10x Genomics for an estimated multiplet rate less than 8%. The scRNA-seq libraries of *P. trichocarpa* were sequenced using Illumina HiSeq 4000 or NovaSeq 6000 (Genomics Co., Ltd), and the libraries of *L. chinense* were sequenced by NovaSeq (Novogene Co., Ltd). The sequenced data of *P. trichocarpa* and *L. chinense* were individually processed using Cell Ranger (10x Genomics; v5.0.1) with the commands “cellranger mkref” and “cellranger count”. The Cell Ranger-compatible references were built with the current genome sequences and annotations (see below) for the following read alignment and quantification. The single-cell transcript abundance matrices were generated and named as “filtered_feature_bc_matrix.h5” by Cell Ranger. The transcript abundance of each gene was calculated based on the number of unique molecular identifier (UMI)-tagged transcripts as “UMI counts”. A total of 19,007 and 2977 transcriptomes were obtained for individual SDX cells in *P. trichocarpa* and *L. chinense*, respectively, each with UMI counts more than 500 (Additional file 1: Fig. S1). In *P. trichocarpa*, total 28,691 genes were detected among the transcriptomic dataset, which represents 82.7% of the whole annotated genes (34,699 genes) (Additional file 1: Fig. S1). In *L. chinense*, 17,926 genes were detected, representing 50.8% of the whole annotated genes (35,269 genes). One batch of *P. trichocarpa* scRNA-seq results (10x_Batch1 in Additional file 1: Fig. S35A, S35B) was used for the following analyses.

### Cell sorting and plate-based scRNA-seq

The massively parallel single-cell RNA-seq2.0 (MARS-seq2.0) [71] protocol was adapted for sequencing *E. grandis* and *T. aralioides* SDX protoplasts using five and four biological replicates, respectively (Additional file 1: Fig. S1). Four biological replicates with total 6 rounds of scRNA-seq for *T. aralioides* were performed in two batches in different seasons. For the first batch (MARS-seq_Batch1 in Additional file 1: Fig. S35D), two trees were used in summer (two rounds of scRNA-seq). For the second batch (MARS-seq_Batch2 in Additional file 1: Fig. S35D), four trees, including the two trees from first batch, were used in winter (four rounds). Three hundred eighty four-well plates were prepared using Beckman Biomek NXR Liquid Handling Workstation (Beckman, USA) and PerkinElmer Janus MDT (PerkinElmer, USA), with 2 μL lysis buffer (0.1% Triton X-100, 0.2U/μL RNAsin Plus and 1 nM reverse transcription (RT) primers with barcodes) in each well. *E. grandis* and *T. aralioides* SDX protoplasts were stained with a working concentration of 0.008 μg/μL fluorescein diacetate (Sigma-Aldrich, USA). The samples were analyzed and sorted by a Beckman Coulter MoFlo XDP (Beckman, USA) or BD FACSMelody™ cell sorter (BD, USA) with 488-nm laser excitation and a 100-μm nozzle. Cells were identified based on forward scatter (FSC), side scatter (SSC), chlorophyll fluorescence (663–737 nm), and fluorescein fluorescence (475–650 nm). A single cell was isolated under the “single cell mode” into individual wells of the prepared 384-well plates. After sorting, the plates were immediately frozen on dry ice and stored at −80°C until processing. The plates were thawed at room temperature, heated to 95°C for 3 min, and cooled on an iced metal block. The RT reagent mixture, including 1:80,000-diluted ERCC spike-in RNA, was dispensed into each well using Mantis v3.2 (Formulatrix, Massachusetts, USA). After RT and Exonuclease I treatment, all wells in each plate were pooled together through 3-min centrifugation at 1800 rpm (Heraeus Multifuge X3R with a TX-750 swinging bucket rotor, Thermo Fisher, USA). Further processing of the plate pools followed the MARS-seq2.0 protocol, including second-strand synthesis, in vitro transcription, DNase treatment, RNA fragmentation, ligation and plate barcoding, and second RT. The quality of the libraries was assessed by quantitative PCR using QuantStudio 12K Flex Real-Time PCR System (Applied Biosystems, USA). The cDNA pools were amplified by a final PCR with the introduction of Illumina I7 indices. Libraries were paired-end sequenced (R1:130, R2:20) on Illumina NextSeq 500 (Genomics Core, Institute of Molecular Biology, Academia Sinica, Taiwan). Sequencing data was analyzed using the MARS-seq2.0 mapping and demultiplexing pipeline [71]. In *E. grandis*, total 23,086 genes were detected among the transcriptomic dataset, which represents 63.5% of the whole annotated genes (36,349 genes) (Additional file 1: Fig. S1). The first batch of *E. grandis* scRNA-seq results (MARS-seq_Batch1 in Additional file 1: Fig. S35C) was used for the following analyses. In *T. aralioides*, 26,226 genes were detected, representing 74.2% of the whole annotated genes (35,328 genes). The first batch of *T. aralioides* scRNA-seq results (MARS-seq_Batch1 in Additional file 1: Fig. S35D) was used for the following analyses. For performance comparison between 10x Chromium and plate-based scRNA-seq (MARS-seq), five biological replicates of *P. trichocarpa* SDX protoplasts were performed using plate-based scRNA-seq by the same pipeline as for *E. grandis* and *T. aralioides* (MARS-seq_Batch1 and MARS-seq_Batch2 in Additional file 1: Fig. S35B). In *P. trichocarpa*, 27,581 genes were detected, which represents 79.5% of the whole annotated genes (34,699 genes) (Additional file 1: Fig. S1).

### The reproducibility of the scRNA-seq results

To ensure the reproducibility of the scRNA-seq results and the robustness of the methods, scRNA-seq for additional biological replicates of *P. trichocarpa* was performed. The transcriptomes of 25,166 cells from seven biological replicates were obtained using 10x Genomics Chromium technology and MARS-seq (Additional file 1: Fig. S1). An integrated analysis of all seven replicates shows highly overlapped distribution of cells from different replicates (Additional file 1: Fig. S35A, S35B). The results also show that single-cell transcriptomes based on 10x scRNA-seq and MARS-seq are comparable (Additional file 1: Fig. S35B; distribution overlap: 99.5%), which indicates the two methods for cell isolation and library preparation have little effects on the transcriptomic profiles observed. For *E. grandis*, the cells from two batches with a total of five biological replicates are also highly overlapped (Additional file 1: Fig. S35C; 99.7%). Since *T. aralioides* samples can only be collected from the field instead of a temperature-controlled greenhouse, we sequenced another batch of samples from four biological replicates in different seasons with additional trees (Additional file 1: Fig. S1). The distribution of cells from the two batches is almost completely overlapped (Additional file 1: Fig. S35D; 100.0%). By examining the batch effects of biological replicates and library preparation methods, we demonstrate the high reproducibility of our scRNA-seq results. In addition, we compared our *P*. *trichocarpa* data with the SDX scRNA-seq data of another poplar species, *Populus alba* var. *pyramidalis*, from a recent study [44]. Two-species clustering of *P. alba* var. *pyramidalis* and *P. trichocarpa* shows that their cell populations largely overlapped (Fig. 4G, Additional file 1: Fig. S36; 96.7%). Five-species clustering also demonstrates that the fusiform and ray lineages of *P. alba* var. *pyramidalis* are conserved with those in *P. trichocarpa* and *E. grandis*, which is consistent with their close evolutionary relationships (Additional file 1: Fig. S37).

### Dimensionality reduction and cell clustering

For *P. trichocarpa* and *L. chinense*, the dimensionality reduction and cell clustering were performed by Cell Ranger (10x Genomics; v5.0.1) using the single-cell transcript abundance matrices with the command “cellranger reanalysis”. For *E. grandis* and *T. aralioides*, the results from MARS-seq2.0 were converted into the single-cell transcript abundance matrices as the input files for Cell Ranger to exert the subsequent command “cellranger reanalysis” for dimensionality reduction and cell clustering. The principal component analysis was performed, and the top 10 principal components were used to conduct UMAP and unsupervised K-means clustering with the parameters “umap_n_neighbors = 50” and “umap_min_dist = 0.5”. Differential expression analysis was performed following the pipeline in Cell Ranger of 10x Genomics platform using two methods. Negative binomial exact test (two-sided), the hypothesis test based on the sSeq method [82], was applied to DEG identification in default. When the counts of transcript abundance in both comparing groups were greater than 900, the hypothesis test would be switched to asymptotic beta test (two-sided) adapted from the implementation in edgeR [83]. For each cell cluster, the DEGs were identified by treating the cells inside and the cells outside this cluster as two samples. Take the cell cluster Ptr1 as an example, each gene was tested whether its mean expression in Ptr1 differs from that in all other cell clusters. The *P* values from the multiple comparisons were corrected by Benjamini-Hochberg method (BH method) [84] to control the false discovery rate (FDR) as 0.05. DEGs of each cell cluster were obtained using the output table “differential_expression.csv” in the folder “kmeans_X_clusters” generated by Cell Ranger. The “X” in “kmeans_X_clusters” was corresponding to the cluster number in each species. Only the clusters with more than or equal to 5 cells would be considered as cell clusters. The cutoff of DEGs was set as adjusted *P* value < 0.05 and the absolute value of log_2_-fold change (log_2_FC) ≥ 1. The UMI counts of each cell were divided by the total UMI counts and multiplied by a scale factor (1000) to obtain the normalized UMI counts. The log_2_-transformation was applied to the normalized UMI counts to plot the gene transcript abundance.

### Laser capture microdissection and RNA sequencing (lcmRNA-seq)

The 8th internodes from six *P. trichocarpa* stems were cut into 5- or 10-mm segments, frozen by liquid nitrogen immediately, and stored at −80°C. The 5-mm segments were separated into four quarters at −20°C in the chamber of a cryostat. The segments were stuck on cryostat chucks using Tissue-Tek O.C.T. Compound (Sakura Finetek, USA) and sliced into 16-μm thick sections using cryostat (Leica CM1900) at −20°C. The cryo-sections were placed onto the PET membrane of metal-frame slides (Leica, USA) and dehydrated using 99.5% ethanol. Around 1,600,000 μm^2^ area from approximate 20 cell layers of cryo-sections (transverse or tangential) of two *P. trichocarpa* plants was collected using laser microdissection systems (Leica LMD7000), and evenly pooled as one biological replicate. A total of three biological replicates were collected for each cell type. The dissected cells were collected within 30 min in 500-μL eppendorf caps loaded with 50 μL RLT buffer (QIAGEN, USA) and 1% beta-mercaptoethanol (Sigma-Aldrich, USA), frozen by liquid nitrogen, and stored at −80°C. Total RNA from dissected cells was extracted using RNeasy Plant Mini Kit (QIAGEN, USA) according to the manufacturer instruction. The mRNA of each cell type was amplified using Arcturus® RiboAmp® HS PLUS RNA Amplification Kit (Applied Biosystems, USA) following the user guide. The amplified mRNA of each sample was used to construct sequencing libraries using NEBNext® Ultra™ II Directional RNA Library Prep Kit (New England Biolabs, USA) according to the instruction manual. The RNA-seq libraries were sequenced on Illumina NextSeq 500 platform (Genomics Core, Institute of Molecular Biology, Academia Sinica, Taiwan) to obtain the reads with paired-end 75-bp length. We avoided the sample contamination throughout the whole process of laser capture microdissection based on the following pipelines: (i) Issue of cell visualization. During cell collection, we visualized and selected our targeting area, which contained the cell type of interest. For a sample section, one side can be visualized, and the other side contacted the slide, which cannot be visualized. When laser cut though the selected area on a section, we collected both visualizable and unvisualizable regions. To avoid the collection of unwanted cells, the thickness of sections should contain only one layer of cells or should be even less than the length or width of one layer of cells, which can thus avoid the unwanted cells hiding in the unvisualizable regions. We set the thickness of cross and tangential sections as 16 μm. For cross sections of libriform fiber collection, the thickness is much less than the cell length of libriform fiber (500–750 μm) [85]. For tangential sections of vessel element collection, the thickness is also much less than the cell diameter of vessel elements (34–48 μm) [85]. (ii) Issue of residual fragments on slides. Laser cutting is actually a process of burning out the cells by laser on the selected paths. While burning out the cells, laser cutting also generates tiny fragments. These fragments either would fall down into the collecting caps along with the targeting cell area or remained on the slides. The fragments remained on the slides may fall down and get collected in the next rounds of cutting. Thus, each round of cutting would result in the collection of targeting cell area and the fragments generated on such round or previous rounds. As a result, each slide was only used for the collection on one cell type to avoid cross contamination of cell types caused by the collection of unwanted residual fragments.

### Paraffin sectioning

Around 1-mm segments were cut from 3rd to 6th stem internodes of *P. trichocarpa* and were fixed using 100% acetone for 40 min under vacuum at 4°C. These segments were further fixed by 100% acetone at 4°C overnight then at 37°C for 1.5 h. The segments were placed into serial n-butanol:acetone solution as 30:70, 50:50, 70:30, 90:10, and 100:0 v/v at 58°C followed by serial paraffin:n-butanol solution as 50:50, 60:40, 80:20, and 100:0 v/v at 58°C. The segments were used to generate 16-μm transverse sections by a microtome (Microm, HM355E). The paraffin embedded in the sections were removed with xylene for 15 min and 99.5% ethanol for 30 min.

### Microscopic imaging

The transverse and tangential sections were observed under Olympus BX53 Upright Microscope in bright field. The sections for LCM were examined using the embedded camera function in laser microdissection systems (Leica LMD7000). The lignin fluorescence was observed by the excitation wavelength and the emission wavelength as 360–370 nm and 420–460 nm, respectively.

### lcmRNA-seq data analysis

The sequencing raw reads were preprocessed with fastp [86] (v0.20.1) using five arguments “--detect_adapter_for_pe, --correction, --cut_front, --cut_tail and --disable_trim_poly_g”. The adapter sequences and low-quality sequences (mean quality value < 20) at the both 5′ and 3′ ends of each read were removed. The reads with any of the following features were discarded: (1) shorter than 15 bps, (2) containing more than five N bases and (3) including more than 40% of bases with low quality (quality value < 15). The filtered reads were mapped to the *P. trichocarpa* reference genome with HISAT2 [87, 88] (v2.2.1) using four arguments “--max-intronlen 17787, --secondary, --fr and --rna-strandness”. The parameter of the argument “--max-intronlen 17787” as maximum intron length was obtained from the current *P. trichocarpa* genome structural annotation (see below). The transcriptome quantification was performed using StringTie [89] (v2.1.3b) with the current annotation to obtain the raw read counts and transcripts per million (TPM) of each gene. The differential expression analysis was implemented with the negative-binomial-based test (two-sided) in DESeq2 [90] (v1.28.1) using the raw read counts. The FDR was controlled with BH method [84], and the significance level was set as 0.05. DEGs of each cell type were identified under the cutoff set as the absolute value of log_2_FC ≥ 1 compared with the other cell types (Additional file 15). For example, the DEGs of libriform fibers were the genes with the absolute value of log_2_FC ≥ 1 both in libriform fibers/vessel elements and libriform fibers/ray parenchyma cells.

### Correlation analysis between scRNA-seq and lcmRNA-seq data

Pearson’s correlation coefficients were calculated between the transcriptome of each cell from scRNA-seq and each cell type from lcmRNA-seq data. UMI counts and average TPM were used to represent the transcript abundance of the transcriptome data of each cell in scRNA-seq and each cell type in lcmRNA-seq, respectively.

### Transcript abundance quantification of SDX

The RNA-seq raw reads from three biological replicates of SDX in *P. trichocarpa* were downloaded from NCBI GEO database using the accession number GSE81077 [69]. The RNA-seq data was processed using the same pipeline as lcmRNA-seq data analysis mentioned above to obtain TPM of each gene in SDX.

### Transcript abundance of the gene sets in different GO terms

Gene Ontology (GO) annotations of *A. thaliana* genes were obtained from TAIR (The Arabidopsis Information Resource) database [91]. The corresponding GO terms of each *P. trichocarpa* gene were assigned by the best hit to *A. thaliana* genes provided in Phytozome database [92]. In each GO term analysis, the set of *P. trichocarpa* genes related to the target GO terms with all descendants (child terms) would be used. For each gene, the local transcript abundance at each cell point was estimated with the weighted average of normalized UMI counts calculated using Gaussian kernel smoother. The Euclidean distance used in the kernel smoother was based on the top 10 principal components from Cell Ranger (10x Genomics). The relative transcript abundances were then defined as the ratio between the local transcript abundance of each cell and the mean local transcript abundance. The mean relative transcript abundance in the gene set of interest was calculated for each cell. The results were visualized as a heatmap on the UMAP from Cell Ranger (10x Genomics). Total 27, 69, and 43 *P. trichocarpa* genes were used for the analyses of “meristem initiation,” “cellulose biosynthetic process,” and “hemicellulose metabolic process,” respectively.

### Transcriptomic analysis for SDX from different internodes

DEGs of each internode section were identified using the same analysis pipeline described in lcmRNA-seq data analysis. The principal component analysis was performed using the normalized counts output from DESeq2 [90] (v1.36.0), and top two principal components were used for visualization.

### Quantification of marker gene transcript abundance in fusiform and ray lineages

Based on the clustering and UMAP results from Cell Ranger (10x Genomics), the pseudotime analysis for *P. trichocarpa* cells was performed by Slingshot [93] (v2.4.0) using our defined cell lineages as fusiform and ray lineages. For each gene, the expected transcript abundances along each lineage were estimated by Nadaraya-Watson kernel regression estimation [94, 95] with Gaussian kernel using normalized UMI counts as the response variable and pseudotime as the explanatory variable.

### Identification of homologous genes

We used sequence similarities to identify homologous protein-coding genes encoded in genomes of the four woody angiosperms and 10 other species with a balanced coverage of the plant diversity, including *Physcomitrium patens*, *Marchantia polymorpha*, *Selaginella moellendorffii*, *Pinus taeda*, *Gnetum montanum*, *Amborella trichopoda*, *Oryza sativa*, *Arabidopsis thaliana*, *Coffea canephora*, and *Solanum lycopersicum*. An all-against-all BLASTP search [96, 97] (v2.6.0) was conducted for the amino acid sequences with an e-value cutoff of 10^−15^. OrthoMCL [98] (v1.3) was used to cluster the protein-coding genes based on their BLASTP similarities into orthologous groups using the Markov Cluster Algorithm [99] with an inflation of 1.5 (Additional file 10). For the cross-species analyses, the ortholog transcript abundance was used from a set of orthologous groups. The ortholog transcript abundance was calculated as the sum of gene transcript abundance within each orthologous group. The ortholog transcript abundance was normalized with the total ortholog transcript abundance in each cell, and then multiplied by a scale factor (1000). The log_2_-transformation was applied to the normalized ortholog transcript abundance to plot the ortholog transcript abundance. Total 25,733 orthologous groups were used in the cross-species analyses. Orthologous groups #1284, #1746, and #8129 represent VNS family orthologs. Orthologous group #4898 is used as the collection of *SND2* gene orthologs.

### Pairwise correlation analysis among cell clusters

For *P. trichocarpa*, Pearson’s correlation coefficients were calculated between each pair of major cell clusters (Ptr1–Ptr8) using the average normalized UMI counts within each cell cluster. For two-species comparisons, the average normalized ortholog transcript abundance was calculated within each major cell cluster (except Egr9, Egr10, Tar10, and Lch10) for generating Pearson’s correlation coefficients between each pair of cross-species cell clusters.

### MetaCell analysis of P. trichocarpa SDX cells

As an alternative to unsupervised K-means clustering and UMAP visualization, the MetaCell package [55] (v0.3.41) was used to group the *P*. *trichocarpa* SDX cells and project them onto a two-dimensional plot. A total of 4278 variable genes were selected as marker genes (T_tot = 20, T_top3 = 1, T_szcor = −0.01, T_vm = 0.2, and T_niche = 0.05) for constructing *k*-nearest neighbor graphs with *K* = 40, followed by coclustering with bootstrapping based on 1000 iterations of resampling 75% of the cells and an approximated target minimum metacell size of 50. Unbalanced edges were filtered with *K* = 40 and alpha = 3. The individual cells were then plotted in a two-dimensional projection (Additional file 1: Fig. S18).

### Two-, four-, or five-species scRNA-seq data integration for woody plants

Seurat pipeline (v4.0.3) was used for the integration for the scRNA-seq results from multiple species [59, 100]. Only the cells with more than 200 detected orthologs and the orthologs detected in more than 3 cells were included in the analysis. The ortholog transcript abundance in the filtered data was normalized according to Seurat guideline. Top 2000 highly variable orthologs were identified in each species and used as the input of canonical correlation analysis (CCA). The correlated gene modules were identified and presented as the cross-species cells embedding in a shared low-dimensional space. L2-normalization was then performed on the cell embedding to reduce the global different between datasets. The anchors, defined as the mutual nearest neighbors between datasets, were identified with *K*-nearest neighbors (*K* = 5) using 2000 highly variable orthologs among two, four, or five species. After anchor scoring and anchor weighting, the anchors were then used for data integration. UMAP and cell clustering were performed based on shared nearest neighbor modularity optimization. The determination for the cell clusters (color) in two-species clustering (*P. trichocarpa*, *E. grandis*, *T. aralioides* and *L. chinense*, Fig. 3A(vii), 3B(vii), 3C(vii)) was mainly based on the majority cell clusters (color) from single-species clustering results. Take a cell cluster in two-species clustering for *P. trichocarpa* and *E. grandis* as an example, if this cell cluster contains 100 cells, 50 from *P. trichocarpa* (48 in red cluster and 2 in brown cluster) and 50 from *E. grandis* (30 in red cluster and 20 in brown cluster), then all 100 cells would be defined as red cell cluster. For the two-species clustering, the cell clusters of *P. alba* var. *pyramidalis* were assigned using the co-located cell clusters in *P. trichocarpa* (Additional file 1: Fig. S36(vii)).

### Data comparisons of scRNA-seq data from same species in different batches or different platforms

Four batches of *P. trichocarpa*, two batches of *E. grandis*, and two batches of *T. aralioides* scRNA-seq data were integrated and visualized respectively following the Seurat pipeline (v4.0.3) and UMAP mentioned above (Additional file 1: Fig. S35).

### Reanalysis of Arabidopsis, rice and P. alba var. pyramidalis scRNA-seq data

For *Arabidopsis* leaves [29], the scRNA-seq data (10x Genomics v2) of one biological replicate (genotype ATML1p::YFP-RCI2A) was downloaded from NCBI GEO database using the accession number GSE167135 as single-cell transcript abundance matrices in one file (filtered_gene_bc_matrices-_h5.h5). This h5 file was analyzed with the command “cellranger reanalysis” in Cell Ranger (10x Genomics; v5.0.1) to obtain the UMAP and unsupervised *K*-means clustering results (Additional file 1: Fig. S38A). According to Lopez-Anido et al. [29], the xylem cells in *Arabidopsis* leaves can be identified using the gene expression of *AT3G21270* (Additional file 1: Fig. S38A). For *Arabidopsis* roots [20], the scRNA-seq data with three biological replicates of wild type was downloaded from NCBI GEO database under the accession number GSE123013 as single-cell transcript abundance matrices composed of three separated files (barcodes.tsv, genes.tsv and matrix.mtx). The three biological replicates of the wild-type cell transcriptomes were integrated and processed (Additional file 1: Fig. S38B) by Seurat as mentioned above. According to Ryu et al. [20], the xylem cells in *Arabidopsis* roots can be identified using the gene expression of *BHLH30* (*AT1G68810*) (Additional file 1: Fig. S38B). For rice roots [28], the scRNA-seq raw reads from two biological replicates of wild type were downloaded from NCBI SRA database under the accession number PRJNA706435. Transcriptome quantification, followed by the UMAP and unsupervised *K*-means clustering, was performed by Cell Ranger (10x Genomics; v5.0.1) using the command “cellranger mkref” and “cellranger count” (Additional file 1: Fig. S38C). According to Zhang et al. [28], the xylem cells in rice roots can be identified using the gene expression of *Os01g0750300* (Additional file 1: Fig. S38C). For *Populus alba* var. *pyramidalis* wood tissues [44], the scRNA-seq raw reads were downloaded from National Genomics Data Center under BioProject accession number PRJCA005543. Transcriptome quantification was performed by Cell Ranger (10x Genomics; v5.0.1) using the genome sequence and annotation of *P. trichocarpa* with the command “cellranger mkref” and “cellranger count”. Each reanalyzed scRNA-seq dataset of *Arabidopsis* roots and leaves, rice roots, and *P. alba* var. *pyramidalis* SDX were integrated with the SDX scRNA-seq results of *P. trichocarpa* by Seurat using the pipeline mentioned above.

### Cell density of overlapped cells

We developed a method to quantify distribution overlap between cells from two different species (Fig. 4) or samples (Additional file 1: Fig. S35) based on minimal spanning trees (MSTs) [101, 102]. MSTs were constructed from integrated UMAP plots (Additional file 1: Fig. S26A, S266E). Subgraphs were obtained by removing cross-species or cross-sample edges (Additional file 1: Fig. S26B, S26C, S26F, S26G). The center node (with the highest closeness centrality) was determined for each subgraph (Additional file 1: Fig. S26D, S26H). MST construction, graph determination, and closeness centrality calculation were performed using igraph [103] (v1.2.6) in R. For single-node subgraphs, the only node was directly defined as the center node (Additional file 1: Fig. S26D, S26H). The distribution overlap was quantified and visualized based on the density distribution of center nodes (Additional file 1: Fig. S26I) with the following procedures. Individual two-dimensional kernel density was calculated using MASS [104] (v7.3-54) from the distribution of center nodes (Additional file 1: Fig. S26J, S26K), weighted by the number of center nodes and the proportion of cells of each species or sample (Additional file 1: Fig. S26L), and concatenated into a single density map (Additional file 1: Fig. S26M). For multi-species analyses, the density maps were normalized by the total density values of *P. trichocarpa* cells (Fig. 4, Additional file 1: Fig. S26N). For the reproducibility analyses of *P. trichocarpa*, the density maps were normalized based on the first batch of single-cell transcriptomic data from 10x Genomics Chromium technology (Additional file 1: S26N; 10x_Batch1 in Additional file 1: Fig. S35A, S35B). For the reproducibility analyses of *E. grandis* and *T. aralioides*, the normalization of density maps was based on the first batch of single-cell transcriptomic data from MARS-seq (Additional file 1: S26N; MARS-seq_Batch1 in Additional file 1: Fig. S35C, S35D).

### Pseudotime trajectory analysis and lineage curve construction

The pseudotime analysis was performed by Slingshot [93] (v2.0.0) based on the integrated UMAP from Seurat. From our defined cell lineages as tracheid, vessel element, libriform fiber, and ray parenchyma lineages, the pseudotime of each cell in our defined cell lineages was obtained, and the cells were ordered based on their corresponding pseudotime. The window size was set as 21 cells and slid one cell at a time to calculate the moving average from the ortholog transcript abundance (UMI) in each cell. For example, one orthologous group in the first 21 cells with minimal pseudotime in a certain cell lineage would generate an average of transcript abundance, and such orthologous group in the 2nd to 22nd cells would generate another average. The union of all of these averages is named as moving average. The differentially expressed orthologous groups (DEOs) were identified using the command “FindAllMarkers” in Seurat with MAST [105] (v1.18.0), which was based on the hurdle model tailored to scRNA-seq analysis. Two-sided tests were performed, and the output *P* values were adjusted based on Bonferroni correction. Under the criteria of adjusted *P* value < 0.05, DEOs were obtained as the orthologous groups differentially expressed in any one of the integrated cell clusters from two species. Each DEO in different cell lineages was plotted using their corresponding moving averages. The DEOs from each cell cluster belong to different modules, yielding total eight modules from 1 to 8. More than 95% of the cells in each cell cluster from every species were used to construct the smooth lineage curves basically passing the centers of each cell cluster according to the pre-defined lineages using Slingshot [93].

### Phylogenetic trees

The phylogenetic trees were constructed based on the protein sequences for each orthologous group (Fig. 6B, C, Additional file 14). For *P. trichocarpa*, *E. grandis*, *Physcomitrium patens*, and *Selaginella moellendorffii*, the sequences of primary transcripts “protein_primaryTranscriptOnly.fa” were used, which were downloaded from Phytozome [92]. For *Arabidopsis thaliana*, *Oryza sativa*, and *Marchantia polymorpha*, the transcripts with the longest coding sequence for each gene were extracted and used. For the other species, their annotations do not include the information about transcript variants. Two steps of analyses were used to build phylogenetic trees. First, multiple sequence alignment based on the input protein sequences was conducted by MAFFT [106, 107] (v7.490). The results were then used to construct phylogenetic trees by IQ-TREE [108–110] (v1.6.12) with ModelFinder for model selection [109] and UFBoot2 for ultrafast bootstrap approximation [110]. The output trees were visualized and converted into images by MEGA 11 [111] (v11.0.11).

### Data source of genome sequences, genome annotations, and peptide sequences

We downloaded the genome sequences, genome annotations or peptide sequences of the 14 species from the following databases: *Populus trichocarpa* (v4.1; Phytozome v13, https://phytozome-next.jgi.doe.gov [92]), *Eucalyptus grandis* (v2.0; Phytozome v13 [92]), *Trochodendron aralioides* (released on October 10^th^ 2019; GigaDB, http://gigadb.org [112]), *Liriodendron chinense* (generated on January 15^th^ 2019; Hardwood Genomics Project, https://hardwoodgenomics.org [113]), *Amborella trichopoda* (Ensembl Genomes, release 49, http://ftp.ensemblgenomes.org/pub/plants/release-49 [114]), *Arabidopsis thaliana* (Araport11; The Arabidopsis Information Resource, https://www.arabidopsis.org [91]), *Coffea canephora* (Coffee Genome Hub, https://coffee-genome.org [115]), *Gnetum montanum* (Dryad Digital Repository, https://datadryad.org [116]), *Marchantia polymorpha* (MpTak1v5.1; MarpolBase, https://marchantia.info [117]), *Oryza sativa* (Ensembl Genomes, release 49 [114]), *Physcomitrium patens* (v3.3; Phytozome v13 [92]), *Pinus taeda* (v2.01; TreeGenes, https://treegenesdb.org [118]), *Selaginella moellendorffii* (v1.0; Phytozome v13 [92]), *Solanum lycopersicum* (ITAG4.0; Sol Genomics Network, https://solgenomics.net [119]).

### Supplementary Information

**Additional file 1: **Figure S1–S38. **Fig. S1.** Summary of scRNA-seq assays of SDX in *P. trichocarpa*, *E. grandis*, *T. aralioides* and *L. chinense*. Statistics of scRNA-seq profiling by 10x Chromium (red) or MARS-seq2.0 (green) are shown for cells with at least 500 and 100 UMIs, respectively, including cell numbers, biological replicate numbers, total detected genes, percentage of detected genes, mean read counts per cell and median number of genes detected per cell. **Fig. S2.** A summary of the xylem cells identified from previous studies. The previous studies with sizable cell numbers from xylem-related cell types are used in this study with highlighted light red color as the background. Four sets of cells from Ryu et al. (907 cells from xylem containing stele) [20], Lopez-Anido et al. (1004 cells from vasculature) [29], Zhang et al. (xylem cell number was not available (NA)) [28] and Chen et al. (3170 cells from wood tissue) [44] are selected. Using the marker genes in the three studies with *Arabidopsis* or Rice, total 353, 30 and 2508 xylem cells are identified. **Fig. S3.** Cell numbers of SDX scRNA-seq cell clusters in *P. trichocarpa*, *E. grandis*, *T. aralioides* and *L. chinense*. (A–D) SDX single-cell transcriptomes of *P. trichocarpa* (A), *E. grandis* (B), *T. aralioides* (C) and *L. chinense* (D) were grouped using unsupervised K-means clustering into 10 cell clusters in each species. The number of cells is shown for each cell cluster. **Fig. S4.** Workflow of laser capture microdissection for three xylem cell types in *P. trichocarpa*. (A) Schematic of transverse and tangential sectioning of tree stems. A whole stem segment and a quarter stem segment are loaded on cryostat chucks for transverse and tangential sectioning, respectively. Sections are then placed on metal-frame slides with PET membrane for subsequent LCM cell type isolation. (B) Real sections before and after laser cutting of libriform fibers, vessel elements and ray parenchyma cells. The area within the green circles represents the cutting area. Scale bars, 100 μm. In (A) and (B), transverse and tangential sections are marked with dark-blue and light-blue backgrounds, respectively. **Fig. S5.** Libriform fiber collection in transverse and tangential views. (A–I) The red area represents the libriform fibers collected using LCM. Libriform fiber collection from transverse sections, showing before cutting (B), area selection (C) and after cutting (D). Scale bar, 500 μm. In (C) and (D), the area switched from red to white, leaving an empty space after the libriform fibers were cut by laser. Libriform fibers in transverse and tangential views (E–I). A transverse section with an area highlighted (red) for libriform fiber collection (E). A closeup of the highlighted area (F). Three-dimensional structure around the highlighted area (G). A tangential section with highlighted area (H). Corresponding panel of Fig. 1E (I). **Fig. S6.** The illustrations for vessel element and ray parenchyma cell collection from transverse and tangential perspectives. (A–C) The schematics of vessel elements from transverse and tangential perspectives. The blue area represents the vessel elements collected using LCM. In (A), the corresponding figure of Fig. 1I represents a tangential section with an area highlighted (blue) for vessel element collection. In (B), a three-dimensional structure of the highlighted area shows the location of the collected vessel elements in the stem structure schematic. In (C), the corresponding figure of Fig. 1H represents a transverse section with highlighted area. (D–F) The schematics of ray parenchyma cells from transverse and tangential perspectives. The pink area represents the ray parenchyma cells collected using LCM. In (D), the corresponding figure of Fig. 1M represents a tangential section with a highlighted area (pink). In (E), a three-dimensional structure of the highlighted area shows the location for the collected ray parenchyma cells in the stem structure schematic. In (F), the corresponding figure of Fig. 1L represents a transverse section of the highlighted area. (G and H) The three-dimensional arrangement of three xylem cell types from tangential (G) and radial (H) perspectives. F, libriform fibers. V, vessel elements. R, ray parenchyma cells. **Fig. S7.** Violin plots of the transcriptomic correlation between each cell type of lcmRNA-seq and each cell cluster of scRNA-seq results. (A–C) The correlations between scRNA-seq results with lcmRNA-seq results of libriform fibers (A), vessel elements (B) or ray parenchyma cells (C), respectively. **Fig. S8.** Bar charts of the scUPlcmUP gene numbers. (A–C) The scUPlcmUP gene numbers of libriform fibers (A), vessel elements (B) and ray parenchyma cells (C). Only the lcmUP genes with average transcripts per million (TPM) more than 4 were included. **Fig. S9.** Previous known genes in the scUPlcmUP genes. (A–N) Secondary cell wall biosynthesis genes expressed in libriform fibers. (O) The expansin gene expressed in vessel elements. (P–W) The photosynthesis-related genes expressed in ray parenchyma cells. **Fig. S10.** Relative transcript abundance of the secondary cell wall biosynthesis genes in the cell clusters. The differentially expressed genes are marked as red for up-regulations and green for down-regulations. **Fig. S11.** Known libriform fiber marker genes. The exclusive expression of *IRX15-L* (*PdDUF579-9*) and *ASPARTIC PROTEASE 66* (*PtAP66*) in libriform fibers. **Fig. S12.** Transcript abundance of the *P. trichocarpa* expansin genes. The *P. trichocarpa* homologs of *A. thaliana* expansin genes are listed with their corresponding transcript abundance and their transcript proportion in SDX among all expansin transcript abundance. **Fig. S13.** Violin plots of the transcript abundance of each orthologous group in the scRNA-seq results. (A–F) Violin plots are used to reveal the log_2_ normalized UMI counts of each orthologous group in each cell cluster. **Fig. S14.** Transcript abundance of the genes from cellulose-, hemicellulose-, meristem-related GO terms and known marker genes across cambial and SDX regions. (A–C) Relative transcript abundance of cellulose- (A), hemicellulose- (B), and meristem-related (C) genes. The GO terms for cellulose biosynthetic process, hemicellulose metabolic process, and meristem initiation are GO: 0030244, GO: 0010410, and GO: 0010014, respectively. (D–E) Time-course transcriptomic analyses across cambial and SDX regions. A schematic reproduction of gene expression results across the cambial and SDX regions from a previous study [52] conducted using a series of 20-μm tangential sections (D). The corresponding transcript abundance in the scRNA-seq results in this study (E). **Fig. S15.** Transcript abundance of up-regulated genes in scRNA-seq results of vessel elements and ray parenchyma cells reveals fusiform and ray cell lineages. (A and B) The gene expression profiles of the up-regulated genes in vessel elements and ray parenchyma cells on the UMAP show continuous cell lineages for fusiform (A) and ray cells (B), respectively. **Fig. S16.** Transcript abundance of up-regulated genes in each cell type across fusiform and ray lineages. (A) Transcript abundance of the up-regulated genes across ray lineage. (B) The normalized mean expression in ray lineage. (C) The normalized mean expression in fusiform lineage. (D) Transcript abundance of the up-regulated genes across fusiform lineage. (E) to (F) Bar charts represent the normalized mean expression of the up-regulated genes and the homologs of *PdMYB156* and *PdMYB221* in *P. trichocarpa* of fusiform lineage and ray lineage, respectively. Normalized mean expression was calculated as the mean UMI counts of each gene normalized with mean UMI counts of all genes. RM, normalized mean expression in ray lineage. FM, normalized mean expression in fusiform lineage. **Fig. S17.** Expression pattern of known marker genes. (A–D) The transcript abundance of *P. trichocarpa* homologous genes of *PdMYB156* (A), *PdMYB221* (B), *ESK1a* (C) and *ABR1* (D) in scRNA-seq results. **Fig. S18.** MetaCell plot of the cell clusters in *P. trichocarpa*. Two-dimensional projection of *P*. *trichocarpa* SDX single cells using MetaCell dimensionality reduction. Cells are labeled with colors corresponding to those in Fig. 1B. V, vessel element. FuIP, fusiform intermediate precursor. RO, ray organizer. FuEP, fusiform early precursor. RP, ray precursor. FuO, fusiform organizer. F, libriform fiber. R, ray parenchyma cell. **Fig. S19.** Pairwise correlation of the cell clusters in *P. trichocarpa*. Pairwise correlation between transcriptomic profiles of Ptr1–Ptr8 of *P. trichocarpa* SDX. V, vessel element. FuIP, fusiform intermediate precursor. RO, ray organizer. FuEP, fusiform early precursor. RP, ray precursor. FuO, fusiform organizer. F, libriform fiber. R, ray parenchyma cell. **Fig. S20.** Schematics of different developmental cell lineages in SDX in *P. trichocarpa*. (A) After stem debarking, the revealed SDX on the stem surface contains xylem organizer and differentiating xylem. (B) Schematic examples of four cell lineage types in SDX, including (i) ray parenchyma cell lineage, (ii) vessel element lineage, (iii) libriform fiber lineage and (iv) another incomplete or undergoing cell lineage as cell-type undetermined fusiform lineage. The first three lineages (i–iii) start with initials to organizers to precursors then to cell-type determined ray parenchyma cells, vessel elements or libriform fibers. **Fig. S21.** Dot plots exhibit preferential patterns of marker genes in each cell cluster of libriform fiber/vessel element or ray parenchyma cell lineages. (A and B) The dot size represents the proportion of cells in each cell cluster with the marker gene expression, and the dot brightness shows the relative mean gene expression of the marker genes in libriform fiber/vessel element (A) or ray parenchyma cell lineages (B), respectively. **Fig. S22.** Transcript abundance of marker genes of each cell cluster in *P. trichocarpa*. Many DEGs from each cell cluster are identified as marker genes if these genes show an exclusive expression in that cell cluster. **Fig. S23.** Transcriptomic analyses of SDX across different internodes in *P. trichocarpa*. (A) Schematics of different stages in vertical growth in *P. trichocarpa*, including primary growth, transition zone and secondary growth. SDX from different sections, sections 1 to 5, were used for RNA-seq. (B) Anatomical analyses of SDX in different developmental stages. The cross-sections were observed under fluorescence microscope, and the blue signals represent deposited lignin. Red arrows indicated bundle vasculature. The yellow arrow showed the transition between bundle and circular vasculature. The green arrow indicated circular vasculature. Scale bars are 100 μm. (C) Principal component analysis and differential expression analyses were conducted using the SDX RNA-seq datasets from different internodes. Total 23,441 genes were expressed in secondary xylem, and 12/6/11/11/18 genes were differentially expressed in sections 1 to 5, respectively. XE, secondary xylem expressed genes. S1 to S5, sections 1 to 5. **Fig. S24.** Representative marker orthologous groups exclusively expressed in the different cell clusters in both *P. trichocarpa* and *E. grandis*. (A) The cell cluster plots obtained through unsupervised K-means clustering and UMAP are based on the SDX scRNA-seq results of *P. trichocarpa* and *E. grandis*, respectively. (B) The cluster-exclusive distributions of each marker orthologous group are represented by Group #1287 for vessel element/late fusiform precursor (blue), Group #316 for ray organizer (orange), Group #1373 for fusiform early precursor (green), Group #132 for ray precursor (yellow), Group #1385 for fusiform organizer (purple), Group #9398 for libriform fiber (red) and Group #180 for ray parenchyma cell (pink). No marker orthologous groups are observed in fusiform intermediate precursor (brown). **Fig. S25.** Representative marker orthologous groups exclusively expressed in the different cell clusters both in *P. trichocarpa* and *T. aralioides*. (A) The cell cluster plots obtained through unsupervised K-means clustering and UMAP are based on the SDX scRNA-seq results of *P. trichocarpa* and *T. aralioides*, respectively. (B) The cluster-exclusive distributions of each marker orthologous group are represented by Group #1848 for vessel element/late fusiform precursor (blue), Group #9334 for fusiform early precursor (green), Group #4229 for fusiform organizer (purple) and Group #1916 for ray parenchyma cell (pink). No marker orthologous groups are observed in fusiform intermediate precursor (brown), ray organizer (orange) and ray precursor (yellow). **Fig. S26.** Analysis of distribution overlap of cells between two species or samples. (A–H) Two schematic examples are given: *P. trichocarpa* vs. *E. grandis* and *P. trichocarpa* vs. *A. thaliana*. (A and E) All the nodes (cells) are connected with the total length of lines as small as possible. (B and F) The lines between the nodes from different species are removed, generating subgraphs. (C and G) The numbers of subgraphs are counted. (D and H) One of the node with the highest centrality in each subgraph is extracted as the center node (circled in red). (I) After removing the other nodes, the density of subgraph centers is calculated with the procedure from (J) to (N). The schematic example of *P. trichocarpa* vs. *E. grandis* is used for explanation. (J) The plots of center nodes are separated by individual species. (K) Density of center nodes is calculated for each plot as individual density. (L) Individual densities are weighted by the proportion of cells and the subgraph numbers to obtain weight densities. (M) Weighted densities are added together into a concatenated density. (N) A concatenated density is normalized by the total density of black cells. **Fig. S27.** Pseudotime analysis to reveal the temporal expression pattern of each cell lineage using two-species clustering of *P. trichocarpa* and *E. grandis*. (A) Ray parenchyma, vessel element and libriform fiber lineages are from ray organizer or fusiform organizer. (B) Representative orthologous groups for each cell cluster can be categorized into eight modules (1–8). Along with the lineages two color bars represent as cells from two species (*P. trichocarpa* as black and *E. grandis* as gold) and their corresponding clusters. Maximum and mean UMI counts of each orthologous group are shown. **Fig. S28.** Pseudotime analysis to reveal the temporal expression pattern of each cell lineage using two-species clustering of *P. trichocarpa* and *T. aralioides*. (A) Ray parenchyma, vessel element/tracheid and libriform fiber lineages from ray organizer or fusiform organizer are shown. (B) Representative orthologous groups for each cell cluster can be categorized into eight modules (1–8). Along with the lineages two color bars represent as cells from two species (*P. trichocarpa* as black and *T. aralioides* as gold) and their corresponding clusters. Only *P. trichocarpa* exhibits cells in libriform fiber cluster (Ptr7) of the libriform fiber lineage. Maximum and mean UMI counts of each orthologous group are shown. **Fig. S29.** The *L. chinense* homologous genes of the fusiform marker genes and monolignol biosynthesis genes from *P. trichocarpa*. (A and B) The transcript abundance of the *L. chinense* homologous genes of the fusiform marker genes (A) and monolignol biosynthesis genes (B) from *P. trichocarpa* are shown on the UMAP plots of two-species analyses (Fig. 3C (iv)). **Fig. S30.** Two-species clustering and visualization of scRNA-seq data between *P. trichocarpa* and *A. thaliana* or *O. sativa*. (A and B) Two-species clustering of SDX cells in *P. trichocarpa* and root cells in *A. thaliana* (A) or *O. sativa* (B). Single-species unsupervised K-means clustering (i–iii). Two-species graph-based cell clustering using orthologous genes (iv–vii). In (i) and (v), black dots are SDX cells from *P*. *trichocarpa*. In (iii), (iv), (v), and (vii), gold dots are cells from *A. thaliana* or *O. sativa*. In (iv), gray dots represent the SDX cells from *P. trichocarpa*, and the xylem cells identified in previous *Arabidopsis* or rice studies are in magenta. In (ii) and (vii), the colors of cell clusters are based on the single-species cell clustering results. The cell clusters in two-species clustering (vi). **Fig. S31.** Transcript abundance of previously identified xylem marker genes. The transcript abundance of the *P. trichocarpa* homologs of *A. thaliana* and *O. sativa* genes are shown on the UMAP plots of scRNA-seq results. **Fig. S32.** Gene copy numbers in the orthologous groups of xylem development related genes across 14 plant species. The different sizes of dots represent gene copy numbers in each orthologous group of each species. The names of each orthologous group are represented by their group numbers (see Additional file 10) followed by their common names or the gene functions. The dashed line separates angiosperms from gymnosperms and seedless plants. **Fig. S33.** The cell-type annotation using *HD-ZIP III* as marker genes in different species. (A) Transcript abundance of *HD-ZIP III* is shown on the UMAP plots of five-species analyses (Additional file 1: Fig. S37A–E). (B–C) Cell-type annotations and developmental lineages derived from Chen et al. [44] (B) and from this study (C). **Fig. S34.** SDX protoplasts of *P. trichocarpa*, *E. grandis*, *T. aralioides* and *L. chinense*. The SDX protoplasts were observed using fluorescence microscope. Upper panel, the morphology of SDX protoplasts from each species in bright field. Lower panel, the SDX protoplasts with FDA staining. Scale bars are 50 μm. **Fig. S35.** Reproducibility of 10x scRNA-seq and MARS-seq in *P. trichocarpa*, *E. grandis* and *T. aralioides*. (A–D) The scRNA-seq data integration (left panel), cell density of the clustering results from different batches or platforms (middle panel) are two-dimensional projected on the UMAP plots for *P. trichocarpa* (A and B), *E. grandis* (C) or *T. aralioides* (D). Densities from 0 to 1 are divided into 500 bins with different color shading, with proportions of different densities shown in a pie chart (right panel). The distribution overlap (indicated above the pie chart) is the sum of proportions excluding the lowest bin, where cells from the two batches or platforms barely co-localize. Each batch of each species represents 1 or multiple biological replicates (Bio-rep) (see Methods for the details). **Fig. S36.** Two-species clustering and visualization of scRNA-seq data between *P. trichocarpa* and *P. alba* var. *pyramidalis*. Two-species clustering of SDX single cells in *P. trichocarpa* and *P. alba* var. *pyramidalis*. Single-species unsupervised K-means clustering (i–iv). Two-species graph-based cell clustering using orthologous genes (v–vii). In (i), (iii), and (v), black dots are SDX cells from *P*. *trichocarpa* and gold dots are cells from *P. alba* var. *pyramidalis*. In (ii), the colors of cell clusters in *P. trichocarpa* are based on the single-species cell clustering results. In (iv), the cell cluster colors are assigned using the co-located colors from *P. trichocarpa* cell clusters. The cell clusters in two-species clustering (vi). In (vii), the colors of two-species clustering are derived from that of single-species clustering. **Fig. S37.** Cell lineages in SDX development in five woody angiosperms. (A–E) Combined five-species analyses and two-dimensional visualization of SDX scRNA-seq data. Individual cells are colored as in Figs. 1 and 2 and Additional file 1: Fig. S36. (F–O) The ray (F–J) and fusiform (K–O) lineages in the five species. **Fig. S38.** Xylem cell identification from previous scRNA-seq results. (A–C) Different replicates of the scRNA-seq results from *A. thaliana* roots and *O. sativa* roots are first integrated for cell clustering using Seurat pipeline. Previous identified xylem cells using marker genes reveal that the xylem cells locate at Cluster 17, Cluster 10 and Cluster 11/20 in *A. thaliana* leaves (A), roots (B) and *O. sativa* roots (C), respectively.

**Additional file 2. **The secondary cell wall biosynthesis genes in the single-cell major clusters of SDX in *P. trichocarpa*.

**Additional file 3. **DEGs in the single-cell major clusters of SDX in *P. trichocarpa*.

**Additional file 4. **DEGs of SDX from different internodes in *P. trichocarpa*.

**Additional file 5. **DEGs in the single-cell major clusters of SDX in *E. grandis*.

**Additional file 6. **DEGs in the single-cell major clusters of SDX in *T. aralioides*.

**Additional file 7. **DEGs in the single-cell major clusters of SDX in *L. chinense*.

**Additional file 8. **Orthologous groups containing DEGs of each cell cluster both in *P. trichocarpa* and *E. grandis* scRNA-seq results.

**Additional file 9. **Orthologous groups containing DEGs of each cell cluster both in *P. trichocarpa* and *T. aralioides* scRNA-seq results.

**Additional file 10.** Orthologous groups identified using orthoMCL.

**Additional file 11. **Distinct modules of orthologous groups in pseudotime analysis of *P. trichocarpa* and *E. grandis*.

**Additional file 12. **Distinct modules of orthologous groups in pseudotime analysis of *P. trichocarpa* and *T. aralioides*.

**Additional file 13. **Expression profiles of the homologous genes of known xylem development related genes in different xylem cell trajectories of *P. trichocarpa*, *P. alba* var. *pyramidalis*, *E. grandis*, *T. aralioides* and *L. chinense*. Empty plots with no coordinates were used to represent the absence of the orthologs in certain species.

**Additional file 14.** Phylogenetic trees of homologous genes of known xylem development related genes from 14 species.

**Additional file 15. **DEGs of each cell type in lcmRNA-seq results of *P. trichocarpa*.

**Additional file 16.** Plate barcode and index information in plate-based scRNA-seq results.

**Additional file 17. **Review history.

## Data Availability

Sequence data from this article can be found in the NCBI GEO under accession number GSE180121 [120]. For *E. grandis*, *T. aralioides*, and *P. trichocarpa*, the plate barcode and Illumina i7 index were used to extract raw reads belonging to *E. grandis*, *T. aralioides*, and *P. trichocarpa* (Additional file 16). Third-party datasets used in this article include scRNA-seq data for *Arabidopsis* leaves [121], *Arabidopsis* roots [122], rice roots [123] and *Populus alba* var. *pyramidalis* wood tissues [124], and RNA-seq data for *P. trichocarpa* SDX [125]. All custom code is available on Zenodo [126] and under the MIT license in the GitHub repository [127].
