# Supplementary material for: Single-cell transcriptomics unveils xylem cell development and evolution
Source: Genome Biol. 2023 Jan 9;24:3. doi: 10.1186/s13059-022-02845-1 (PMC9830878; doi:10.1186/s13059-022-02845-1)
Supplement: Supplementary file 1 — Additional file 1: Figure S1–S38. Fig. S1. Summary of scRNA-seq assays of SDX in P. trichocarpa, E. grandis, T. aralioides and L. chinense. Statistics of scRNA-seq profiling by 10x Chromium (red) or MARS-seq2.0 (green) are shown for cells with at least 500 and 100 UMIs, respectively, including cell numbers, biological replicate numbers, total detected genes, percentage of detected genes, mean read counts per cell and median number of genes detected per cell. Fig. S2. A summary of the xylem cells identified from previous studies. The previous studies with sizable cell numbers from xylem-related cell types are used in this study with highlighted light red color as the background. Four sets of cells from Ryu et al. (907 cells from xylem containing stele) [20], Lopez-Anido et al. (1004 cells from vasculature) [29], Zhang et al. (xylem cell number was not available (NA)) [28] and Chen et al. (3170 cells from wood tissue) [44] are selected. Using the marker genes in the three studies with Arabidopsis or Rice, total 353, 30 and 2508 xylem cells are identified. Fig. S3. Cell numbers of SDX scRNA-seq cell clusters in P. trichocarpa, E. grandis, T. aralioides and L. chinense. (A–D) SDX single-cell transcriptomes of P. trichocarpa (A), E. grandis (B), T. aralioides (C) and L. chinense (D) were grouped using unsupervised K-means clustering into 10 cell clusters in each species. The number of cells is shown for each cell cluster. Fig. S4. Workflow of laser capture microdissection for three xylem cell types in P. trichocarpa. (A) Schematic of transverse and tangential sectioning of tree stems. A whole stem segment and a quarter stem segment are loaded on cryostat chucks for transverse and tangential sectioning, respectively. Sections are then placed on metal-frame slides with PET membrane for subsequent LCM cell type isolation. (B) Real sections before and after laser cutting of libriform fibers, vessel elements and ray parenchyma cells. The area within the green circles represen [file 13059_2022_2845_MOESM1_ESM.pdf]

	P. trichocarpa		*E. grandis*	*T. aralioides*	*L. chinense*
Cells	19,007	6,159	9,009	4,972	2,977
Biological replicates	2	5	5	4	1
Minimal UMI counts	500	100	100	100	500
Detected genes	28,691	27,581	23,086	26,226	17,926
Detected genes coverage	82.7%	79.5%	63.5%	74.2%	50.8%
Mean reads/cell	50,605	11,837	12,910	15,406	119,952
Median genes/cell	1,538	539	564	274	1,030

Fig. S1. Summary of scRNA-seq assays of SDX in *P. trichocarpa*, *E. grandis*, *T. aralioides* and *L. chinense*. Statistics of scRNA-seq profiling by 10x Chromium (red) or MARS-seq2.0 (green) are shown for cells with at least 500 and 100 UMIs, respectively, including cell numbers, biological replicate numbers, total detected genes, percentage of detected genes, mean read counts per cell and median number of genes detected per cell.

Reference	Organ source	Xylem-related cell type	# of cells
Denyer et al., 2019	*Arabidopsis* root	Xylem	143
Jean-Baptiste et al., 2019	*Arabidopsis* root	Xylem	72
Ryu et al., 2019	*Arabidopsis* root	Xylem containing stele	907 (353)
Shulse et al., 2019	*Arabidopsis* root	Protoxylem	About 500
Shulse et al., 2019	*Arabidopsis* root	Meristematic xylem	About 100
Turco et al., 2019	*Arabidopsis* root	Protoxylem	347
Wendrich et al., 2020	*Arabidopsis* root	Xylem	251
Kim et al., 2021	*Arabidopsis* leaf	Xylem parenchyma	173
Xu et al., 2021	Maize ear	Xylem	626
Zhang et al., 2021	*Arabidopsis* shoot apex	Xylem	1205
Liu et al., 2021	Rice root	Metaxylem	49
Lopez-Anido et al., 2021	*Arabidopsis* leaf	Vasculature	1004 (30)
Zhang et al., 2021	Rice root	Xylem	NA (2508)
Li et al., 2021	Poplar stem	Differentiating xylem	9798
Chen et al., 2021	Poplar stem	Wood tissue	3170

Fig. S2. A summary of the xylem cells identified from previous studies. The previous studies with sizable cell numbers from xylem-related cell types are used in this study with highlighted light red color as the background. Four sets of cells from Ryu et al. (907 cells from xylem containing stele) [20], Lopez-Anido et al. (1004 cells from vasculature) [29], Zhang et al. (xylem cell number was not available (NA)) [28] and Chen et al. (3170 cells from wood tissue) [44] are selected. Using the marker genes in the three studies with *Arabidopsis* or Rice, total 353, 30 and 2508 xylem cells are identified.

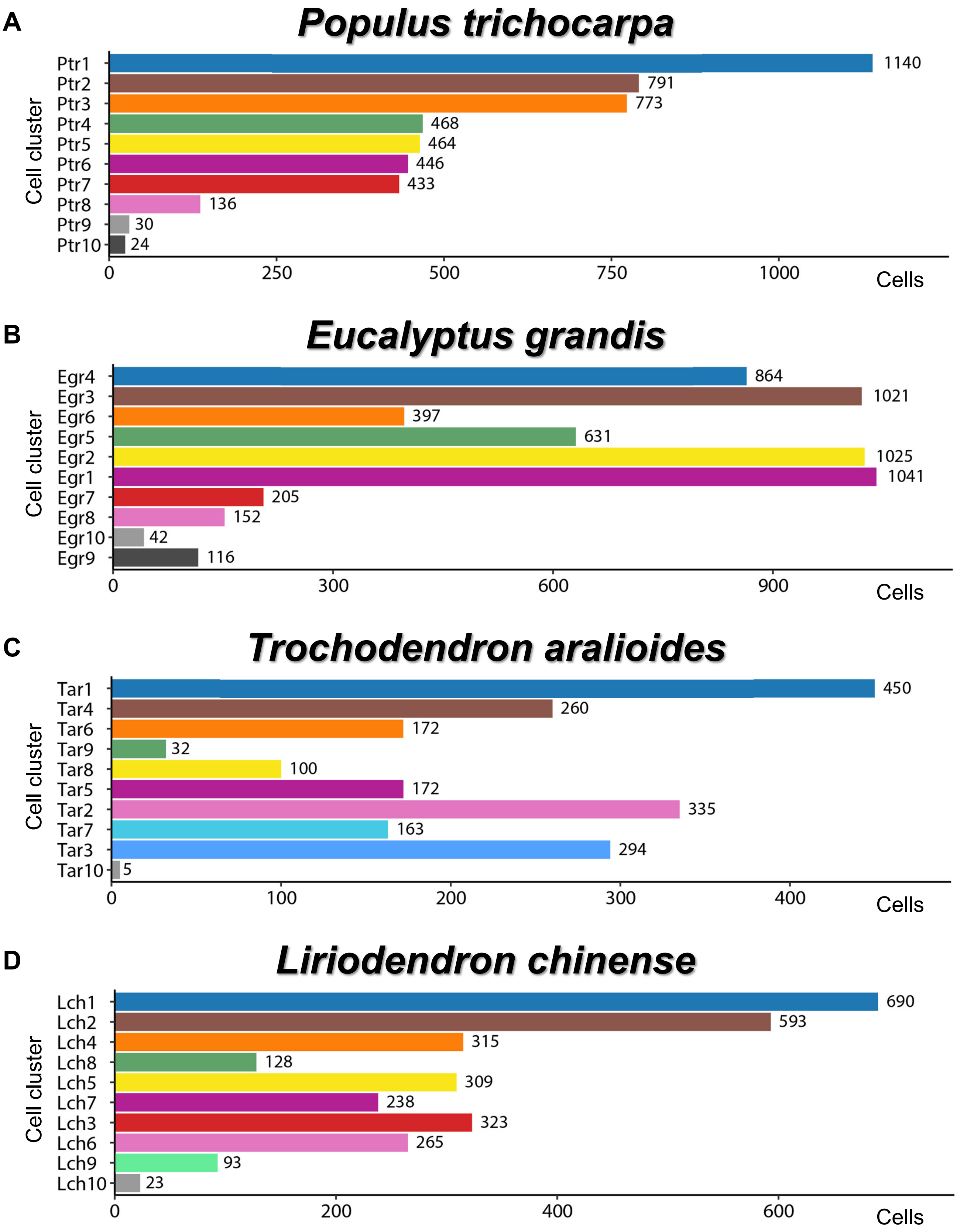

Fig. S3. Cell numbers of SDX scRNA-seq cell clusters in *P. trichocarpa*, *E. grandis*, *T. aralioides* and *L. chinense*. (A–D) SDX single-cell transcriptomes of *P. trichocarpa* (A), *E. grandis* (B), *T. aralioides* (C) and *L. chinense* (D) were grouped using unsupervised K-means clustering into 10 cell clusters in each species. The number of cells is shown for each cell cluster.

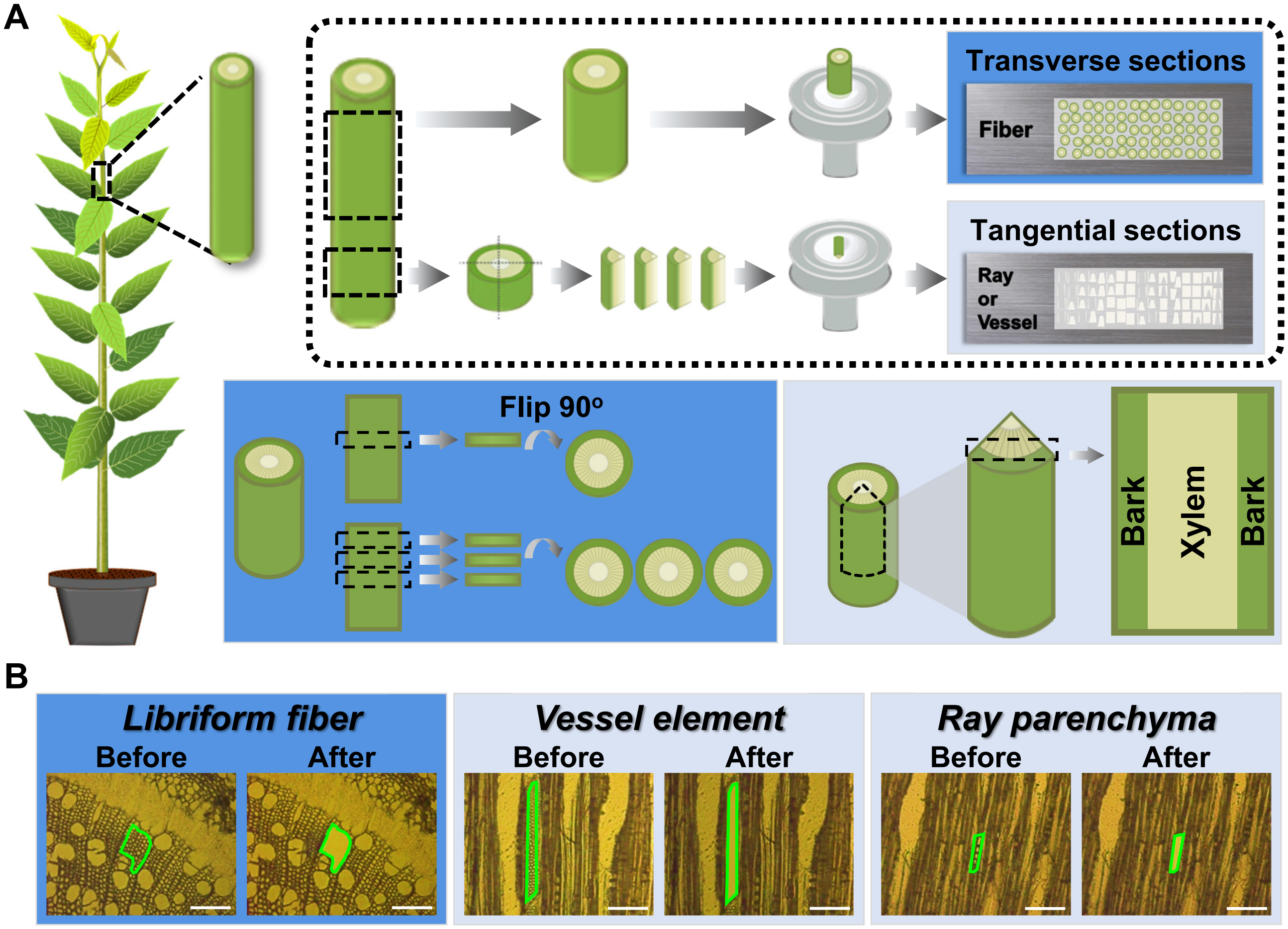

Fig. S4. Workflow of laser capture microdissection for three xylem cell types in *P. trichocarpa*. (A) Schematic of transverse and tangential sectioning of tree stems. A whole stem segment and a quarter stem segment are loaded on cryostat chucks for transverse and tangential sectioning, respectively. Sections are then placed on metal-frame slides with PET membrane for subsequent LCM cell type isolation. (B) Real sections before and after laser cutting of libriform fibers, vessel elements and ray parenchyma cells. The area within the green circles represents the cutting area. Scale bars, 100 µm. In (A) and (B), transverse and tangential sections are marked with dark-blue and light-blue backgrounds, respectively.

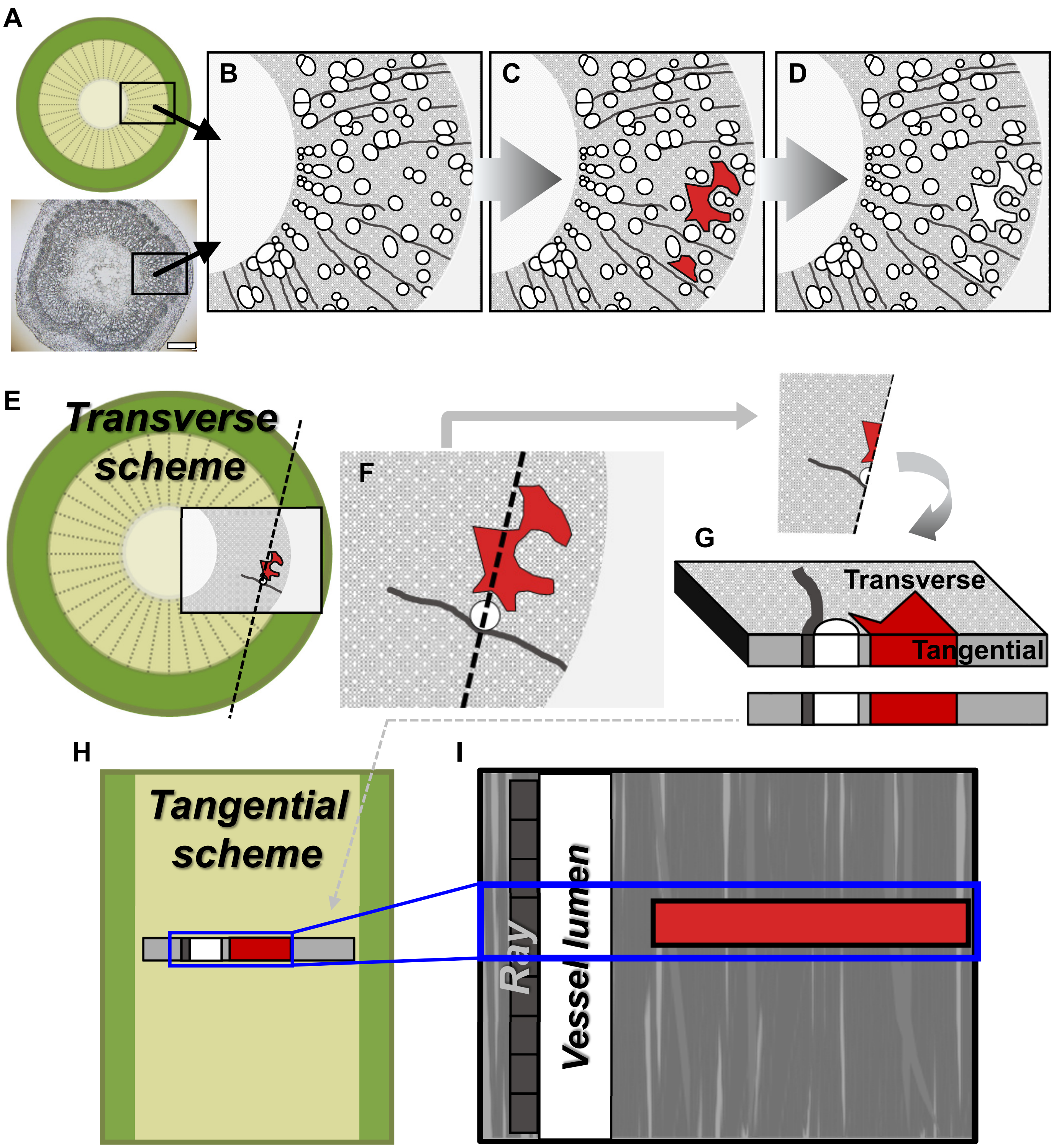

Fig. S5. Libriform fiber collection in transverse and tangential views. (A–I) The red area represents the libriform fibers collected using LCM. Libriform fiber collection from transverse sections, showing before cutting (B), area selection (C) and after cutting (D). Scale bar, 500 µm. In (C) and (D), the area switched from red to white, leaving an empty space after the libriform fibers were cut by laser. Libriform fibers in transverse and tangential views (E–I). A transverse section with an area highlighted (red) for libriform fiber collection (E). A closeup of the highlighted area (F). Three-dimensional structure around the highlighted area (G). A tangential section with highlighted area (H). Corresponding panel of Fig. 1E (I).

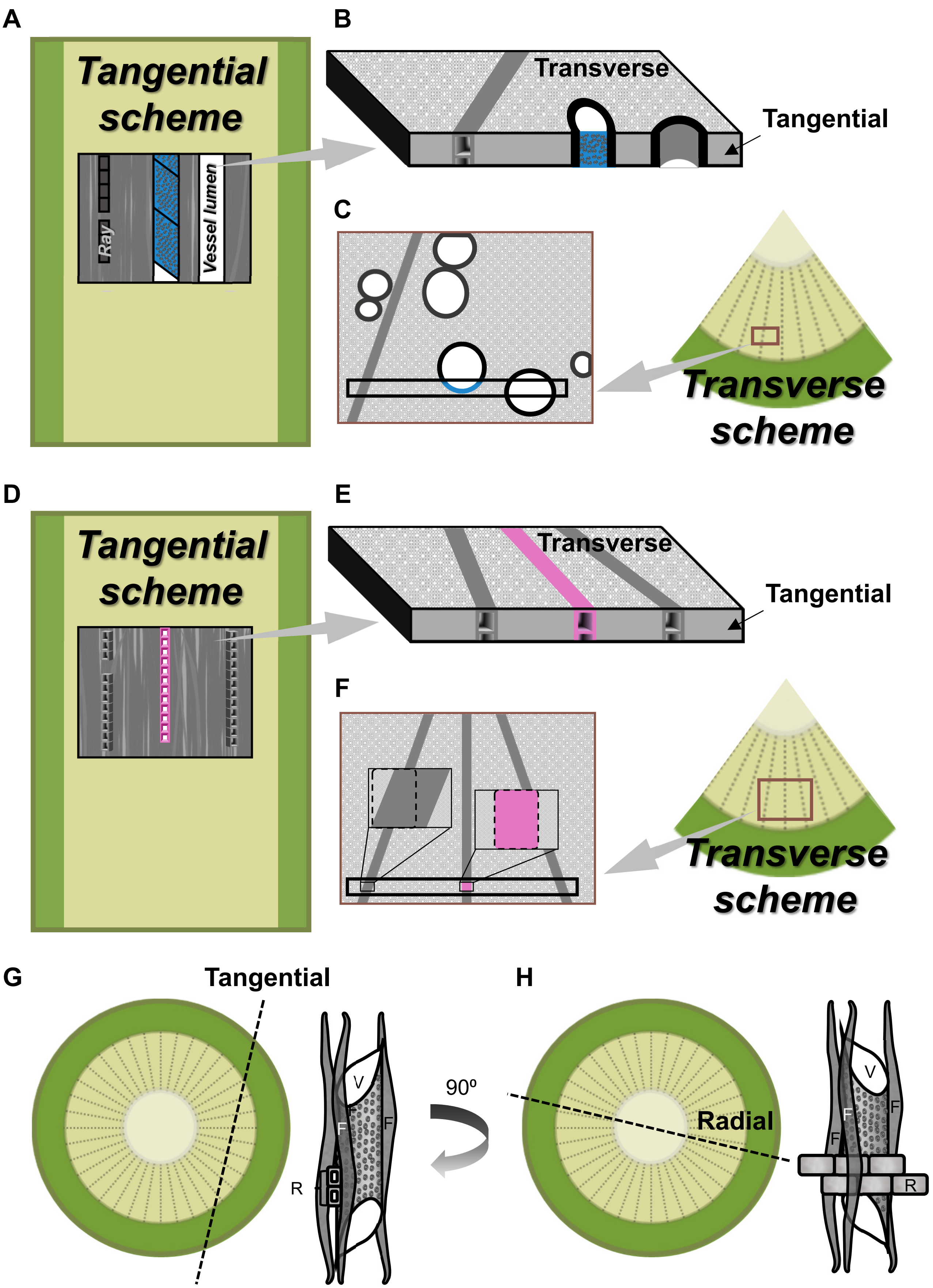
A
Tangential scheme
Ray
Vessel lumen
B
Transverse
Tangential
C
Transverse scheme
D
Tangential scheme
E
Transverse
Tangential
F
Transverse scheme
G
Tangential
V
F
F
R
90º
H
Radial
V
F
F
F
R

Fig. S6. The illustrations for vessel element and ray parenchyma cell collection from transverse and tangential perspectives. (A–C) The schematics of vessel elements from transverse and tangential perspectives. The blue area represents the vessel elements collected using LCM. In (A), the corresponding figure of Fig. 1I represents a tangential section with an area highlighted (blue) for vessel element collection. In (B), a three-dimensional structure of the highlighted area shows the location of the collected vessel elements in the stem structure schematic. In (C), the corresponding figure of Fig. 1H represents a transverse section with highlighted area. (D–F) The schematics of ray parenchyma cells from transverse and tangential perspectives. The pink area represents the ray parenchyma cells collected using LCM. In (D), the corresponding figure of Fig. 1M represents a tangential section with a highlighted area (pink). In (E), a three-dimensional structure of the highlighted area shows the location for the collected ray parenchyma cells in the stem structure schematic. In (F), the corresponding figure of Fig. 1L represents a transverse section of the highlighted area. (G and H) The three-dimensional arrangement of three xylem cell types from tangential (G) and radial (H) perspectives. F, libriform fibers. V, vessel elements. R, ray parenchyma cells.

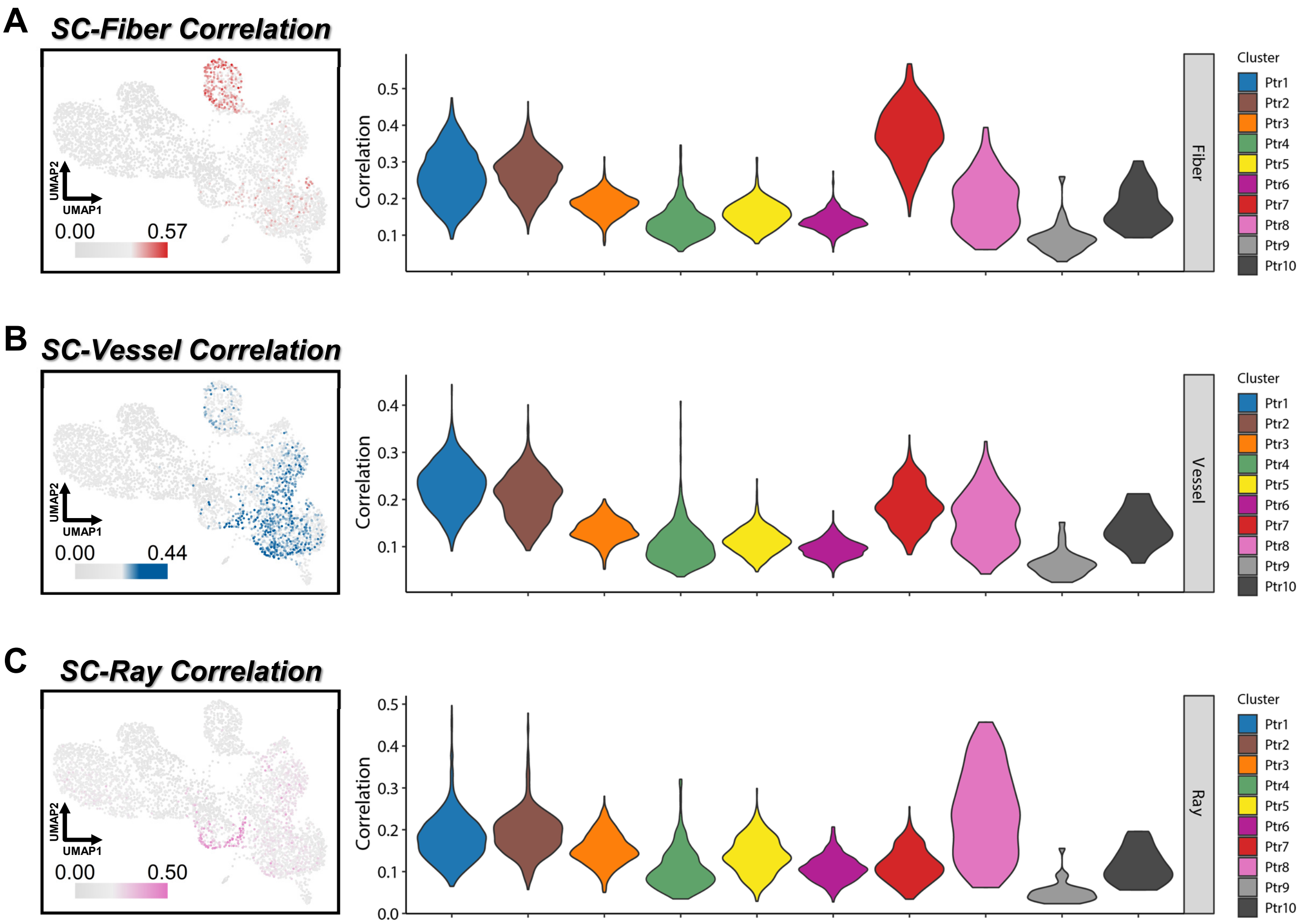

Fig. S7. Violin plots of the transcriptomic correlation between each cell type of lcmRNA-seq and each cell cluster of scRNA-seq results. (A–C) The correlations between scRNA-seq results with lcmRNA-seq results of libriform fibers (A), vessel elements (B) or ray parenchyma cells (C), respectively.

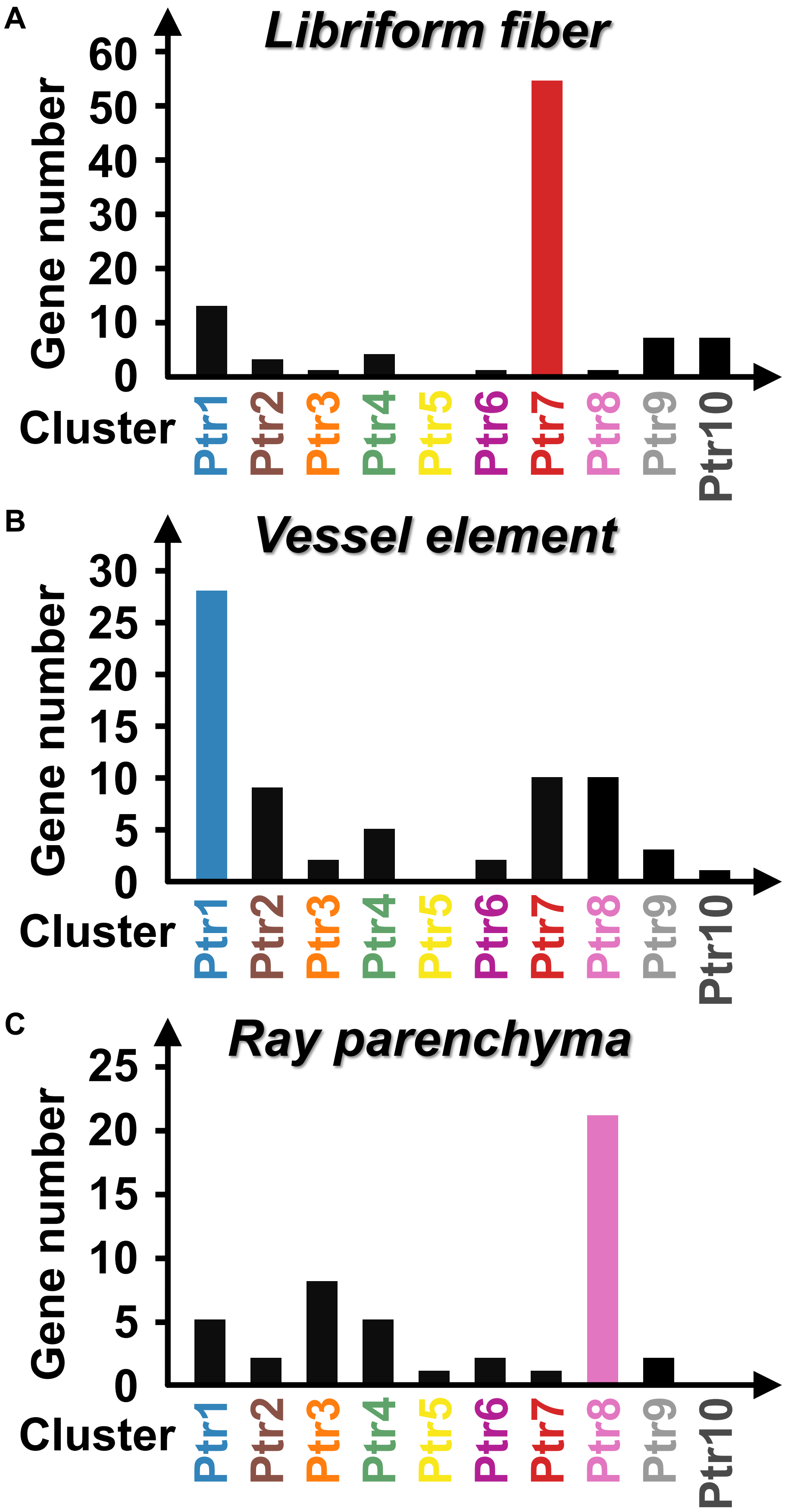

Fig. S8. Bar charts of the scUPlcmUP gene numbers. (A–C) The scUPlcmUP gene numbers of libriform fibers (A), vessel elements (B) and ray parenchyma cells (C). Only the lcmUP genes with average transcripts per million (TPM) more than 4 were included.

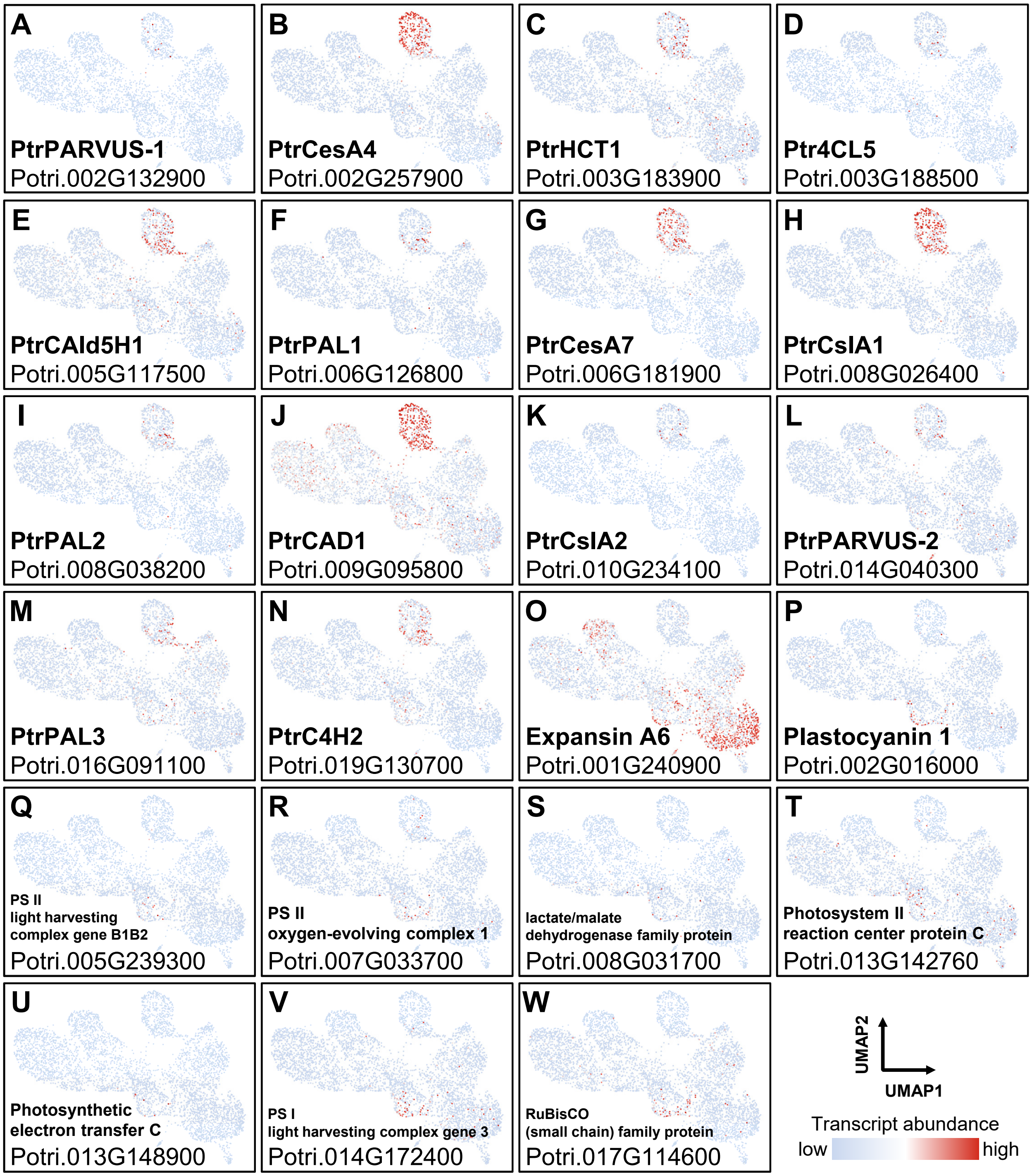

Fig. S9. Previous known genes in the scUPlcmUP genes. (A–N) Secondary cell wall biosynthesis genes expressed in libriform fibers. (O) The expansin gene expressed in vessel elements. (P–W) The photosynthesis-related genes expressed in ray parenchyma cells.

Log2 Fold Change

Common name	SCW	Ptr7	Ptr8	Ptr4	Ptr2	Ptr5	Ptr1	Ptr6	Ptr3
PtrCesA4	Cellulose	8	0	0	-4	-3	-3	-3	-5
PtrCesA7	Cellulose	8	0	-2	-3	-4	-3	-3	-5
PtrCslA1	Hemi-cellulose	8	0	-2	-2	-3	-3	-4	-5
PtrCslA2	Hemi-cellulose	7	0	0	0	0	0	-3	-3
PtrPARVUS-1	Hemi-cellulose	6	0	0	0	0	0	0	-2
PtrPARVUS-2	Hemi-cellulose	2	0	0	-3	-2	0	1	-2
Ptr4CL5	Lignin	5	0	0	0	0	0	0	0
PtrC4H2	Lignin	3	2	0	-1	0	-3	-3	0
PtrCAD1	Lignin	3	0	0	-1	-1	-1	0	0
PtrCAld5H1	Lignin	3	0	0	0	0	-2	0	-1
PtrHCT1	Lignin	4	0	0	0	-2	0	-1	-3
PtrPAL1	Lignin	4	0	0	0	0	-2	-2	0
PtrPAL2	Lignin	5	0	0	0	0	0	-2	-1
PtrPAL3	Lignin	2	0	0	1	0	0	-3	0

Fig. S10. Relative transcript abundance of the secondary cell wall biosynthesis genes in the cell clusters. The differentially expressed genes are marked as red for up-regulations and green for down-regulations.

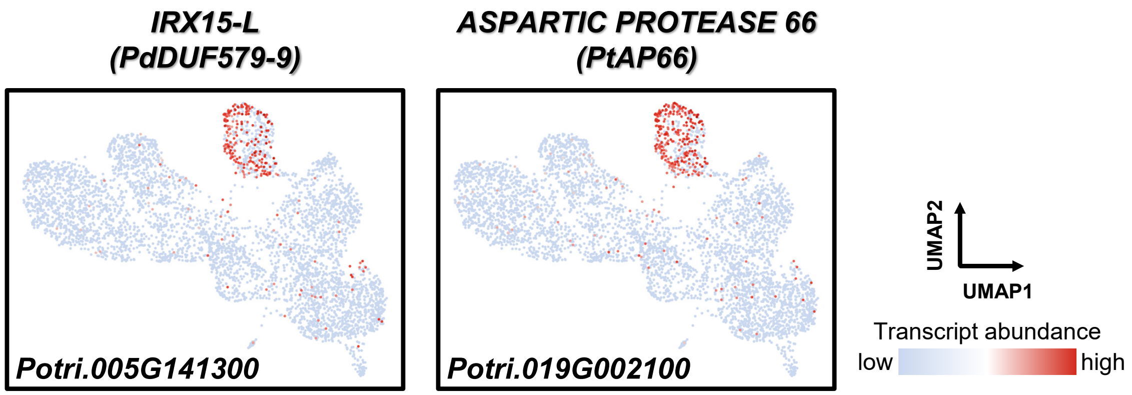

Fig. S11. Known libriform fiber marker genes. The exclusive expression of *IRX15-L* (*PdDUF579-9*) and *ASPARTIC PROTEASE 66* (*PtAP66*) in libriform fibers.

P. trichocarpa gene ID	Arabidopsis best-hit	Transcript abundance	Transcript proportion
Potri.008G057100	Expansin A4	8	2%
Potri.010G202500		26	
**Potri.001G240900**	Expansin A6	646	**45%**
Potri.009G031800		342	24%
Potri.001G112900	Expansin A7	1	0%
Potri.013G154700	Expansin A8	2	0%
Potri.016G135200		0	
Potri.019G057500		1	
Potri.002G017900	Expansin A11	0	0%
Potri.005G244100		0	
Potri.001G401700	Expansin A12	0	0%
Potri.004G080200	Expansin A13	17	9%
Potri.017G140000		106	
Potri.008G088300	Expansin A15	62	7%
Potri.010G167200		33	
Potri.002G184700	Expansin A17	0	0%
Potri.004G208300	Expansin A20	0	0%
Potri.009G169500		3	
Potri.017G092700	Expansin A21	0	0%
Potri.014G066300	Expansin B2	0	0%
Potri.013G134300	Expansin B3	8	6%
Potri.019G101900		80	
Potri.004G181700	Expansin-like A3	57	7%
Potri.009G141400		38	
Potri.001G151500	Expansin-like B1	0	0%
Potri.003G083200		0	

Fig. S12. Transcript abundance of the *P. trichocarpa* expansin genes. The *P. trichocarpa* homologs of *A. thaliana* expansin genes are listed with their corresponding transcript abundance and their transcript proportion in SDX among all expansin transcript abundance.

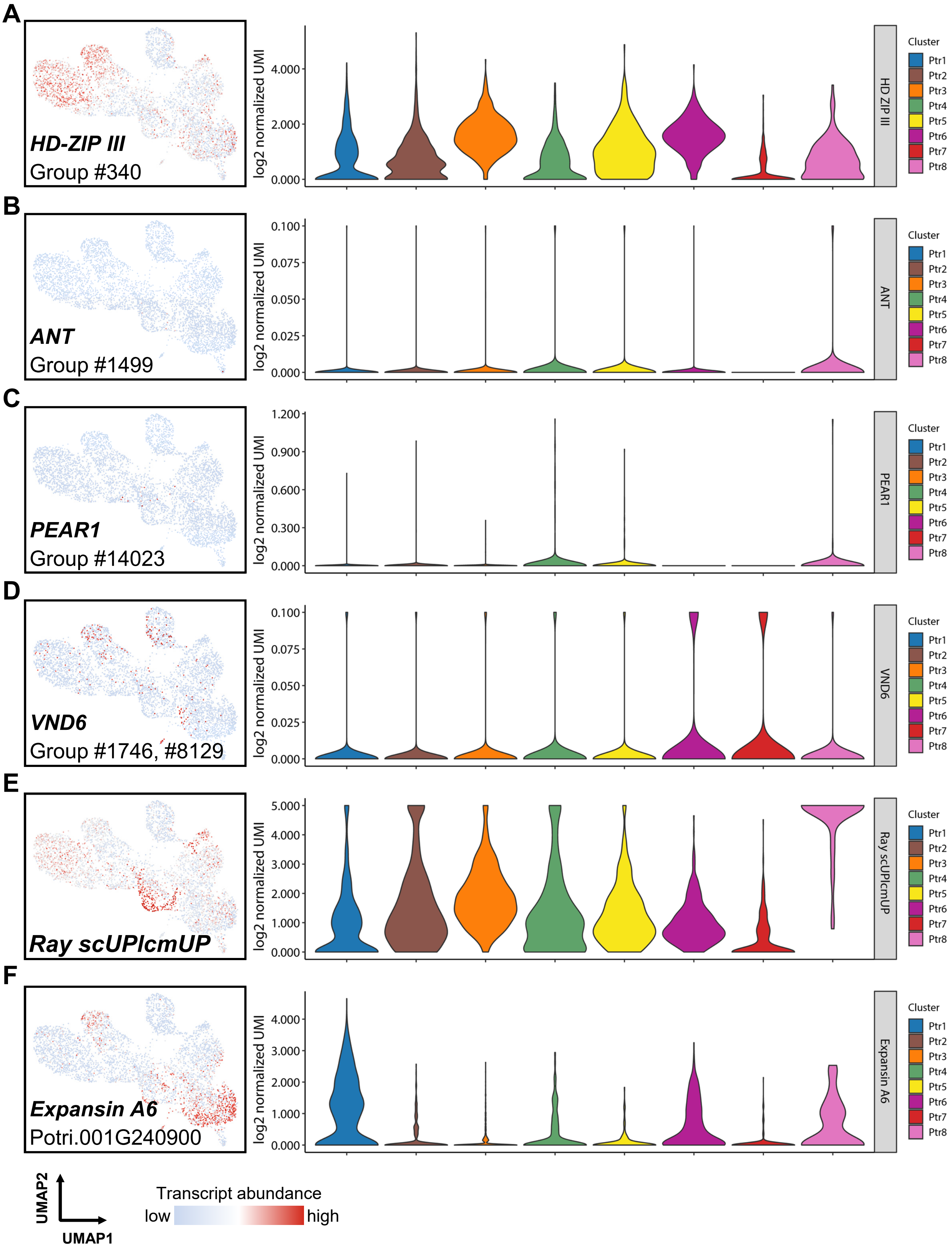

Fig. S13. Violin plots of the transcript abundance of each orthologous group in the scRNA-seq results. (A–F) Violin plots are used to reveal the $\log_2$ normalized UMI counts of each orthologous group in each cell cluster.

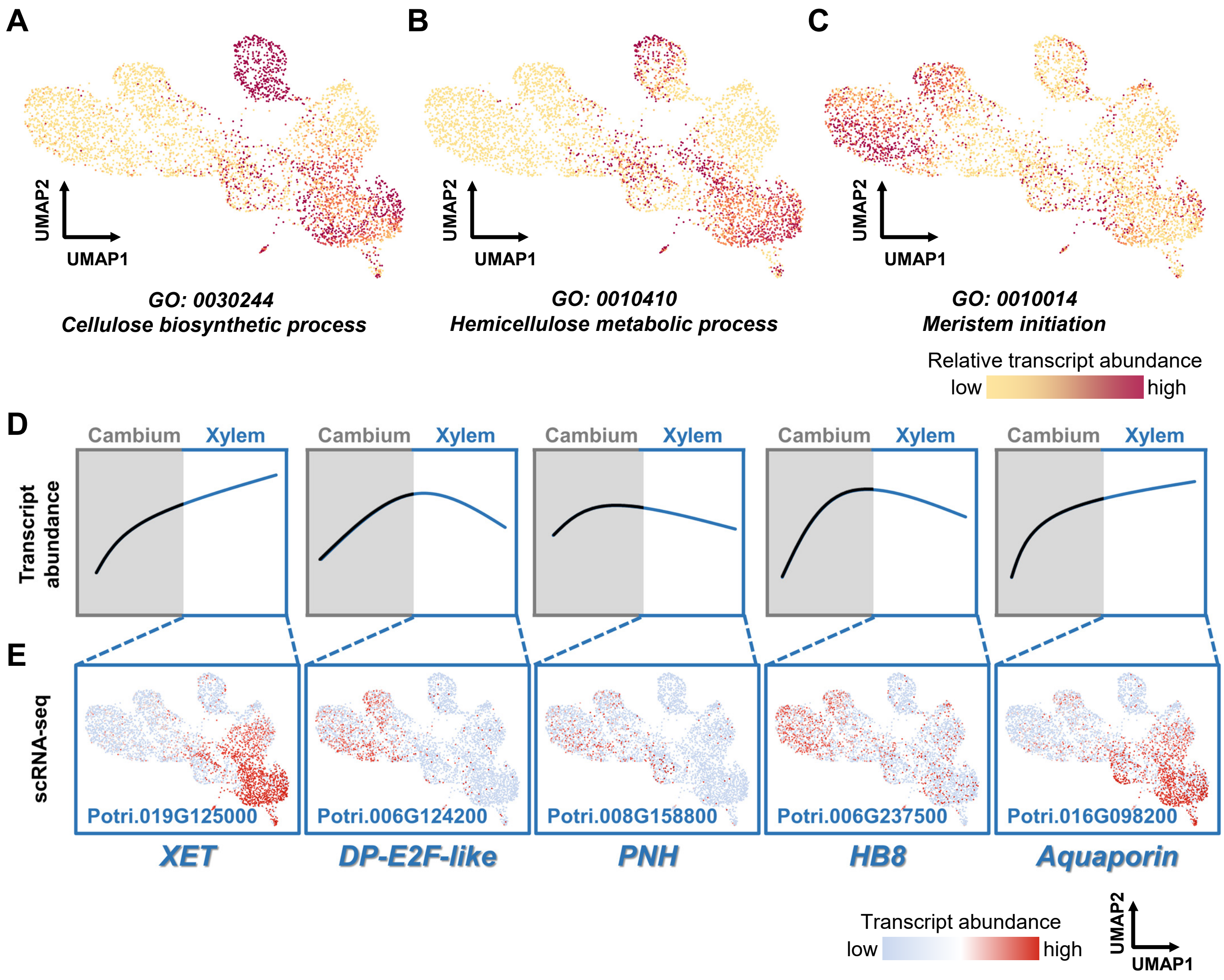

Fig. S14. Transcript abundance of the genes from cellulose-, hemicellulose-, meristem-related GO terms and known marker genes across cambial and SDX regions. (A–C) Relative transcript abundance of cellulose- (A), hemicellulose- (B), and meristem-related (C) genes. The GO terms for cellulose biosynthetic process, hemicellulose metabolic process, and meristem initiation are GO: 0030244, GO: 0010410, and GO: 0010014, respectively. (D–E) Time-course transcriptomic analyses across cambial and SDX regions. A schematic reproduction of gene expression results across the cambial and SDX regions from a previous study [52] conducted using a series of 20-µm tangential sections (D). The corresponding transcript abundance in the scRNA-seq results in this study (E).

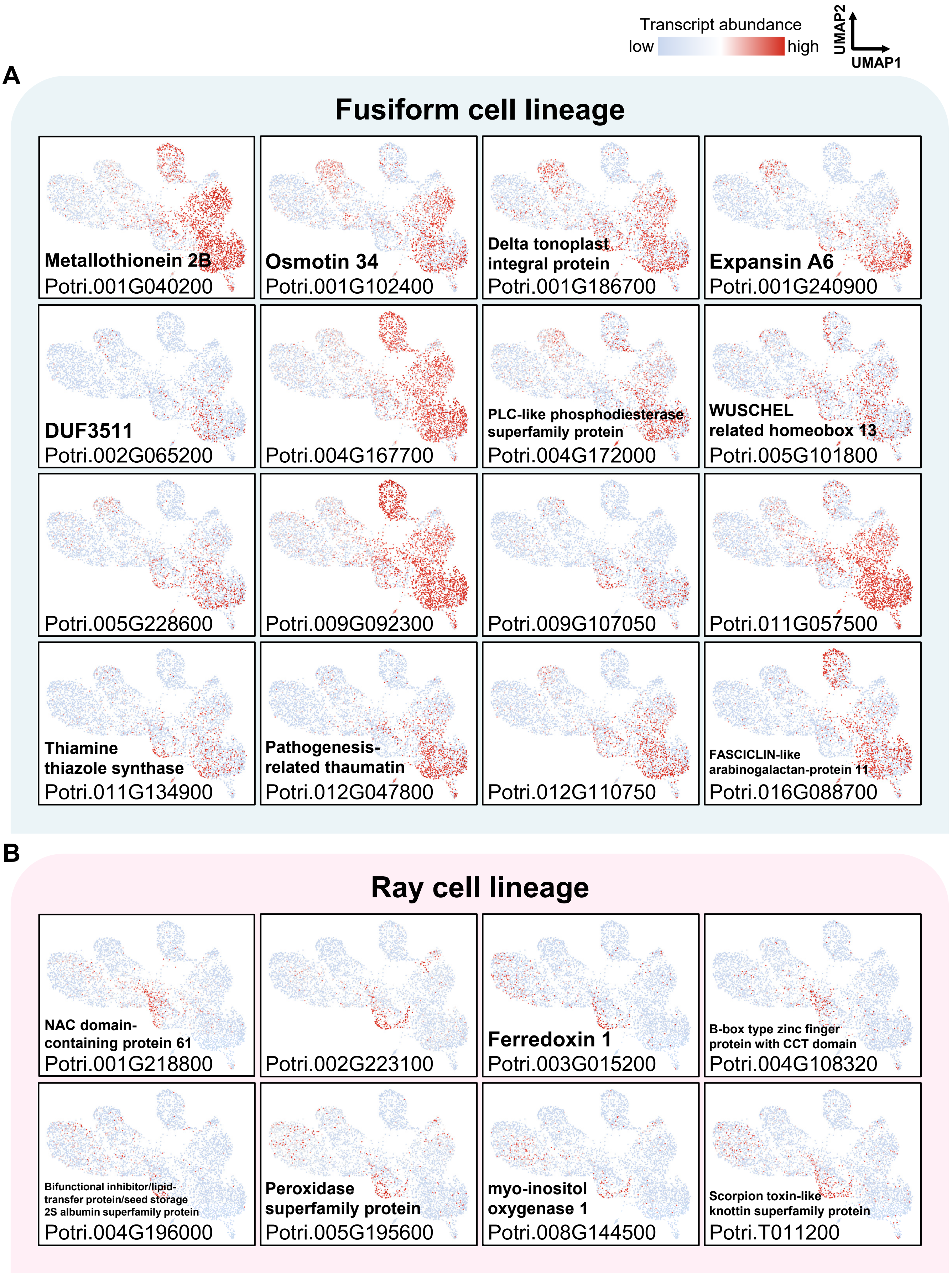

Fig. S15. Transcript abundance of up-regulated genes in scRNA-seq results of vessel elements and ray parenchyma cells reveals fusiform and ray cell lineages. (A and B) The gene expression profiles of the up-regulated genes in vessel elements and ray parenchyma cells on the UMAP show continuous cell lineages for fusiform (A) and ray cells (B), respectively.

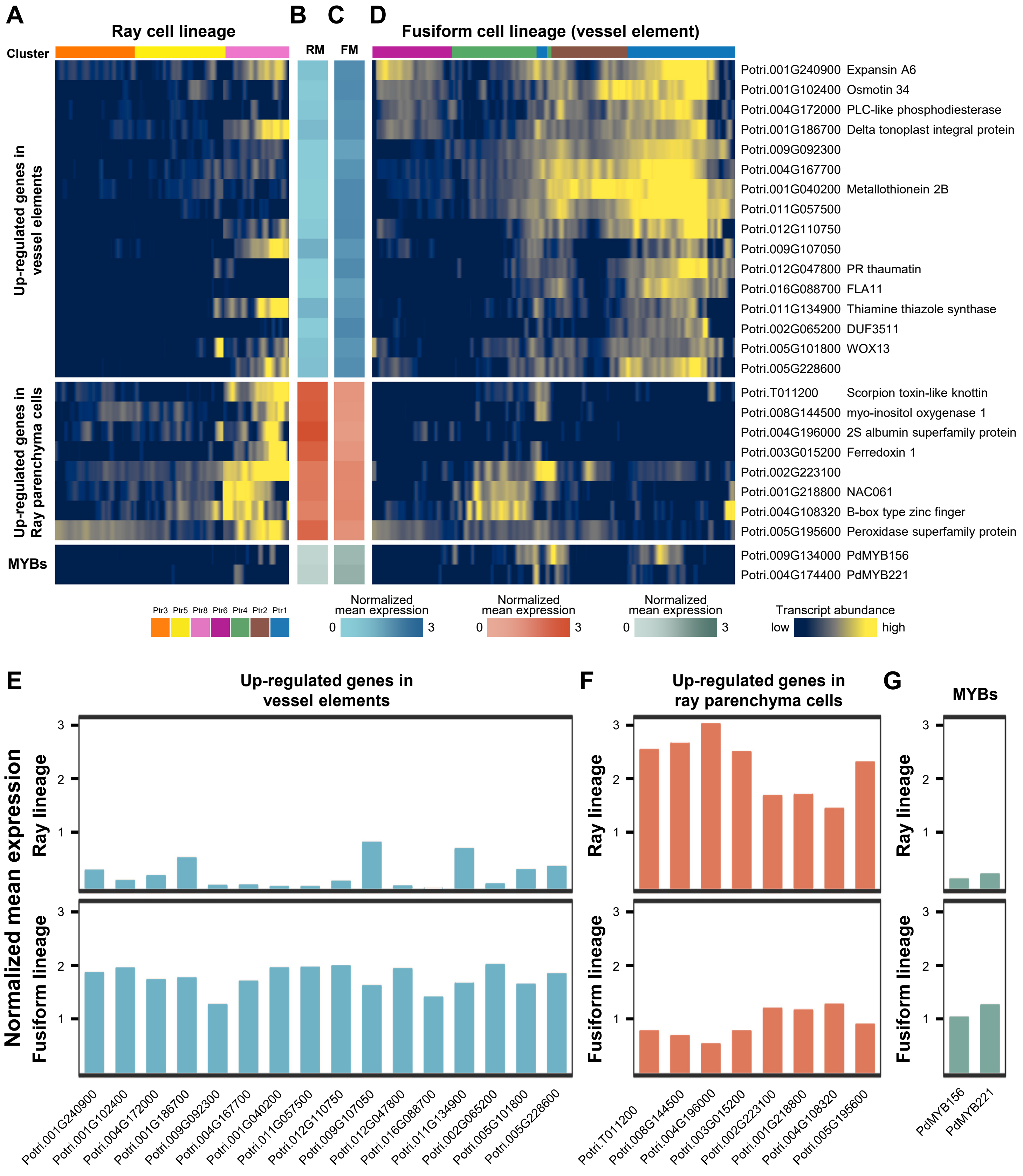

Fig. S16. Transcript abundance of up-regulated genes in each cell type across fusiform and ray lineages. (A) Transcript abundance of the up-regulated genes across ray lineage. (B) The normalized mean expression in ray lineage. (C) The normalized mean expression in fusiform lineage. (D) Transcript abundance of the up-regulated genes across fusiform lineage. (E) to (F) Bar charts represent the normalized mean expression of the up-regulated genes and the homologs of *PdMYB156* and *PdMYB221* in *P. trichocarpa* of fusiform lineage and ray lineage, respectively. Normalized mean expression was calculated as the mean UMI counts of each gene normalized with mean UMI counts of all genes. RM, normalized mean expression in ray lineage. FM, normalized mean expression in fusiform lineage.

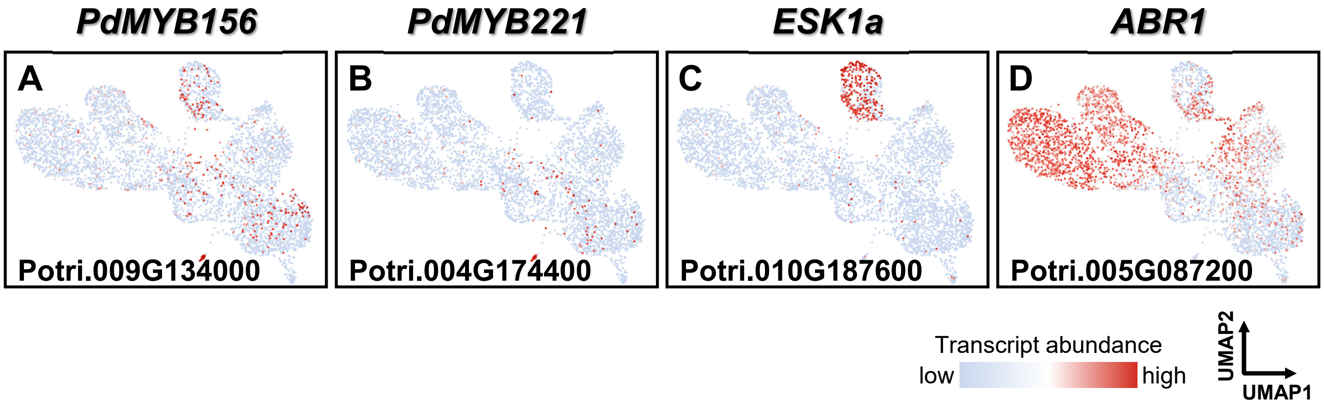

Fig. S17. Expression pattern of known marker genes. (A–D) The transcript abundance of *P. trichocarpa* homologous genes of *PdMYB156* (A), *PdMYB221* (B), *ESK1a* (C) and *ABR1* (D) in scRNA-seq results.

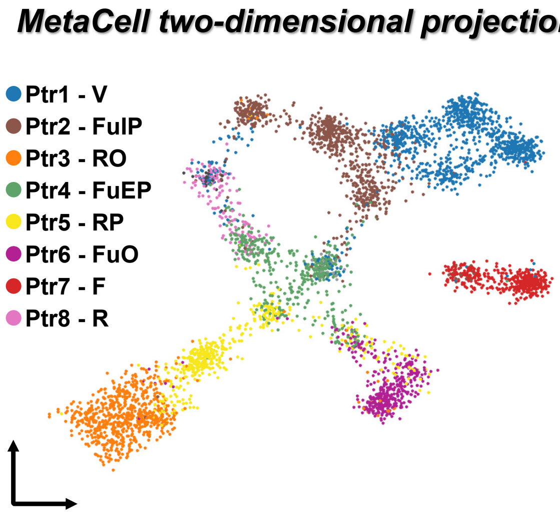

Fig. S18. MetaCell plot of the cell clusters in *P. trichocarpa*. Two-dimensional projection of *P. trichocarpa* SDX single cells using MetaCell dimensionality reduction. Cells are labeled with colors corresponding to those in Fig. 1B. V, vessel element. FuIP, fusiform intermediate precursor. RO, ray organizer. FuEP, fusiform early precursor. RP, ray precursor. FuO, fusiform organizer. F, libriform fiber. R, ray parenchyma cell.

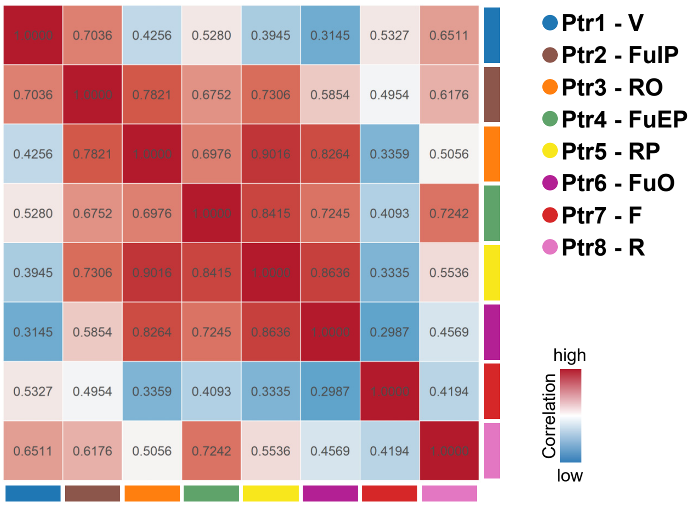

Fig. S19. Pairwise correlation of the cell clusters in *P. trichocarpa*. Pairwise correlation between transcriptomic profiles of Ptr1–Ptr8 of *P. trichocarpa* SDX. V, vessel element. FuIP, fusiform intermediate precursor. RO, ray organizer. FuEP, fusiform early precursor. RP, ray precursor. FuO, fusiform organizer. F, libriform fiber. R, ray parenchyma cell.

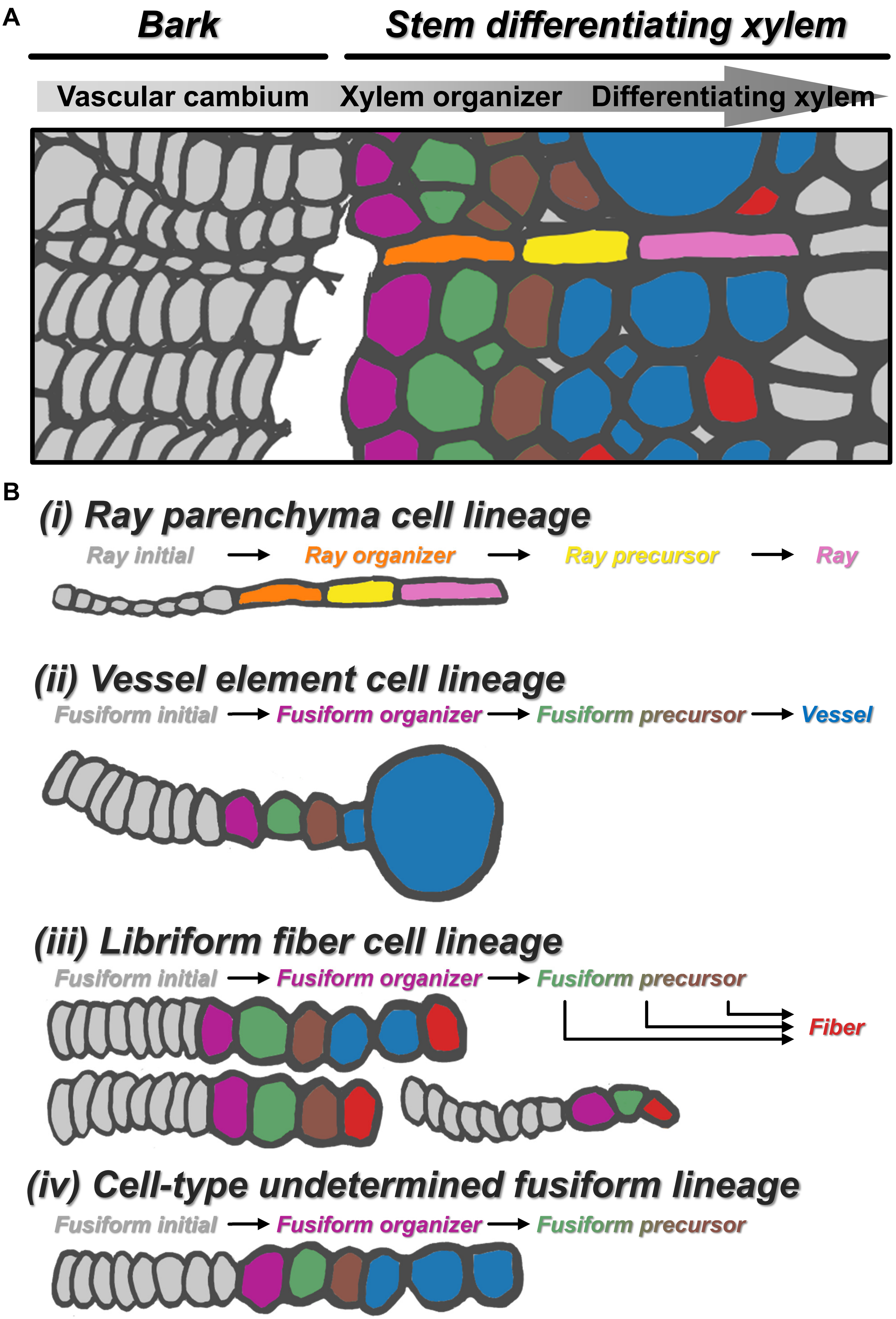
A
Bark
Stem differentiating xylem
Vascular cambium
Xylem organizer
Differentiating xylem
B
(i) Ray parenchyma cell lineage
Ray initial → Ray organizer → Ray precursor → Ray
(ii) Vessel element cell lineage
Fusiform initial → Fusiform organizer → Fusiform precursor → Vessel
(iii) Libriform fiber cell lineage
Fusiform initial → Fusiform organizer → Fusiform precursor
Fiber
(iv) Cell-type undetermined fusiform lineage
Fusiform initial → Fusiform organizer → Fusiform precursor

Fig. S20. Schematics of different developmental cell lineages in SDX in *P. trichocarpa*. (A) After stem debarking, the revealed SDX on the stem surface contains xylem organizer and differentiating xylem. (B) Schematic examples of four cell lineage types in SDX, including (i) ray parenchyma cell lineage, (ii) vessel element lineage, (iii) libriform fiber lineage and (iv) another incomplete or undergoing cell lineage as cell-type undetermined fusiform lineage. The first three lineages (i–iii) start with initials to organizers to precursors then to cell-type determined ray parenchyma cells, vessel elements or libriform fibers.

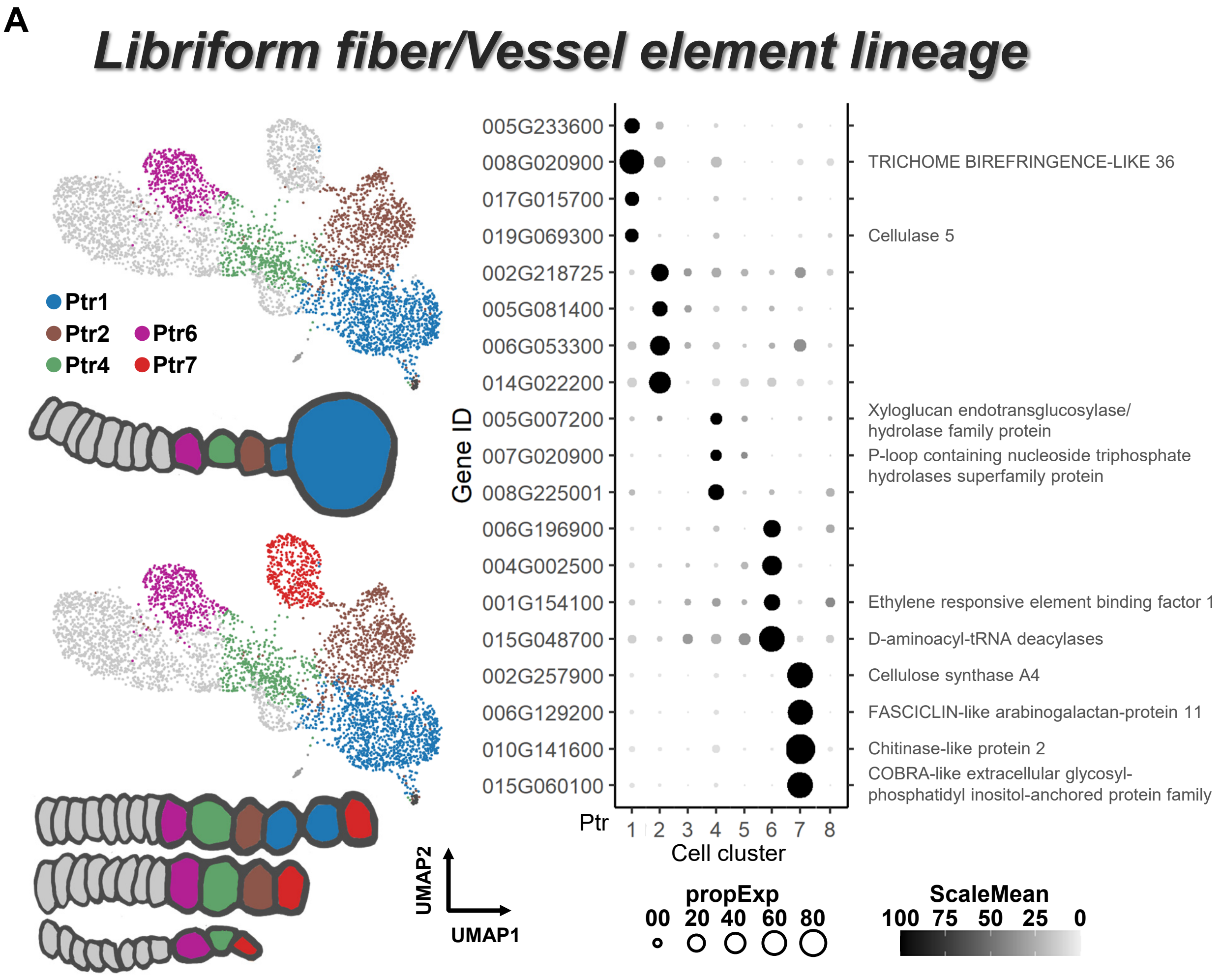

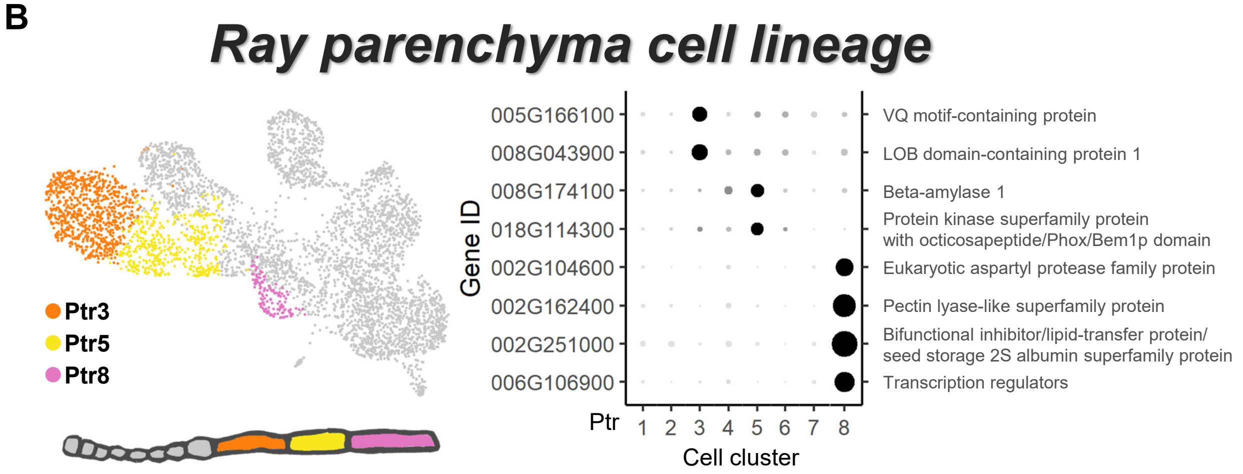

Fig. S21. Dot plots exhibit preferential patterns of marker genes in each cell cluster of libriform fiber/vessel element or ray parenchyma cell lineages. (A and B) The dot size represents the proportion of cells in each cell cluster with the marker gene expression, and the dot brightness shows the relative mean gene expression of the marker genes in libriform fiber/vessel element (A) or ray parenchyma cell lineages (B), respectively.

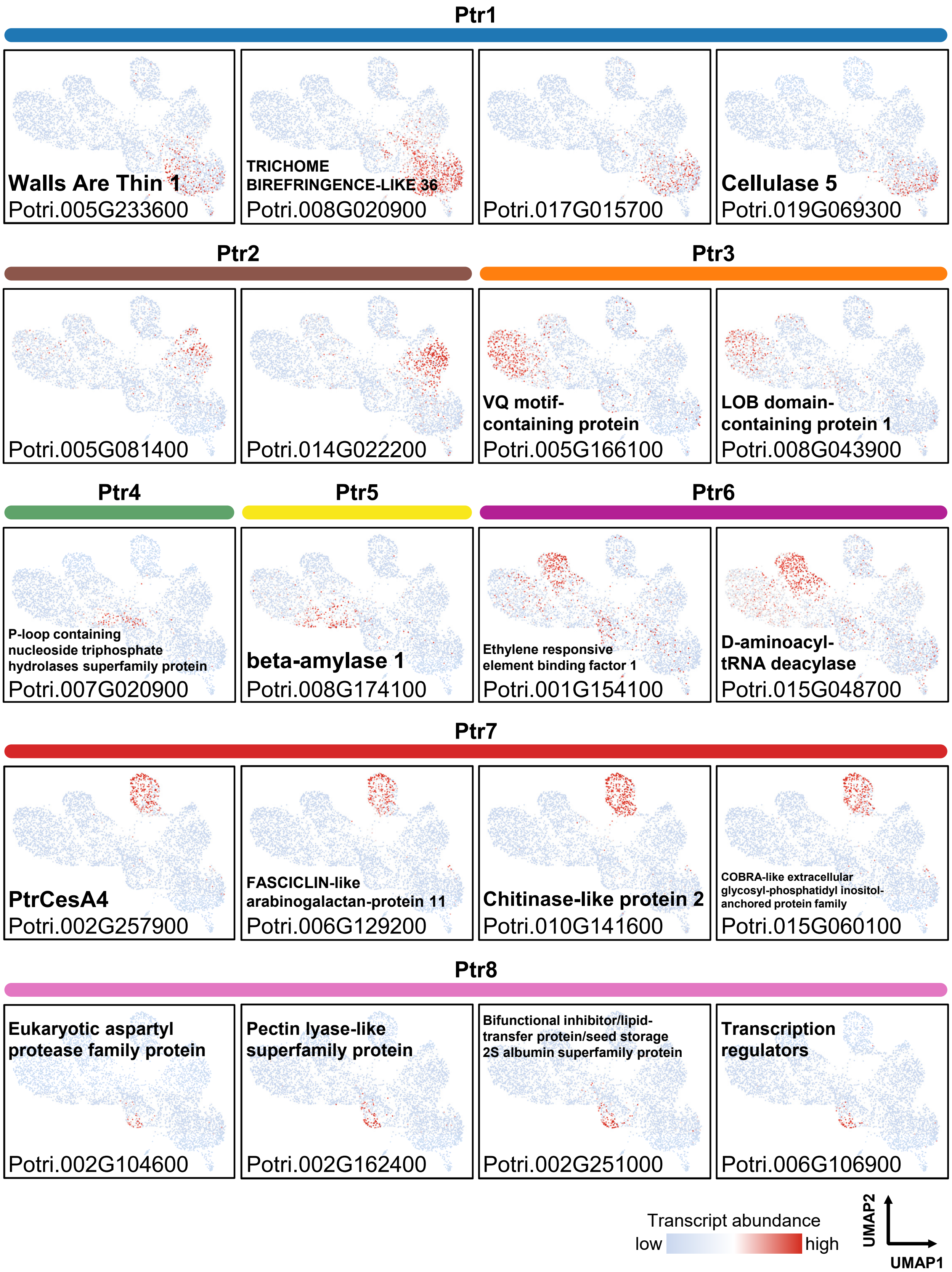

Fig. S22. Transcript abundance of marker genes of each cell cluster in *P. trichocarpa*. Many DEGs from each cell cluster are identified as marker genes if these genes show an exclusive expression in that cell cluster.

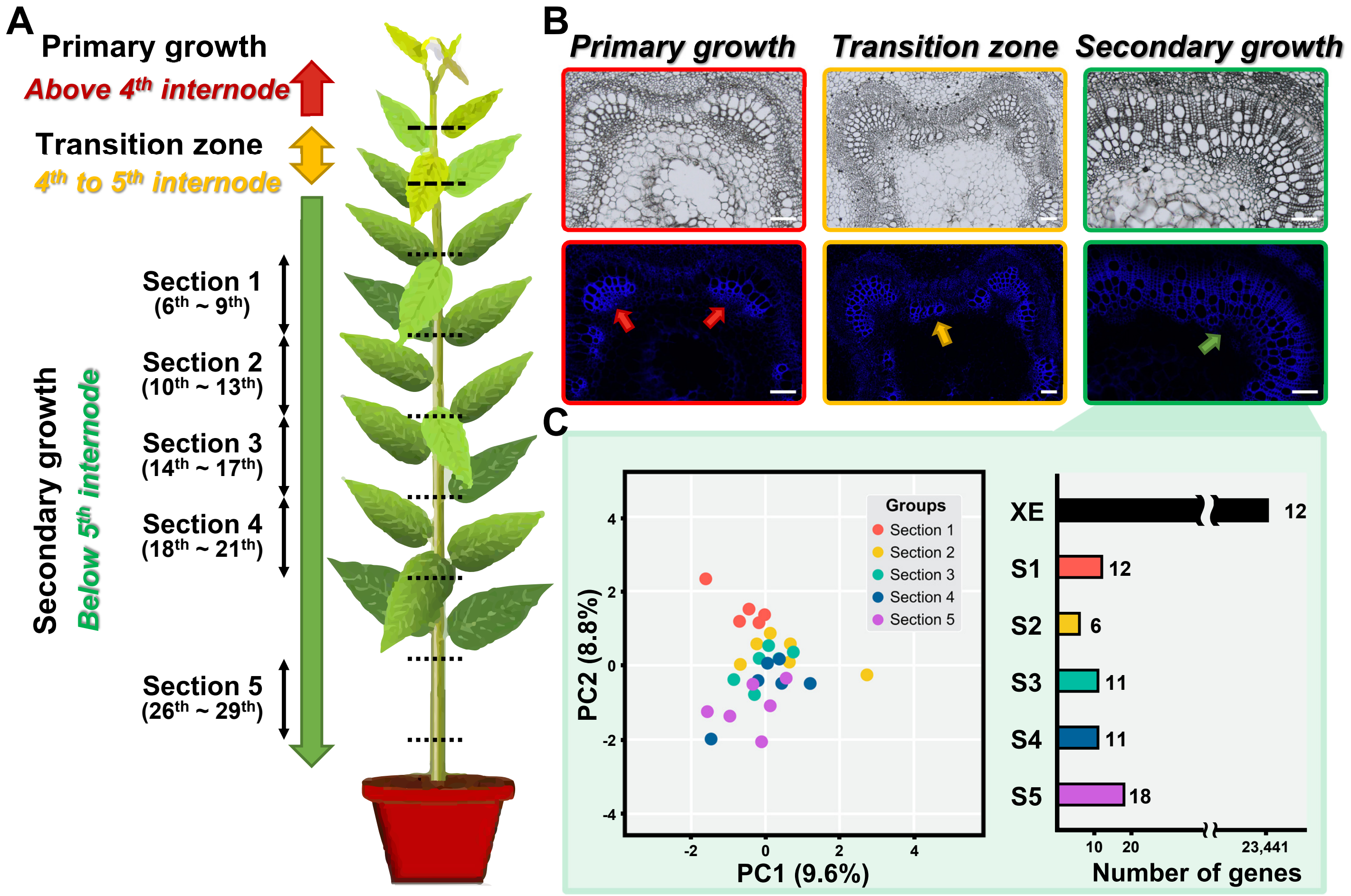

Fig. S23. Transcriptomic analyses of SDX across different internodes in *P. trichocarpa*. (A) Schematics of different stages in vertical growth in *P. trichocarpa*, including primary growth, transition zone and secondary growth. SDX from different sections, sections 1 to 5, were used for RNA-seq. (B) Anatomical analyses of SDX in different developmental stages. The cross-sections were observed under fluorescence microscope, and the blue signals represent deposited lignin. Red arrows indicated bundle vasculature. The yellow arrow showed the transition between bundle and circular vasculature. The green arrow indicated circular vasculature. Scale bars are 100 μm. (C) Principal component analysis and differential expression analyses were conducted using the SDX RNA-seq datasets from different internodes. Total 23,441 genes were expressed in secondary xylem, and 12/6/11/11/18 genes were differentially expressed in sections 1 to 5, respectively. XE, secondary xylem expressed genes. S1 to S5, sections 1 to 5.

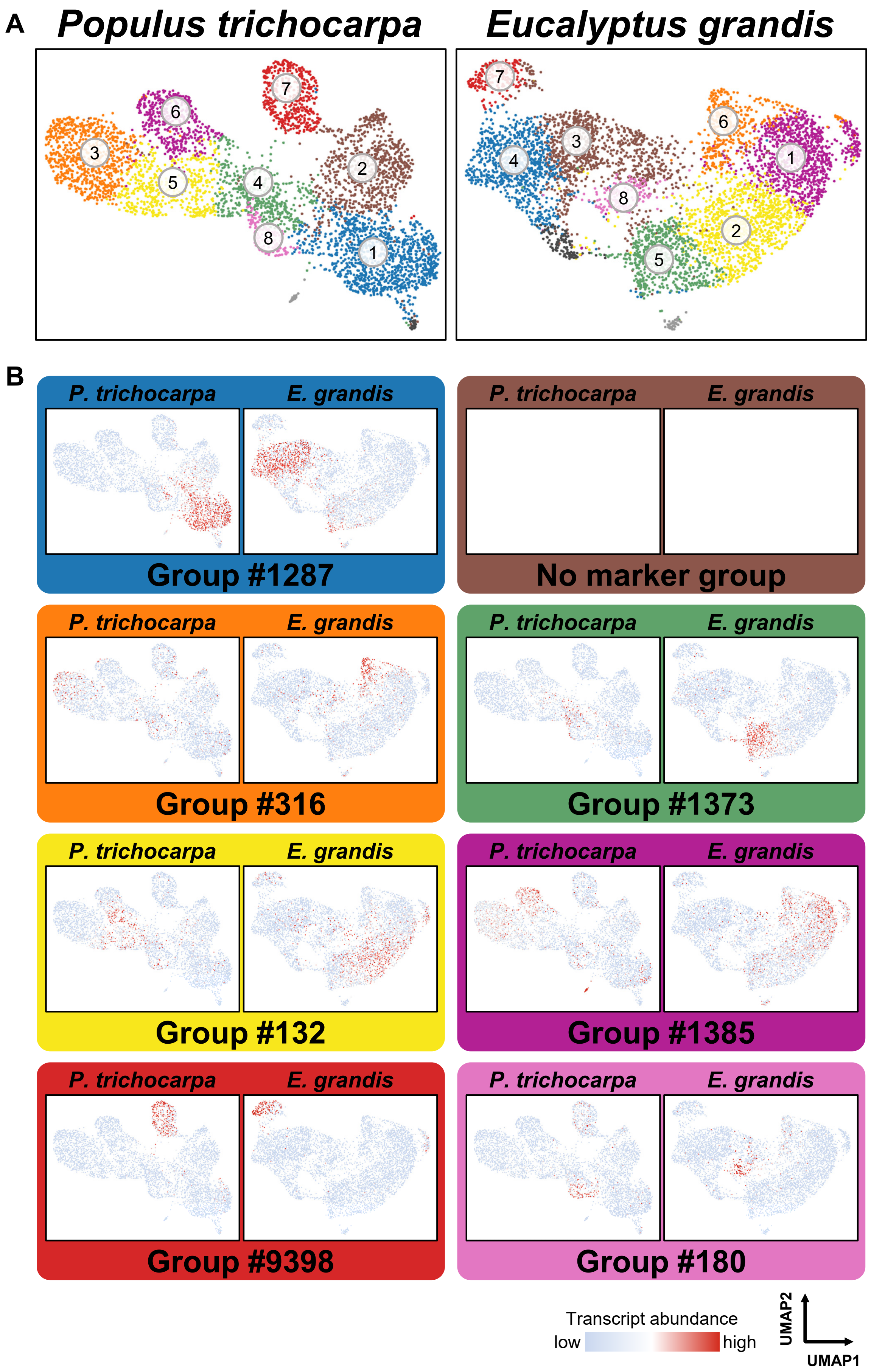

A
Populus trichocarpa
Eucalyptus grandis
B
P. trichocarpa
E. grandis
Group #1287
No marker group
Group #316
Group #1373
Group #132
Group #1385
Group #9398
Group #180
Transcript abundance
low
high
UMAP2
UMAP1

Fig. S24. Representative marker orthologous groups exclusively expressed in the different cell clusters in both *P. trichocarpa* and *E. grandis*. (A) The cell cluster plots obtained through unsupervised K-means clustering and UMAP are based on the SDX scRNA-seq results of *P. trichocarpa* and *E. grandis*, respectively. (B) The cluster-exclusive distributions of each marker orthologous group are represented by Group #1287 for vessel element/late fusiform precursor (blue), Group #316 for ray organizer (orange), Group #1373 for fusiform early precursor (green), Group #132 for ray precursor (yellow), Group #1385 for fusiform organizer (purple), Group #9398 for libriform fiber (red) and Group #180 for ray parenchyma cell (pink). No marker orthologous groups are observed in fusiform intermediate precursor (brown).

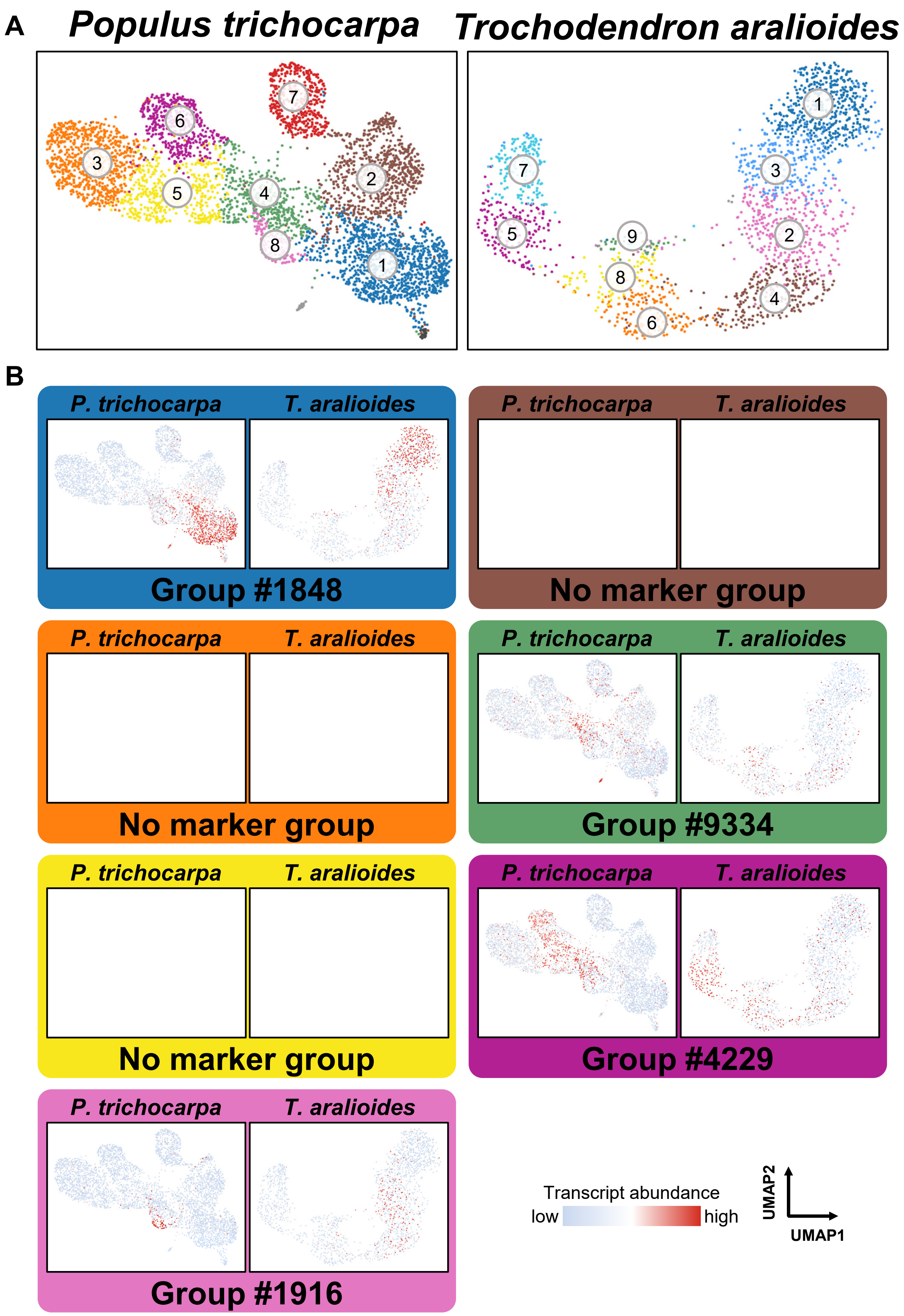
A
Populus trichocarpa
Trochodendron aralioides
B
P. trichocarpa
T. aralioides
Group #1848
No marker group
Group #9334
Group #4229
Group #1916
Transcript abundance
low
high
UMAP2
UMAP1

Fig. S25. Representative marker orthologous groups exclusively expressed in the different cell clusters both in *P. trichocarpa* and *T. aralioides*. (A) The cell cluster plots obtained through unsupervised K-means clustering and UMAP are based on the SDX scRNA-seq results of *P. trichocarpa* and *T. aralioides*, respectively. (B) The cluster-exclusive distributions of each marker orthologous group are represented by Group #1848 for vessel element/late fusiform precursor (blue), Group #9334 for fusiform early precursor (green), Group #4229 for fusiform organizer (purple) and Group #1916 for ray parenchyma cell (pink). No marker orthologous groups are observed in fusiform intermediate precursor (brown), ray organizer (orange) and ray precursor (yellow).

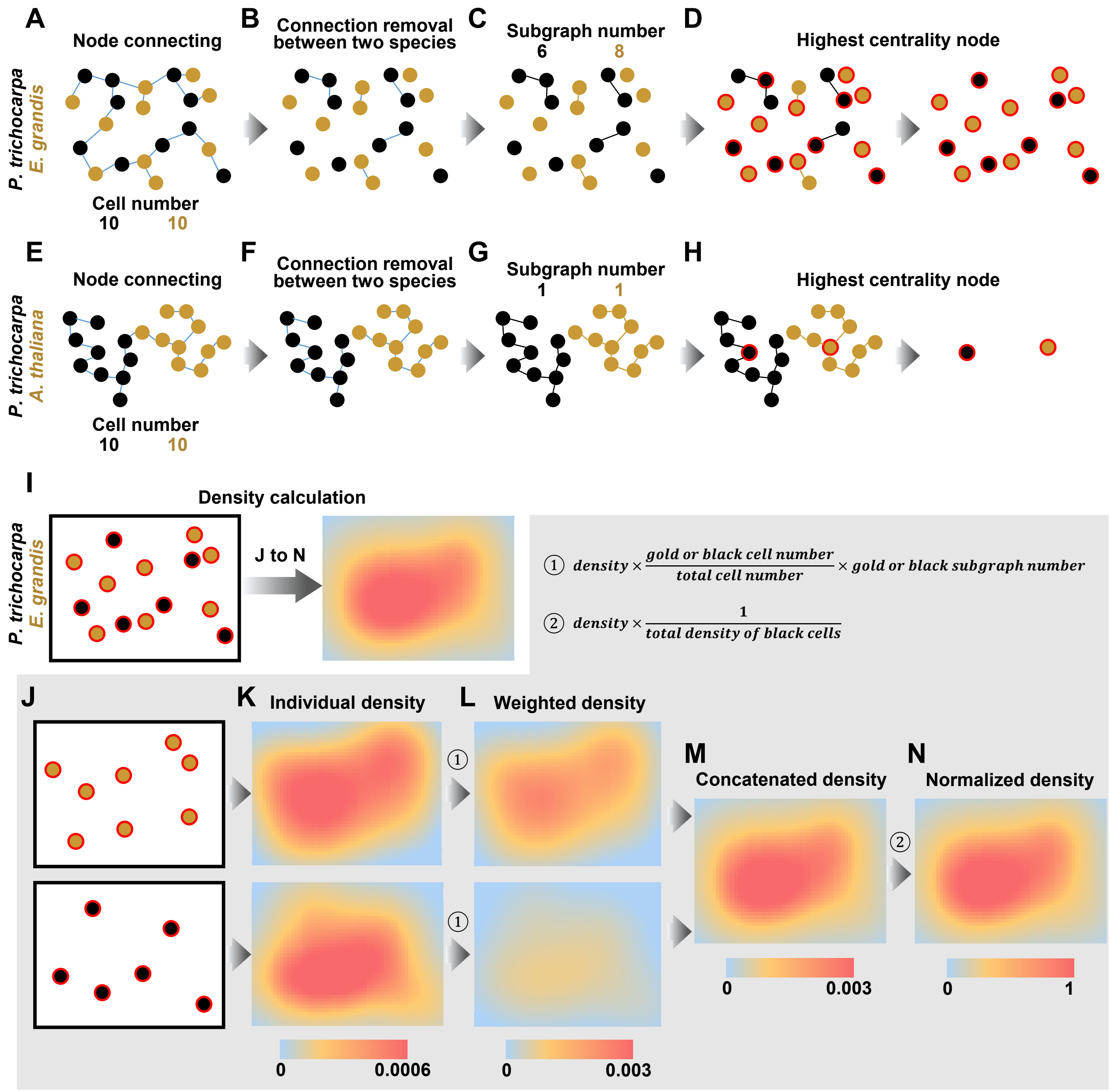

Fig. S26. Analysis of distribution overlap of cells between two species or samples. (A–H) Two schematic examples are given: *P. trichocarpa* vs. *E. grandis* and *P. trichocarpa* vs. *A. thaliana*. (A and E) All the nodes (cells) are connected with the total length of lines as small as possible. (B and F) The lines between the nodes from different species are removed, generating subgraphs. (C and G) The numbers of subgraphs are counted. (D and H) One of the node with the highest centrality in each subgraph is extracted as the center node (circled in red). (I) After removing the other nodes, the density of subgraph centers is calculated with the procedure from (J) to (N). The schematic example of *P. trichocarpa* vs. *E. grandis* is used for explanation. (J) The plots of center nodes are separated by individual species. (K) Density of center nodes is calculated for each plot as individual density. (L) Individual densities are weighted by the proportion of cells and the subgraph numbers to obtain weight densities. (M) Weighted densities are added together into a concatenated density. (N) A concatenated density is normalized by the total density of black cells.

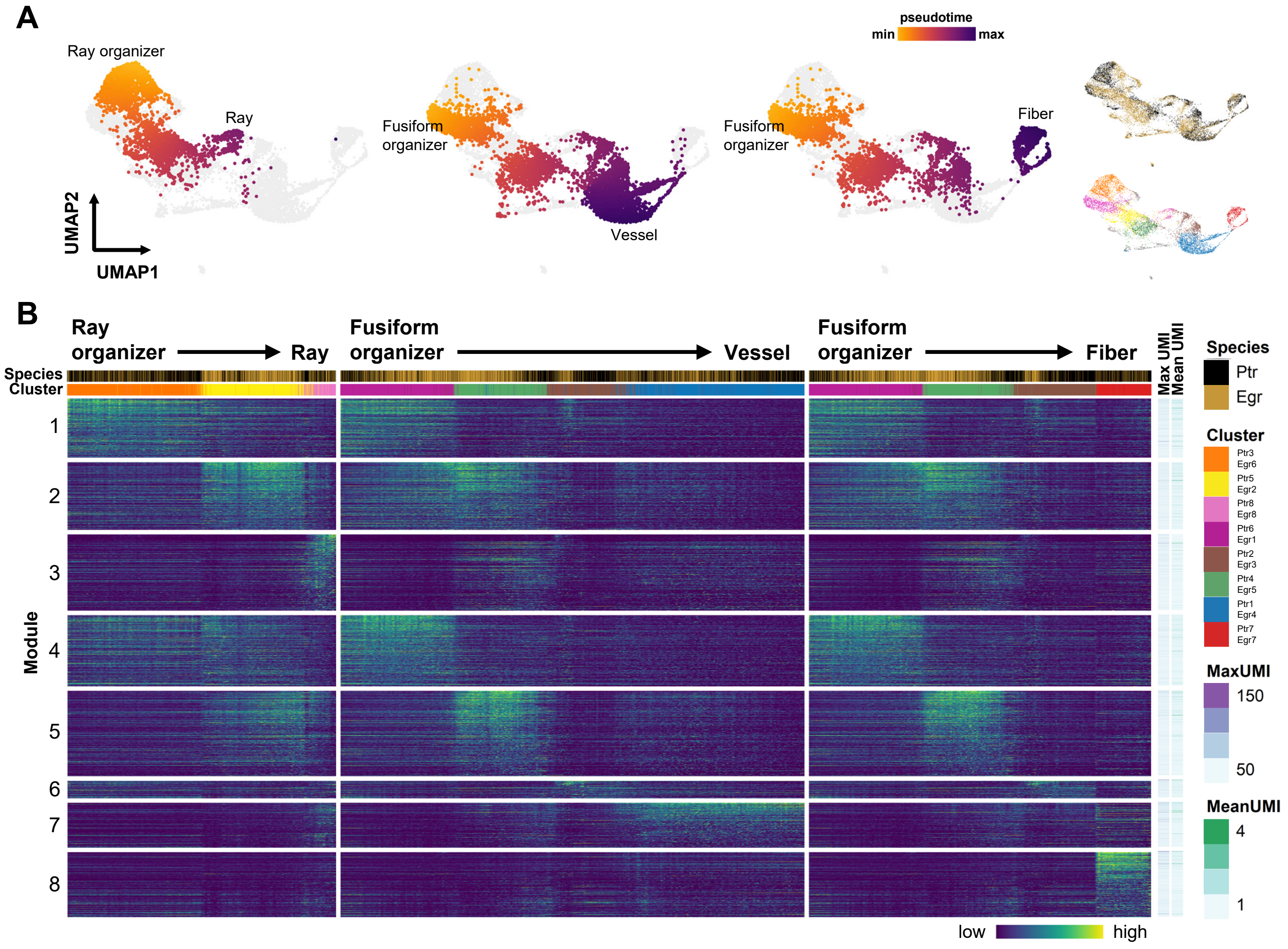

Fig. S27. Pseudotime analysis to reveal the temporal expression pattern of each cell lineage using two-species clustering of *P. trichocarpa* and *E. grandis*. (A) Ray parenchyma, vessel element and libriform fiber lineages are from ray organizer or fusiform organizer. (B) Representative orthologous groups for each cell cluster can be categorized into eight modules (1–8). Along with the lineages two color bars represent as cells from two species (*P. trichocarpa* as black and *E. grandis* as gold) and their corresponding clusters. Maximum and mean UMI counts of each orthologous group are shown.

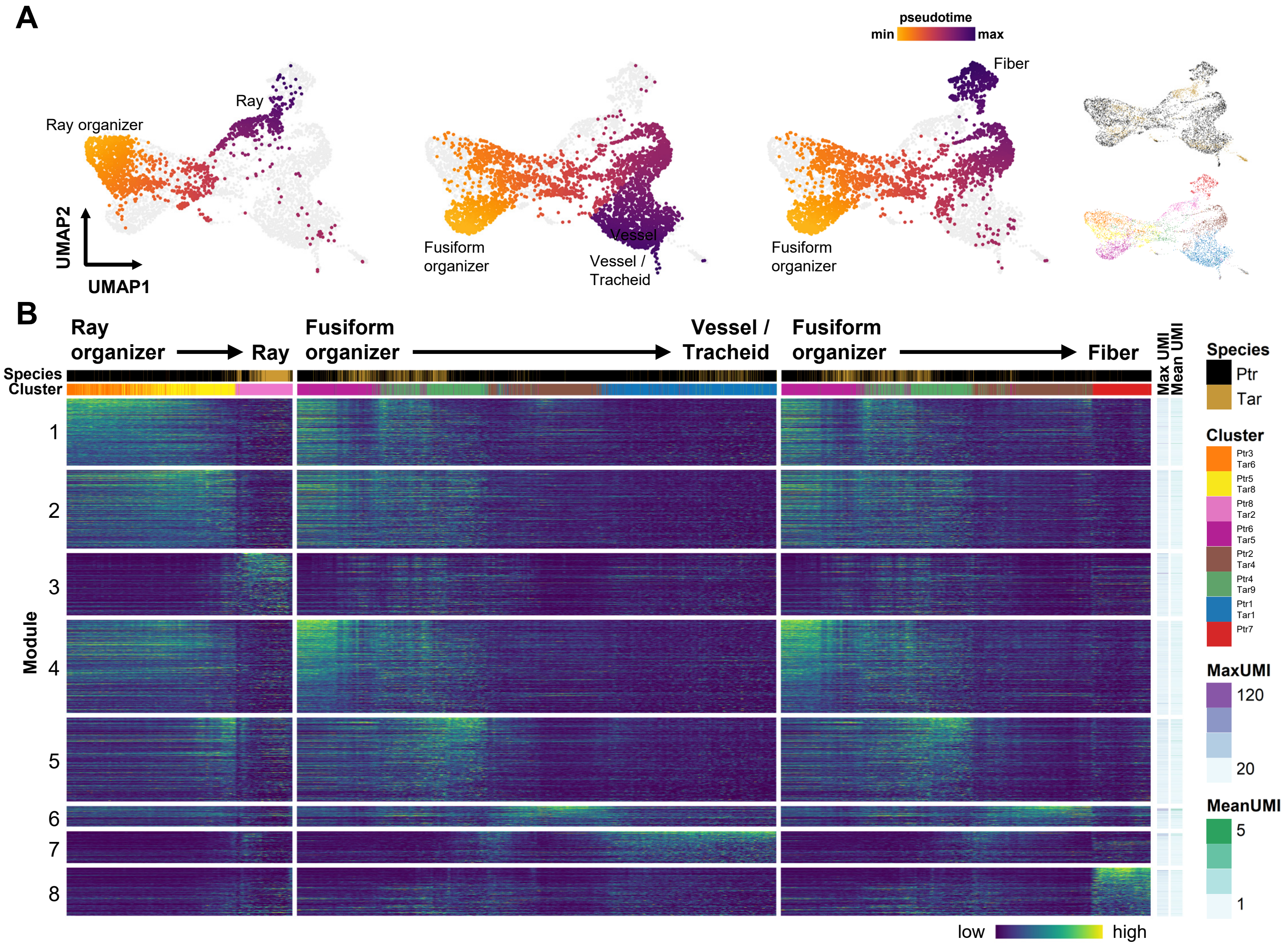

Fig. S28. Pseudotime analysis to reveal the temporal expression pattern of each cell lineage using two-species clustering of *P. trichocarpa* and *T. aralioides*. (A) Ray parenchyma, vessel element/tracheid and libriform fiber lineages from ray organizer or fusiform organizer are shown. (B) Representative orthologous groups for each cell cluster can be categorized into eight modules (1–8). Along with the lineages two color bars represent as cells from two species (*P. trichocarpa* as black and *T. aralioides* as gold) and their corresponding clusters. Only *P. trichocarpa* exhibits cells in libriform fiber cluster (Ptr7) of the libriform fiber lineage. Maximum and mean UMI counts of each orthologous group are shown.

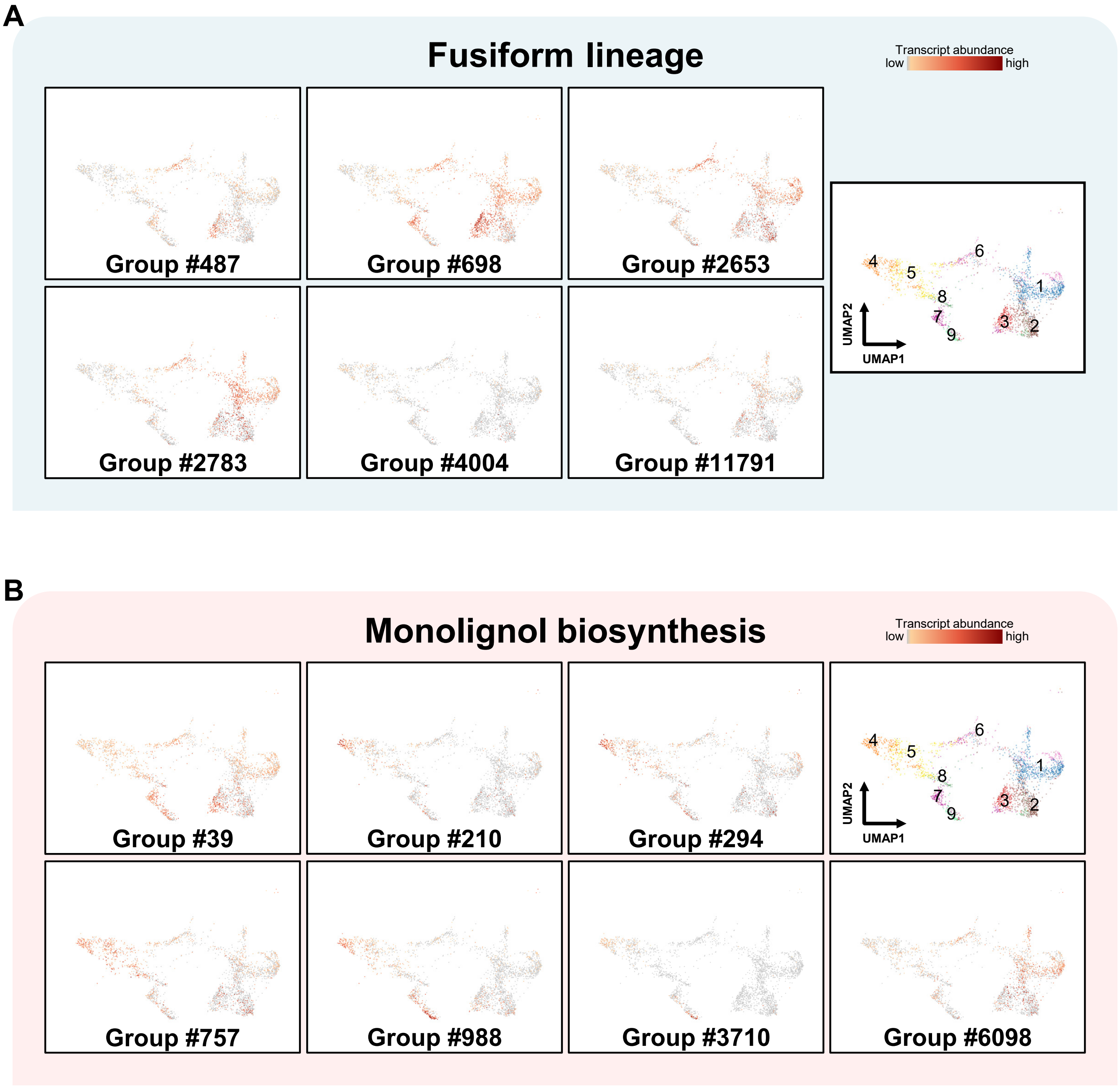

Fig. S29. The *L. chinense* homologous genes of the fusiform marker genes and monolignol biosynthesis genes from *P. trichocarpa*. (A and B) The transcript abundance of the *L. chinense* homologous genes of the fusiform marker genes (A) and monolignol biosynthesis genes (B) from *P. trichocarpa* are shown on the UMAP plots of two-species analyses (Fig. 3C(iv)).

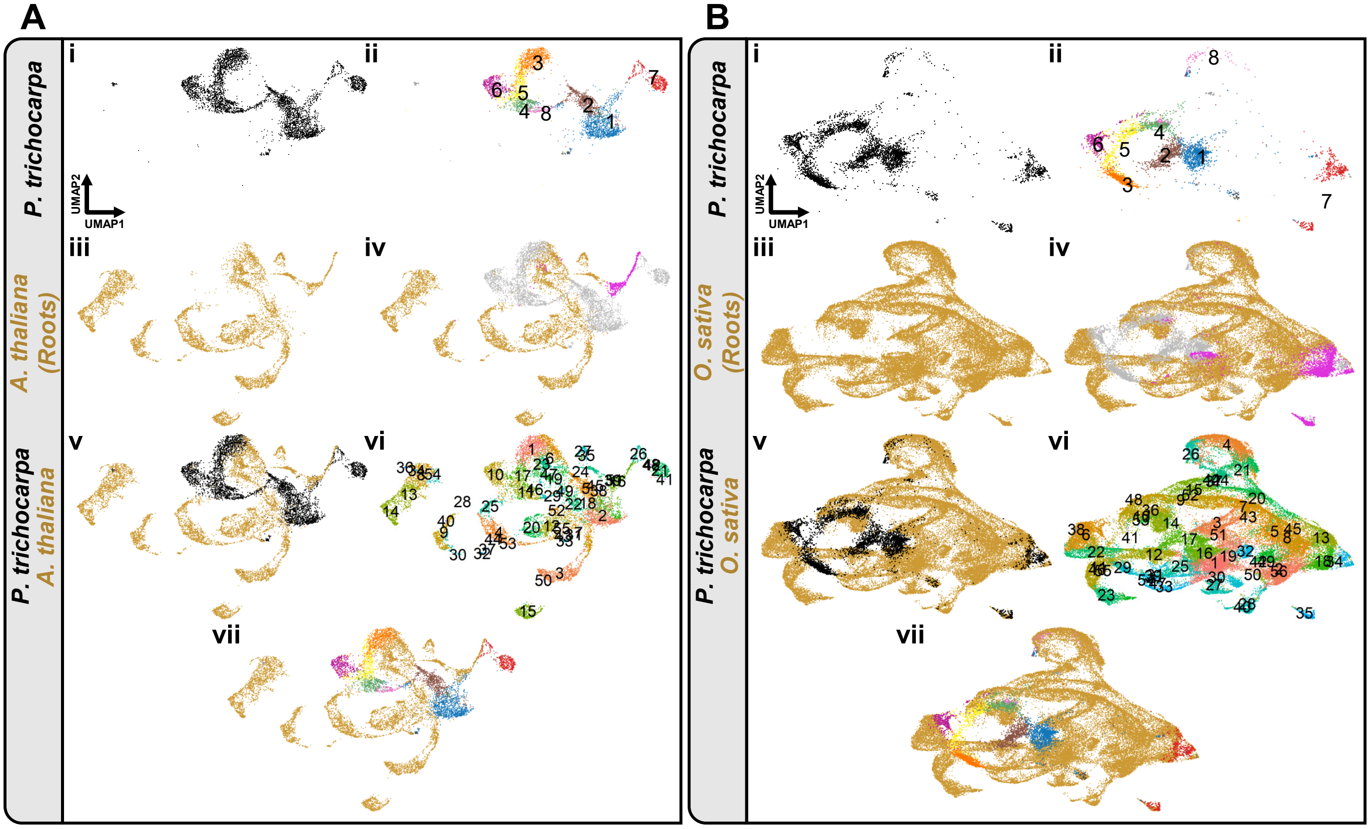

Fig. S30. Two-species clustering and visualization of scRNA-seq data between *P. trichocarpa* and *A. thaliana* or *O. sativa*. (A and B) Two-species clustering of SDX cells in *P. trichocarpa* and root cells in *A. thaliana* (A) or *O. sativa* (B). Single-species unsupervised K-means clustering (i–iii). Two-species graph-based cell clustering using orthologous genes (iv–vii). In (i) and (v), black dots are SDX cells from *P. trichocarpa*. In (iii), (iv), (v), and (vii), gold dots are cells from *A. thaliana* or *O. sativa*. In (iv), grey dots represent the SDX cells from *P. trichocarpa*, and the xylem cells identified in previous *Arabidopsis* or rice studies are in magenta. In (ii) and (vii), the colors of cell clusters are based on the single-species cell clustering results. The cell clusters in two-species clustering (vi).

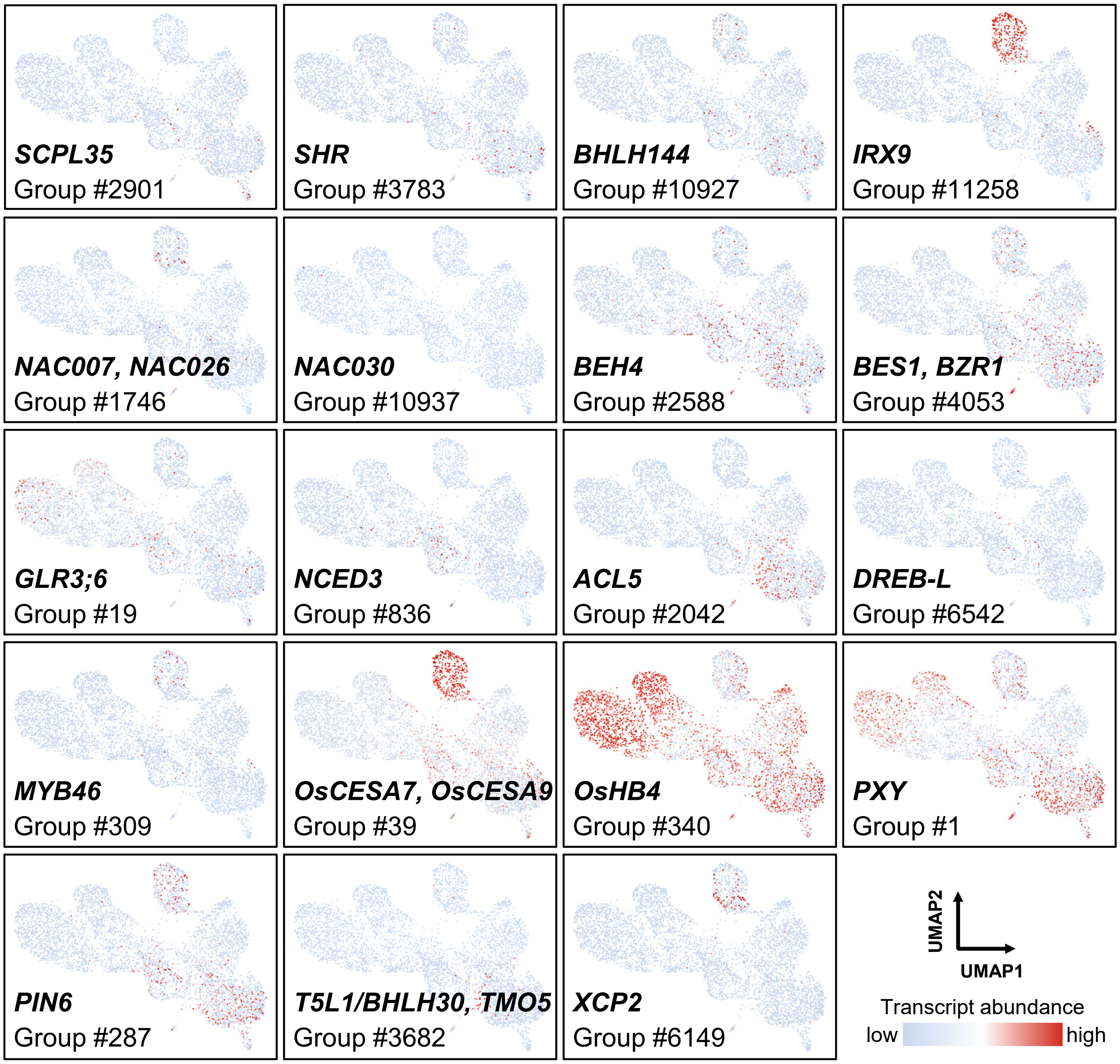

Fig. S31. Transcript abundance of previously identified xylem marker genes. The transcript abundance of the *P. trichocarpa* homologs of *A. thaliana* and *O. sativa* genes are shown on the UMAP plots of scRNA-seq results.

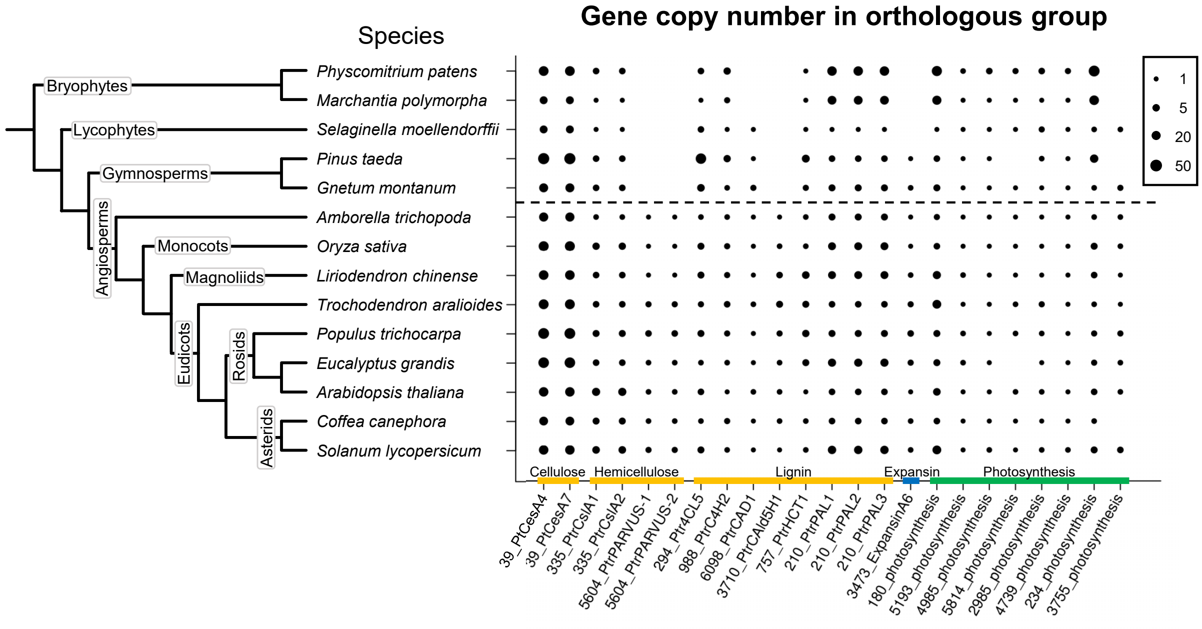

Fig. S32. Gene copy numbers in the orthologous groups of xylem development related genes across 14 plant species. The different sizes of dots represent gene copy numbers in each orthologous group of each species. The names of each orthologous group are represented by their group numbers (see Additional file 10) followed by their common names or the gene functions. The dashed line separates angiosperms from gymnosperms and seedless plants.

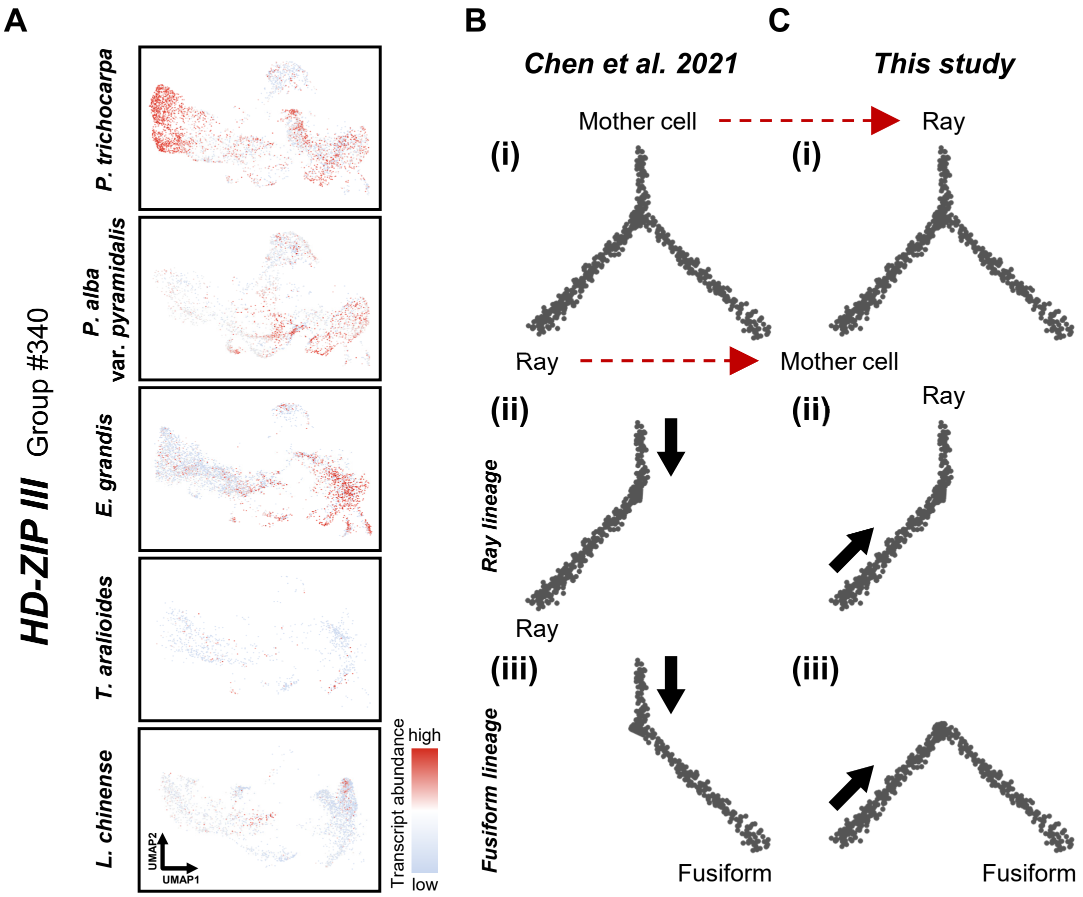

Fig. S33. The cell-type annotation using *HD-ZIP III* as marker genes in different species. (A) Transcript abundance of *HD-ZIP III* is shown on the UMAP plots of five-species analyses (Additional file 1: Fig. S37A–E). (B–C) Cell-type annotations and developmental lineages derived from Chen et al. [44] (B) and from this study (C).

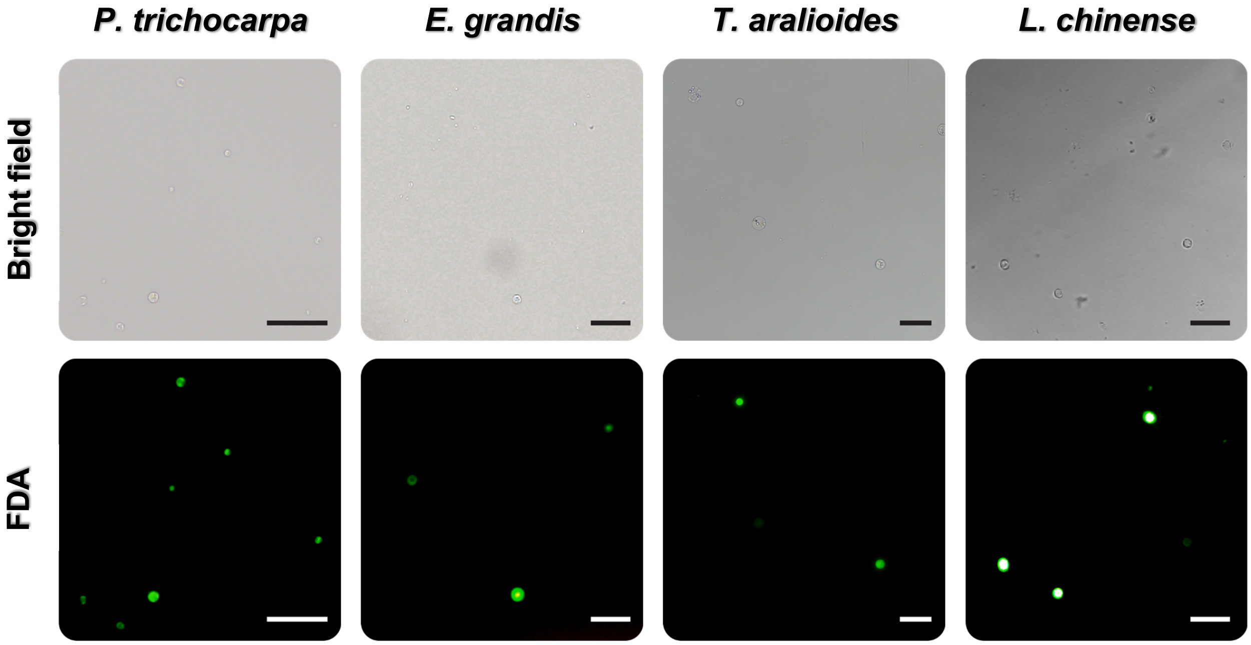

Fig. S34. SDX protoplasts of *P. trichocarpa*, *E. grandis*, *T. aralioides* and *L. chinense*. The SDX protoplasts were observed using fluorescence microscope. Upper panel, the morphology of SDX protoplasts from each species in bright field. Lower panel, the SDX protoplasts with FDA staining. Scale bars are 50 µm.

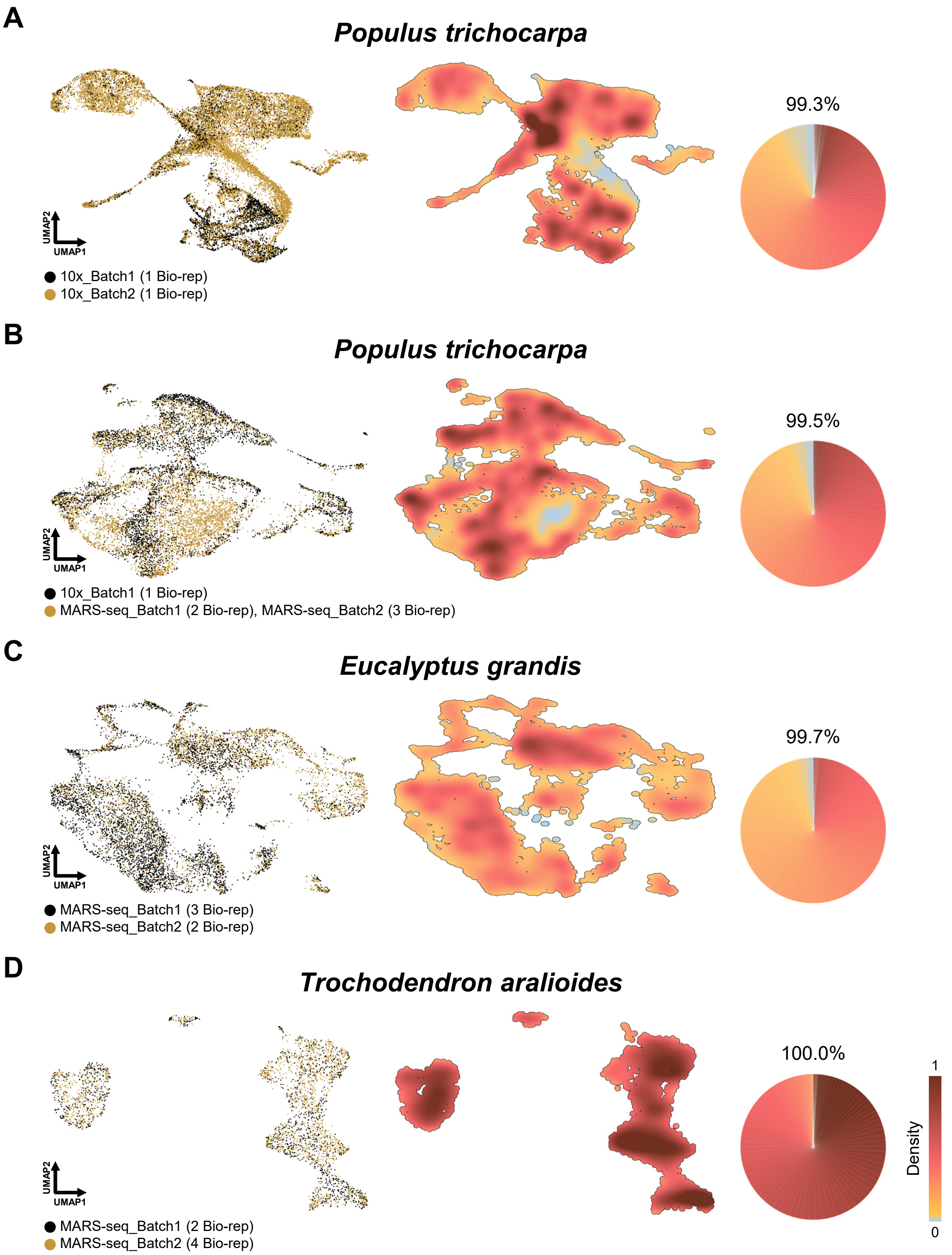

Fig. S35. Reproducibility of 10x scRNA-seq and MARS-seq in *P. trichocarpa*, *E. grandis* and *T. aralioides*. (A–D) The scRNA-seq data integration (left panel), cell density of the clustering results from different batches or platforms (middle panel) are two-dimensional projected on the UMAP plots for *P. trichocarpa* (A and B), *E. grandis* (C) or *T. aralioides* (D). Densities from 0 to 1 are divided into 500 bins with different color shading, with proportions of different densities shown in a pie chart (right panel). The distribution overlap (indicated above the pie chart) is the sum of proportions excluding the lowest bin, where cells from the two batches or platforms barely co-localize. Each batch of each species represents 1 or multiple biological replicates (Bio-rep) (see Methods for the details).

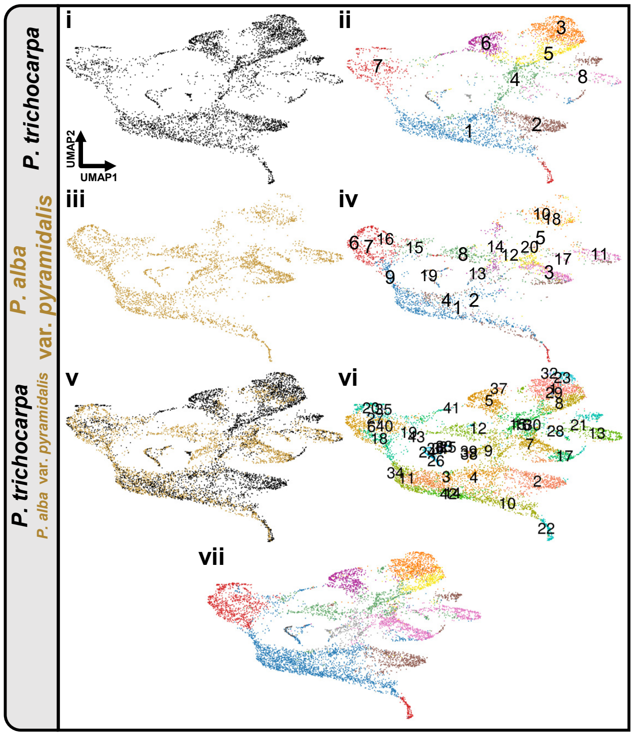

Fig. S36. Two-species clustering and visualization of scRNA-seq data between *P. trichocarpa* and *P. alba* var. *pyramidalis*. Two-species clustering of SDX single cells in *P. trichocarpa* and *P. alba* var. *pyramidalis*. Single-species unsupervised K-means clustering (i–iv). Two-species graph-based cell clustering using orthologous genes (v–vii). In (i), (iii), and (v), black dots are SDX cells from *P. trichocarpa* and gold dots are cells from *P. alba* var. *pyramidalis*. In (ii), the colors of cell clusters in *P. trichocarpa* are based on the single-species cell clustering results. In (iv), the cell cluster colors are assigned using the co-located colors from *P. trichocarpa* cell clusters. The cell clusters in two-species clustering (vi). In (vii), the colors of two-species clustering are derived from that of single-species clustering.

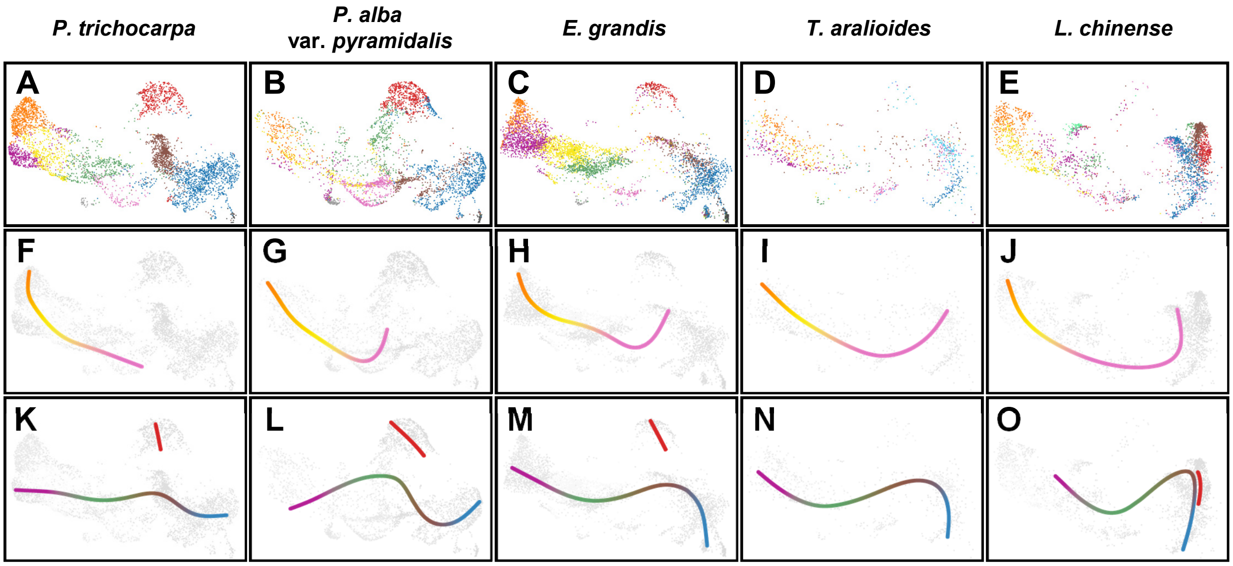

Fig. S37. Cell lineages in SDX development in five woody angiosperms. (A–E) Combined five-species analyses and two-dimensional visualization of SDX scRNA-seq data. Individual cells are colored as in Fig. 1, 2 and Additional file 1: Fig. S36. (F–O) The ray (F–J) and fusiform (K–O) lineages in the five species.

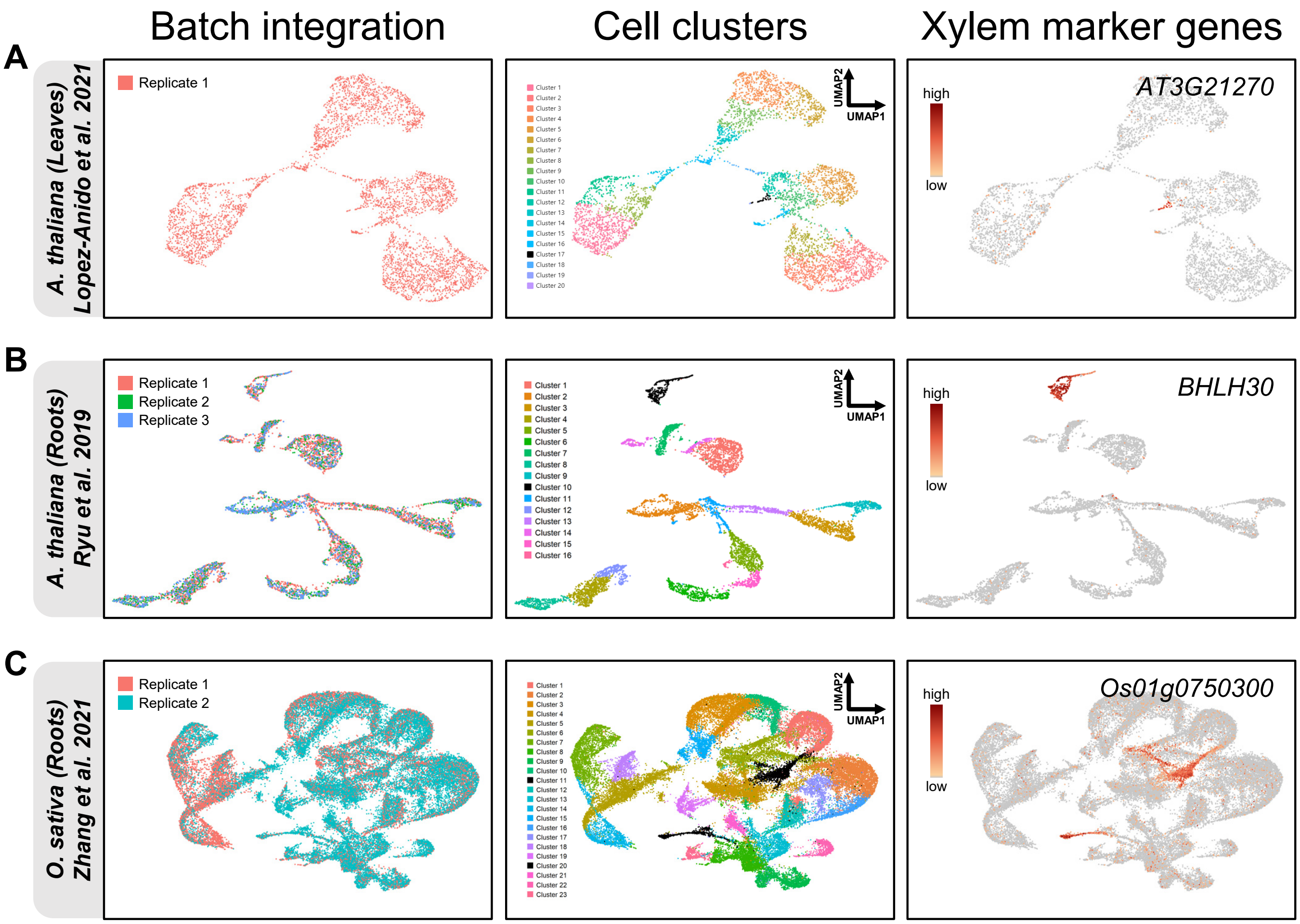

Fig. S38. Xylem cell identification from previous scRNA-seq results. (A–C) Different replicates of the scRNA-seq results from *A. thaliana* roots and *O. sativa* roots are first integrated for cell clustering using Seurat pipeline. Previous identified xylem cells using marker genes reveal that the xylem cells locate at Cluster 17, Cluster 10 and Cluster 11/20 in *A. thaliana* leaves (A), roots (B) and *O. sativa* roots (C), respectively.
