# Supplementary figures and images for: Single-cell transcriptomics unveils xylem cell development and evolution
Source: Genome Biol. 2023 Jan 9;24:3. doi: 10.1186/s13059-022-02845-1 (PMC9830878; doi:10.1186/s13059-022-02845-1)

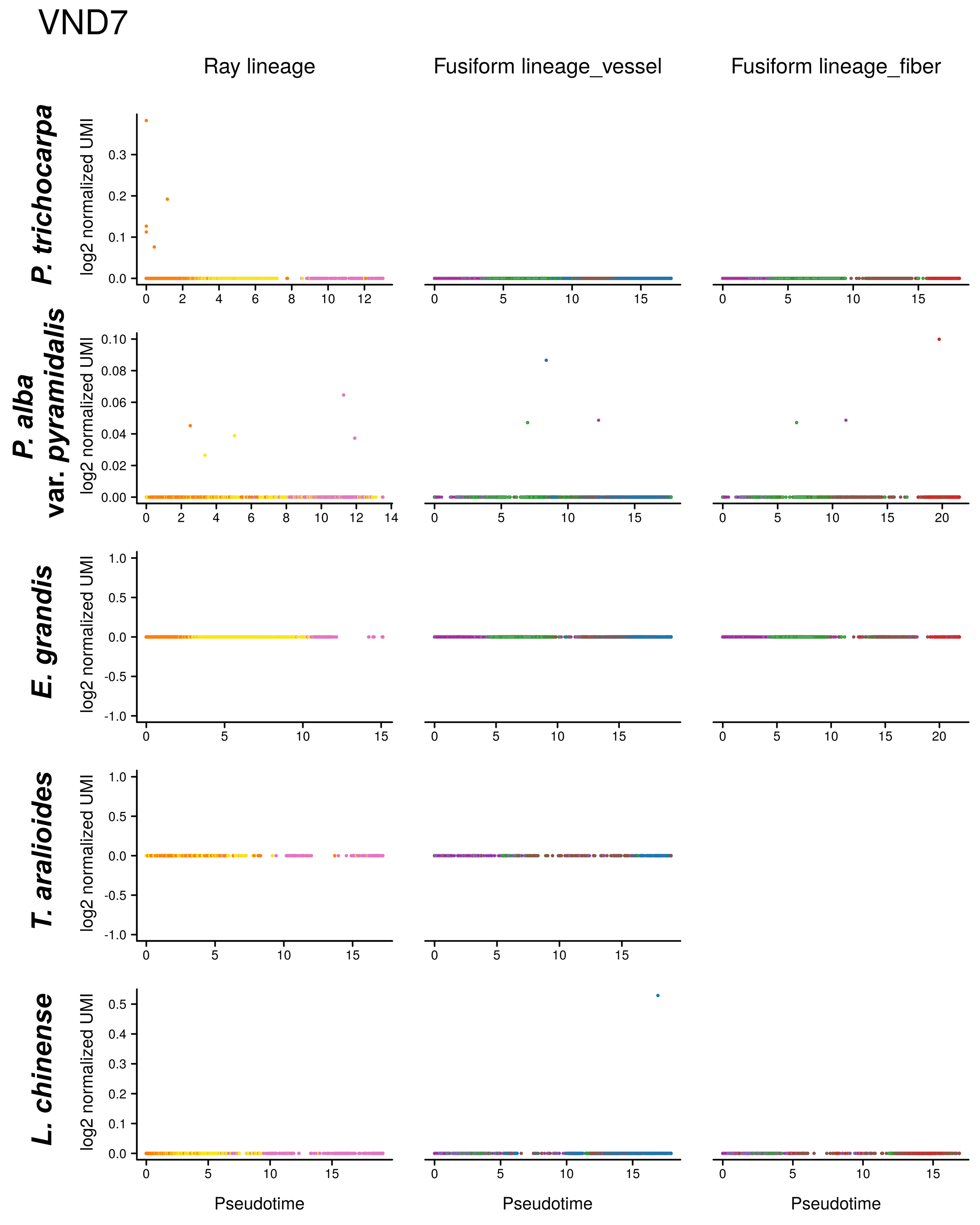

Supplement: Supplementary file 13 — Additional file 13. Expression profiles of the homologous genes of known xylem development related genes in different xylem cell trajectories of P. trichocarpa, P. alba var. pyramidalis, E. grandis, T. aralioides and L. chinense. Empty plots with no coordinates were used to represent the absence of the orthologs in certain species. [file 13059_2022_2845_MOESM13_ESM.zip › Additional file 13/Ortholog_10937_VND7.png]

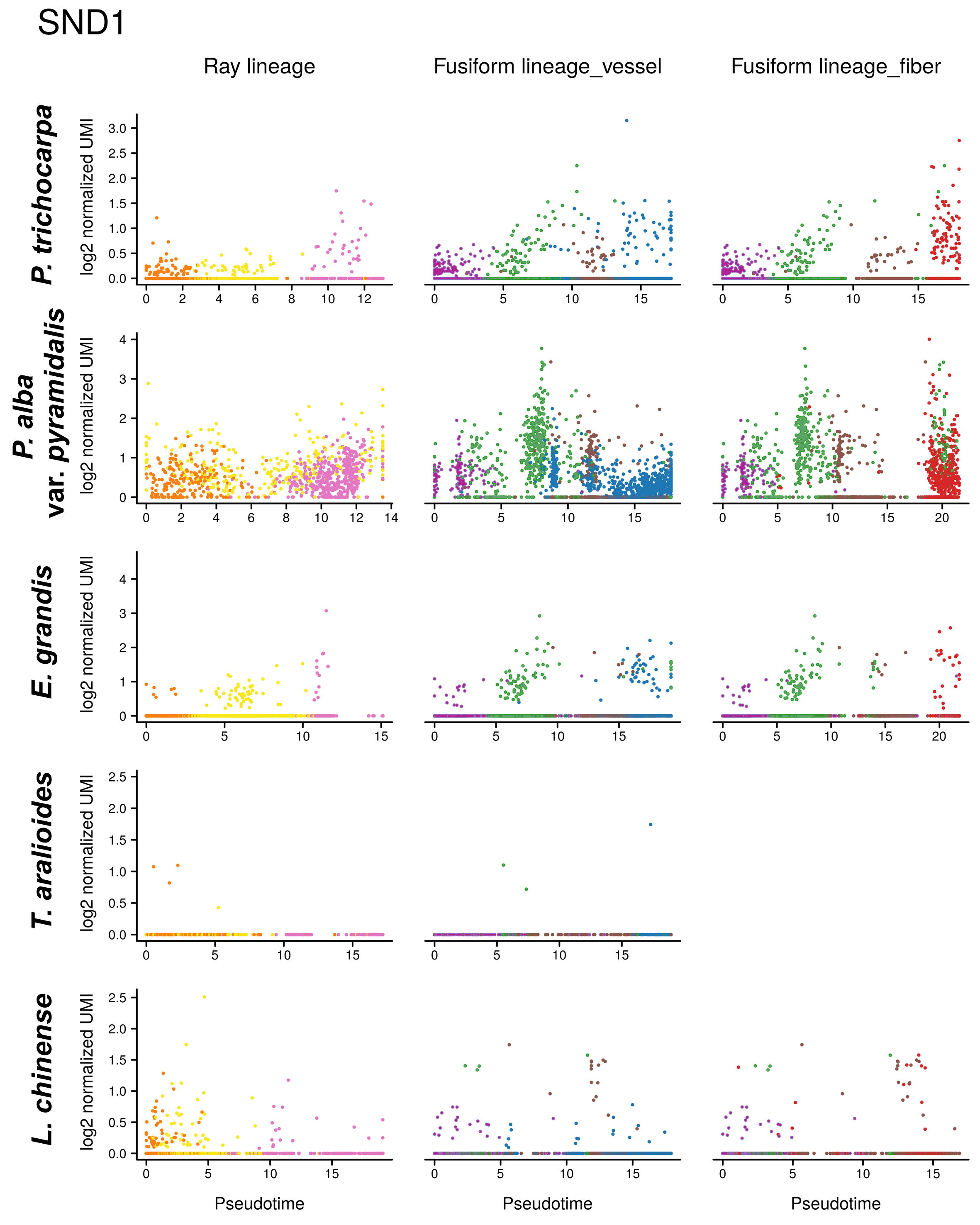

Supplement: Supplementary file 13 — Additional file 13. Expression profiles of the homologous genes of known xylem development related genes in different xylem cell trajectories of P. trichocarpa, P. alba var. pyramidalis, E. grandis, T. aralioides and L. chinense. Empty plots with no coordinates were used to represent the absence of the orthologs in certain species. [file 13059_2022_2845_MOESM13_ESM.zip › Additional file 13/Ortholog_1284_SND1.png]

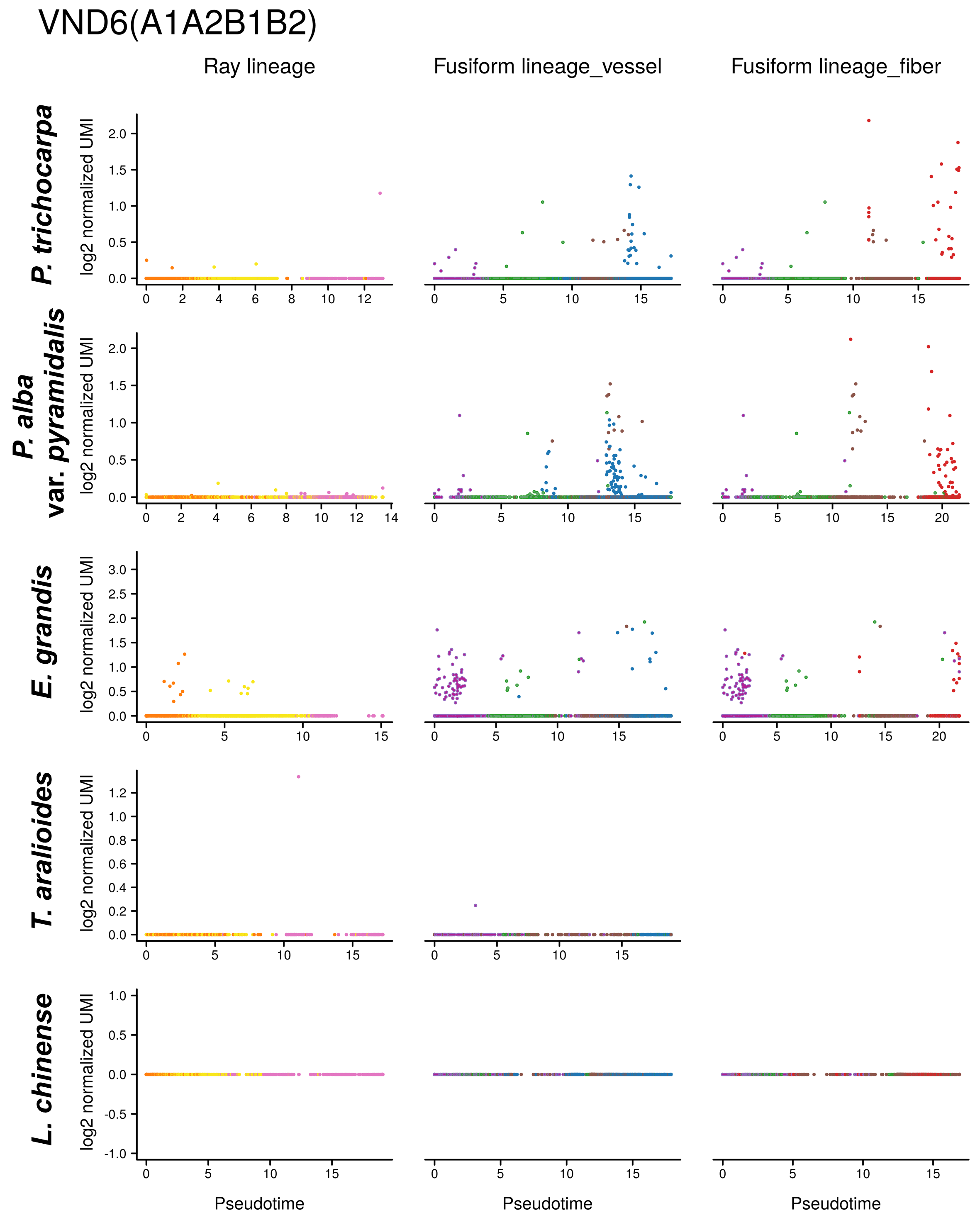

Supplement: Supplementary file 13 — Additional file 13. Expression profiles of the homologous genes of known xylem development related genes in different xylem cell trajectories of P. trichocarpa, P. alba var. pyramidalis, E. grandis, T. aralioides and L. chinense. Empty plots with no coordinates were used to represent the absence of the orthologs in certain species. [file 13059_2022_2845_MOESM13_ESM.zip › Additional file 13/Ortholog_1746_VND6(A1A2B1B2).png]

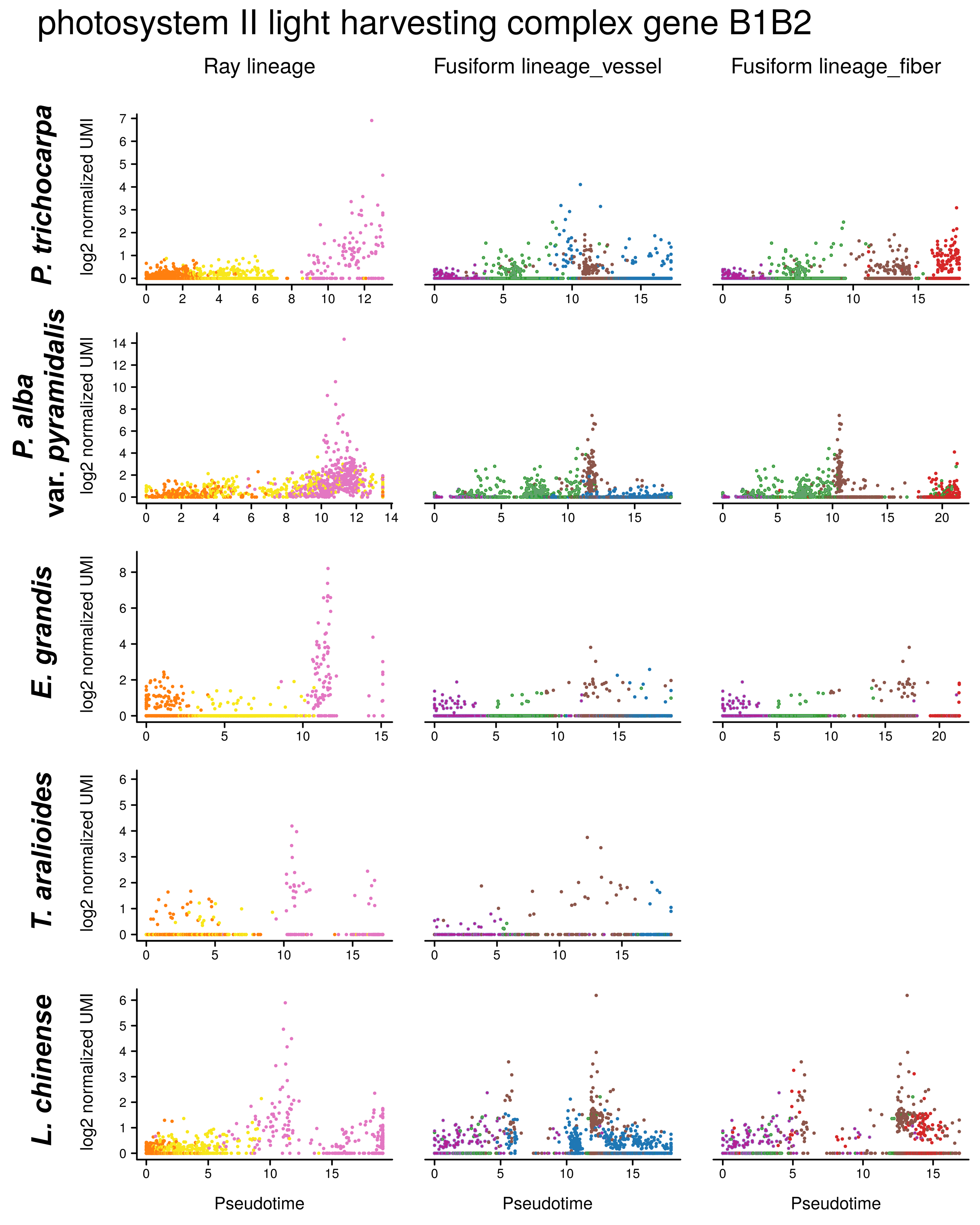

Supplement: Supplementary file 13 — Additional file 13. Expression profiles of the homologous genes of known xylem development related genes in different xylem cell trajectories of P. trichocarpa, P. alba var. pyramidalis, E. grandis, T. aralioides and L. chinense. Empty plots with no coordinates were used to represent the absence of the orthologs in certain species. [file 13059_2022_2845_MOESM13_ESM.zip › Additional file 13/Ortholog_180_photosystem II light harvesting complex gene B1B2.png]

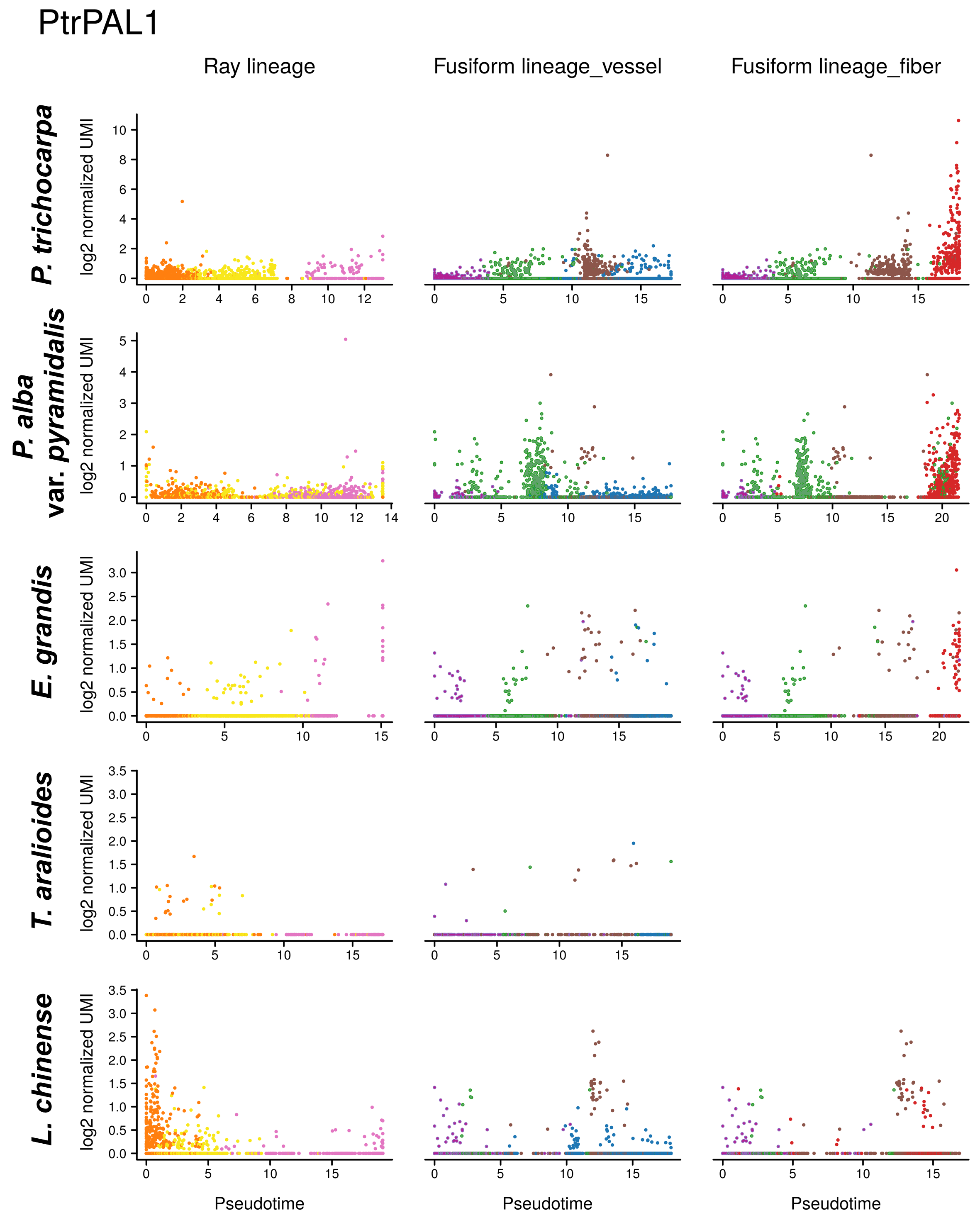

Supplement: Supplementary file 13 — Additional file 13. Expression profiles of the homologous genes of known xylem development related genes in different xylem cell trajectories of P. trichocarpa, P. alba var. pyramidalis, E. grandis, T. aralioides and L. chinense. Empty plots with no coordinates were used to represent the absence of the orthologs in certain species. [file 13059_2022_2845_MOESM13_ESM.zip › Additional file 13/Ortholog_210_PtrPAL1.png]

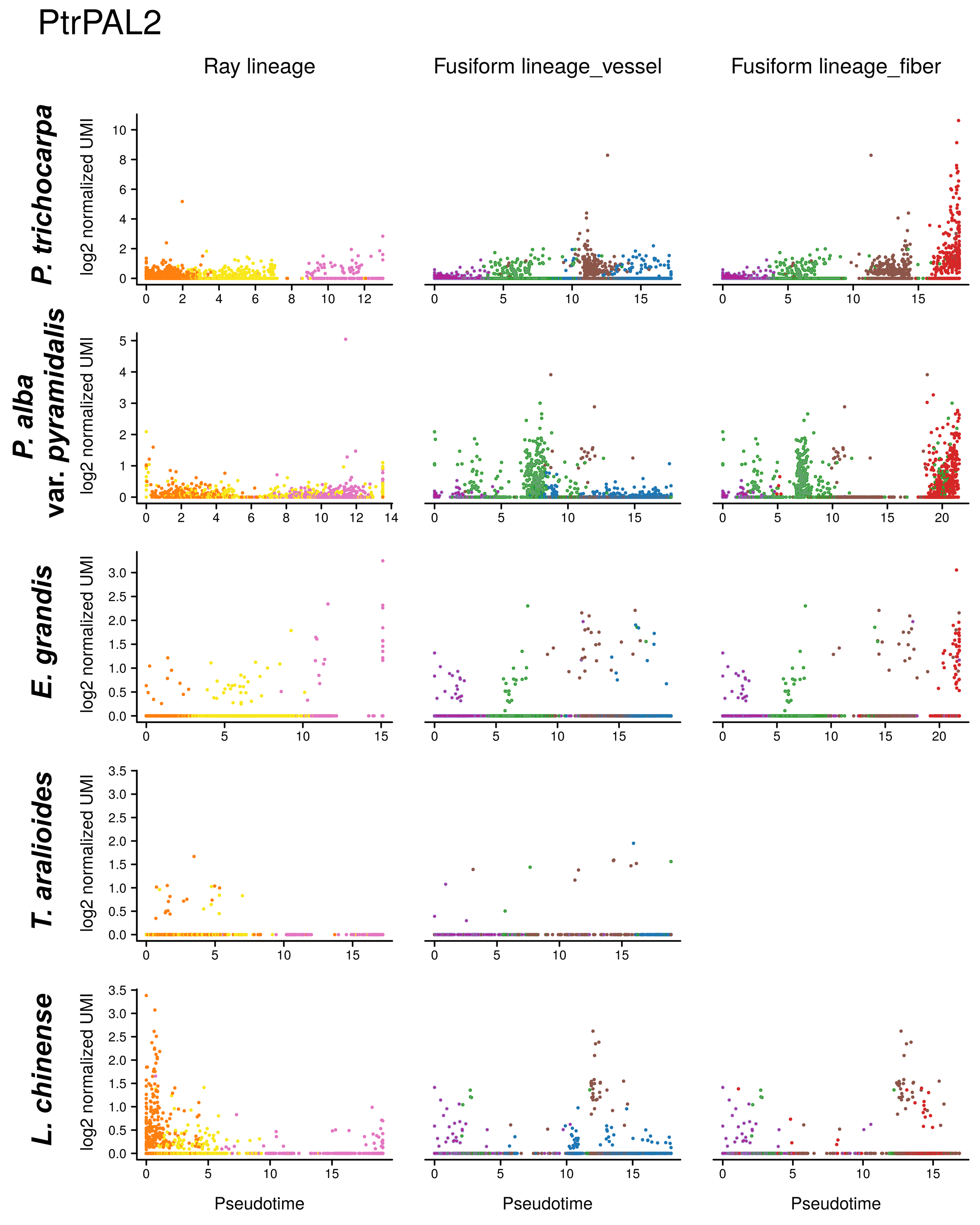

Supplement: Supplementary file 13 — Additional file 13. Expression profiles of the homologous genes of known xylem development related genes in different xylem cell trajectories of P. trichocarpa, P. alba var. pyramidalis, E. grandis, T. aralioides and L. chinense. Empty plots with no coordinates were used to represent the absence of the orthologs in certain species. [file 13059_2022_2845_MOESM13_ESM.zip › Additional file 13/Ortholog_210_PtrPAL2.png]

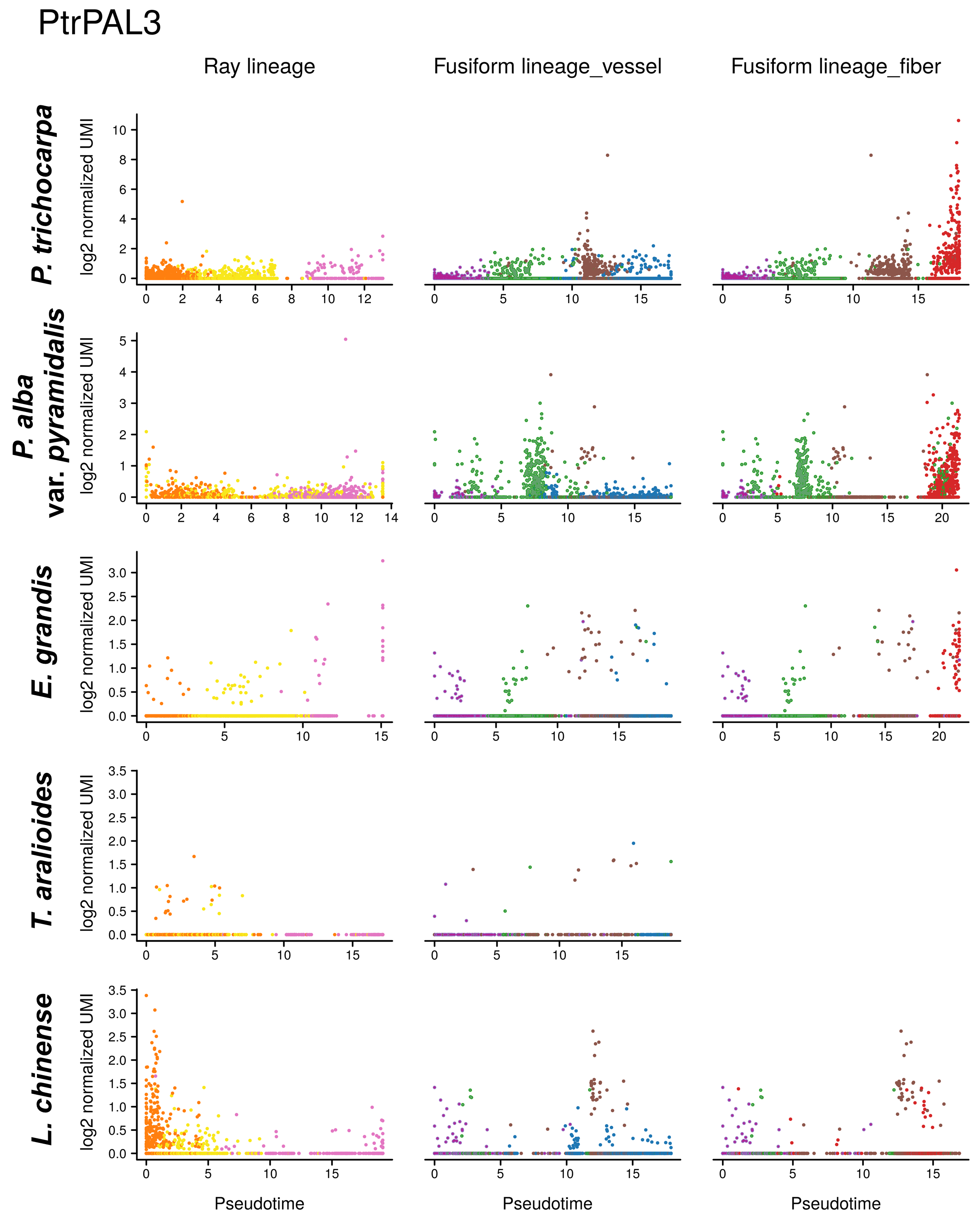

Supplement: Supplementary file 13 — Additional file 13. Expression profiles of the homologous genes of known xylem development related genes in different xylem cell trajectories of P. trichocarpa, P. alba var. pyramidalis, E. grandis, T. aralioides and L. chinense. Empty plots with no coordinates were used to represent the absence of the orthologs in certain species. [file 13059_2022_2845_MOESM13_ESM.zip › Additional file 13/Ortholog_210_PtrPAL3.png]

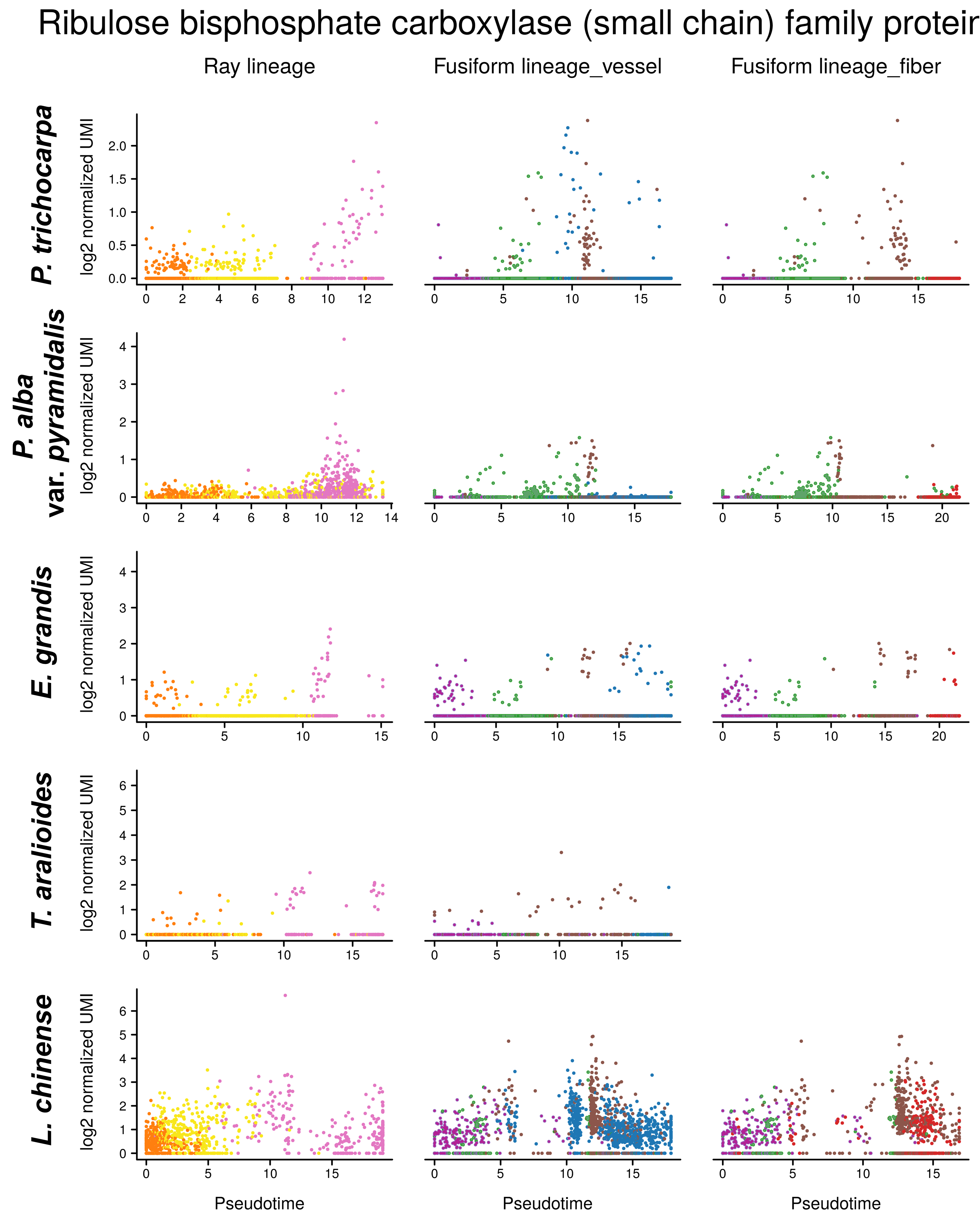

Supplement: Supplementary file 13 — Additional file 13. Expression profiles of the homologous genes of known xylem development related genes in different xylem cell trajectories of P. trichocarpa, P. alba var. pyramidalis, E. grandis, T. aralioides and L. chinense. Empty plots with no coordinates were used to represent the absence of the orthologs in certain species. [file 13059_2022_2845_MOESM13_ESM.zip › Additional file 13/Ortholog_234_Ribulose bisphosphate carboxylase (small chain) family protein.png]

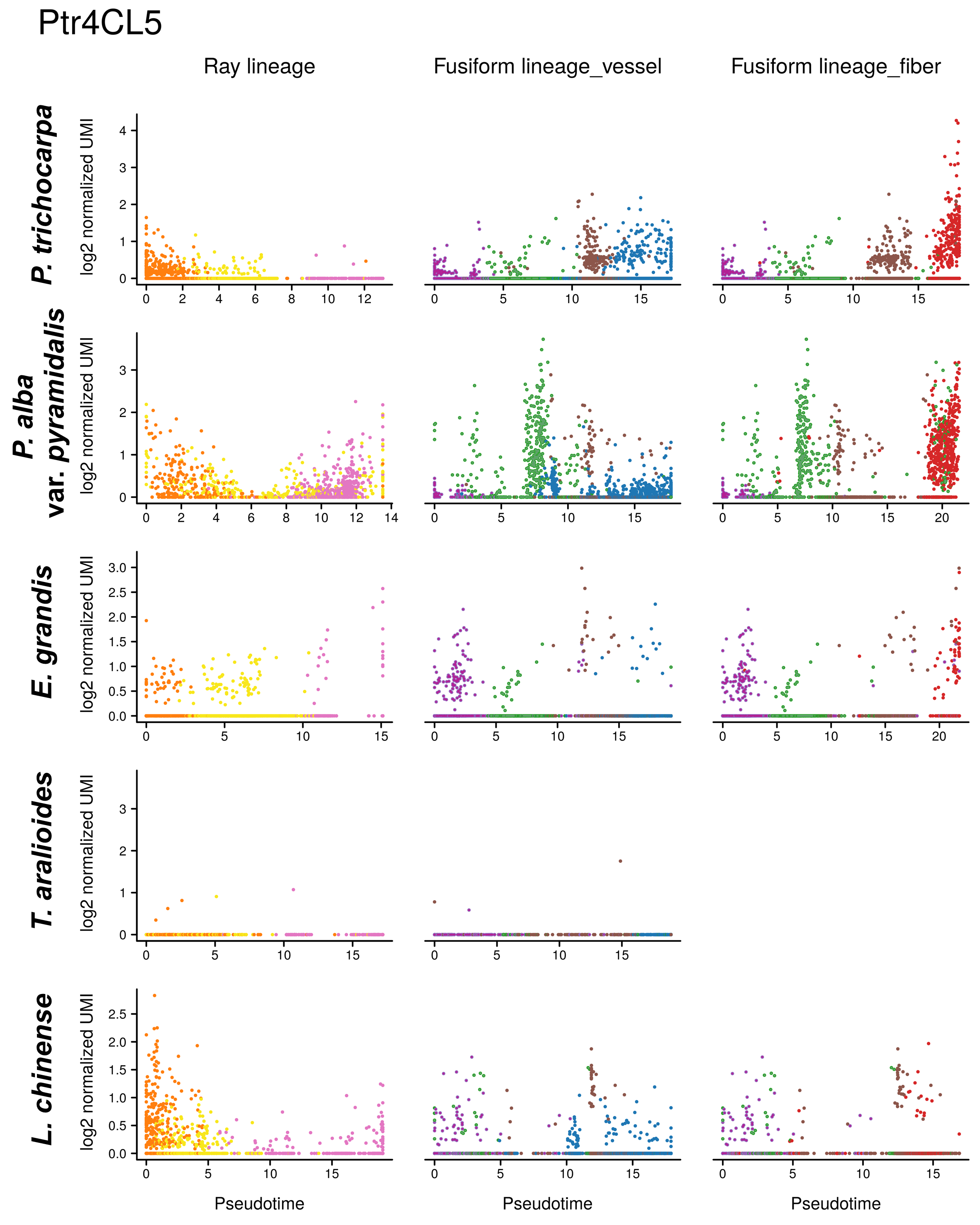

Supplement: Supplementary file 13 — Additional file 13. Expression profiles of the homologous genes of known xylem development related genes in different xylem cell trajectories of P. trichocarpa, P. alba var. pyramidalis, E. grandis, T. aralioides and L. chinense. Empty plots with no coordinates were used to represent the absence of the orthologs in certain species. [file 13059_2022_2845_MOESM13_ESM.zip › Additional file 13/Ortholog_294_Ptr4CL5.png]

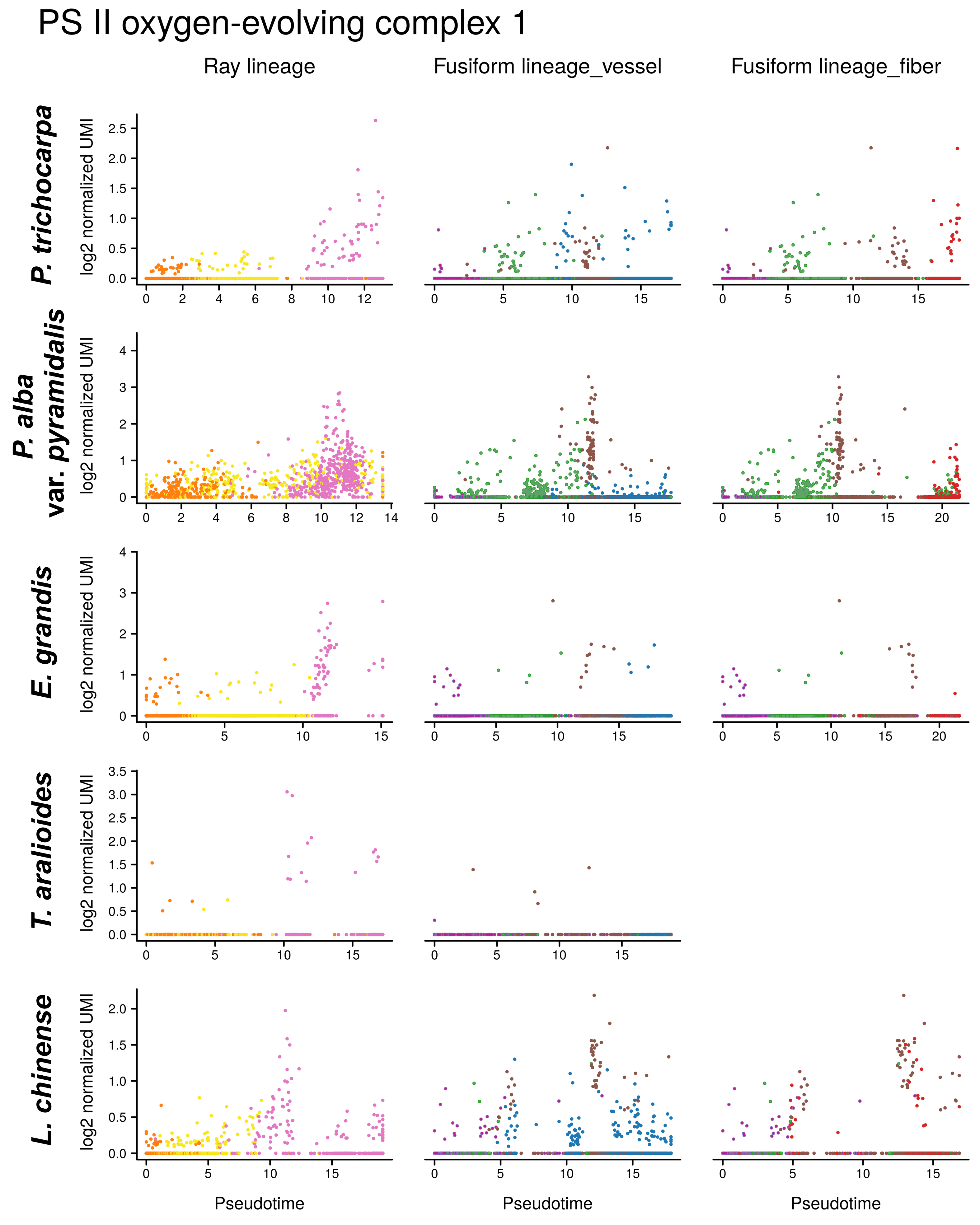

Supplement: Supplementary file 13 — Additional file 13. Expression profiles of the homologous genes of known xylem development related genes in different xylem cell trajectories of P. trichocarpa, P. alba var. pyramidalis, E. grandis, T. aralioides and L. chinense. Empty plots with no coordinates were used to represent the absence of the orthologs in certain species. [file 13059_2022_2845_MOESM13_ESM.zip › Additional file 13/Ortholog_2985_PS II oxygen-evolving complex 1.png]

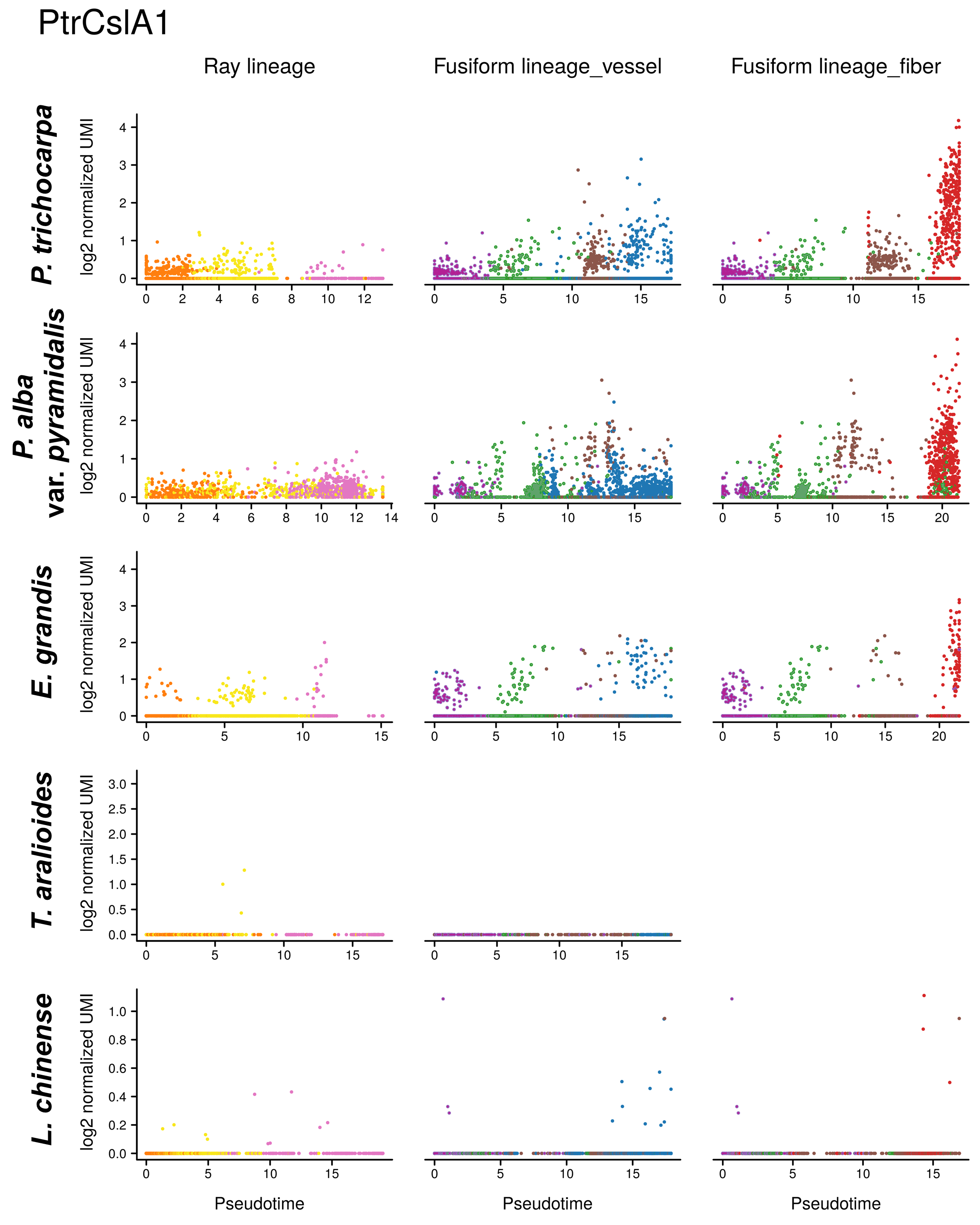

Supplement: Supplementary file 13 — Additional file 13. Expression profiles of the homologous genes of known xylem development related genes in different xylem cell trajectories of P. trichocarpa, P. alba var. pyramidalis, E. grandis, T. aralioides and L. chinense. Empty plots with no coordinates were used to represent the absence of the orthologs in certain species. [file 13059_2022_2845_MOESM13_ESM.zip › Additional file 13/Ortholog_335_PtrCslA1.png]

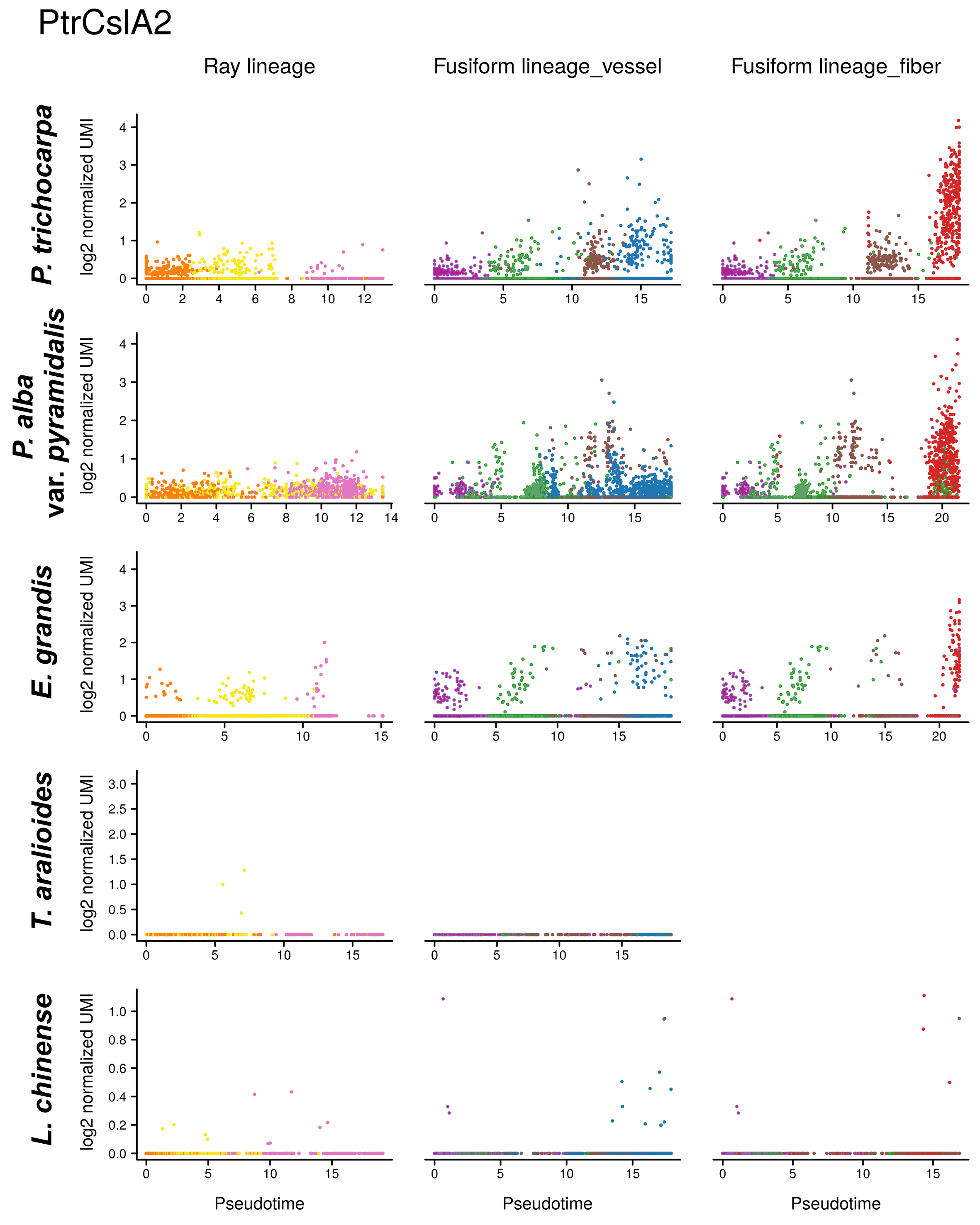

Supplement: Supplementary file 13 — Additional file 13. Expression profiles of the homologous genes of known xylem development related genes in different xylem cell trajectories of P. trichocarpa, P. alba var. pyramidalis, E. grandis, T. aralioides and L. chinense. Empty plots with no coordinates were used to represent the absence of the orthologs in certain species. [file 13059_2022_2845_MOESM13_ESM.zip › Additional file 13/Ortholog_335_PtrCslA2.png]

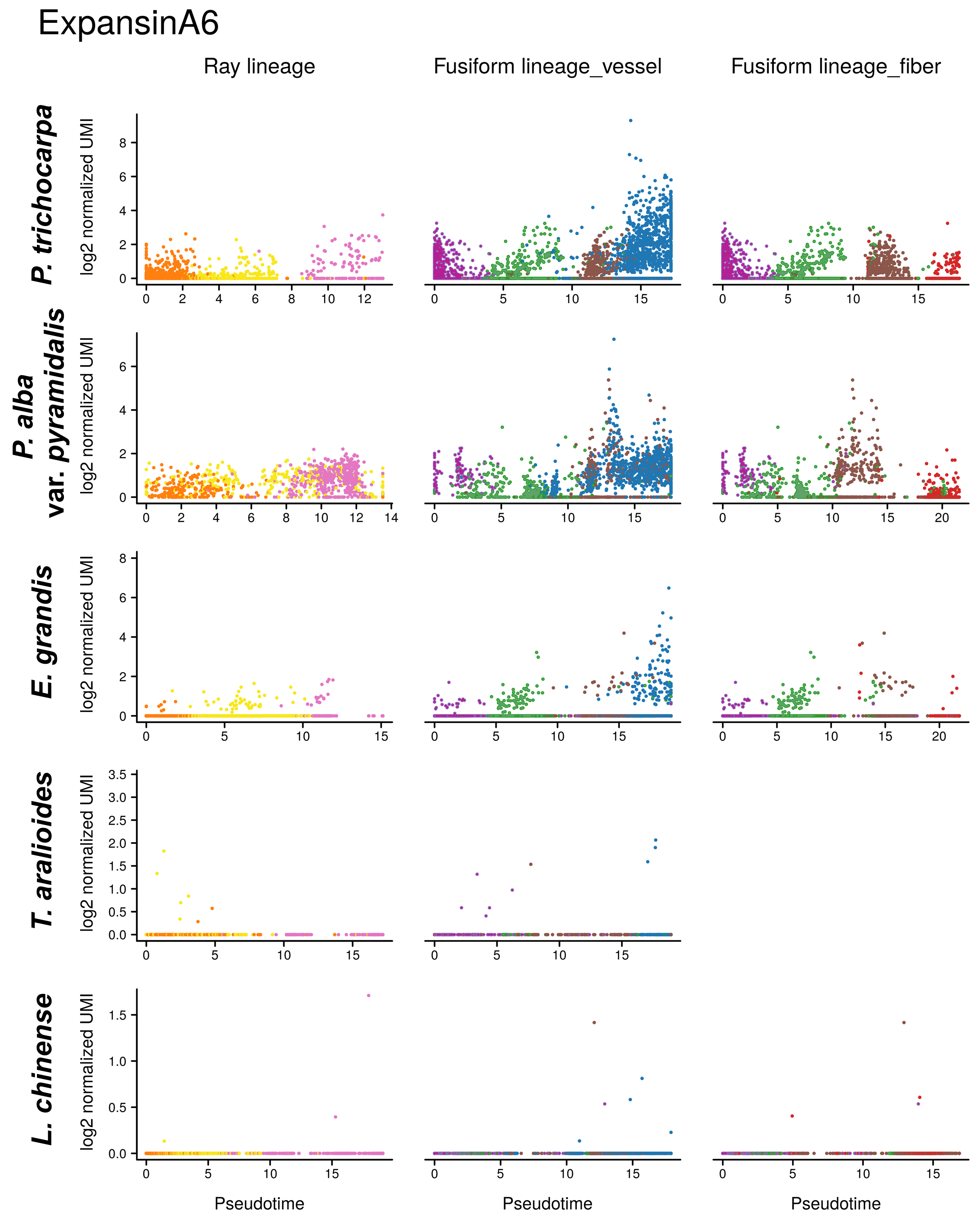

Supplement: Supplementary file 13 — Additional file 13. Expression profiles of the homologous genes of known xylem development related genes in different xylem cell trajectories of P. trichocarpa, P. alba var. pyramidalis, E. grandis, T. aralioides and L. chinense. Empty plots with no coordinates were used to represent the absence of the orthologs in certain species. [file 13059_2022_2845_MOESM13_ESM.zip › Additional file 13/Ortholog_3473_ExpansinA6.png]

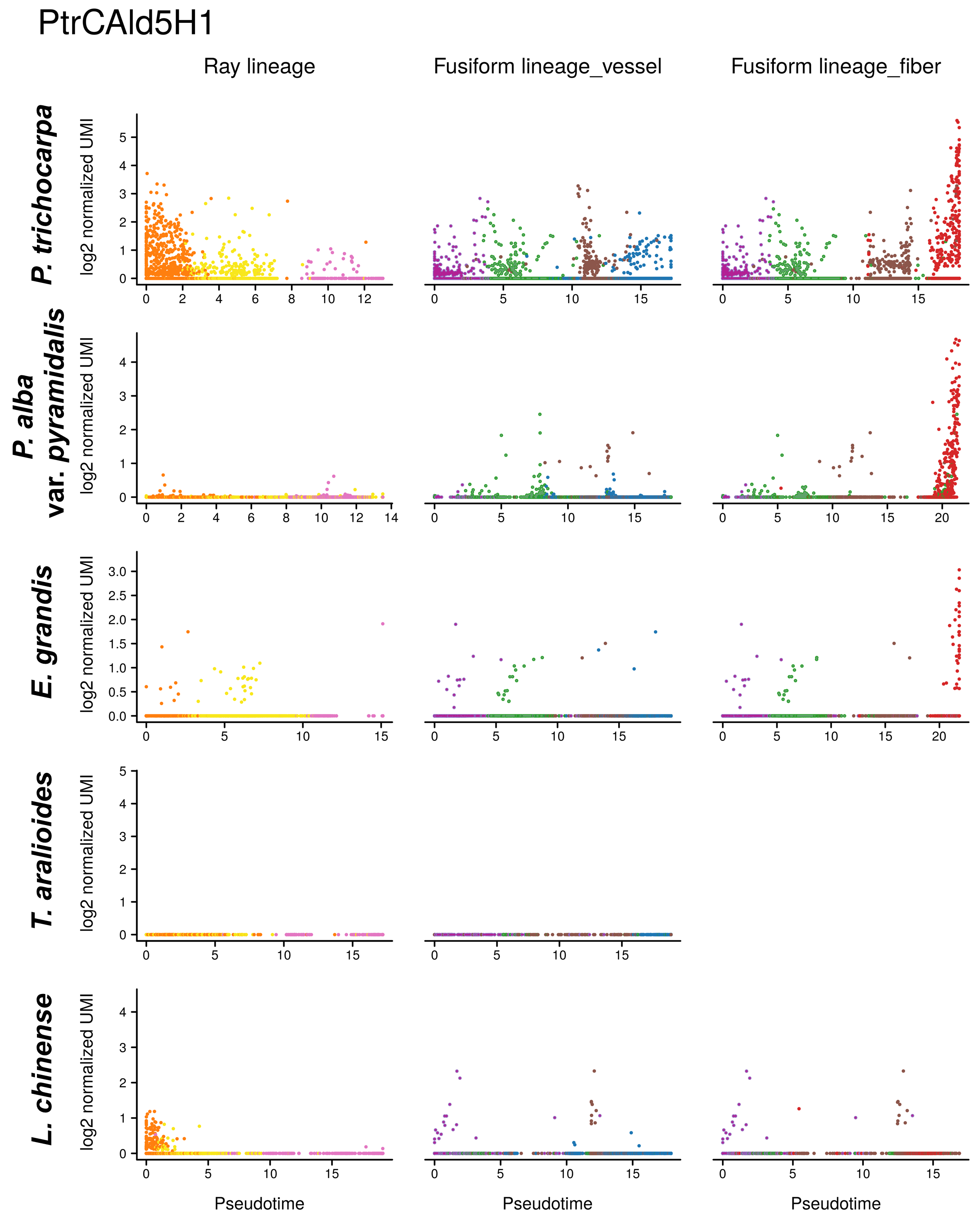

Supplement: Supplementary file 13 — Additional file 13. Expression profiles of the homologous genes of known xylem development related genes in different xylem cell trajectories of P. trichocarpa, P. alba var. pyramidalis, E. grandis, T. aralioides and L. chinense. Empty plots with no coordinates were used to represent the absence of the orthologs in certain species. [file 13059_2022_2845_MOESM13_ESM.zip › Additional file 13/Ortholog_3710_PtrCAld5H1.png]

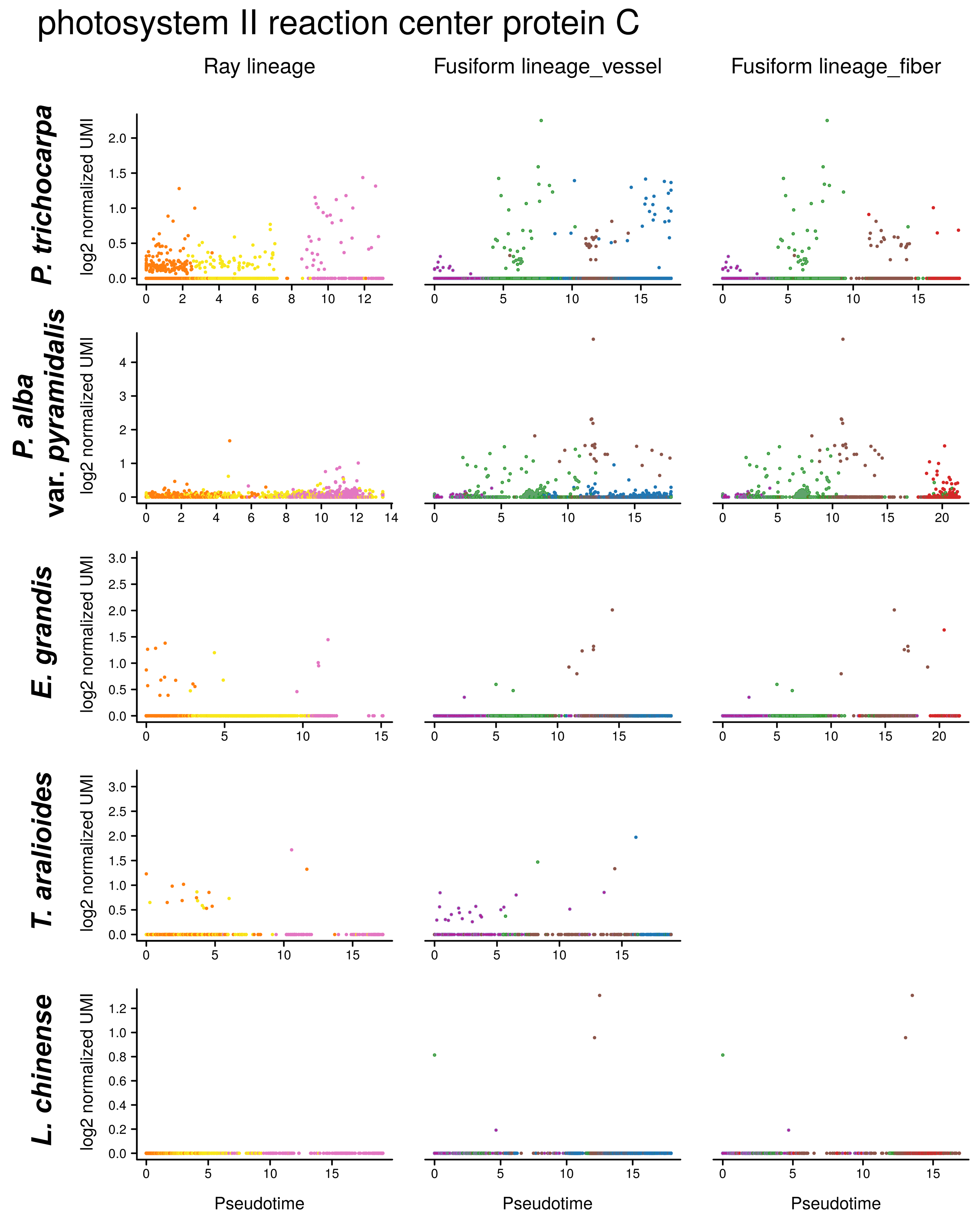

Supplement: Supplementary file 13 — Additional file 13. Expression profiles of the homologous genes of known xylem development related genes in different xylem cell trajectories of P. trichocarpa, P. alba var. pyramidalis, E. grandis, T. aralioides and L. chinense. Empty plots with no coordinates were used to represent the absence of the orthologs in certain species. [file 13059_2022_2845_MOESM13_ESM.zip › Additional file 13/Ortholog_3755_photosystem II reaction center protein C.png]

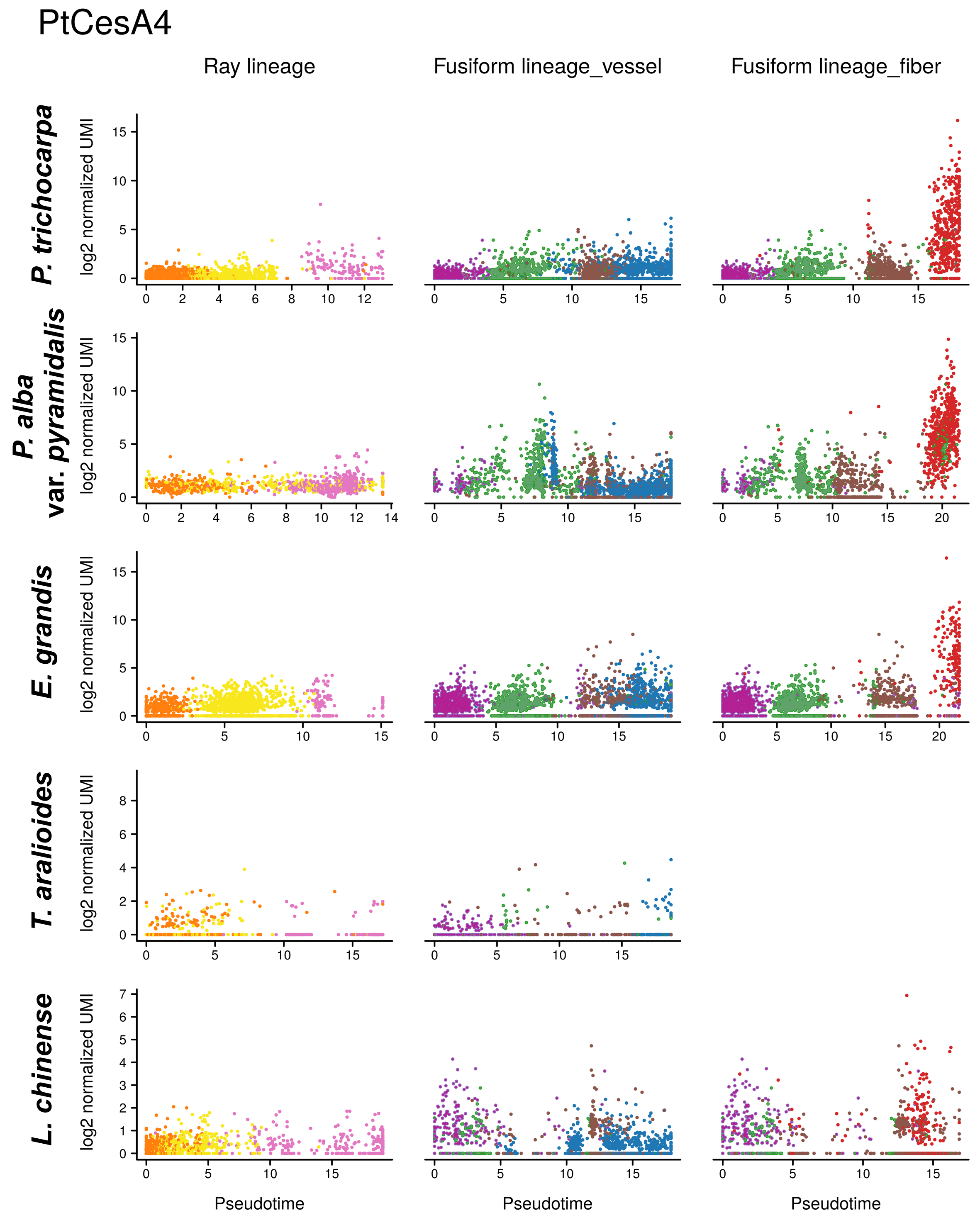

Supplement: Supplementary file 13 — Additional file 13. Expression profiles of the homologous genes of known xylem development related genes in different xylem cell trajectories of P. trichocarpa, P. alba var. pyramidalis, E. grandis, T. aralioides and L. chinense. Empty plots with no coordinates were used to represent the absence of the orthologs in certain species. [file 13059_2022_2845_MOESM13_ESM.zip › Additional file 13/Ortholog_39_PtCesA4.png]

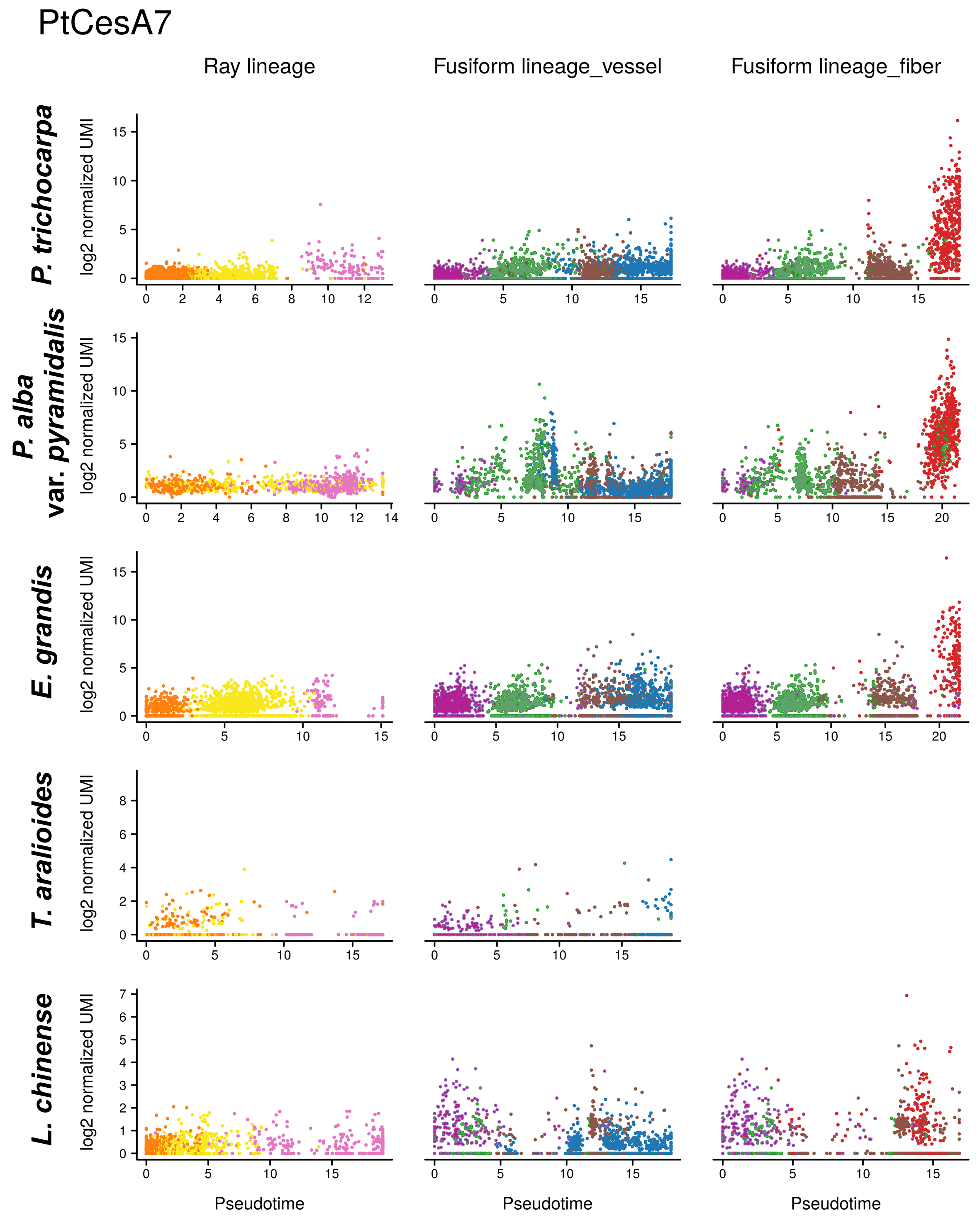

Supplement: Supplementary file 13 — Additional file 13. Expression profiles of the homologous genes of known xylem development related genes in different xylem cell trajectories of P. trichocarpa, P. alba var. pyramidalis, E. grandis, T. aralioides and L. chinense. Empty plots with no coordinates were used to represent the absence of the orthologs in certain species. [file 13059_2022_2845_MOESM13_ESM.zip › Additional file 13/Ortholog_39_PtCesA7.png]

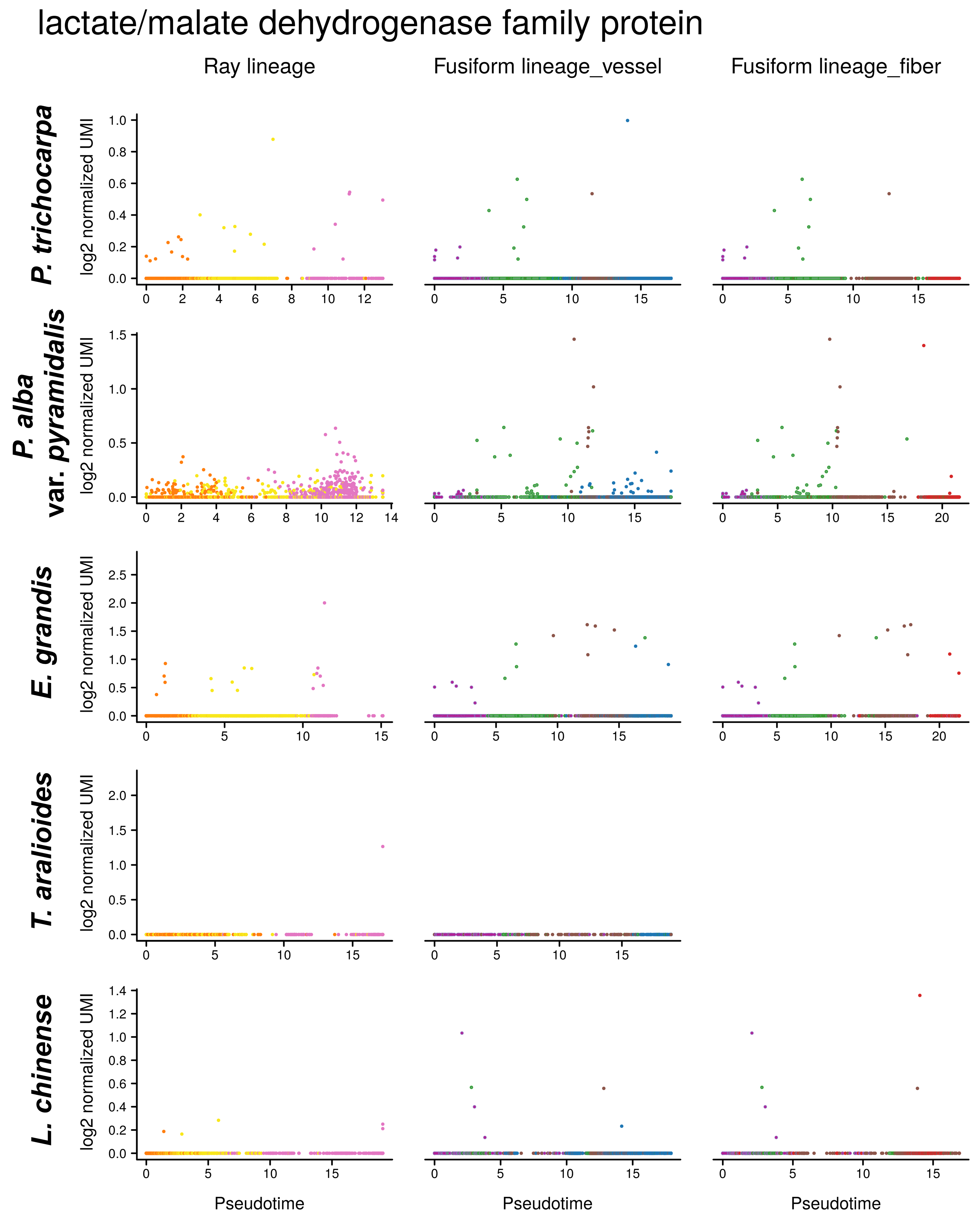

Supplement: Supplementary file 13 — Additional file 13. Expression profiles of the homologous genes of known xylem development related genes in different xylem cell trajectories of P. trichocarpa, P. alba var. pyramidalis, E. grandis, T. aralioides and L. chinense. Empty plots with no coordinates were used to represent the absence of the orthologs in certain species. [file 13059_2022_2845_MOESM13_ESM.zip › Additional file 13/Ortholog_4739_lactate malate dehydrogenase family protein.png]

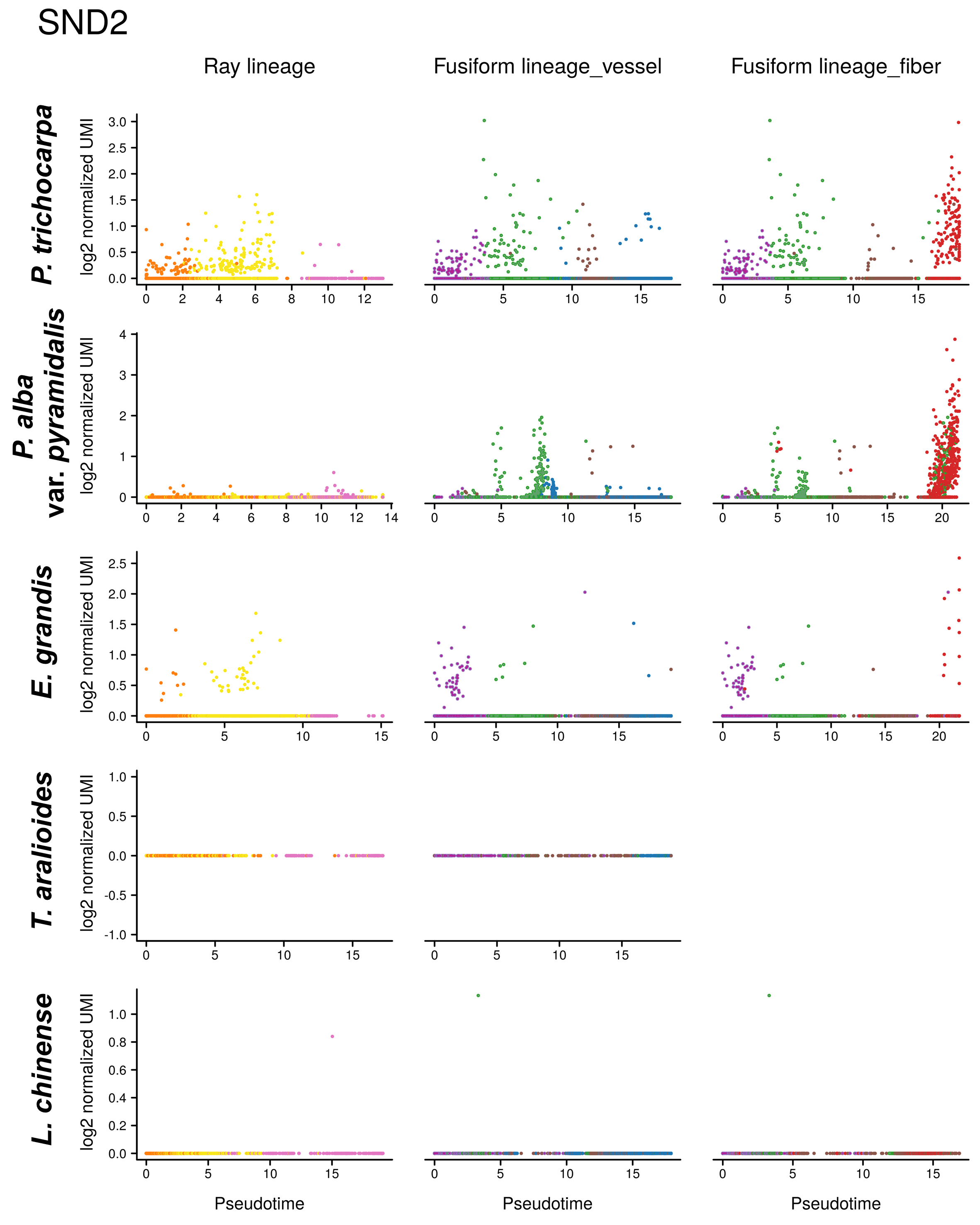

Supplement: Supplementary file 13 — Additional file 13. Expression profiles of the homologous genes of known xylem development related genes in different xylem cell trajectories of P. trichocarpa, P. alba var. pyramidalis, E. grandis, T. aralioides and L. chinense. Empty plots with no coordinates were used to represent the absence of the orthologs in certain species. [file 13059_2022_2845_MOESM13_ESM.zip › Additional file 13/Ortholog_4898_SND2.png]

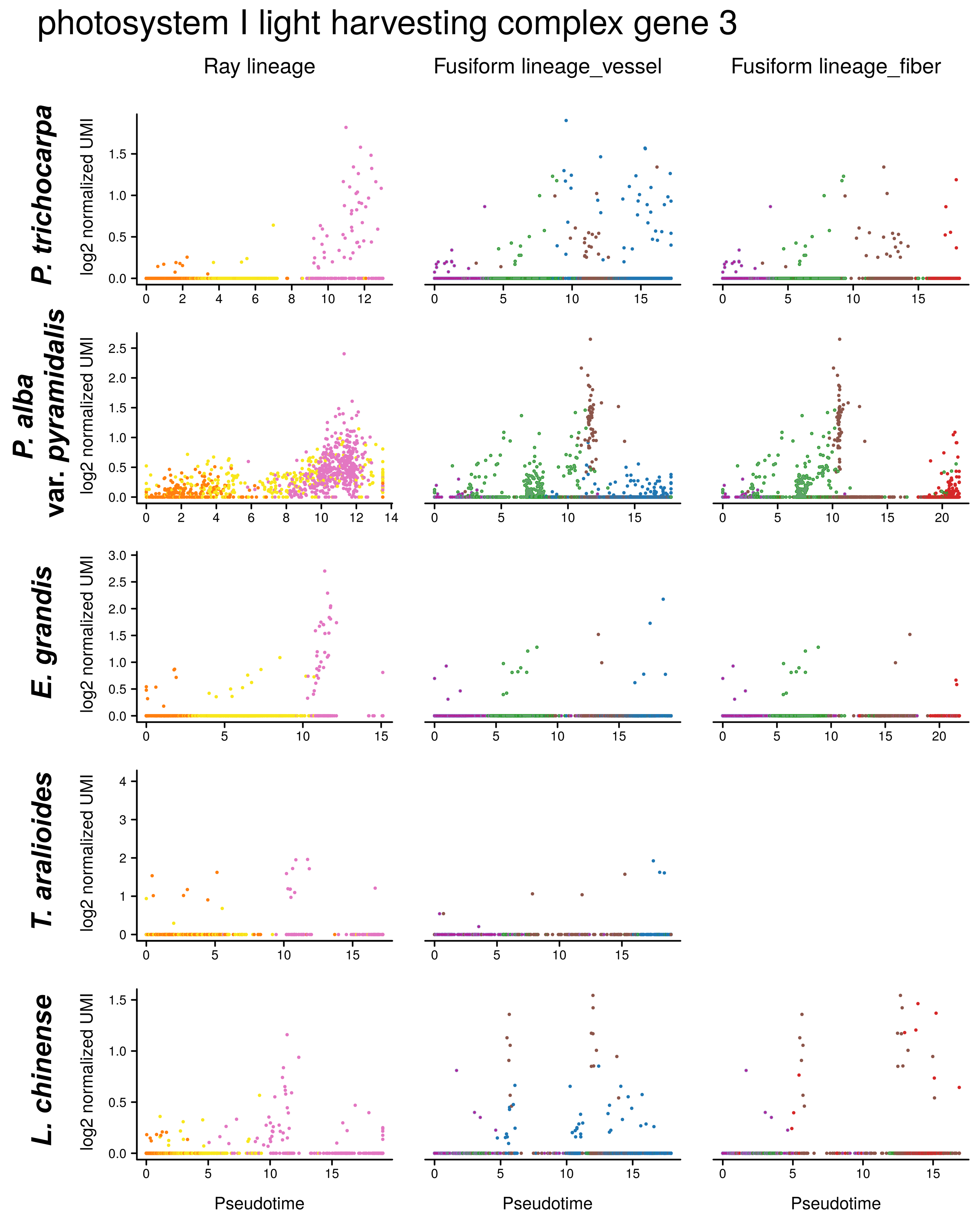

Supplement: Supplementary file 13 — Additional file 13. Expression profiles of the homologous genes of known xylem development related genes in different xylem cell trajectories of P. trichocarpa, P. alba var. pyramidalis, E. grandis, T. aralioides and L. chinense. Empty plots with no coordinates were used to represent the absence of the orthologs in certain species. [file 13059_2022_2845_MOESM13_ESM.zip › Additional file 13/Ortholog_4985_photosystem I light harvesting complex gene 3.png]

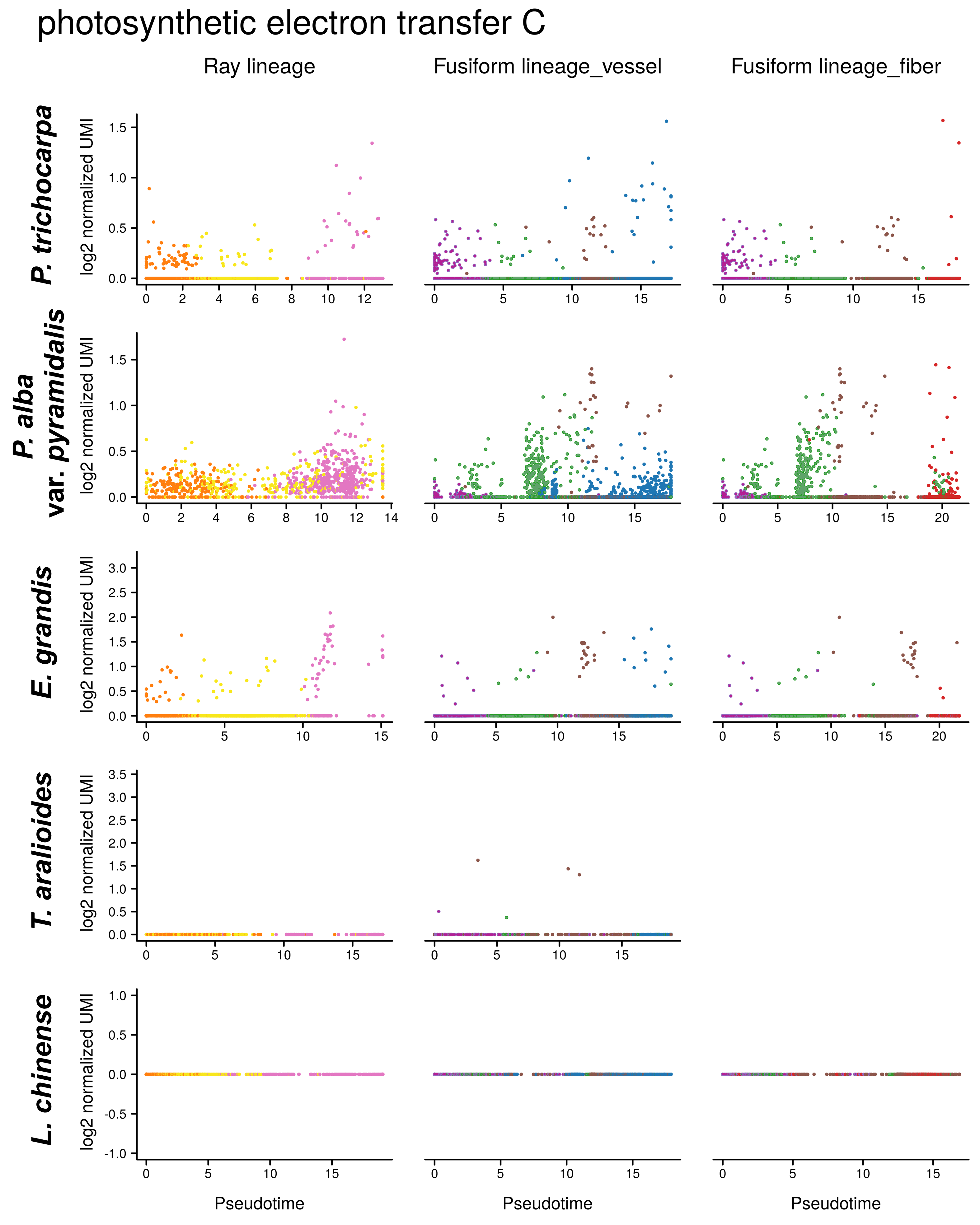

Supplement: Supplementary file 13 — Additional file 13. Expression profiles of the homologous genes of known xylem development related genes in different xylem cell trajectories of P. trichocarpa, P. alba var. pyramidalis, E. grandis, T. aralioides and L. chinense. Empty plots with no coordinates were used to represent the absence of the orthologs in certain species. [file 13059_2022_2845_MOESM13_ESM.zip › Additional file 13/Ortholog_5193_photosynthetic electron transfer C.png]

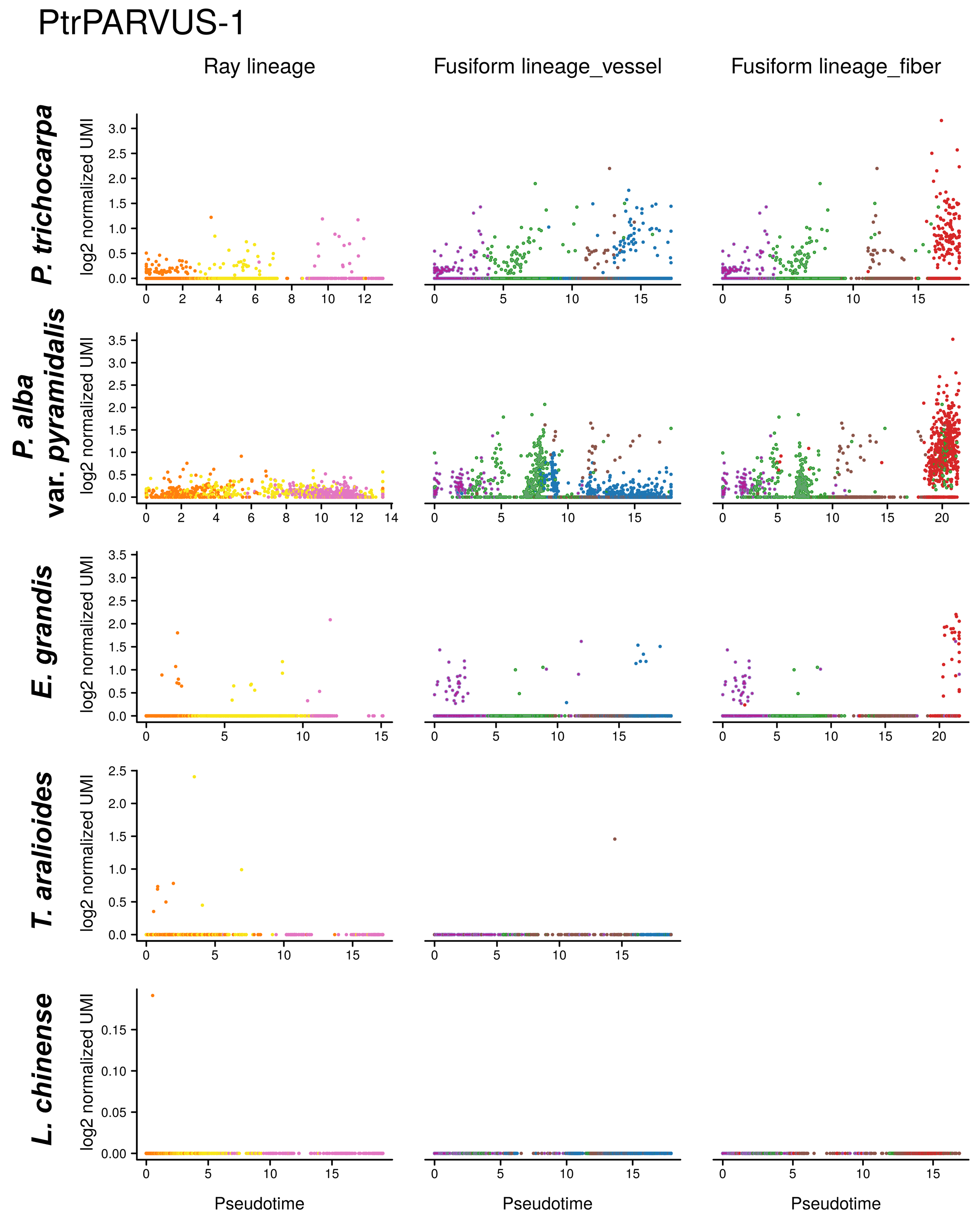

Supplement: Supplementary file 13 — Additional file 13. Expression profiles of the homologous genes of known xylem development related genes in different xylem cell trajectories of P. trichocarpa, P. alba var. pyramidalis, E. grandis, T. aralioides and L. chinense. Empty plots with no coordinates were used to represent the absence of the orthologs in certain species. [file 13059_2022_2845_MOESM13_ESM.zip › Additional file 13/Ortholog_5604_PtrPARVUS-1.png]

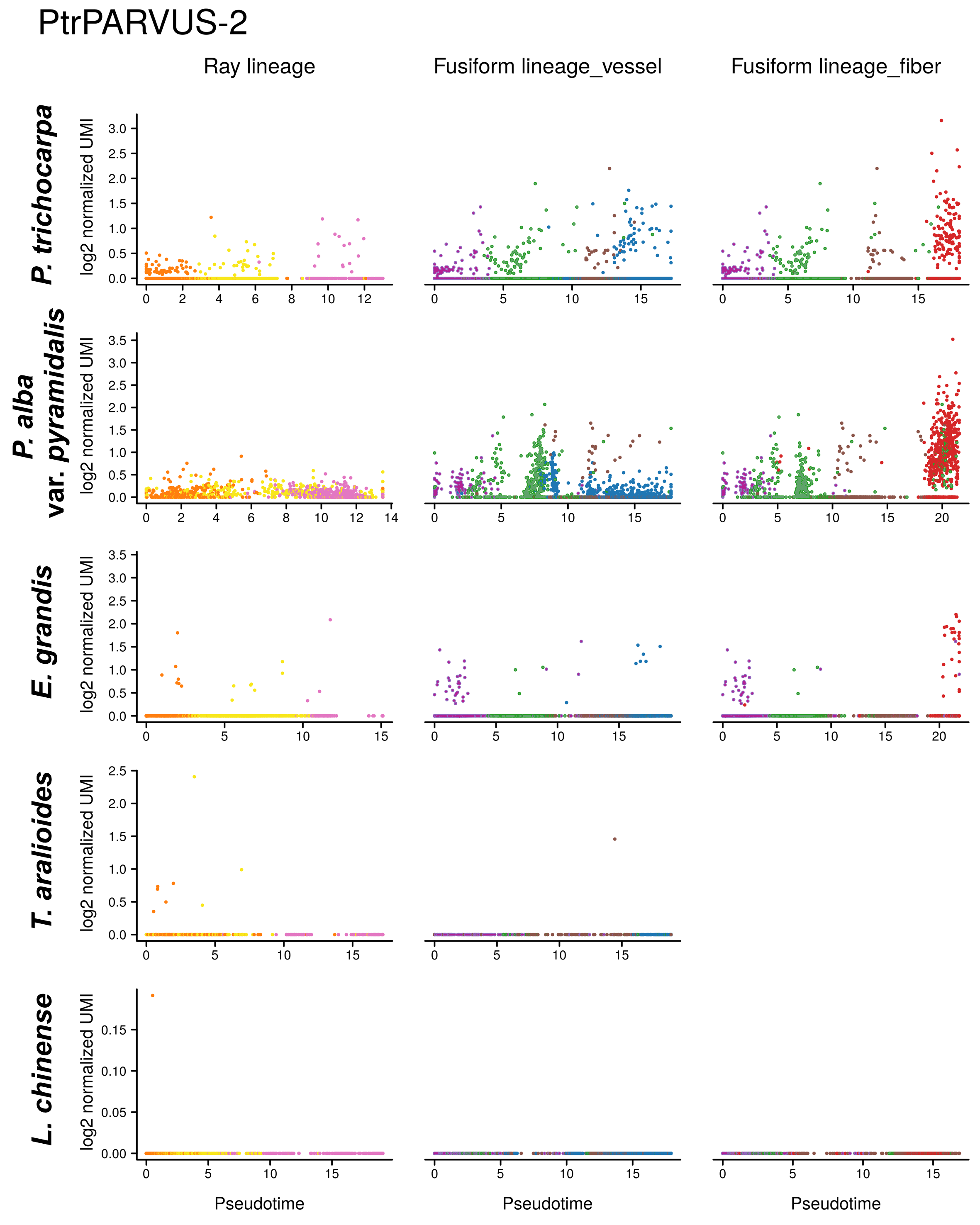

Supplement: Supplementary file 13 — Additional file 13. Expression profiles of the homologous genes of known xylem development related genes in different xylem cell trajectories of P. trichocarpa, P. alba var. pyramidalis, E. grandis, T. aralioides and L. chinense. Empty plots with no coordinates were used to represent the absence of the orthologs in certain species. [file 13059_2022_2845_MOESM13_ESM.zip › Additional file 13/Ortholog_5604_PtrPARVUS-2.png]

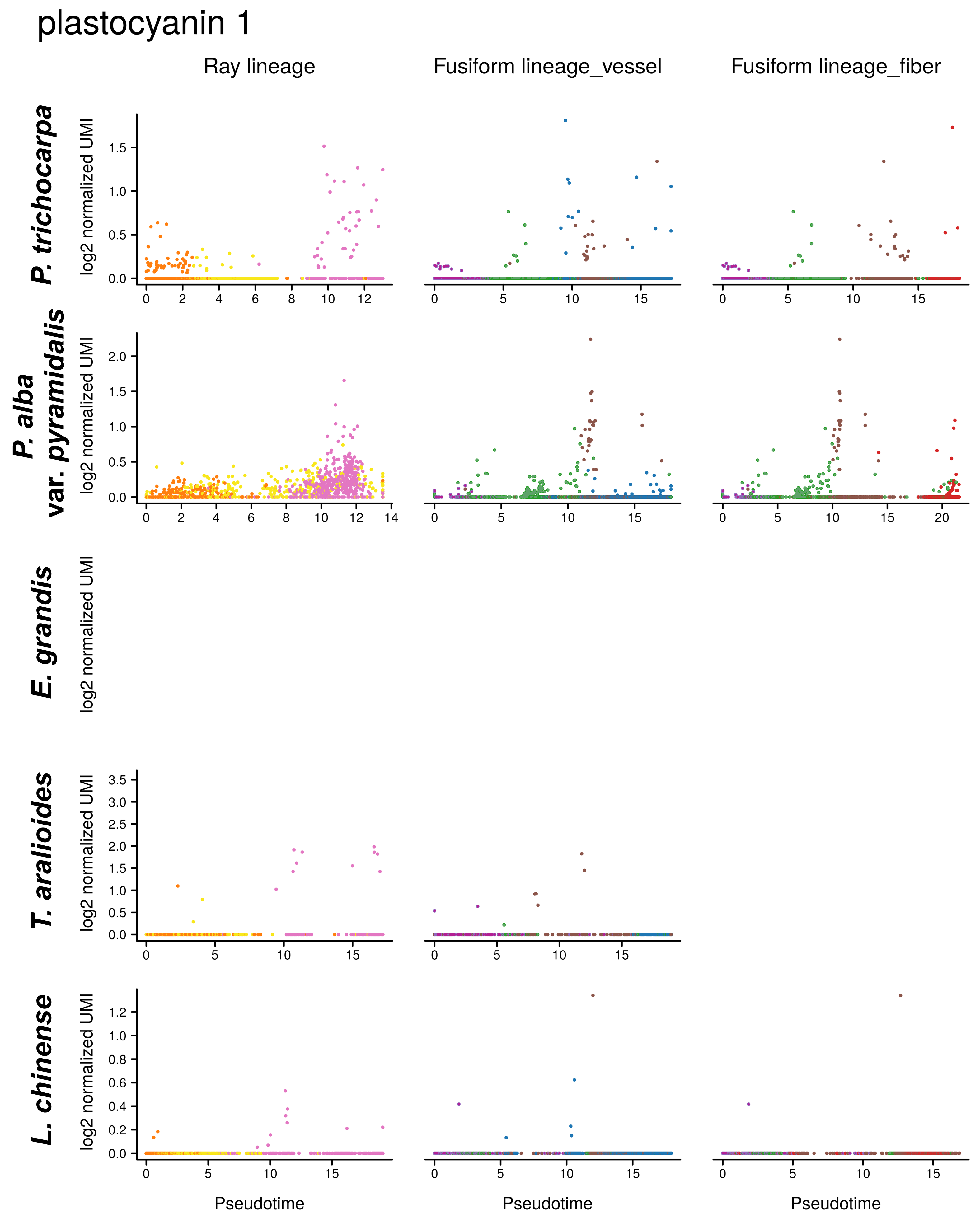

Supplement: Supplementary file 13 — Additional file 13. Expression profiles of the homologous genes of known xylem development related genes in different xylem cell trajectories of P. trichocarpa, P. alba var. pyramidalis, E. grandis, T. aralioides and L. chinense. Empty plots with no coordinates were used to represent the absence of the orthologs in certain species. [file 13059_2022_2845_MOESM13_ESM.zip › Additional file 13/Ortholog_5814_plastocyanin 1.png]

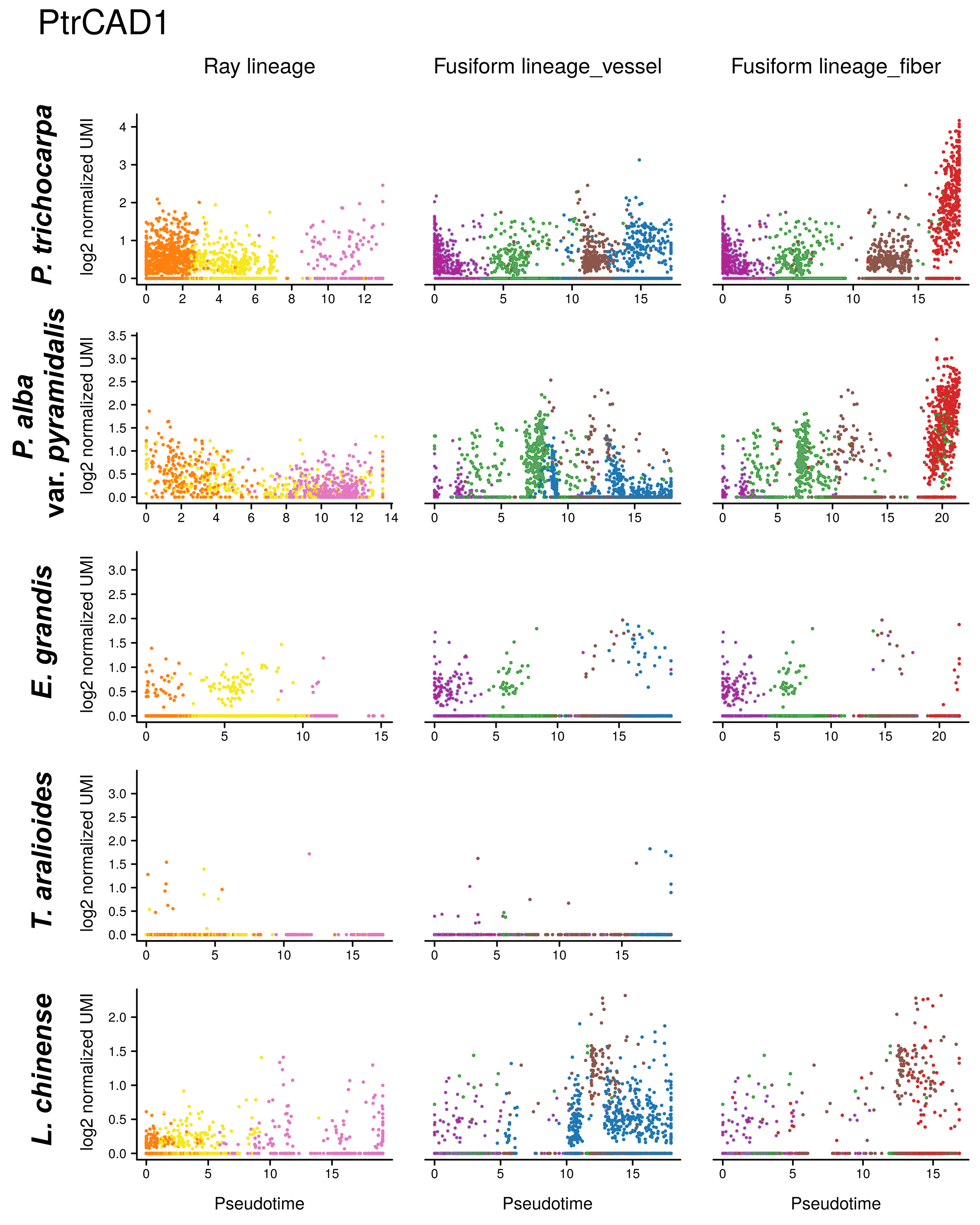

Supplement: Supplementary file 13 — Additional file 13. Expression profiles of the homologous genes of known xylem development related genes in different xylem cell trajectories of P. trichocarpa, P. alba var. pyramidalis, E. grandis, T. aralioides and L. chinense. Empty plots with no coordinates were used to represent the absence of the orthologs in certain species. [file 13059_2022_2845_MOESM13_ESM.zip › Additional file 13/Ortholog_6098_PtrCAD1.png]

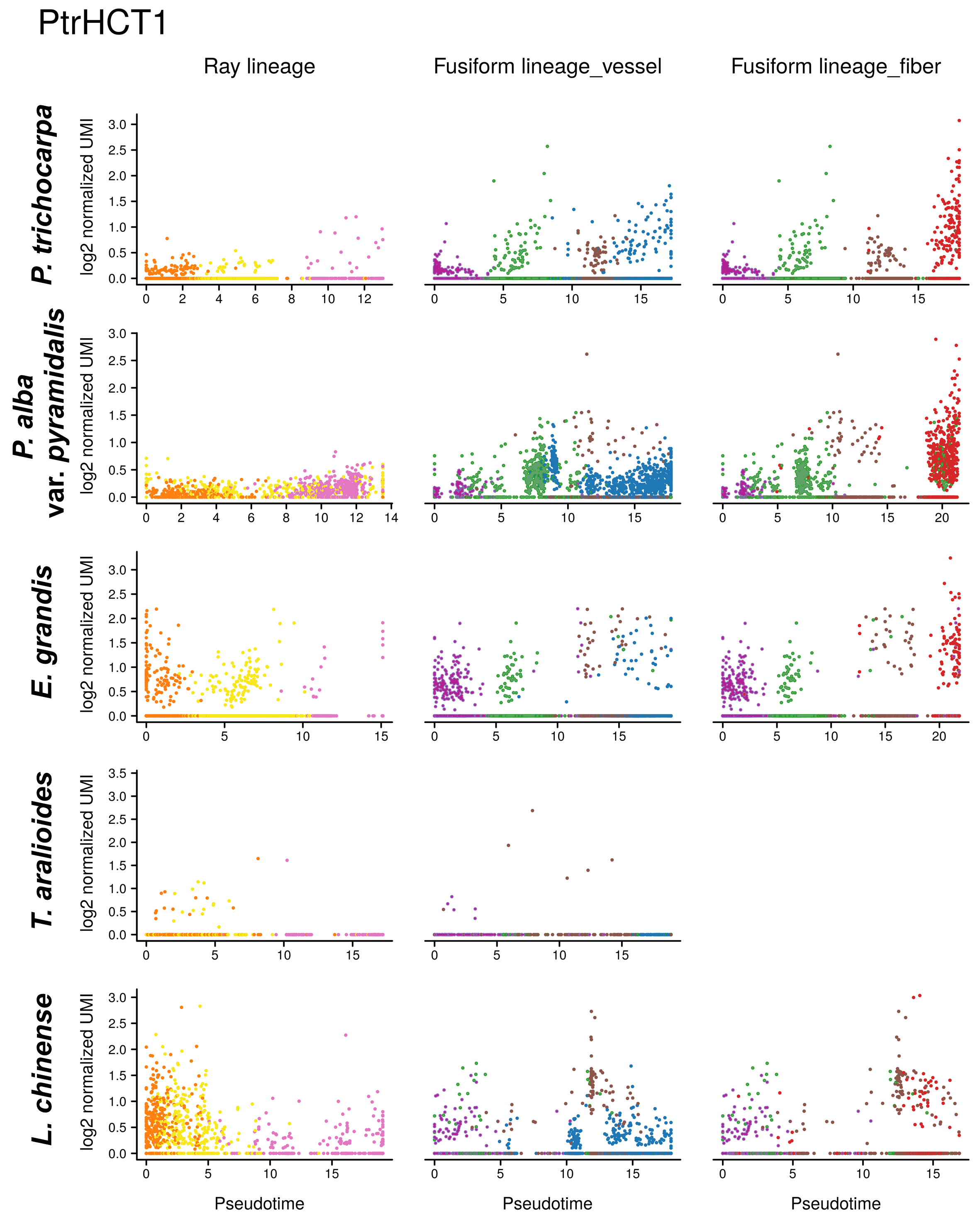

Supplement: Supplementary file 13 — Additional file 13. Expression profiles of the homologous genes of known xylem development related genes in different xylem cell trajectories of P. trichocarpa, P. alba var. pyramidalis, E. grandis, T. aralioides and L. chinense. Empty plots with no coordinates were used to represent the absence of the orthologs in certain species. [file 13059_2022_2845_MOESM13_ESM.zip › Additional file 13/Ortholog_757_PtrHCT1.png]

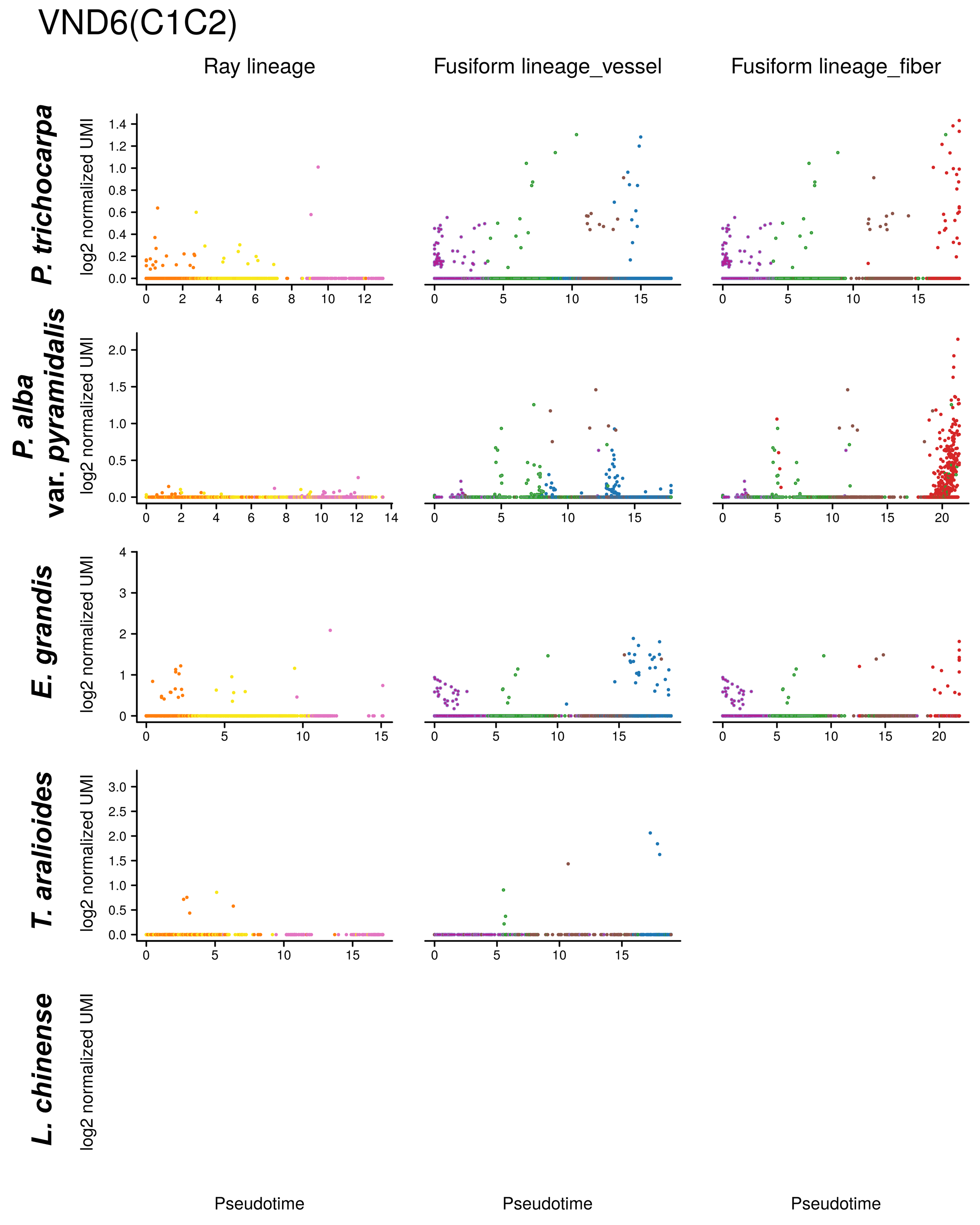

Supplement: Supplementary file 13 — Additional file 13. Expression profiles of the homologous genes of known xylem development related genes in different xylem cell trajectories of P. trichocarpa, P. alba var. pyramidalis, E. grandis, T. aralioides and L. chinense. Empty plots with no coordinates were used to represent the absence of the orthologs in certain species. [file 13059_2022_2845_MOESM13_ESM.zip › Additional file 13/Ortholog_8129_VND6(C1C2).png]

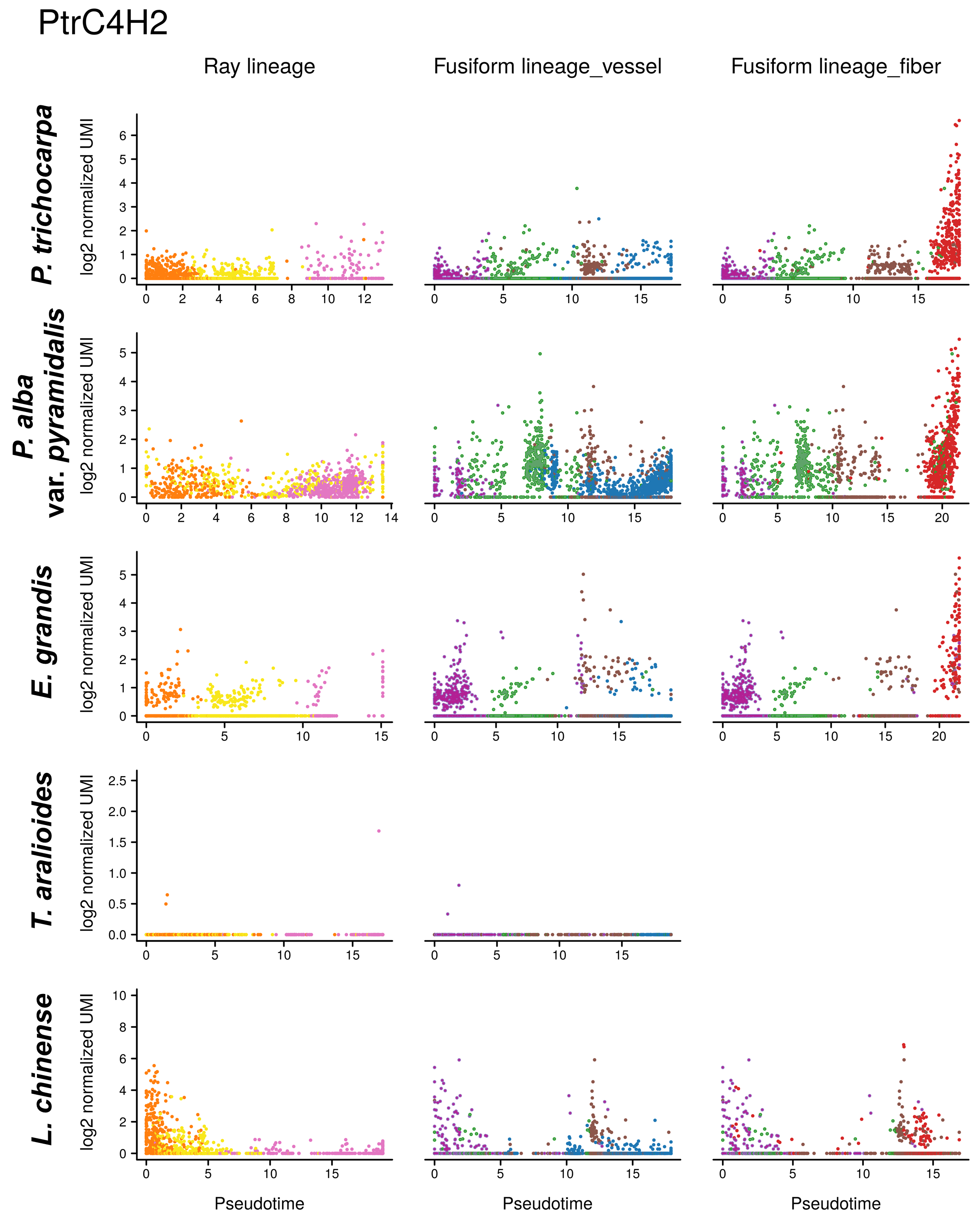

Supplement: Supplementary file 13 — Additional file 13. Expression profiles of the homologous genes of known xylem development related genes in different xylem cell trajectories of P. trichocarpa, P. alba var. pyramidalis, E. grandis, T. aralioides and L. chinense. Empty plots with no coordinates were used to represent the absence of the orthologs in certain species. [file 13059_2022_2845_MOESM13_ESM.zip › Additional file 13/Ortholog_988_PtrC4H2.png]

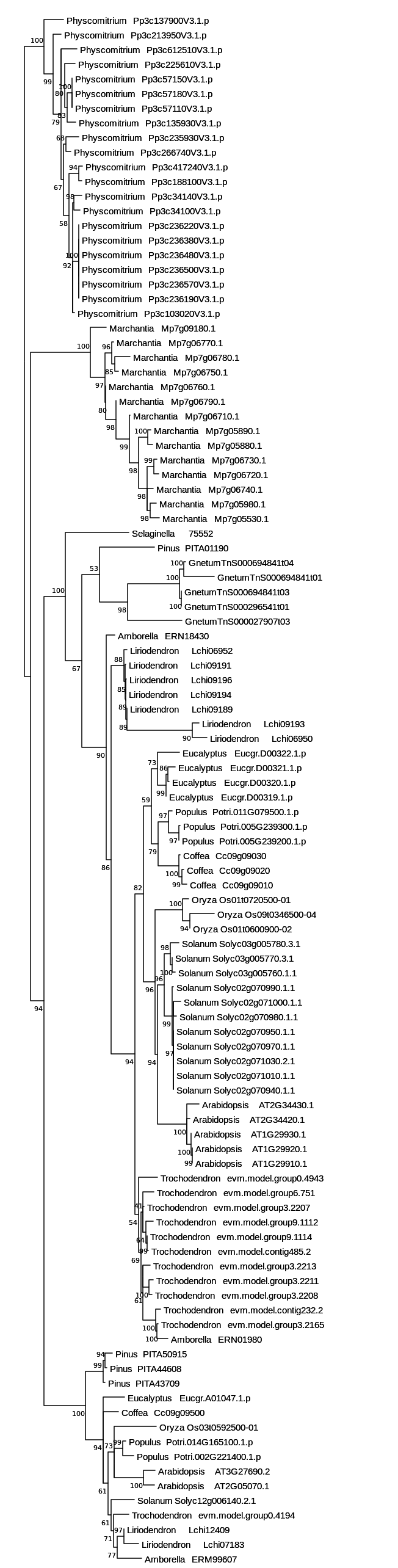

Supplement: Supplementary file 14 — Additional file 14. Phylogenetic trees of homologous genes of known xylem development related genes from 14 species. [file 13059_2022_2845_MOESM14_ESM.zip › Additional file 14/180_photosynthesis_scale_color.png]

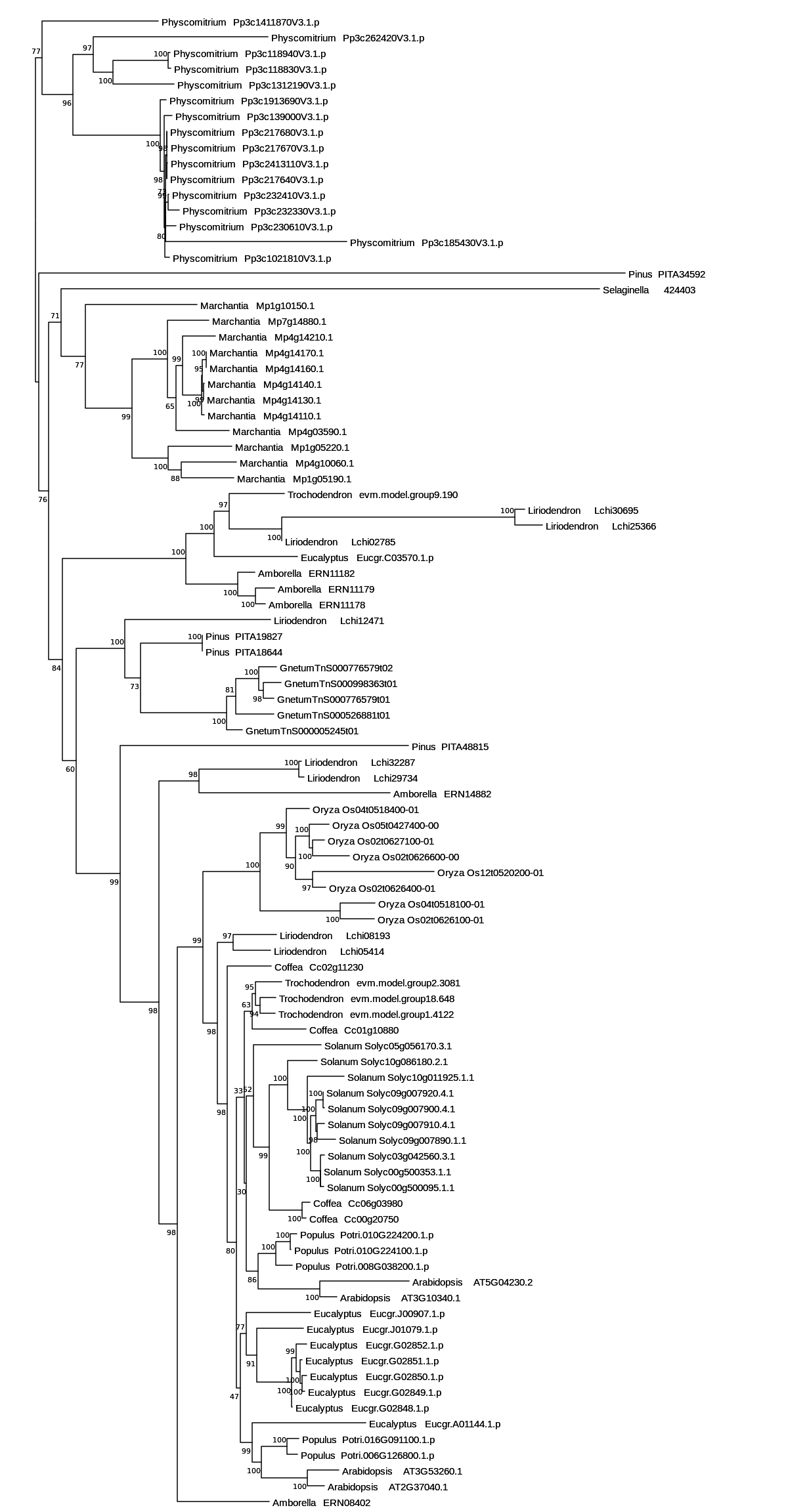

Supplement: Supplementary file 14 — Additional file 14. Phylogenetic trees of homologous genes of known xylem development related genes from 14 species. [file 13059_2022_2845_MOESM14_ESM.zip › Additional file 14/210_PtrPAL1_PtrPAL2_PtrPAL3_scale_color.png]

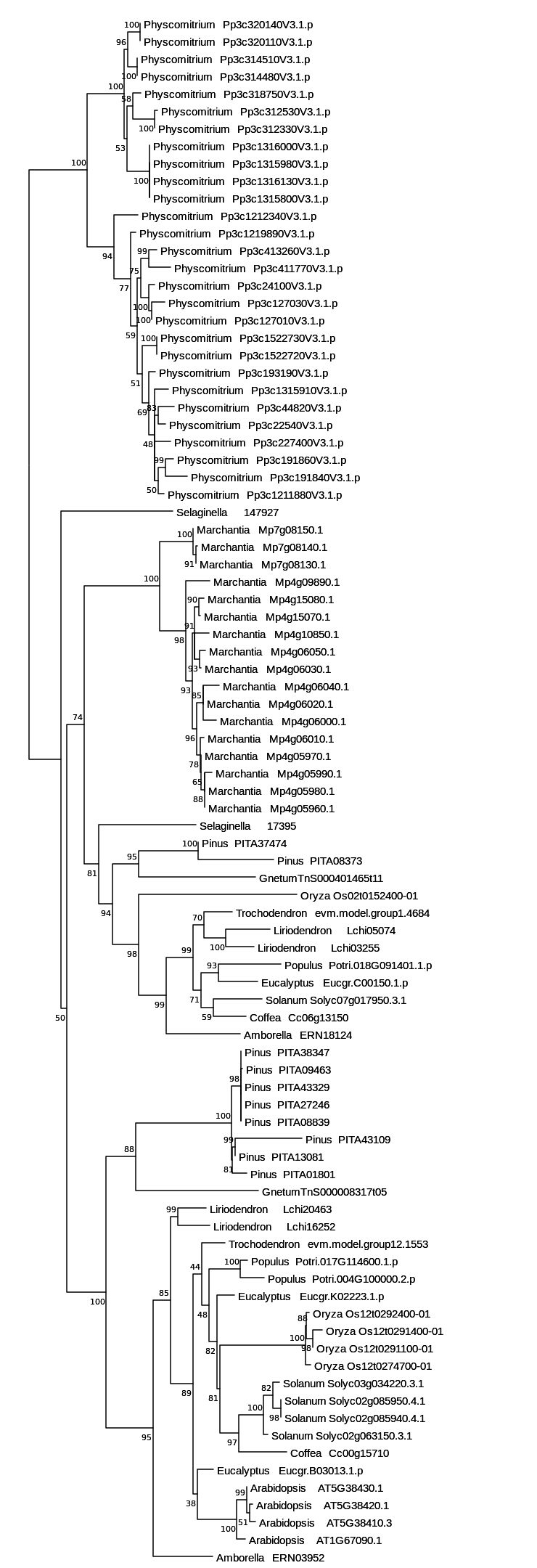

Supplement: Supplementary file 14 — Additional file 14. Phylogenetic trees of homologous genes of known xylem development related genes from 14 species. [file 13059_2022_2845_MOESM14_ESM.zip › Additional file 14/234_photosynthesis_scale_color.png]

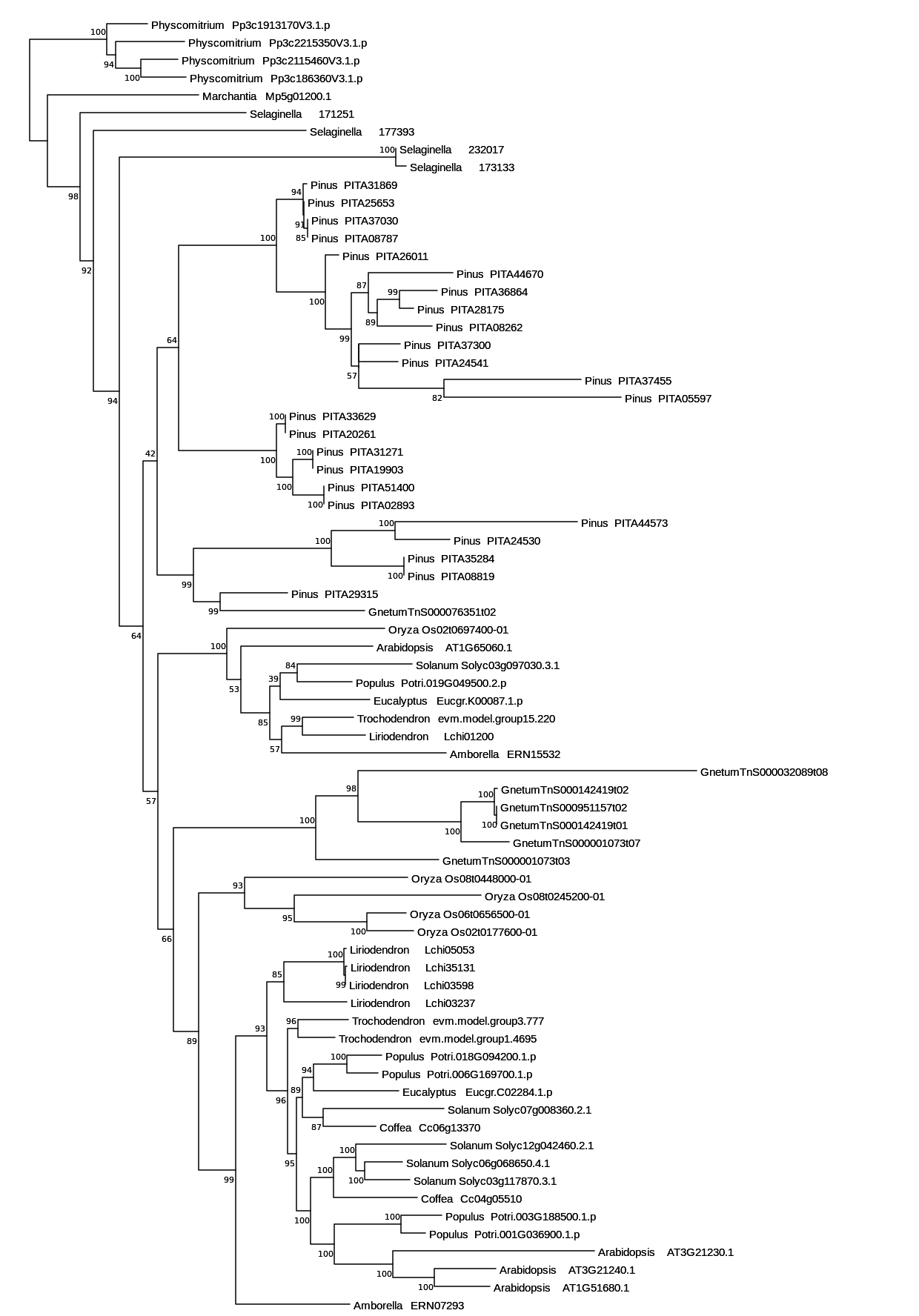

Supplement: Supplementary file 14 — Additional file 14. Phylogenetic trees of homologous genes of known xylem development related genes from 14 species. [file 13059_2022_2845_MOESM14_ESM.zip › Additional file 14/294_Ptr4CL5_scale_color.png]

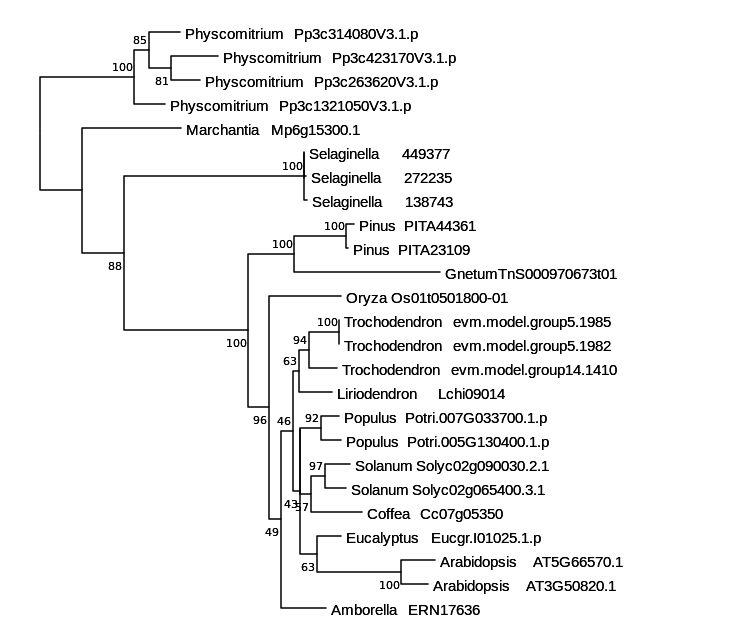

Supplement: Supplementary file 14 — Additional file 14. Phylogenetic trees of homologous genes of known xylem development related genes from 14 species. [file 13059_2022_2845_MOESM14_ESM.zip › Additional file 14/2985_photosynthesis_scale_color.png]

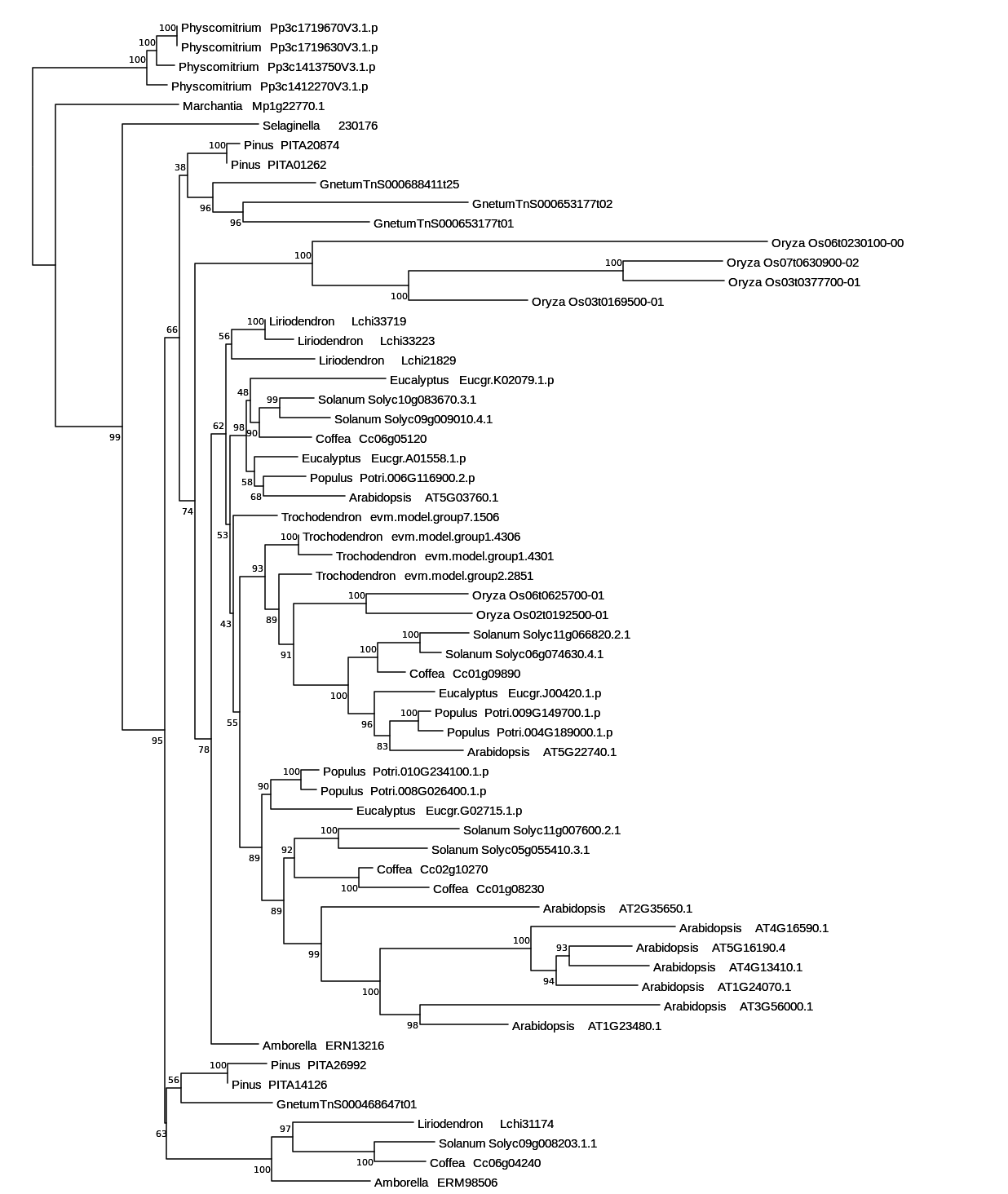

Supplement: Supplementary file 14 — Additional file 14. Phylogenetic trees of homologous genes of known xylem development related genes from 14 species. [file 13059_2022_2845_MOESM14_ESM.zip › Additional file 14/335_PtrCslA1_PtrCslA2_scale_color.png]

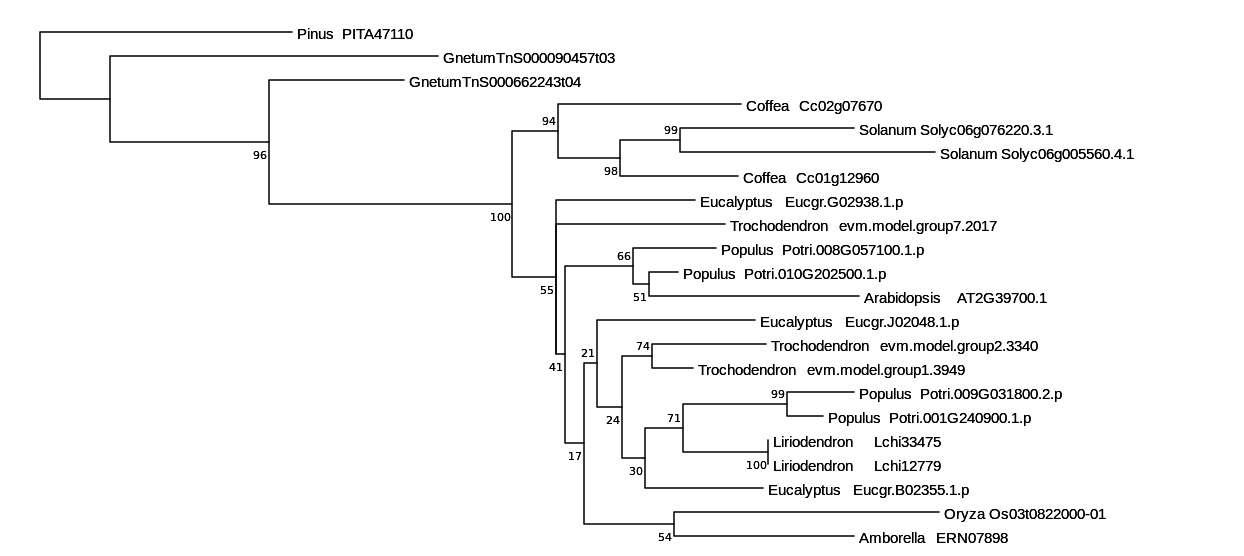

Supplement: Supplementary file 14 — Additional file 14. Phylogenetic trees of homologous genes of known xylem development related genes from 14 species. [file 13059_2022_2845_MOESM14_ESM.zip › Additional file 14/3473_ExpansinA6_scale_color.png]

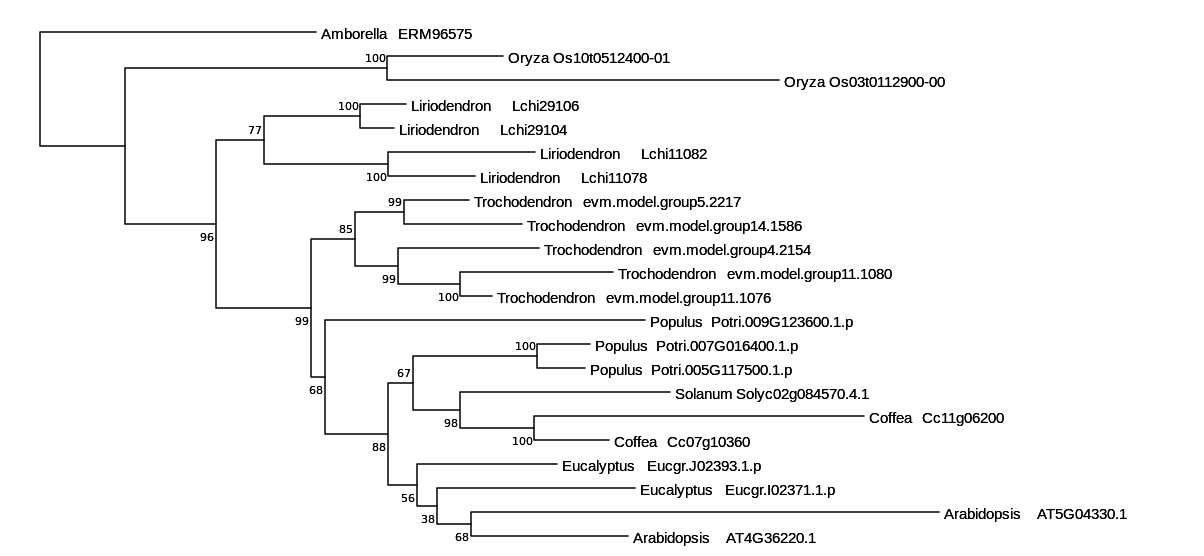

Supplement: Supplementary file 14 — Additional file 14. Phylogenetic trees of homologous genes of known xylem development related genes from 14 species. [file 13059_2022_2845_MOESM14_ESM.zip › Additional file 14/3710_PtrCAld5H1_scale_color.png]

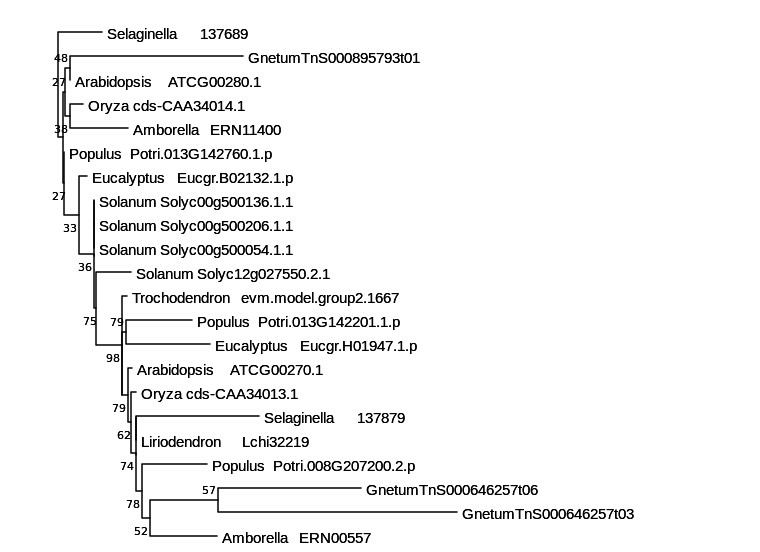

Supplement: Supplementary file 14 — Additional file 14. Phylogenetic trees of homologous genes of known xylem development related genes from 14 species. [file 13059_2022_2845_MOESM14_ESM.zip › Additional file 14/3755_photosynthesis_scale_color.png]

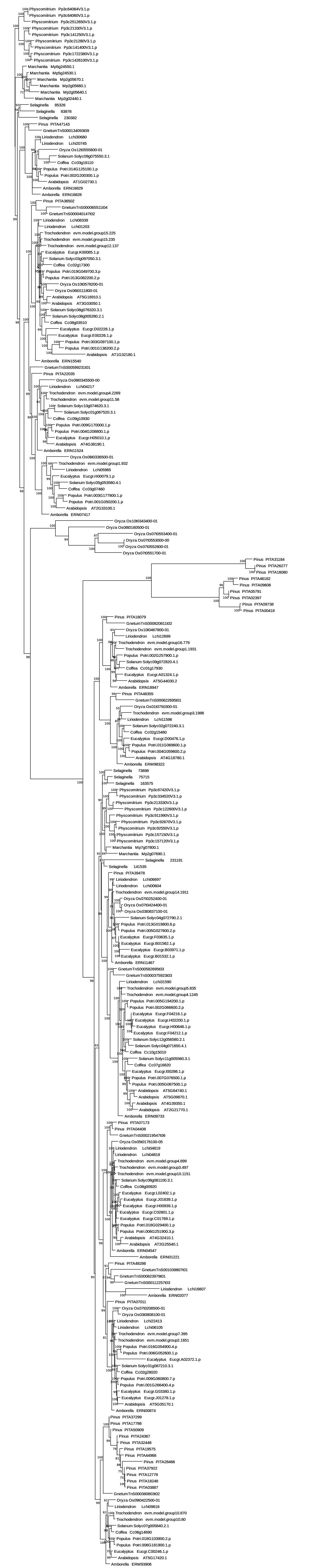

Supplement: Supplementary file 14 — Additional file 14. Phylogenetic trees of homologous genes of known xylem development related genes from 14 species. [file 13059_2022_2845_MOESM14_ESM.zip › Additional file 14/39_PtCesA4_PtCesA7_scale_color.png]

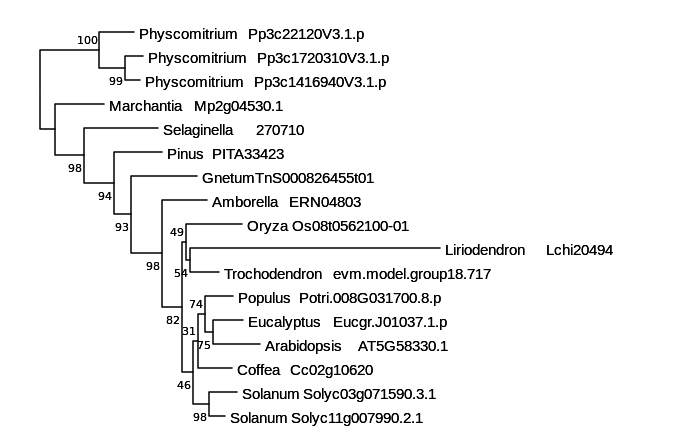

Supplement: Supplementary file 14 — Additional file 14. Phylogenetic trees of homologous genes of known xylem development related genes from 14 species. [file 13059_2022_2845_MOESM14_ESM.zip › Additional file 14/4739_photosynthesis_scale_color.png]

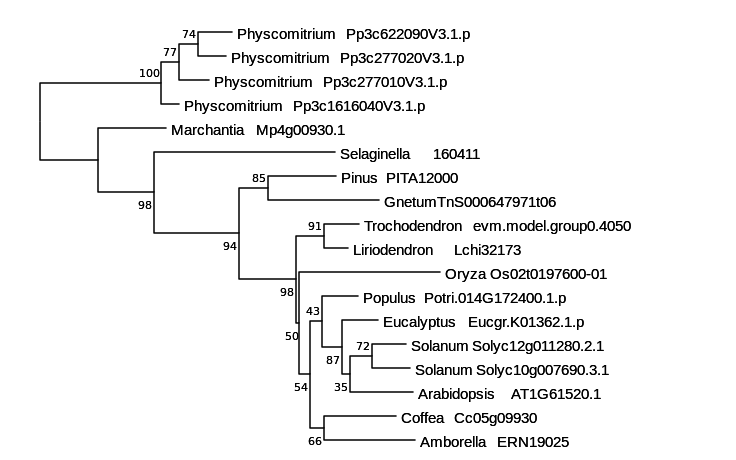

Supplement: Supplementary file 14 — Additional file 14. Phylogenetic trees of homologous genes of known xylem development related genes from 14 species. [file 13059_2022_2845_MOESM14_ESM.zip › Additional file 14/4985_photosynthesis_scale_color.png]

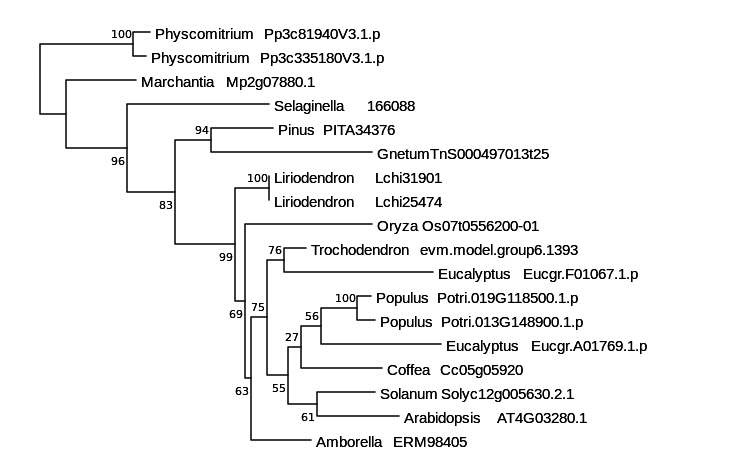

Supplement: Supplementary file 14 — Additional file 14. Phylogenetic trees of homologous genes of known xylem development related genes from 14 species. [file 13059_2022_2845_MOESM14_ESM.zip › Additional file 14/5193_photosynthesis_scale_color.png]

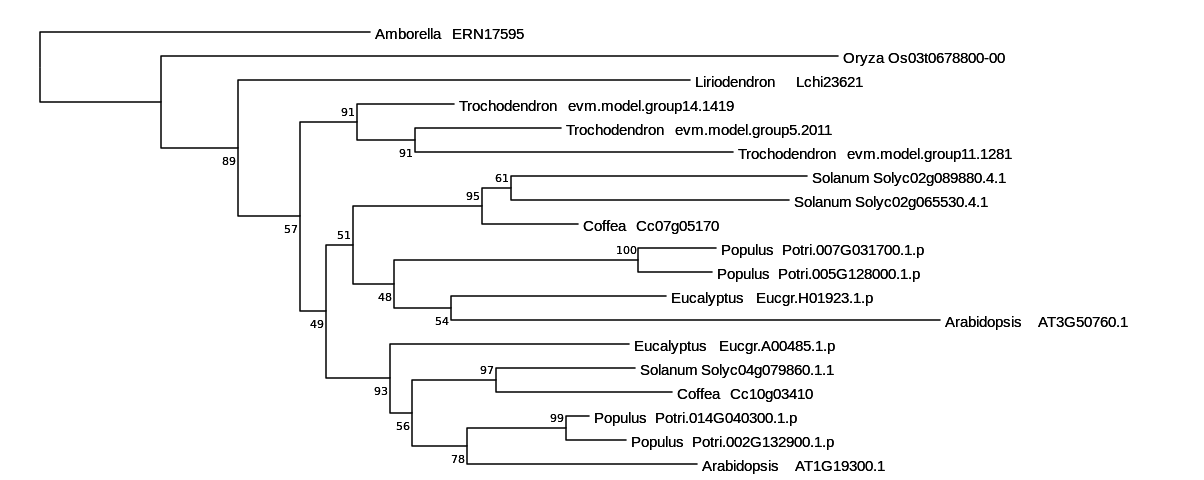

Supplement: Supplementary file 14 — Additional file 14. Phylogenetic trees of homologous genes of known xylem development related genes from 14 species. [file 13059_2022_2845_MOESM14_ESM.zip › Additional file 14/5604_PtrPARVUS-1_PtrPARVUS-2_scale_color.png]

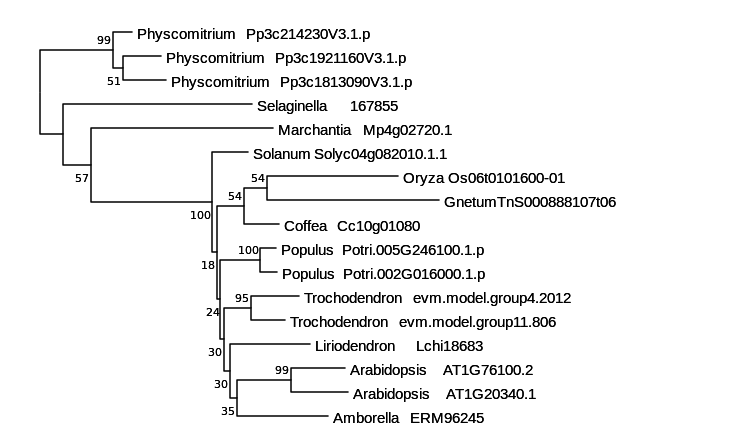

Supplement: Supplementary file 14 — Additional file 14. Phylogenetic trees of homologous genes of known xylem development related genes from 14 species. [file 13059_2022_2845_MOESM14_ESM.zip › Additional file 14/5814_photosynthesis_scale_color.png]

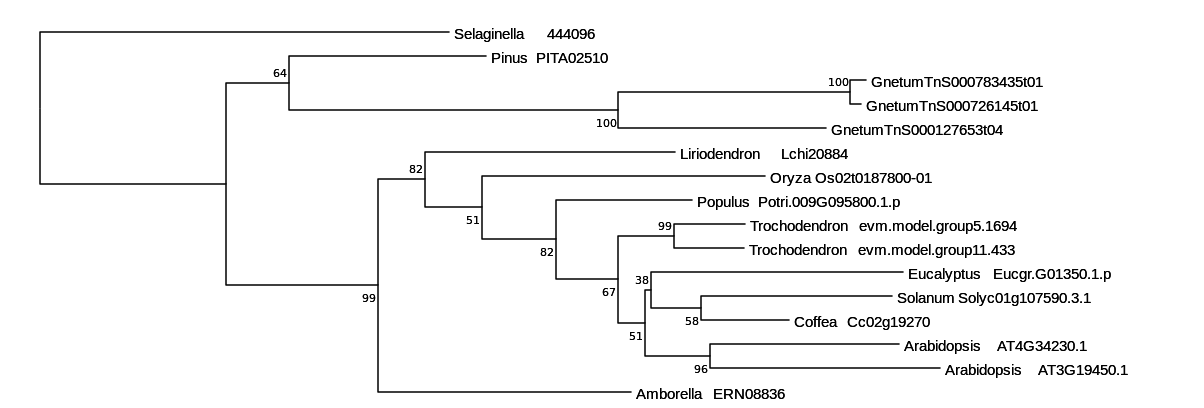

Supplement: Supplementary file 14 — Additional file 14. Phylogenetic trees of homologous genes of known xylem development related genes from 14 species. [file 13059_2022_2845_MOESM14_ESM.zip › Additional file 14/6098_PtrCAD1_scale_color.png]

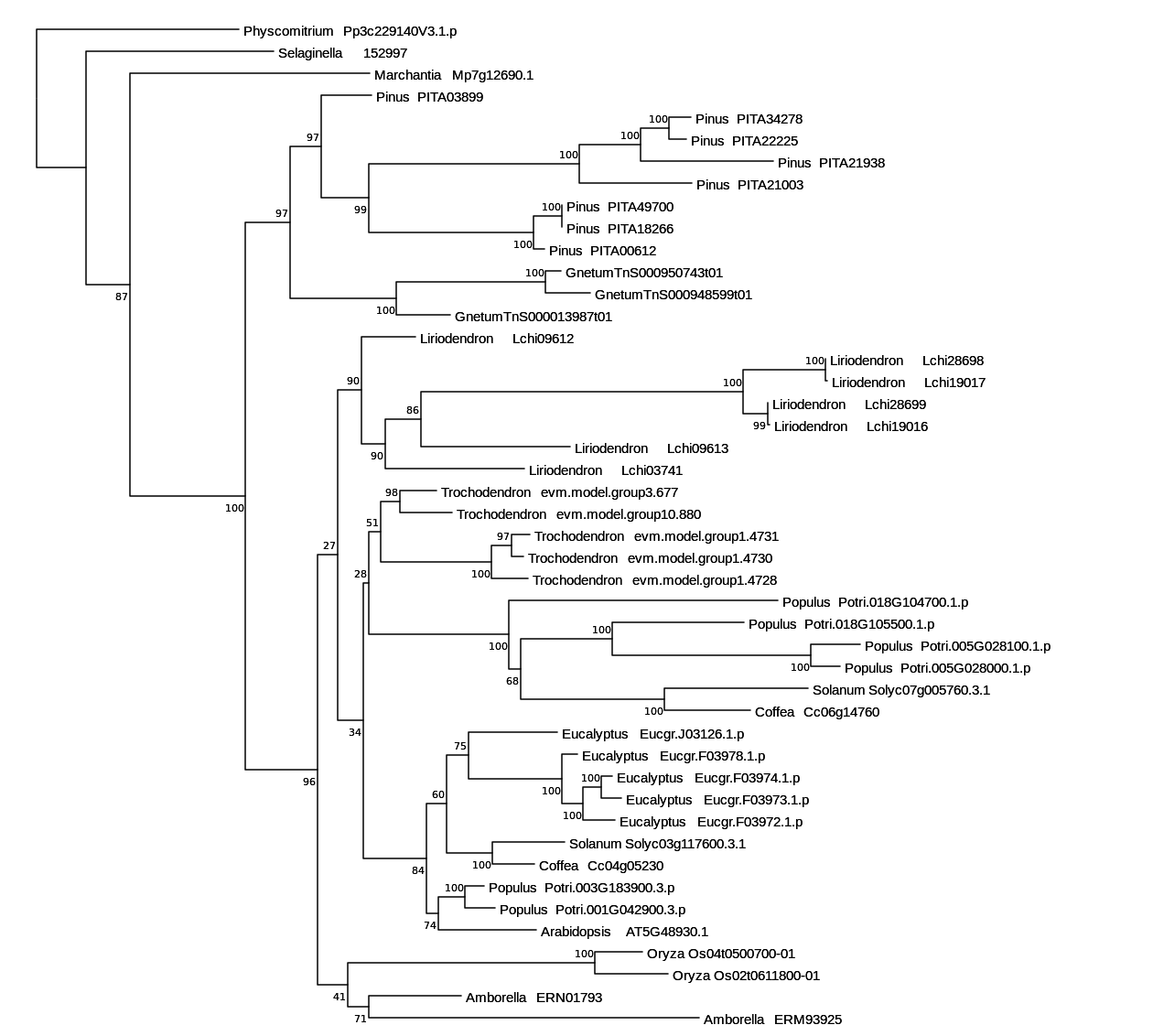

Supplement: Supplementary file 14 — Additional file 14. Phylogenetic trees of homologous genes of known xylem development related genes from 14 species. [file 13059_2022_2845_MOESM14_ESM.zip › Additional file 14/757_PtrHCT1_scale_color.png]

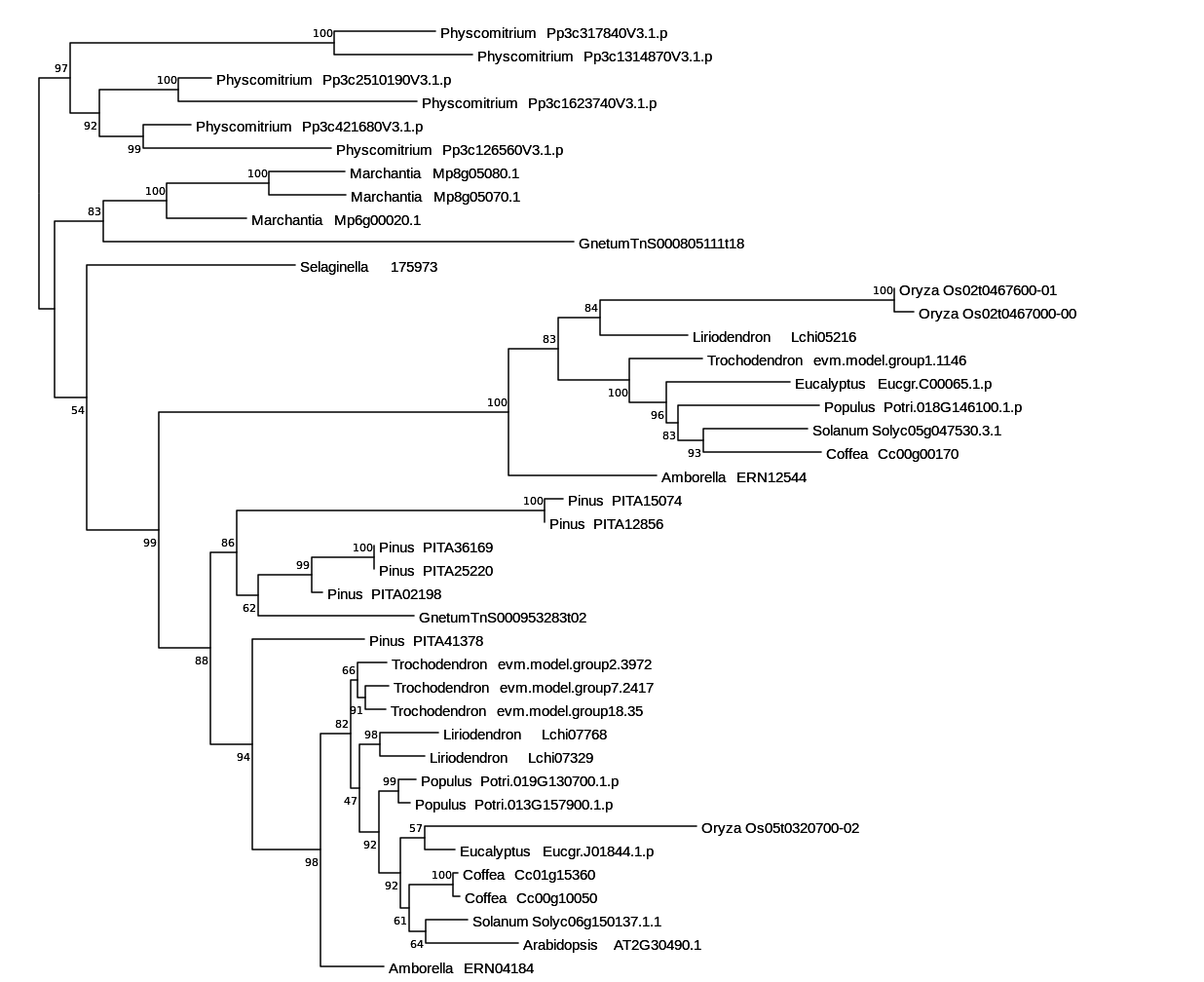

Supplement: Supplementary file 14 — Additional file 14. Phylogenetic trees of homologous genes of known xylem development related genes from 14 species. [file 13059_2022_2845_MOESM14_ESM.zip › Additional file 14/988_PtrC4H2_scale_color.png]
