# Supplementary material for: Single-cell transcriptomics unveils xylem cell development and evolution
Source: Genome Biol. 2023 Jan 9;24:3. doi: 10.1186/s13059-022-02845-1 (PMC9830878; doi:10.1186/s13059-022-02845-1)
Supplement: Supplementary file 17 — Additional file 17. Review history. [file 13059_2022_2845_MOESM17_ESM.docx]

Review History

**First round of review**

**Reviewer 1**

**Were you able to assess all statistics in the manuscript, including the appropriateness of statistical tests used?**

Yes, and I have assessed the statistics in my report.

**Comments to author:**

Xylem development produces the largest plant biomass on the earth. This manuscript analyzed the formation and developmental dynamics of stem-differentiating xylem in four woody plants by using laser capture microdissection RNA-seq and single-cell RNA-seq (10x Genomics Chromium technology and fluorescence-activated cell sorting coupled with the plate-based MARS-seq method). The authors firstly focused on P. trichocarpa and identified the cell types of SDX, then compared the SDX cell types with other three angiosperms: E. grandis (core eudicot), T. aralioides (basal eudicot), and L. chinense (magnoliid). Based on the above data and analysis, the authors revealed the common and unique developmental trajectories for the fusiform and ray lineages. Finally, they examined the expression patterns of the known genes for xylem development, and laid special stress on analyzing the transcriptional expression and phylogenetic history for VNS family and SND2 and found their possible link to libriform fiber development in tracheid-bearing plants. Totally, this is a well-designed evo-devo study for xylem development study. Compared to previous scRNA-seq studies for xylem, this manuscript exhibits a higher resolution map to illustrate the developmental paths of xylem cells.

However, some following major and minor concerns should be answered: Major concerns:

1, Because the authors identified the transcriptional profiling for libriform fiber, vessel element, and ray parenchyma by using the laser capture microdissection from the tissue sections, the cell identities for the three cell types of the scRNA-seq cell clusters in this study are convincible. While for the pseudo-time analysis for the cell populations, the development path seems to be only based on gene expression of scRNA-seq data. In principle, the start and end cell clusters and the direction of the trajectory should be analyzed aided by time-course RNA-seq. I strongly suggest that the authors performed bulked RNA-seq for the stem xylems at different developmental stages, such as the examples from different internodes. Current development trajectories were only based on the characterization of three terminal differentiated cell types, and thus the prediction for the path and developmental direction is more like a conjecture based on existing knowledge.

2, In the analysis of the cell cloud of UMAP, it is very far-fetched or can even be said to be completely wrong to judge the relationship between each two cell types just based on the "intermixing". During UMAP's dimensionality reduction process, such so-called "intermixing" actually is a prevalent problem. Although they are "intermixed" in 2D, they may be far away at third latitudes or higher. Giving biological meaning to explain the cell differentiation or transition based on just such a small amount of intermixed cells is a very risky proposition. In lines 172-174, actually no clear "intermixing" could be seen in my eyes based on your current figures. In lines 209-210, the conclusion is seriously flawed and might deviate from the factual basis. If the authors insisted on this, please provide more evidence.

3, It is worth noting that the authors attempt to conduct comparative single cell-transcriptomics studies for four plant species. It's a very meaningful attempt. However, in the selection of materials, no emphasis is placed on the selection of plant species with significant differences in xylem tissue development characteristics, for example, gymnosperms that lack xylem vessel elements and fibers can be considered for comparison. If possible, it would be a better option to introduce a gymnosperm to the study.

Minor concerns:

1, What is the "DGE" for an individual cell group, such as one of that in line 231? Is that mean maximum expressed genes? Usually, DGE only means the differentially expressed genes between two samples, so it is not suitable to use it here.

2, For the materials, the authors use the different internodes of stems for different plants, what is the criterion for judging this? Is there a marker at the molecular level? These different internodes may be at different stages of development and may also have a significant impact on the results.

3, The quality of the pictures provided by this manuscript is too bad, which seriously affects the reviewer's understanding of the content.

4, The results of the article need to be described in a more objective way, and not too literary, such as line327, "the tip of the iceberg".

5, The section "The reproducibility of the scRNA-seq results" should be adjusted to the front part or the method section. Current position looks a little strange.

**Reviewer 2**

**Were you able to assess all statistics in the manuscript, including the appropriateness of statistical tests used?**

Yes, and I have assessed the statistics in my report.

**Comments to author:**

The paper presents both single-cell and laser capture microdissection transcriptomic profiling of xylem cell types across four divergent angiosperm tree species. The paper is well structure and well written, and provide a clear leap in scope and understanding compared to previous work.

Some aspects of the paper should be celarified:

L38: «an evolutionarily reversed character». It is unclear to me what this means. Character -> trait?

L40: Why abbreviate "evo" and not "development"? "evo-devo" is commonly used as an expression.

L50-L56: Could you clarify how apical and lateral growth are linked in this paragraph too? Does the vascular cambium arise from the shoot apical meristem? That's what it sounds like, but you don't actually say it.

L58: Is this the right use of the word "pluripotency"? Do you mean that the ability of a stem cell to differentiate into other cell types is maintained by organizer cells? Or that the type of cell differentiation is maintained by organizer cells?

L131: It is unclear what "both these cell clusters" refer to since there are three clusters mentioned in the previous sentence. Also, "DEGs upregulated" compared to what? The other clusters?

L155-6: Confusing sentence. Ptr8 is not in the right part of the figure(?). It would be better to simply say that the other clusters are unidentified.

L240: Why would "a few master regulators" result in low correlations of homologous cell types?

L240: It is unclear how correlation was computed across species. Few additional details are given in the method section (L711). To compute correlation between samples across species you need a set of common genes (or a representative gene from each orthogroup). Did you compute mean/sum expression across paralogs within each orthogroup? How many orthogroups was used to compute correlation?

Figure 4: It is not clear to me what the colors represent in this figur. Does red in A) mean mostly cells from trichocarpa compared to grandis?

L373: "the expression patterns …were different … due to their low expression". This does not make sense to me. Was it the patterns or the level that was different?

L432: "has been remained" -> "has remained"

L435: "which took six-fold time more than that required". I don't understand the meaning of this sentence. Rewrite.

L504: "Two contradicted conclusions" -> "Two contradicting conclusions ".

**Reviewer 3**

**Were you able to assess all statistics in the manuscript, including the appropriateness of statistical tests used?**

No, I do not feel adequately qualified to assess the statistics.

**Comments to author:**

This is a very elaborate analysis of gene expression on a single cell level in the woody tissues of Populus trichocarpa. It goes beyond the previously published scRNAseq analyses of protoplasts isolated from the woody tissues of tree stems in using laser dissection to validate the scRNAseq data, and in trying to utilise the P. trichocarpa scRNAseq data to compare xylem cell differentiation and functionalities between four different angiosperm tree species. The work seems to be done in a careful way. Indeed, the manuscript presents analyses of higher number of xylem cells, higher number of biological replicates, and higher number of different techniques for the sequencing (scRNAseq and MARSseq) than the previous studies in Arabidopsis or Populus vasculature. They also seem to have identified a significant mistake in an earlier scRNAseq analysis of Populus wood, which is valuable. I have however some concerns on a few parts of the manuscript. The most serious one concerns the clustering analysis of the Populus trichocarpa scRNAseq data and the conclusions drawn from this.

Clustering of the Populus trichocarpa scRNAseq data

10 clusters are identified. The first question is why only one biological replicate (with only 4705 cells) was used for the clustering as altogether seven replicates were used for P. trichocarpa?

The clusters are given cellular identities on the basis of comparison with the laser dissected material. This is great even though it would not have harmed to validate the identities with reporter lines for some marker genes or with in situs. Then, "developmental paths" are determined on the basis of the clustering analysis , basically closeness of the clusters to each other, and identities are given for the clusters largely on the basis of the location within the developmental paths as well as a few marker genes. This is quite unconventional. Data is shown in Figure S13 for 24 genes, accompanied by a statement that "Transcript abundance of up-regulated genes in scRNA-seq results of vessel elements and ray parenchyma cells reveals fusiform and ray cell lineages. How it is possible to draw this conclusion remains unclear. I am not convinced either that the slight "intermixing" or the proposed cell types between the different clusters is enough proof for the claims that fibers can adopt different routes of differentiation, as proposed for instance in Fig. 1S or Fig. S17. We know on the basis of earlier studies that fiber differentiation is relatively synchronous throughout the circumference of the stem. Yet another controversial claim is that all fusiform cells are destined to vessel differentiation, but it is not really clear what is the proof for this claim. Furthermore, it is difficult to see the grounds for the conclusion on lines 165-167 "Our results suggest that the position of organizer cells in SDX (Fig. 1S) is similar to that of SAM organizer cells between stem cells/initials and xylem cells ". I propose the authors better back up their statements against existing literature, make clearer how their conclusions are supported by their data, and clarify which claims are purely hypothetical.

In addition to clusters of fibers, vessels and rays, clusters are called as fusiform organizers, fusiform early precursors, fusiform intermediate precursors, fusiform late precursors, ray organizers, and ray precursors in ways that are problematic. For the first, we don't know whether fibers or rays have organisers. This is demonstrated only for vessels, and only in Arabidopsis thaliana. Second, this nomenclature indicates that xylem differentiation is fully accomplished within the six layers of cells (which is the typical distance of cells where protoplasts are according to the authors derived with the used method). Also the use of the terms "non-terminal fiber lineage " or "terminal differentiated stages" is confusing . "Terminal differentiation" makes you think the fibers have terminated their differentiation program, including secondary cell wall formation, which is not the case for the cells isolated in this study. All in all, the authors present a confusing nomenclature which might lead an unacquainted reader to believe that this is an actual, well established sequence of xylem differentiation and that an accepted nomenclature exists for xylem differentiation in the presented manner. Of course it is easier to present the data by giving names for the clusters, but it is better to be clear that these are artificial/working names for the different clusters.

About the identities of the clusters

Clusters Ptr3 and Ptr6 are defined as "organiser cells" for ray and fusiform developmental fates. These are very big clusters which is surprising considering that there should be (on the basis of the Arabidopsis data for vessels) only one organiser in each cell file. The clusters Ptr3 and Ptr6 are given these identities on the basis of preferential expression of the III HD-ZIP transcription factors. How about other genes, like Monopteros that acts upstream of the class III HD-Zip factors? PXY, that should be expressed in the cambial cells, or perhaps towards the organiser, can be found in pretty much all of the clusters (Fig S27), making the identity of the clusters Ptr3 and Ptr6 as "organisers" uncertain.

The cluster Ptr1 is defined as "fusiform late precursor and vessel element". Even though the laser dissection supported the identity of the vessels in this cluster, I wonder if anyway also some fibers could reside in this cluster. Is it possible that some fibers are included in the samples obtained by laser dissection of the vessels (please see my comment below on additional details needed for the LCM method description)? The text refers only to the extensive expansion of the vessels, but also the fibers display substantial expansion, and not only radially but also longitudinally. And there is no reason to believe that the expansins, discussed as diagnostic for this cluster, would be specific to the vessel elements. Where elsewhere do the fibers undergoing expansion reside in the cluster analysis? Another problem here is that an impression is given in the manuscript that this cluster would contain mature vessels, but my question is why there is no apparent expression of the secondary cell wall biosynthetic genes in Ptr1? I find it necessary to clarify the fate of the maturing vessels in the clustering analysis.

Even more curious is the identity of the cells in the cluster Ptr7, which is defined as consisting mainly of fibers. The dominating feature is the high and specific expression of the secondary cell wall biosynthetic genes as well as cell death genes (XCP2) in the cells of this cluster. The secondary cell wall biosynthetic genes listed here are normally expressed both in vessel elements and fibers. And XCP2 might actually be vessel specific on the basis of data from Arabidopsis. It is therefore tempting to think that Ptr7 would represent (also?) vessel elements. Vessels normally differentiate earlier than fibers, and it does not seem likely that the expression of the cell wall biosynthetic genes would be derived among the SDX cell population only from the fibers. It is actually more likely that if there are cells in the SDX cell preparations (from the six first xylem cell layers) that have reached the stage of secondary cell wall formation and cell death they are likely to be vessel elements. It is not likely that fibers reach the stage of secondary cell wall formation or cell death within the six first rows of xylem differentiation. If Ptr7 represents only fibers, where do the vessel elements undergoing secondary cell wall formation and maturation reside in that case? The identify of Ptr7 as fibers is supported again by the laser dissected samples, but I wonder if it were possible that transcripts from maturing vessels could have ended up in the laser dissected fiber samples . This is actually supported by Figure 1J, but not discussed in the manuscript. The discrepancies with existing knowledge on differentiation of these cell types is so striking that more evidence from comparative analyses (with for instance the published scRNAseq data in Populus wood or published studies using laser dissection in the different cell types of Populus wood) is needed to prove the identities of the different clusters. For instance, information on expression of supposedly fiber-specific genes such as IRX15-L, ASPARTIC PROTEASE 66, and PtAP17 would be most helpful.

Interestingly, scRNA analysis of Trochodendron aralioides does not reveal a cluster that would correlate with the cluster of P. trichocarpa fiber cluster Ptr7. Considering that vessels evolved more recently than libriform fibers and that it is the presence/absence of vessels that varies between the basal angiosperms, it would be more likely that T. aralioides main xylem cell type is more closely related to fibers. This would again support the identity of the Ptr7 cells as vessels. Also, SND2 might not be the best marker for angiosperm cell lineages since SND2 homologs seem to exist also in the gymnosperms (https://doi.org/10.1111/nph.14458).

What are clusters Ptr9 and Ptr10. They look really interesting.

Minor details

The protoplasting method requires some more clarifications. The protoplasting was done during three hours which is a relatively long time compared to other reports even in the tree species. Information is needed on the effect of the protoplasting on the viability of the cells and preferably also on the transcriptome. Please include photographs on the isolated protoplasts preferably in all studied species. These images with included scale bars would give more information on the presence of the different cell types in the protoplast mixtures.

Also the LCM method needs more clarifications. How many cell layers were used to dissect the cells? In figure 1D it seems that more than six cell layers were used. Were any control experiments performed to exclude the possibility of heat , generated by the laser, influencing gene expression or success to isolate the expected cell types ? what was the number of cells / amount of RNA obtained from each cell type ? The comparison between the sc and lcm data (supplementary figure 8) identifies 28 shared DEGs between the two datasets, which is less than 1% of the DEGs in lcmRNAseq and less than 0,5% of all DEGs in Ptr1. Why is there so poor overlap ? Also, the laser dissection method resulted in P. trichocarpa to over 3500 DEGs in vessels and only around 300 in rays and fibers (additional file 14). What does this mean?

No information is given on formation (or removal) of doublets in the data derived from the 10x sorted cells. Doublet formation becomes an issue when targeting higher number of cells. No information exists either on correcting/removing the variation that could exist due to presence of cell cycle or mitochondrial/ chloroplast genes. This could lead to removing certain cells and provide a better picture of clustering.

Line 172 . is there more evidence for the rather controversial claim that "ray initials can elongate and convert into fusiform initials" ?

Lines 321-322. this notion is unnecessary as primary roots of 5-day old Arabidopsis seedlings don't have fibers.

Lines 485-492. The discusson on Sanyos four, and the "mother cells" is a bit outdated now that it seems that cambium has one initial which divides most frequently and that there does not seem to exist any "mother cells" . This comment concerns also nomenclature in Fig S16 .

Lines 591-594. Is the data for T. aralioides from the winter conditions included somewhere?

Figure 1 O-R please indicate the gene models in the figure legend. In general, it would be great to see annotations for genes presented in all figures.

Figure S2 should be updated. For instance the Populus publications are missing here.

**Authors Response**

**Point-by-point responses to the reviewers’ comments:**

**Reviewer #1**

Xylem development produces the largest plant biomass on the earth. This manuscript analyzed the formation and developmental dynamics of stem-differentiating xylem in four woody plants by using laser capture microdissection RNA-seq and single-cell RNA-seq (10x Genomics Chromium technology and fluorescence-activated cell sorting coupled with the plate-based MARS-seq method). The authors firstly focused on P. trichocarpa and identified the cell types of SDX, then compared the SDX cell types with other three angiosperms: E. grandis (core eudicot), T. aralioides (basal eudicot), and L. chinense (magnoliid). Based on the above data and analysis, the authors revealed the common and unique developmental trajectories for the fusiform and ray lineages. Finally, they examined the expression patterns of the known genes for xylem development, and laid special stress on analyzing the transcriptional expression and phylogenetic history for VNS family and SND2 and found their possible link to libriform fiber development in tracheid-bearing plants. Totally, this is a well-designed evo-devo study for xylem development study. Compared to previous scRNA-seq studies for xylem, this manuscript exhibits a higher resolution map to illustrate the developmental paths of xylem cells.

*[Author Response]*

*We thank the Reviewer for the valuable comments on our manuscript. We have*

*responded to each point raised by the Reviewer below.*

However, some following major and minor concerns should be answered.

Major concerns:

1, Because the authors identified the transcriptional profiling for libriform fiber, vessel element, and ray parenchyma by using the laser capture microdissection from the tissue sections, the cell identities for the three cell types of the scRNA-seq cell clusters in this study are convincible. While for the pseudo-time analysis for the cell populations, the development path seems to be only based on gene expression of scRNA-seq data. In principle, the start and end cell clusters and the direction of the trajectory should be analyzed aided by time-course RNA-seq. I strongly suggest that the authors performed bulked RNA-seq for the stem xylems at different developmental stages, such as the examples from different internodes. Current development trajectories were only based on the characterization of three terminal differentiated cell types, and thus the prediction for the path and developmental direction is more like a conjecture based on existing knowledge.

*[Author Response]*

*Based on the suggestions of Reviewer, we performed two types of transcriptomic analyses and anatomical dissections to provide time-course information on the different developmental stages of xylem development. We compared different developmental stages of xylem during vertical growth and horizontal growth:*

*(1) Time-course information on vertical growth.*

*As suggested by the Reviewer, we performed bulk RNA-seq for stem xylem from different internodes, and anatomical analyses were also performed for the examination of xylem developmental stages in P. trichocarpa. Our anatomical analyses found primary growth (bundle vasculature) above 4^th^ internode (from the top), transition zone between 4^th^ and 5^th^ internodes, and secondary growth below 5^th^ internode (please figure B below). The stems used in this study for protoplast isolation in P. trichocarpa were below 12^th^ internodes to ensure the xylem materials were all from secondary xylem. We collected the xylem samples from five sections of internodes to represent different developmental stages of in secondary xylem. Sections 1 to 5 include 6^th^ to 9^th^, 10^th^ to 13^th^, 14^th^ to 17^th^, 18^th^ to 21^th^ and 26^th^ to 29^th^ internodes, respectively (figure A). Six biological replicates were used for the xylem in every section for bulk RNA-seq (total 30 samples as 5 sections × 6 biological replicates = 30). In the principal component analysis results, the low percentage of PC1 suggests the absence of a dominant factor to differentiate the samples, which demonstrated that the percentage of the variation across different developmental internodes is subtle (figure C). Very few differentially expressed genes (DEGs) were detected, with only 12, 6, 11, 11, and 18 DEGs from sections 1 to 5, respectively (figure C). Many of these DEG functions are unknown, and the rest of the annotated DEG functions are basically unrelated to xylem development (please see Additional file 4 in the revised manuscript). Given that these DEGs only correspond to less than 0.1% of all expressed 23,441 genes in secondary xylem of these*

*five sections, the transcriptomic profiles from different internodes are nearly identical. These results suggested that xylem development is highly similar in different internodes with secondary growth, and that the developmental lineages discovered in our single-cell datasets are representative of the overall stem-differentiating xylem developmental trajectories.*

*Figure legends in the revised manuscript:*

*Fig. S23. Transcriptomic analyses of SDX across different internodes in P. trichocarpa. (A) Schematics of different stages in vertical growth in P. trichocarpa, including primary growth, transition zone and secondary growth. SDX from different sections, sections 1 to 5, were used for RNA-seq. (B) Anatomical analyses of SDX in different developmental stages. The cross-sections were observed under fluorescence microscope, and the blue signals represent deposited lignin. Red arrows indicated bundle vasculature. The yellow arrow showed the transition between bundle and circular vasculature. The green arrow indicated circular vasculature. Scale bars are 100 µm. (C) Principal component analysis and differential expression analyses were conducted using the SDX RNA-seq datasets from different internodes. Total 23,441 genes were expressed in secondary xylem, and 12/6/11/11/18 genes were differentially expressed in sections 1 to 5, respectively. XE, secondary xylem expressed genes. S1 to S5, sections 1 to 5.*

*We are working on depositing the newly generated sequencing raw datasets to NCBI GEO database, and the raw datasets are available in the following Dropbox link at current stage:*

[*www.dropbox.com/sh/7z2320jb60ltnvp/AAC9RecTbquG9BVwbJKdMfWCa?dl=0*](http://www.dropbox.com/sh/7z2320jb60ltnvp/AAC9RecTbquG9BVwbJKdMfWCa?dl=0)

*(2) Time-course information on horizontal growth.*

*Intensive cell wall deposition is a critical indication of xylem development. In other words, xylem development involves a process of thin-wall cells turning into thick-wall cells. We selected the gene sets from the GO terms for cell wall, cellulose and hemicellulose, and plotted the transcript abundance of these gene sets on the UMAP. The results showed an increasing enrichment of transcript abundance toward libriform fibers and vessel elements. Organizer cells are derived directly from vascular meristem. We also extracted the gene sets of GO term as meristem, and their transcript abundances were highly enriched in both organizer cell clusters. The enriched transcript abundance in the gene sets of meristem in organizer cells (the “start”) and the increasing transcript abundance toward libriform fibers and vessel elements in the gene sets of cell wall genes (the “end”) provides another layer of time-course evidence for horizontal xylem development.*

*Figure legends in the revised manuscript:*

*Fig. S14. Relative transcript abundance of cellulose-, hemicellulose-, and meristem-related genes. The GO terms for cellulose biosynthetic process, hemicellulose metabolic process, and meristem initiation are GO: 0030244, GO: 0010410, and GO: 0010014, respectively.*

*A time-course transcriptomic analysis on horizontal growth was performed in a previous study using a series of 20-µm tangential sections across the cambial and SDX regions (PMID: 15316113). We compared our results to the marker genes identified in the previous study, and found many genes with consistent expression pattern throughout SDX development (please see the figure below; the figure A below was a reproduction of the results in Fig. 6 and 7 from PMID: 15316113). In addition, HB8 is a member in HD-ZIP III family, which was used as the marker gene for organizer cell annotation in this study in P. trichocarpa and the organizer cell identification in a previous study in Arabidopsis (PMID: 30626967). The results in the previous time-course study (PMID: 15316113) supports the use of HD-ZIP III as organizer cell annotation in both P. trichocarpa and Arabidopsis.*

*Figure legends in the revised manuscript:*

*Fig. S14. Time-course transcriptomic analyses across cambial and SDX regions. (A) A schematic reproduction of gene expression results across the cambial and SDX regions from a previous study conducted using a series of 20-µm tangential sections. (B) The corresponding transcript abundance in the scRNA-seq results in this study.*

*Thus, the relative transcript abundance of the genes in the GO terms (cell wall and meristem) and the series sections across cambial/SDX regions both provide additional layers of time-course evidences of our results. We incorporated the description above into Results, and the corresponding methods were added in the Methods. Please see lines 185-202 (Results), 254-276 (Results), 783-791 (Methods), 796-797 (Methods) and 831-851 (Methods) in the revised manuscript.*

2, In the analysis of the cell cloud of UMAP, it is very far-fetched or can even be said to be completely wrong to judge the relationship between each two cell types just based on the "intermixing". During UMAP's dimensionality reduction process, such so-called "intermixing" actually is a prevalent problem. Although they are "intermixed" in 2D, they may be far away at third latitudes or higher. Giving biological meaning to explain the cell differentiation or transition based on just such a small amount of intermixed cells is a very risky proposition. In lines 172-174, actually no clear "intermixing" could be seen in my eyes based on your current figures. In lines 209-210, the conclusion is seriously flawed and might deviate from the factual basis. If the authors insisted on this, please provide more evidence.

*[Author Response]*

*We agree with the Reviewer that there is no direct evidence to support it. We removed the description related to “intermixing” (original lines 169-174), and moved the paragraph on cell fate (original lines 194-210) from Results to Discussion with revisions, including the removal of the conclusion drawn from “intermixing”.*

*The revised paragraph (lines 558-574 in the revised manuscript), which now reads: “It is still debated whether the cell fate of fusiform cells is determined at the very beginning in the initial cells or later during the differentiation of xylary derivatives [76]. Based*

*on the ubiquitous expression of VNS family genes in all early fusiform cells [77], it has been proposed that all fusiform cells originally differentiate toward vessel elements but become committed toward libriform fibers upon an external or positional cue [76]. As a response to mechanical stress, for example, tension wood contains drastically increased libriform fibers [36, 78]. In this study, the fusiform lineage in T. aralioides is similar to that in P. trichocarpa and E. grandis (Fig. 5J-L). The fusiform lineage in T. aralioides solely developed into tracheid, and both tracheids and vessel elements share highly similar transcriptomes. The results indicated that the fusiform lineages of P. trichocarpa and E. grandis undergo the same differentiation path as that of T. aralioides into vessel elements or tracheids, which supports the hypothesis from the previous studies. Interestingly, the libriform fibers (Ptr7) are the most disjoint cell cluster (Fig. 1B, Additional file 1: Fig. S18), implying the differentiation into libriform fibers took place rapidly, leaving few intermediate cells to be captured for scRNA-seq. Such disconnection in cell states has also been demonstrated for malaria parasite cell types, where an abrupt transcriptomic change is caused by fast turn-on/off of transcriptional modules [79]. Our results suggested that fusiform cells differentiate into vessel elements by default, but can rapidly switch to libriform fibers.”.*

3, It is worth noting that the authors attempt to conduct comparative single cell-transcriptomics studies for four plant species. It's a very meaningful attempt. However, in the selection of materials, no emphasis is placed on the selection of plant species with significant differences in xylem tissue development characteristics, for example, gymnosperms that lack xylem vessel elements and fibers can be considered for comparison. If possible, it would be a better option to introduce a gymnosperm to the study.

*[Author Response]*

*The four tree species were selected to represent evolutionary and SDX tissue diversity of angiosperms. We have revised the relevant paragraph (original lines 220-222) to better describe these species: “To further explore evolutionary conservation and divergence of SDX development in angiosperms, we performed scRNA-seq of SDX cells in three additional woody species, including E. grandis, T. aralioides (wheel tree) and L. chinense (Chinese tulip poplar). E. grandis also belongs to the same evolutionary clade as P. trichocarpa in core eudicots, and may possess similar xylem characteristics. L. chinense resides in magnoliids as an early diverging lineage, which allows the investigation of xylem development under a larger evolutionary scale. In contrast to the vast majority of flowering plants, T. aralioides possesses tracheids instead of vessel elements and libriform fibers ...”. Please see lines 278-283 in the revised manuscript. It would be indeed of great importance to perform scRNA-seq for the xylem cells in gymnosperms. Yet, a platform of protoplast isolation for scRNA-seq in a very different species would require tremendous time and effort, including the harvest of protoplast*

*with high viability rate, protoplast yield optimization and minimization of cell debris. For these reasons, adding a gymnosperm to our scRNA-seq analyses is practically impossible at the moment. This study focuses on angiosperms, and we hope to further investigate xylem cell development in gymnosperm in the future.*

Minor concerns:

1, What is the "DGE" for an individual cell group, such as one of that in line 231? Is that mean maximum expressed genes? Usually, DGE only means the differentially expressed genes between two samples, so it is not suitable to use it here.

*[Author Response]*

*DEG identification was performed following the pipeline in Cell Ranger of 10x Genomics platform. For each cell cluster, the DEGs were identified by treating the cells inside and the cells outside this cluster as two samples. Take the cell cluster Ptr1 as an example (Fig. 1B), each gene was tested whether its mean expression in Ptr1 differs from that in all other cell clusters.*

*We added the detailed description on the identification of differentially expressed genes into Methods. Please see lines 723-731 in the revised manuscript.*

2, For the materials, the authors use the different internodes of stems for different plants, what is the criterion for judging this? Is there a marker at the molecular level? These different internodes may be at different stages of development and may also have a significant impact on the results.

*[Author Response]*

*Different internodes were chosen in different species to ensure the xylem materials all from secondary growth.*

*As suggested by the Reviewer, we performed bulk RNA-seq for stem xylem from the 6th and lower internodes, which showed secondary growth based on anatomical examination of xylem developmental stages. There is little difference among the transcriptome profiles of different internodes (please see our response to Major Comment 1), suggesting that different internodes capture the development of SDX equally well.*

3, The quality of the pictures provided by this manuscript is too bad, which seriously affects the reviewer's understanding of the content.

*[Author Response]*

*We are sorry to know that the Reviewer did not receive high-resolution figures. We actually did upload high-resolution figures during our first submission, and the Editorial Office has confirmed the availability of our high-resolution figures for Editor and Reviewers. Please see the email below.*

*The following instruction confirmed by the Editorial Office may be helpful: “In the PDF file, there is a link on the top right for each figure to download the high-resolution version of the figures.”*

4, The results of the article need to be described in a more objective way, and not too literary, such as line327, "the tip of the iceberg".

*[Author Response]*

*We revised the sentence as follows: “only provide a partial understanding of xylem*

*transcriptomic variation.” Please see lines 386-387 in the revised manuscript.*

*We have also carefully gone through the manuscript and tried to improve the objectivity*

*of its text.*

5, The section "The reproducibility of the scRNA-seq results" should be adjusted to the front part or the method section. Current position looks a little strange.

*[Author Response]*

*We thank the Reviewer for this suggestion that improves the text flow. We have now moved the section "The reproducibility of the scRNA-seq results" to the Methods section. Please see lines 691-713 in the revised manuscript.*

**Reviewer #2**

The paper presents both single-cell and laser capture microdissection transcriptomic profiling of xylem cell types across four divergent angiosperm tree species. The paper is well structure and well written, and provide a clear leap in scope and understanding compared to previous work.

*[Author Response]*

*We thank the Reviewer for the valuable comments on our manuscript. For the ease of reading, we labeled the comments by numbers, such as (1), (2) and (3). We have responded to each point raised by the Reviewer below.*

Some aspects of the paper should be clarified:

(1) L38: «an evolutionarily reversed character». It is unclear to me what this means. Character -> trait?

*[Author Response]*

*We have changed “character” into “trait”. Please see lines 38 and 317 in the revised*

*manuscript.*

(2) L40: Why abbreviate "evo" and not "development"? "evo-devo" is commonly used as an expression.

*[Author Response]*

*We have revised “evo-development” into “evo-devo”. Please see lines 40 and 490 in*

*the revised manuscript.*

(3) L50-L56: Could you clarify how apical and lateral growth are linked in this paragraph too? Does the vascular cambium arise from the shoot apical meristem? That's what it sounds like, but you don't actually say it.

*[Author Response]*

*We have added the description as follows: “... horizontally into leaves or flowers and basally into stems, vascular cambium and vascular tissues.” Please see lines 52-53 in the revised manuscript.*

(4) L58: Is this the right use of the word "pluripotency"? Do you mean that the ability of a stem cell to differentiate into other cell types is maintained by organizer cells? Or that the type of cell differentiation is maintained by organizer cells?

*[Author Response]*

*Yes, it is correct. We use “pluripotency” to describe the ability of a stem cell/initial to differentiate into other cell types. We have provided more references to support this statement. The following sentences are extracted from the references:*

*[PMID: 26028217] “Stem cells in plants and animals are maintained pluripotent by signals from adjacent niche cells.”*

*[PMID: 23333352] “... by the transcription factor WUSCHEL, maintaining stem cell*

*pluripotency.”*

*[PMID: 31766002] “The SAM contains pluripotent stem cells, ...”*

*[PMID: 22404469] “the shoot meristem stem cells can be viewed as pluripotent.”*

*The stem cells/initials are maintained pluripotent by organizer cells based on the following referecnes:*

*[PMID: 24755933] “... stem cells are maintained by short-range signals that arise from specialized organizer cells ...”*

*[PMID: 23333352] “A small group of L3 cells, termed the organizing center (OC), maintains the stem cells undifferentiated by ...” The organizing center is called organizer cells in our study.*

*We added these references to support the statement of the sentence in the manuscript. Please see line 59 in the revised manuscript.*

(5) L131: It is unclear what "both these cell clusters" refer to since there are three clusters mentioned in the previous sentence. Also, "DEGs upregulated" compared to what? The other clusters?

*[Author Response]*

*“Both” was used to describe the transcriptomes in both “these cell cluster” and “their corresponding lcmRNA-seq cell type”. For a clearer description, we revised the sentence as follows: “... differentially expressed genes (DEGs) up-regulated in both transcriptomes of these cell clusters and their corresponding lcmRNA-seq cell type ...”. Please see lines 132-134 in the revised manuscript.*

*In cell clusters of scRNA-seq, “DEGs upregulated” means the DEGs upregulated in one cell cluster relative to all the other cell clusters, and the DEG identification pipeline was performed using Cell Ranger (10x Genomics platform). We now described in more detail on the identification of DEGs of cell clusters in Methods. Please see lines 723­731 in the revised manuscript.*

(6) L155-6: Confusing sentence. Ptr8 is not in the right part of the figure(?). It would be better to simply say that the other clusters are unidentified.

*[Author Response]*

*We revised the sentence, which now reads: “The three cell clusters Ptr1/7/8 at cell-type determined stages were annotated (Fig. 1B), while the rest of the cell clusters remained unidentified (Fig. 1B).” Please see lines 163-164 in the revised manuscript.*

(7) L240: Why would "a few master regulators" result in low correlations of homologous cell types?

*[Author Response]*

*The first scenario is that most expressed genes are not related to the developmental paths and show low correlations, but the expression of a few key regulatory genes can drive the organizer cells to develop into the same cell types in different species. The use of master regulators here might be confusing.*

*We have revised the sentence as follows: “First, most expressed genes are not related to the developmental paths and show low correlations. The SDX cell differentiation might be regulated by only a few key regulatory genes. Although most of gene expressions showed low correlations, the organizer cells can still develop into the same cell types under the control of these key regulators.” Please see lines 301-305 in the revised manuscript.*

(8) L240: It is unclear how correlation was computed across species. Few additional details are given in the method section (L711). To compute correlation between samples across species you need a set of common genes (or a representative gene from each orthogroup). Did you compute mean/sum expression across paralogs within each orthogroup? How many orthogroups was used to compute correlation?

*[Author Response]*

*To compute the cross-species correlation, we used the ortholog transcript abundance from a set of orthologous groups. The ortholog transcript abundance was calculated as the sum of gene transcript abundance within each orthologous group. Total 25,733 orthologous groups were used in the cross-species analyses. We added the description in Methods. Please see lines 869-872 and 875 in the revised manuscript.*

(9) Figure 4: It is not clear to me what the colors represent in this figure. Does red in A) mean mostly cells from trichocarpa compared to grandis?

*[Author Response]*

*As we mentioned in the legends of Fig. 4 “Densities from 0 to 1 are divided into 500 bins with different color shading ...”, the color represents the distribution density of cells. The red color in Fig. 4A represent high distribution density in that area.*

(0) L373: "the expression patterns ...were different ... due to their low expression". This does not make sense to me. Was it the patterns or the level that was different?

*[Author Response]*

*We revised the sentence as follows: “In L. chinense, the expression level of the orthologs of VND6, SND1, SND2, expansin A6 and hemicellulose biosynthesis genes were much lower than that in the three eudicots due to their low expression.” Please see lines 406-408 in the revised manuscript.*

(11) L432: "has been remained" -> "has remained"

*[Author Response]*

*We have revised the sentence as suggested. Please see line 464 in the revised*

*manuscript.*

(12) L435: "which took six-fold time more than that required". I don't understand the meaning of this sentence. Rewrite.

*[Author Response]*

*We revised the sentences as suggested, which now reads: “To avoid the contamination from the other two cell types, we carefully collected a very small area of ray parenchyma cells in each tangential section. Since extremely low amount of area were collectable for ray parenchyma cells in each section, tremendous labor and time was needed for the section preparation and cell collection. The time required for collecting enough ray parenchyma cells was six-fold more than that required for libriform fibers or vessel elements.” Please see lines 465-470 in the revised manuscript.*

(13) L504: "Two contradicted conclusions" -> "Two contradicting conclusions ".

*[Author Response]*

*We have revised the sentence as suggested. Please see line 543 in the revised*

*manuscript.*

**Reviewer #3**

This is a very elaborate analysis of gene expression on a single cell level in the woody tissues of Populus trichocarpa. It goes beyond the previously published scRNAseq analyses of protoplasts isolated from the woody tissues of tree stems in using laser dissection to validate the scRNAseq data, and in trying to utilise the P. trichocarpa scRNAseq data to compare xylem cell differentiation and functionalities between four different angiosperm tree species. The work seems to be done in a careful way. Indeed, the manuscript presents analyses of higher number of xylem cells, higher number of biological replicates, and higher number of different techniques for the sequencing (scRNAseq and MARSseq) than the previous studies in Arabidopsis or Populus vasculature. They also seem to have identified a significant mistake in an earlier scRNAseq analysis of Populus wood, which is valuable. I have however some concerns on a few parts of the manuscript. The most serious one concerns the clustering analysis of the Populus trichocarpa scRNAseq data and the conclusions drawn from this.

*[Author Response]*

*We thank the Reviewer for the valuable comments on our manuscript. For the ease of reading, we labeled the comments by numbers, such as Major Comment (1), (2) and (3). We have listed our point-by-point responses to all comments and suggestions below. In addition to the figures generated from our newly performed experiments and analyses, we also reused some figures from previous studies to provide clearer explanation. The reused figures would be used only in the response to Reviewers at the current stage.*

Major Comments

(1) Clustering of the *Populus trichocarpa* scRNAseq data 10 clusters are identified. The first question is why only one biological replicate (with only 4705 cells) was used for the clustering as altogether seven replicates were used for *P. trichocarpa*?

*[Author Response]*

*Through a very comprehensive survey on 68 tools for scRNA-seq analyses, a recent study (PMID: 34949812, Nature Methods, December 2021) benchmarking scRNA-seq data integration points out that “The integration results, however, often remove biological variation along with the batch effect, showcasing the aforementioned tradeoff between batch removal and bio-conservation.” In other words, more biological variation is likely to be removed from our analyses if different sequencing samples were integrated together. To avoid the elimination of biological variation, our main analyses of P. trichocarpa was based on one sample. We also showed that different samples had more than 99% overlapping rate (Fig. S35), and that bulk RNA-seq profiles have little difference between replications or internode sections (please see our response to Reviewer#1 Major Comment1). Our results demonstrated (i) the reproducibility among different biological replicates, (ii) using first biological replicate is sufficient to*

*represent the results from seven replicates, and (iii) focusing on one sample avoids the introduction of unwanted technical variation that can interfere with the detection of biological variation.*

(2) The clusters are given cellular identities on the basis of comparison with the laser dissected material. This is great even though it would not have harmed to validate the identities with reporter lines for some marker genes or with in situs.

*[Author Response]*

*We listed the following in situ hybridizations of different genes from previous studies, and these results provided another layer of evidences to support our conclusions from different perspectives. We summarized the figures from previous studies (please see the figure A-E below, and all scale bars in figure A-E are 25 µm).*

(i) *In the in situ hybridization results, the signal of ESK1a in poplar was highly enriched in libriform fibers (figure A) (PMID: 34809675), which is consistent to our scRNA-seq results (figure F). The results provided another layer of evidence, in situ hybridization, for the cell type annotation of libriform fibers.*
2. *In the original study, ABR1 was claimed to be expressed in ray parenchyma cells (PMID: 34809675). Due to their incorrect cell type annotation, the ray parenchyma cells in that previous study are supposed to be the organizer cells. The signal of ABR1 in poplar was enriched in the first several layers of outermost xylem area, which locate right next to vascular cambium (figure B). To provide a more precise assessment, we quantified the signal intensities from cambium to SDX (the selected yellow area in figure K, and the scale bars are 100 µm). An obvious enrichment could be observed on the outermost xylem area right next to cambium (figure K and L), and ABR1 was also enriched in Ptr3 and Ptr6 in the scRNA-seq results (figure G). Since we proposed Ptr3 and Ptr6 as organizer cells, the in situ hybridization evidence supports our conclusion that organizer cells are next to cambium.*
(i) *PdMYB156 and PdMYB221 transcription factors are known as the regulators for the development of libriform fibers and vessel elements (PMID: 26179205). The in situ hybridization results showed that these two MYBs were expressed in both libriform fiber and vessel element lineages (figure C and D) (PMID: 26179205). An enriched transcript abundance of PdMYB156 and PdMYB221 on the fusiform lineage in our study (figure H and I) further supports the roles of the two MYBs on the regulation of the development of libriform fibers and vessel elements.*

*In this previous study, the probe for PdMYB221 was also used for the detection of PdMYB156, so the figures for sense probes in PdMYB221 and PdMYB156 are the same.*

(ii) *APL was reported as a phloem marker gene supported by in situ hybridization (figure E). The nearly absence of APL expression supports the robustness of our protoplast isolation from SDX (figure J).*

*Figure legends:*

*(A-E) The in situ hybridization results from previous studies. (F-J) The scRNA-seq results in this study. (K) The area within yellow boxes were used for the quantification of the signal intensity. (L) The orange and grey lines represent the quantified signals of antisense and sense probes, respectively. Ve, vessel elements. F, libriform fibers. Ca, vascular cambium. Ph, phloem. Xy, xylem. Or, organizer cells.*

*We added a new paragraph in Results to describe the evidence from in situ hybridization as follows: “Our working hypothesis from scRNA-seq results were also supported by the in situ hybridization results from previous studies, including ESK1a for libriform fibers, ABR1 for organizer cell location, and PdMYB156/221 for fusiform lineage (Additional file 1: Fig. S17).” Please see lines 231-235 in the revised manuscript.*

(3) Then, "developmental paths" are determined on the basis of the clustering analysis, basically closeness of the clusters to each other, and identities are given for the clusters largely on the basis of the location within the developmental paths as well as a few marker genes. This is quite unconventional. Data is shown in Figure S13 for 24 genes, accompanied by a statement that "Transcript abundance of up-regulated genes in scRNA-seq results of vessel elements and ray parenchyma cells reveals fusiform and ray cell lineages. How it is possible to draw this conclusion remains unclear.

*[Author Response]*

*Since vessel elements and ray parenchyma cells belong to fusiform and ray lineages, respectively, the expressing patterns of the up-regulated genes in each cell type would indicate their lineages. To provide more precise evidence, we added the quantified results of these up-regulated genes in each lineage, including their transcript abundance*

*across the lineages (please see the figure A and D below) and average transcript abundance in each lineage (figure B and C) with bar charts (figure E and F).*

*In addition, two paralogous MYB transcription factors in poplar, PdMYB156 and PdMYB221, were reported as critical regulators on the development of vessel elements and libriform fibers (PMID: 26179205; PMID: 28117379). The in situ hybridization results showed that these two MYBs were highly expressed in both vessel element and libriform fiber lineage (PMID: 26179205). In our scRNA-seq results, the homologous genes of these two MYBs in P. trichocarpa showed enriched transcript abundances in the fusiform lineages (please see details in Major Comment 2). We also provided the quantified results to demonstrate that the transcript abundance of these two MYBs were enriched in fusiform lineage in the scRNA-seq results (the bottom panel in figure A to D; figure G). The enriched transcript abundance of up-regulated genes and known regulators both supported our proposed cell lineages. We added the description in the revised manuscript. Please see lines 206-209 (Results), 213-220 (Results) and 853-859 (Methods) in the revised manuscript.*

*Figure legends in the revised manuscript:*

*Fig. S16. Transcript abundance of up-regulated genes in each cell type across fusiform and ray lineages. (A) Transcript abundance of the up-regulated genes across ray lineage. (B) The normalized mean expression in ray lineage. (C) The normalized mean expression in fusiform lineage. (D) Transcript abundance of the up-regulated genes across fusiform lineage. (E) to (F) Bar charts represent the normalized mean expression of the up-regulated genes and the homologs of PdMYB156 and PdMYB221 in P. trichocarpa of fusiform lineage and ray lineage, respectively. Normalized mean expression was calculated as the mean UMI counts of each gene normalized with mean UMI counts of all genes. RM, normalized mean expression in ray lineage. FM, normalized mean expression in fusiform lineage.*

(4) I am not convinced either that the slight "intermixing" or the proposed cell types between the different clusters is enough proof for the claims that fibers can adopt different routes of differentiation, as proposed for instance in Fig. 1S or Fig. S17. We know on the basis of earlier studies that fiber differentiation is relatively synchronous throughout the circumference of the stem. Yet another controversial claim is that all fusiform cells are destined to vessel differentiation, but it is not really clear what is the proof for this claim.

*[Author Response]*

*We agree with the Reviewer that there is no direct evidence to support it. We removed the description related to “intermixing” (original lines 169-174), and moved the paragraph on cell fate (original lines 194-210) from Results to Discussion with revisions, including the removal of the conclusion drawn from “intermixing”.*

*The revised paragraph (lines 558-574 in the revised manuscript), which now reads: “It is still debated whether the cell fate of fusiform cells is determined at the very beginning in the initial cells or later during the differentiation of xylary derivatives [76]. Based on the ubiquitous expression of VNS family genes in all early fusiform cells [77], it has been proposed that all fusiform cells originally differentiate toward vessel elements but become committed toward libriform fibers upon an external or positional cue [76]. As a response to mechanical stress, for example, tension wood contains drastically increased libriform fibers [36, 78]. In this study, the fusiform lineage in T. aralioides is similar to that in P. trichocarpa and E. grandis (Fig. 5J-L). The fusiform lineage in T. aralioides solely developed into tracheid, and both tracheids and vessel elements share highly similar transcriptomes. The results indicated that the fusiform lineages of P. trichocarpa and E. grandis undergo the same differentiation path as that of T. aralioides into vessel elements or tracheids, which supports the hypothesis from the previous studies. Interestingly, the libriform fibers (Ptr7) are the most disjoint cell cluster (Fig. 1B, Additional file 1: Fig. S18), implying the differentiation into libriform fibers took place rapidly, leaving few intermediate cells to be captured for scRNA-seq. Such*

*disconnection in cell states has also been demonstrated for malaria parasite cell types, where an abrupt transcriptomic change is caused by fast turn-on/off of transcriptional modules [79]. Our results suggested that fusiform cells differentiate into vessel elements by default, but can rapidly switch to libriform fibers.”.*

(5) Furthermore, it is difficult to see the grounds for the conclusion on lines 165-167 "Our results suggest that the position of organizer cells in SDX (Fig. 1S) is similar to that of SAM organizer cells between stem cells/initials and xylem cells ".

*[Author Response]*

*During protoplast isolation, the tree bark was peeled off from the stems. The breakage happened in the area between differentiating xylem and cambium (PMID: 25144270). Namely, phloem and initials were on the bark side, and organizer cells and SDX remained on the surface of the stems. With the protoplast isolation, several layers on the debarked stem surface were released. In our study, we proposed the starting layers on the debarked stem are organizer cells, leading to the scenario that initials differentiate into organizer cells then into differentiating xylem cells. In SAM and vascular cambium in Arabidopsis, organizer cells locate between stem cells and xylem cells, which is similar to our hypothetical developmental paths of stem xylem.*

*The original lines 165-167 have been revised with clearer explanation: “During protoplast isolation, the tree bark was peeled off from the stems and removed. The breakage happened in the area between SDX and cambium [38]. Namely, phloem and initials were on the bark side, and organizer cells and SDX remained on the surface of the stems. We detected none or extremely low expression of ANT (a stem cell/initial cell marker gene [34]; Fig. 1P, Additional file 1: Fig. S13B) or PEAR1 (a phloem marker gene [34]; Additional file 1: Fig. S13C) in any of the cell clusters, as expected from the removal of initials and phloem during the debarking step of protoplast preparation [38] (Fig. 1A). The results led to a highly possible developing path as initials differentiating into organizer cells then into xylem cells, and the position of organizer cells, between stem cells/initials and xylem cells (Fig. 1S), is similar to that in SAM and vascular cambium [2, 14].” Please see lines 173-183 in the revised manuscript.*

(6) I propose the authors better back up their statements against existing literature, make clearer how their conclusions are supported by their data, and clarify which claims are purely hypothetical.

*[Author Response]*

*We revised the descriptions of cell differentiation and cell cluster nomenclatures. For the parts without direct evidence supports, we clarified these descriptions as our hypotheses and moved to Discussion. Please see our responses to Major Comments 4, 7-1, 7-2, 7-3, 8 and 18.*

(7) In addition to clusters of fibers, vessels and rays, clusters are called as fusiform organizers, fusiform early precursors, fusiform intermediate precursors, fusiform late precursors, ray organizers, and ray precursors in ways that are problematic.

(7-1) For the first, we don't know whether fibers or rays have organisers. This is demonstrated only for vessels, and only in Arabidopsis thaliana.

*[Author Response]*

*We added the following description to clarify that the existence of organizer cells for libriform fiber and ray parenchyma remained hypothetical but highly possible: “In Arabidopsis, organizer cells were found in the vessel element differentiation, but whether the differentiation of libriform fibers and ray parenchyma cells involve organizer cells remained unknown. HD-ZIP III played a critical role in organizer cells (REF) in Arabidopsis, and was used as a marker gene for organizer cell annotation in P. trichocarpa in this study. Besides the regulation of vessel element differentiation, previous studies in Arabidopsis and poplar have shown that HD-ZIP III can also accelerate the differentiation of libriform fibers (PMID: 11402194; PMID: 23288865), suggesting the existence of organizer cells in the cell types beyond vessel elements.” Please see lines 535-541 in the revised manuscript.*

(7-2) Second, this nomenclature indicates that xylem differentiation is fully accomplished within the six layers of cells (which is the typical distance of cells where protoplasts are according to the authors derived with the used method). Also the use of the terms "non-terminal fiber lineage" or "terminal differentiated stages" is confusing. "Terminal differentiation" makes you think the fibers have terminated their differentiation program, including secondary cell wall formation, which is not the case for the cells isolated in this study.

*[Author Response]*

*“Terminal differentiated stage” was revised into “cell-type determined stage” to indicate that cell types were already determined at these stages. “Non-terminal fusiform lineage” was changed into “cell-type undetermined lineage” to indicate the cell type remained undetermined. Please see lines 163, 238, 380 and 1130 in the revised manuscript.*

*We have additionally clarified the nomenclature of libriform fibers, vessel elements and ray parenchyma cells as follows: “Since the terminal stage of xylem cell differentiation represents the dead xylem cells, it would be impossible to profile the transcriptome of “mature” xylem cells. The annotated libriform fibers, vessel elements and ray parenchyma cells represent the cells with determined cell types and under differentiation toward the mature stage.” Please see lines 154-157 in the revised manuscript.*

(7-3) All in all, the authors present a confusing nomenclature which might lead an unacquainted reader to believe that this is an actual, well established sequence of xylem differentiation and that an accepted nomenclature exists for xylem differentiation in the presented manner. Of course it is easier to present the data by giving names for the clusters, but it is better to be clear that these are artificial/working names for the different clusters.

*[Author Response]*

*We added a description in Discussion to clarify the nomenclature of cell clusters, which now reads: “Cell differentiation is a continuous process, and cell clustering methods divide cells into discrete clusters based on their transcriptomic profiles. The nomenclature of cell clusters in this study represents our working hypothesis for SDX developmental paths and was adopted to facilitate our discussion of the cell clusters.” Please see lines 227-231 in the revised manuscript.*

About the identities of the clusters

(8) Clusters Ptr3 and Ptr6 are defined as "organiser cells" for ray and fusiform developmental fates. These are very big clusters which is surprising considering that there should be (on the basis of the Arabidopsis data for vessels) only one organiser in each cell file.

*[Author Response]*

*We added a description to clarify the nomenclature of Ptr3 and Ptr6 (Fig. 1B) as follows: “The nomenclature of each cell cluster basically represents three groups of cells: (i) cells of such cell type, (ii) cells under the differentiation toward such cell type, (iii) cells under the differentiation away from such cell type. For example, Ptr6 as fusiform organizer cells would contain three groups of cells: the organizer cells, the cells under the differentiation from initials into fusiform organizer cells, and the organizer cells already under differentiation toward differentiating xylem.” Please see lines 222-227 in the revised manuscript.*

(9) The clusters Ptr3 and Ptr6 are given these identities on the basis of preferential expression of the III HD-ZIP transcription factors. How about other genes, like Monopteros that acts upstream of the class III HD-Zip factors? PXY, that should be expressed in the cambial cells, or perhaps towards the organiser, can be found in pretty much all of the clusters (Fig S27), making the identity of the clusters Ptr3 and Ptr6 as "organisers" uncertain.

*[Author Response]*

*Monopteros showed low transcript abundance in many cell clusters. As we mentioned in the manuscript that not all marker genes in Arabidopsis can be used to explain our proposed cell lineages in other species. In this study, we used HD-ZIP III as the*

*marker gene for cell type annotation of organizer cells, but its gene expression pattern is not conserved between P. trichocarpa and other species in this study. The results raised an issue: Is HD-ZIP III a robust marker gene for the identification of organizer cells? If the answer is yes, then organizer cells would not exist in all species due to the nearly absence of HD-ZIP III expression in some species (Fig. S33A). If the answer is no, then it brings up the next issue: Whether all species in this study possess organizer cells? If organizer cells are a common cell type, then different species would use varies mechanisms, without HD-ZIP III, for the production of organizer cells. If organizer cells do not exist in certain species, the initials would require other cell types or other mechanisms for the maintenance. Based on the current knowledge, SAM, RAM and vascular cambium in Arabidopsis all require organizer cells for the maintenance of stem cells/initials, the existence of organizer cells in all of the species in this study appears to be highly possible. HD-ZIP III, instead, seems not necessary for organizer cells in every species.*

*During protoplast isolation, debarking led to the breakage between differentiating xylem and cambium. Since organizer cells were found to locate right next to vascular cambium, organizer cells are supposed to be the cells with least differentiating level in the cells isolated from debarked stems. Ptr3 and Ptr6 locate at the farthest position of differentiating libriform fibers/vessel elements/ray parenchyma cells, suggesting the identity of Ptr3 and Ptr6 as organizer cells.*

*We incorporated the description above into Results and Discussion, please see lines 169-173 (Results) and 521-533 (Discussion) in the revised manuscript.*

(10) The cluster Ptr1 is defined as "fusiform late precursor and vessel element". Even though the laser dissection supported the identity of the vessels in this cluster, I wonder if anyway also some fibers could reside in this cluster. Is it possible that some fibers are included in the samples obtained by laser dissection of the vessels (please see my comment below on additional details needed for the LCM method description)?

*[Author Response]*

*We avoided the sample contamination throughout the whole process of laser capture microdissection. Based on the following reasons, we are confident that the possibility of sample contamination is extremely low. Even if a tiny amount of cross-contamination occurred, such low amount of contamination would be nearly impossible to affect the transcriptomic profile of our targeted cell type.*

*(i) Issue of cell visualization. During cell collection, we visualized and selected our targeting area, which contained the cell type of interest. For a sample section, one side can be visualized, and the other side contacted the slide, which cannot be visualized. When laser cut though the selected area on a section, we collected both visualizable and unvisualizable regions. To avoid the collection of unwanted cells, the thickness of sections should contain only one layer of cells, or should be even less than the length*

*or width of one layer of cells, which can thus avoid the unwanted cells hiding in the unvisualizable regions.*

*We set the thickness of cross- and tangential-sections as 16 µm. For cross-sections of libriform fiber collection, the thickness is much less than the cell length of libriform fiber (500-750 µm) (PMID: 17403651). For tangential-sections of vessel element collection, the thickness is also much less than the cell diameter of vessel elements (34­48 µm) (PMID: 17403651).*

*(ii) Issue of residual fragments on slides. Laser cutting is actually a process of burning out the cells by laser on the selected paths. While burning out the cells, laser cutting also generates tiny fragments. These fragments would either fall down into the collecting caps along with the targeting cell area, or remained on the slides. The fragments remained on the slides may fall down and get collected in the next rounds of cutting. Thus, each round of cutting would result in the collection of targeting cell area and the fragments generated on such round or previous rounds. As a result, each slide was only used for the collection on one cell type to avoid cross contamination of cell types caused by the collection of unwanted residual fragments.*

*To obtain robust and reliable results, all of the criteria had to be strictly fulfilled throughout the cell collection, including the section thickness setting, exclusion of the unwanted residual fragments, and all of the other techniques mentioned in the manuscript. These time-consuming and delicate-labor-intensive tasks led to a gigantic obstacle for the success of transcriptomic comparison among three xylem cell types, which may explain the reason that none of the previous studies accomplish this critical and necessary goal.*

*We added the description above into Methods. Please see lines 762-781 in the revised manuscript.*

(11) The text refers only to the extensive expansion of the vessels, but also the fibers display substantial expansion, and not only radially but also longitudinally. And there is no reason to believe that the expansins, discussed as diagnostic for this cluster, would be specific to the vessel elements. Where elsewhere do the fibers undergoing expansion reside in the cluster analysis?

*[Author Response]*

*In Fig. S9O, both libriform fibers (Ptr7) and vessel elements (Ptr1) expressed the expansin gene, while a significant enrichment of transcript abundance in vessel elements (Ptr1). The results demonstrated that both libriform fibers and vessel elements undergo cell expansion, and the cell expansion is more intensive in vessel elements, which is consistent to the anatomical observation in previous studies. In poplar, vessel elements possess around 4.36 times of volume comparing to libriform fibers (please see the detailed calculation below). Since vessels possess much larger cell volume than libriform fibers, more extensive cell expansion would be conducted in vessels, which*

*can explain the enriched transcript abundance in vessels. Previous studies also reported that vessel elements undergo more complex cell expansion, symplastic and intrusive growth, than libriform fibers to yield larger cell diameter (PMID: 11554475; PMID: 18065553).*

*Volume calculation: In poplar in early summer, the cross-sectional area of a libriform fiber is around 100-275 µm^2^ with the length around 500-750 µm. The cross-sectional area of a vessel element is around 900-1800 µm^2^ with the length as 380-500 µm (PMID: 17403651). For a simpler calculation, we take the shape of both libriform fibers and vessel elements as cylinder, then their volume would be as following:*

*Libriform fiber: The maximum volume would be 275×750=206,250 µm^3^*

*Vessel element: The maximum volume would be 1800×500= 900,000 µm^3^ 900,000/206,250 = 4.36 folds.*

(12) Another problem here is that an impression is given in the manuscript that this cluster would contain mature vessels, but my question is why there is no apparent expression of the secondary cell wall biosynthetic genes in Ptr1? I find it necessary to clarify the fate of the maturing vessels in the clustering analysis.

*[Author Response]*

*According to the revision suggested by the Reviewer in Major Comment 8-2, we revised “mature vessel elements” into “cell-type determined vessel elements”. Both libriform fibers and vessel elements expressed secondary cell wall biosynthetic genes, while these genes were strongly enriched in libriform fibers. We selected the gene sets from the GO terms for cell wall, cellulose and hemicellulose, and plotted the transcript abundance of these gene sets on the UMAP. The results showed an increasing enrichment of transcript abundance toward libriform fibers and vessel elements, and vessel elements showed obvious enriched expression of secondary cell wall biosynthetic genes than the cells with relative thinner wall, such as organizer cells. To put it simply, vessel elements also undergo secondary cell wall thickening but not as intensive as libriform fibers. The results were consistent to the anatomical observation on the cell wall thickness in previous studies, which reported that the cell wall of libriform fibers is two times thicker than that of vessel elements (PMID: 28541580;* [*https://doi.org/10.1111/jse.12595).*](https://doi.org/10.1111/jse.12595).)

*Figure legends:*

*Relative transcript abundance of cellulose- and hemicellulose-related genes. The GO terms for cellulose biosynthetic process and hemicellulose metabolic process are GO: 0030244 and GO: 0010410, respectively.*

(13) Even more curious is the identity of the cells in the cluster Ptr7, which is defined as consisting mainly of fibers. The dominating feature is the high and specific expression of the secondary cell wall biosynthetic genes as well as cell death genes (XCP2) in the cells of this cluster. The secondary cell wall biosynthetic genes listed here are normally expressed both in vessel elements and fibers. And XCP2 might actually be vessel specific on the basis of data from Arabidopsis. It is therefore tempting to think that Ptr7 would represent (also?) vessel elements.

*[Author Response]*

*XCP2 is indeed specifically expressed in vessel in Arabidopsis, and is regulated by the vessel-specific master regulator, VND. However, VND family express in both vessel elements and libriform fibers in P. trichocarpa (PMID: 29078399), which can explain our detection of XCP2 in libriform fibers.*

*In this study, XCP2 is enriched in libriform fibers in the scRNAseq results, but is enriched in vessel elements in the lcmRNAseq results. This inconsistency, again, demonstrates the risk of cell type annotation using few known marker genes, and also shows the necessity of the use of in situ transcriptomic data (lcmRNAseq) for cell type annotation, which was also emphasized in the Discussion. In our manuscript, XCP2 was only used as a xylem marker.*

(14) Vessels normally differentiate earlier than fibers, and it does not seem likely that the expression of the cell wall biosynthetic genes would be derived among the SDX cell population only from the fibers. It is actually more likely that if there are cells in the SDX cell preparations (from the six first xylem cell layers) that have reached the stage of secondary cell wall formation and cell death they are likely to be vessel elements. It is not likely that fibers reach the stage of secondary cell wall formation or cell death within the six first rows of xylem differentiation. If Ptr7 represents only fibers, where do the vessel elements undergoing secondary cell wall formation and maturation reside in that case?

*[Author Response]*

*As the response to Major Comment 13, both vessel elements (Ptr1) and libriform fibers (Ptr7) expressed secondary cell wall biosynthetic genes, while an intensive enrichment of these gene expression was observed libriform fibers, which can explain the two times thicker cell wall in libriform fibers than vessel elements. Please see the details in Major Comment 13.*

(15) The identify of Ptr7 as fibers is supported again by the laser dissected samples, but I wonder if it were possible that transcripts from maturing vessels could have ended up in the laser dissected fiber samples. This is actually supported by Figure 1J, but not discussed in the manuscript. The discrepancies with existing knowledge on differentiation of these cell types is so striking that more evidence from comparative analyses (with for instance the published scRNAseq data in Populus wood or published studies using laser dissection in the different cell types of Populus wood) is needed to prove the identities of the different clusters.

*[Author Response]*

*We downloaded the lcmRNAseq results for libriform fibers and vessel elements from previous studies (PMID: 29078399; PMID: 28083709). In these studies, vessel elements were collected from stem cross-sections instead of tangential sections in this study. The lcmRNAseq from the previous studies and this study show highly consistent results (please see the figure below). In other words, the transcriptomes of vessel elements collected from stem cross-sections (PMID: 29078399; PMID: 28083709) and tangential sections (from this study) lead to the same cell type annotation. The results demonstrate the reproducibility of our experiments, and low possibility of contamination during LCM cell collection.*

*Figure legends:*

*Upper panel, the correlation between scRNA-seq results (this study) and the lcmRNA-seq results (this study) of three cell types, libriform fibers, vessel elements, and ray parenchyma cells. Lower panel, the correlation between scRNA-seq results (this study) and the lcmRNA-seq results (PMID: 29078399; PMID: 28083709) of two cell types, libriform fibers and vessel elements.*

(16) For instance, information on expression of supposedly fiber-specific genes such as IRX15-L, ASPARTIC PROTEASE 66, and PtAP17 would be most helpful.

*[Author Response]*

*The transcript abundance of these three genes were also analyzed in the previous SDX scRNA-seq study in P. alba (PMID: 34809675). We first identified the homologs of P. alba IRX15-L (PdDUF579-9), ASPARTIC PROTEASE 66 (PtAP66), and PtAP17 in P. trichocarpa as Potri.005G141300, Potri.019G002100 and Potri.004G007600, respectively. We then examined the transcript abundance of these three genes on the UMAP of scRNA-seq results of P. trichocarpa, and the expression patterns are highly consistent to that in P. alba. IRX15-L (PdDUF579-9)/Potri.005G141300 and ASPARTIC PROTEASE 66 (PtAP66)/Potri.019G002100 are exclusively expressed in libriform fibers (please see the figure below). PtAP17/Potri.004G007600 expressed in both organizer cells and libriform fibers.*

*Figure legends:*

*In each gene, left panels are the results from the previous study (PMID: 34809675), and*

*the right panels are the results in this study.*

*We incorporated the following description in Results: “The exclusive expression of previously reported libriform fiber marker genes, IRX15-L and ASPARTIC PROTEASE 66, in the libriform fiber cell cluster in our scRNA-seq provide further evidence for the cell type annotation in our study.” Please see lines 143-146 in the revised manuscript.*

(17) Interestingly, scRNA analysis of Trochodendron aralioides does not reveal a cluster that would correlate with the cluster of P. trichocarpa fiber cluster Ptr7. Considering that vessels evolved more recently than libriform fibers and that it is the presence/absence of vessels that varies between the basal angiosperms, it would be more likely that T. aralioides main xylem cell type is more closely related to fibers. This would again support the identity of the Ptr7 cells as vessels.

*[Author Response]*

*Many studies have indicated the co-evolution of vessel elements and libriform fibers:*

(i) *Vessel elements and libriform fibers in angiosperms appeared at a similar time, around Cretaceous (please see Reference#1 and #2 below).*
(ii) *The co-evolution of libriform fibers compensate for the mechanical weakness from*

*vessel elements (Reference#2 and #3).*

(iii) *The structural changes in vessel elements were closely synchronized with that in libriform fibers (Reference#4).*
(iv) *A higher proportion of libriform fibers was observed in the species containing more*

*specialized vessel elements (Reference#5).*

*A previous study reported that the tracheids in T. aralioides is similar to vessel elements*

*due to the absence of plasmodesmata from pit membrane in both cell types. In contrast,*

*libriform fibers possess plasmodesmata in pit membrane (Reference#6). Thus, the main*

*xylem cell type in T. aralioides should be more closely related to vessel elements other*

*than libriform fibers based on the anatomical observation from the previous study and*

*the evidence discovered in this study.*

*Reference#1:* [*https://doi.org/10.1086/368398*](https://doi.org/10.1086/368398)

*Reference#2:* [*https://doi.org/10.1016/S0065-2296(08)60361-4*](https://doi.org/10.1016/S0065-2296(08)60361-4)

*Reference#3:* [*https://doi.org/10.1016/B978-012339552-8/50016-0*](https://doi.org/10.1016/B978-012339552-8/50016-0)

*Reference#4:* [*https://doi.org/10.2307/2438485*](https://doi.org/10.2307/2438485)

*Reference#5:* [*https://doi.org/10.1525/9780520320567*](https://doi.org/10.1525/9780520320567)

*Reference#6:* [*https://doi.org/10.1007/BF00392724*](https://doi.org/10.1007/BF00392724)

(18) Also, SND2 might not be the best marker for angiosperm cell lineages since SND2 homologs seem to exist also in the gymnosperms [(](https://doi.org/10.1111/nph.14458)https://doi.org/10.1111/nph.14458).

*[Author Response]*

*We toned down our description as follows: “The absence of SND2 genes in many gymnosperms and seedless vascular plants is thus consistent with their lack of libriform fibers (Fig. 6A). Intriguingly, T. aralioides, which also lacks libriform fibers, does possess SND2 genes (Fig. 6A, 6C), but their expression was not detected in SDX cells (Fig. 6D). Namely, the function of SND2 could be turned off by the absence of this gene or its expression.” Please see lines 430-434 in the revised manuscript.*

(19) What are clusters Ptr9 and Ptr10. They look really interesting.

*[Author Response]*

*Ptr9 and Ptr10 seems to be cell clusters related to vessel elements (Fig. 1B), because they located near vessel elements on UMAP. Since no apparent or related evidence for Ptr9 and Ptr10, we would like not to annotate the cell types for these two cell clusters.*

Minor details

(1) The protoplasting method requires some more clarifications. The protoplasting was done during three hours which is a relatively long time compared to other reports even in the tree species. Information is needed on the effect of the protoplasting on the viability of the cells and preferably also on the transcriptome.

*[Author Response]*

*We summarized the cell wall digestion time for SDX protoplast release in three previous studies and this study (please see the figure below). Although the digestion time differed from 0.3-3 hours, all digestion time showed the release of 6-10 layers. Since the digestion enzymes cannot digest lignin in cell wall, the cell wall of the cells beyond 10 layers may possess high contents of lignin, which would not be digestible. We used 3-hour digestion time in this study to reach a more uniform digestion for the protoplast release. For example, the digestion would be uneven if 3 layers of SDX in some area were released, while other area released 6 layers. Uneven digestion for protoplast isolation would lead to an enrichment of cells toward the surface/cambial area, which would cause unexpected bias. A longer digestion time would provide more even digestion to avoid unexpected enrichment of certain cells.*

*In our previous study, we used trypan blue dye exclusion test to demonstrate that ~98% of the protoplasts are viable after 0.3-hour or even up to 3-hour enzyme digestion (PMID: 24280390).*

*Figure legends:*

*The protoplast isolation under different enzyme digestion times from 0.3 to 3 hours. In the left panels of each study, the orange circles indicate the cell layers.*

*Please note that Chen et al may mark the scale bars incorrectly. The scale bars as 5 µm would cause the diameter of libriform fibers into a range around 1 µm, which is inconsistent to the cell size reported from previous studies.*

(2) Please include photographs on the isolated protoplasts preferably in all studied species. These images with included scale bars would give more information on the presence of the different cell types in the protoplast mixtures.

*[Author Response]*

*We incorporated the photographs of the SDX protoplasts from each species in this study*

*to Methods (please see the figure below).*

*Please see Fig. S34 and the related descriptions in lines 618-619 in the revised*

*manuscript as follows: “The isolated SDX protoplasts were observed using fluorescence microscope (Fig. S34).”*

*Figure legends in the revised manuscript:*

*Fig. S34. The SDX protoplasts were observed using fluorescence microscope. Upper panel, the morphology of SDX protoplasts from each species in bright field. Lower panel, the SDX protoplasts with FDA staining. Scale bars are 50 µm.*

(3) Also the LCM method needs more clarifications.

(3-1) How many cell layers were used to dissect the cells? In figure 1D it seems that

more than six cell layers were used.

*[Author Response]*

*We collected around 20-layer cells, which took us more than one year on the LCM cell collection. There are too few cells within the six outermost cell layers, and it would require much more time for the cell collection.*

*We added the information of cell layer in Methods as follows: “Around 1,600,000 µm^2^ area from approximate 20 cell layers of cryo-sections (transverse or tangential) ...”. Please see lines 748-749 in the revised manuscript.*

(3-2) Were any control experiments performed to exclude the possibility of heat, generated by the laser, influencing gene expression or success to isolate the expected cell types?

*[Author Response]*

*To prevent tissue or RNA degradation, the cross- and tangential-sections were already fixed and dehydrated by ethanol. Besides ethanol fixation, the sections were produced at -20^o^C to avoid the perturbation of the transcriptomes. Laser itself might cause damage to RNA, and may lead to certain degree of RNA degradation, but we doubt it would cause systematic bias of RNA profiles.*

(3-3) What was the number of cells / amount of RNA obtained from each cell type?

*[Author Response]*

*We collected same volume of cells for each cell type (thickness as 16 µm; area as*

*1,600,000 µm^2^). The RNA amount from these cells was too low to be detected and*

*quantified. The following is the calculation for the estimated collected cell numbers:*

*Collected volume: 16 µm ×1,600,000 µm^2^ = 25,600,000 µm^3^*

*The volume of one libriform fiber: 206,250 µm^3^ (please see details in Major Comment*

*11). The volume of one vessel element: 900,000 µm^3^ (please see details in Major*

*Comment 11). The volume of one ray parenchyma cell: 314 µm (length; please see*

*Reference#1 below)×60 µm^2^ (area; Reference#2)= 18,840 µm^3^.*

*The estimated collected cell number:*

*Libriform fiber: 25,600,000/206,250 = ~124*

*Vessel element: 25,600,000/900,000 = ~28*

*Ray parenchyma cell: 25,600,000/18,840 = ~1359*

*Reference#1:* [*https://doi.org/10.1007/s00226-016-0860-y*](https://doi.org/10.1007/s00226-016-0860-y) *Reference#2:* [*https://doi.org/10.1007/s10086-010-1168-4*](https://doi.org/10.1007/s10086-010-1168-4)

(4) The comparison between the sc and lcm data (supplementary figure 8) identifies 28 shared DEGs between the two datasets, which is less than 1% of the DEGs in lcmRNAseq and less than 0.5% of all DEGs in Ptr1. Why is there so poor overlap?

*[Author Response]*

*The difference between the two sample and library preparation methods (lcmRNA-seq and scRNA-seq) is still not neglectable, but lcmRNA-seq is still a very effective method for cell type annotation in scRNA-seq results. For example, the correlation between vessel elements in lcmRNA-seq and Ptr1 in scRNA-seq is around 0.44 (Fig. 1J), while the correlations between vessel elements in lcmRNA-seq and other cell clusters in scRNA-seq are much lower or even almost 0 in some clusters. To ensure the marker genes were reliable, we thus only selected the DEGs with highly consistent expression pattern in both platforms, which is a relatively stringent criterion.*

(5) Also, the laser dissection method resulted in P. trichocarpa to over 3500 DEGs in vessels and only around 300 in rays and fibers (additional file 14). What does this mean?

*[Author Response]*

*The expressed gene numbers in vessel elements from lcmRNA-seq results are much lower than that in libriform fibers and ray parenchyma cells (please see below for the details). Among the 3552 DEGs in vessel elements, 113 were up-regulated and 3439 were down-regulated. Among the 3439 down-regulated DEGs, 2998 of them exhibit no expression in vessel elements, which suggested that vessel elements expressed much less genes than libriform fibers and ray parenchyma cells.*

*Detected gene number:*

*Vessel element bio#1/2/3: 4306/6634/11417 genes*

*Vessel element bio#1+2+3: 14237 genes*

*Libriform fiber bio#1/2/3: 20144/21636/19020 genes*

*Libriform fiber bio#1+2+3: 25457 genes*

*Ray parenchyma bio#1/2/3: 20885/22995/23636 genes*

*Ray parenchyma bio#1+2+3: 27915 genes*

(6) No information is given on formation (or removal) of doublets in the data derived from the 10x sorted cells. Doublet formation becomes an issue when targeting higher number of cells.

*[Author Response]*

*Doublet annotation has been a very challenging task during scRNA-seq analysis, especially in homotypic datasets (cells from a same species) (PMID: 33338399). A recent study (PMID: 33338399, Cell Systems, February 2021) performed a comprehensive survey on the nine cutting-edge computational doublet-detection platforms, and found diverse performance among these platforms. To put it simply, a droplet would be annotated as a doublet by one platform, but would be annotated as a singlet by another platform. We quoted a sentence from such study: “These results demonstrate the general difficulty in detecting doublets from scRNA-seq data and suggest possible room for improvement by future method development.” Thus, doublet removal at current stage would cause unexpected bias to the downstream analysis.*

*To minimize the doublets, we followed the guideline provided by 10x Genomics during sample preparation. We added the following description in the “10x Chromium scRNA-seq” section of Methods: “To minimize the formation of doublets or even multiplets, the number of loaded cells was following the instruction in the manual of 10x Genomics for an estimated multiplet rate less than 8%.” Please see lines 628-630 in the revised manuscript.*

(7) No information exists either on correcting/removing the variation that could exist due to presence of cell cycle or mitochondrial/chloroplast genes. This could lead to removing certain cells and provide a better picture of clustering.

*[Author Response]*

*The mitochondrial and chloroplast genome information is not available in every species in this study, so such examination is not feasible at current stage. To avoid the inclusion of mitochondria and chloroplast as cells, we mapped the reads to genomes followed by an UMI cutoff as 500. If the transcripts (UMIs) from mitochondria or chloroplast were mapped to genomes, these mapped UMIs have to be more than 500 in a type of barcodes to be considered as a cell. Through the genome mapping and UMI cutoff, we minimized the possibility for the inclusion of mitochondria and chloroplast as cells.*

*A previous study reported that cell cycle genes are mainly involved in dividing cells (PMID: 34850215): “cluster 32 (root) and cluster 13a (pooled ED/EN) share 82 marker genes (Jaccard-Score 0.31). Both clusters were assigned to cells that undergo division and, thus, we assumed that those genes might be involved in the regulation of the cell cycle. Indeed, 32 out of these 82 genes can be found in the most enriched GO category “cell cycle” (GO:0007049; adj. P-value: 7.22E−26).” Since SDX cells do not undergo cell division, it seems not necessary to exclude the cell cycle genes in the analysis.*

*In the previous stem scRNA-seq study (PMID: 34809675), the authors found that the cell-cycle gene exclusion software package unexpectedly failed to execute the exclusion of cell cycle genes. We quoted the following sentence from that study: “we still found that the genes involved in cell cycling and division were specifically expressed in clusters 14 and 15”. As a result, the inclusion of cell cycle genes does not seem to perturb cell clustering for protoplasts from stems (SDX and bark).*

*The inclusion and exclusion of cell cycle genes also did not affect the cell clustering of scRNA-seq results in maize root tips (*[*https://doi.org/10.1016/j.cj.2022.02.004):*](https://doi.org/10.1016/j.cj.2022.02.004):) *“To evaluate the impact of cell cycle in clustering, ...” “the effect of cell cycle on clustering was limited.”*

*Based on the description above, we thus did not correct/remove cell-cycle or mitochondrial/chloroplast genes.*

(8) Line 172. is there more evidence for the rather controversial claim that "ray initials can elongate and convert into fusiform initials"?

*[Author Response]*

*The conversion and elongation of ray initials into fusiform initials was reported in many*

*species in angiosperms, such as Cochlospermum (please see Reference#1 below),*

*Gmelina (Reference#2), leaf-bearing cacti (Reference#3) and Cola (Reference#4).*

*Reference#1:* [*https://doi.org/10.1002/j.1537-2197.1940.tb13955.x*](https://doi.org/10.1002/j.1537-2197.1940.tb13955.x)

*Reference#2:* [*https://doi.org/10.1016/S0367-2530(17)30384-5*](https://doi.org/10.1016/S0367-2530(17)30384-5)

*Reference#3:* [*https://doi.org/10.5962/p.185641*](https://doi.org/10.5962/p.185641)

*Reference#4:* [*https://doi.org/10.1093/oxfordjournals.forestry.a063360*](https://doi.org/10.1093/oxfordjournals.forestry.a063360)

*However, we removed this sentence (original lines 171-172) due to the lack of evidence*

*to support the “intermix” hypothesis (please see details in Major Comment 4).*

(9) Lines 321-322. this notion is unnecessary as primary roots of 5-day old Arabidopsis seedlings don't have fibers.

*[Author Response]*

*We revised the sentence as suggested, which now reads: “In addition, almost no Arabidopsis xylem cells co-localize with the cell-type determined libriform fiber cluster (Fig. 3D(iv), Additional file 1: Fig. S30A(iv)), which is consistent to the absence of libriform fibers of their source tissue as the primary roots in 5-day-old Arabidopsis [60-*

*64].” Please see lines 379-382 in the revised manuscript.*

(10) Lines 485-492. The discussion on Sanyos four, and the "mother cells" is a bit outdated now that it seems that cambium has one initial which divides most frequently and that there does not seem to exist any "mother cells". This comment concerns also nomenclature in Fig S16.

*[Author Response]*

*We thank the information provided by the Reviewer, and agree with the Reviewer’s suggestion. Many studies have reported that cambium has only a single initial, and the lack of proliferative progenitor cells makes the term “mother cells” outdated. We thus removed the paragraph in Discussion (original lines 485-502), and also removed the related figure (original Fig. S33).*

(11) Lines 591-594.

(11-1) Is the data for T. aralioides from the winter conditions included somewhere?

*[Author Response]*

*Yes, total four trees were used in winter for scRNA-seq as second batch, and the data*

*was used to examine the reproducibility (overlapping rate as 100% in Fig. S35D).*

(11-2) Figure 1 O-R please indicate the gene models in the figure legend.

*[Author Response]*

*We have indicated the gene models in the legends of Fig. 1O-R as suggested.*

(11-3) In general, it would be great to see annotations for genes presented in all figures.

*[Author Response]*

*We have annotated the genes and provided the gene IDs in all figures as suggested. The functional annotations of many genes are not available, which then remained blank on the figures.*

(11-4) Figure S2 should be updated. For instance, the Populus publications are missing here.

*[Author Response]*

*We incorporated the two Populus publications into Fig. S2 as suggested (Li et al., 2021;*

*Chen et al., 2021).*

**Second round of review**

**Reviewer 1**

The authors have addressed all my concerns. I have no further comment.

**Reviewer 2**

My concerns have been addressed.

I’ve carefully read the answers to reviewer 3, again, and to the best of my knowledge these concerns are addressed faithfully. In fact, I was impressed by the detail of the answers and revisions.
